# An anti-ANGPTL3/8 antibody decreases circulating triglycerides by binding to a LPL-inhibitory leucine zipper-like motif

**DOI:** 10.1016/j.jlr.2022.100198

**Published:** 2022-03-17

**Authors:** Deepa Balasubramaniam, Oliver Schroeder, Anna M. Russell, Jonathan R. Fitchett, Aaron K. Austin, Thomas P. Beyer, Yan Q. Chen, Jonathan W. Day, Mariam Ehsani, Aik Roy Heng, Eugene Y. Zhen, Julian Davies, Wolfgang Glaesner, Bryan E. Jones, Robert W. Siegel, Yue-Wei Qian, Robert J. Konrad

**Affiliations:** Lilly Research Laboratories, Eli Lilly and Company, Indianapolis, IN, USA

**Keywords:** lipoprotein lipase (LPL), triglycerides (TG), angiopoietin-like protein (ANGPTL), apolipoprotein (Apo), hydrogen-deuterium exchange mass spectrometry (HDXMS), leucine zipper, nano-imaging, molecular modeling, epitopes, transmission electron microscopy (TEM), ApoA5, apolipoprotein A5, CCD, coiled coil domain, EL, endothelial lipase, FLD, fibrinogen-like domain, HDXMS, hydrogen-deuterium exchange mass spectrometry, PL, phospholipid, TEM, transmission electron microscopy

## Abstract

Triglycerides (TG) are required for fatty acid transport and storage and are essential for human health. Angiopoietin-like-protein 8 (ANGPTL8) has previously been shown to form a complex with ANGPTL3 that increases circulating TG by potently inhibiting LPL. We also recently showed that the TG-lowering apolipoprotein A5 (ApoA5) decreases TG levels by suppressing ANGPTL3/8-mediated LPL inhibition. To understand how LPL binds ANGPTL3/8 and ApoA5 blocks this interaction, we used hydrogen-deuterium exchange mass-spectrometry and molecular modeling to map binding sites of LPL and ApoA5 on ANGPTL3/8. Remarkably, we found that LPL and ApoA5 both bound a unique ANGPTL3/8 epitope consisting of N-terminal regions of ANGPTL3 and ANGPTL8 that are unmasked upon formation of the ANGPTL3/8 complex. We further used ANGPTL3/8 as an immunogen to develop an antibody targeting this same epitope. After refocusing on antibodies that bound ANGPTL3/8, as opposed to ANGPTL3 or ANGPTL8 alone, we utilized bio-layer interferometry to select an antibody exhibiting high-affinity binding to the desired epitope. We revealed an ANGPTL3/8 leucine zipper-like motif within the anti-ANGPTL3/8 epitope, the LPL-inhibitory region, and the ApoA5-interacting region, suggesting the mechanism by which ApoA5 lowers TG is via competition with LPL for the same ANGPTL3/8-binding site. Supporting this hypothesis, we demonstrate that the anti-ANGPTL3/8 antibody potently blocked ANGPTL3/8-mediated LPL inhibition in vitro and dramatically lowered TG levels in vivo. Together, these data show that an anti-ANGPTL3/8 antibody targeting the same leucine zipper-containing epitope recognized by LPL and ApoA5 markedly decreases TG by suppressing ANGPTL3/8-mediated LPL inhibition.

The ability to regulate triglyceride (TG) metabolism to deliver FAs to tissues when and where they are needed is essential for health and survival. Control of TG metabolism is extremely complicated, and it is increasingly being recognized that tissue-specific regulation of the enzyme LPL plays a crucial role (1). LPL hydrolyzes TG, giving rise to FA that can then be taken up into the tissues where LPL is active (2, 3, 4). In the adipose tissue, uptake of FA leads to their re-esterification and storage as TG, whereas in oxidative tissues such as skeletal muscle, FAs are more likely to be used for energy (2, 3, 4). It is known that LPL itself is expressed in adipocytes and myocytes and that its transport from the outer surface of these parenchymal cells to the luminal surface of the local capillary endothelium is mediated by the protein glycosylphosphatidylinositol-anchored HDL-binding protein 1 (GPIHBP1) (5, 6). This step is essential for LPL to become anchored to the endothelial surfaces of capillaries perfusing the local tissues where FA uptake is required. A number of additional proteins, however, are involved in regulating the local activity of LPL, and the complex interplay between these various proteins and LPL itself is only partially understood (1, 2, 3, 4).

On one hand are the apolipoproteins ApoC1, ApoC2, and ApoC3. These proteins circulate at μg/ml levels. ApoC2 is believed to be a required cofactor for LPL activation, while ApoC1 and especially ApoC3 are believed to be inhibitors of LPL (7, 8, 9). On the other hand, the angiopoietin-like (ANGPTL) proteins ANGPTL3, ANGPTL4, and ANGPTL8 all circulate at ng/ml concentrations (10, 11). In particular, the most recently discovered ANGPTL protein, ANGPTL8, has become recognized as a critical regulator of circulating TG concentrations (12, 13). This protein was first characterized as an unusual ANGPTL protein, which lacked the C-terminal fibrinogen-like domain (FLD) present in ANGPTL3 and ANGPTL4 and which increased TG levels in a manner that was dependent upon ANGPTL3 (14, 15, 16, 17). Subsequently, it was suggested that ANGPTL8 in fact required ANGPTL3 for its optimal expression and needed to work together with ANGPTL3 to achieve functional inhibition of LPL activity (18, 19, 20, 21).

We recently demonstrated that ANGPTL8 is an insulin-responsive protein that forms distinct complexes with ANGPTL3 and ANGPTL4 in order to markedly increase or decrease their respective LPL-inhibitory activities (22). The ANGPTL3/8 complex manifests greatly increased LPL-inhibitory activity relative to ANGPTL3 alone and acts largely in an endocrine manner to inhibit LPL in skeletal muscle so that TG are routed toward the fat after a meal. In contrast, the ANGPTL4/8 complex manifests much less LPL-inhibitory activity than ANGPTL4 and acts in a more localized manner in the adipose tissue through reduction of localized ANGPTL4-mediated LPL inhibition in order to preserve LPL activity needed for postprandial uptake of FA into the fat. These properties of ANGPTL8 allow for increased postprandial LPL-inhibition in muscle and decreased postprandial LPL inhibition in fat so that the utilization of FA can be responsive to caloric intake (22, 23).

Connecting these two families of proteins is the unusual apolipoprotein A5 (ApoA5), which lowers TG while circulating at ng/ml levels, which are much lower than those of other apolipoproteins but still higher than those of the ANGPTL proteins (24, 25). While it was discovered in 2001, the exact mechanism of action of ApoA5 had remained uncertain, although it was agreed upon very quickly that it played an important role in lowering TG (26, 27). ApoA5 overexpression studies in mice resulted in marked lowering of circulating TG, while ApoA5 knockout mice had dramatically increased serum TG (28, 29, 30, 31). Similarly, when ApoA5 and ApoC3 were either both knocked out or both overexpressed, the result was relatively normal circulating TG levels, even though ApoC3 levels during the overexpression experiments were much greater than those of over-expressed ApoA5 (32). In addition, ApoA5 mutations correlated with TG levels in human subjects (33, 34, 35, 36), and ApoA5 mRNA and protein levels were shown to be increased by peroxisome proliferator-activated receptor-α agonists such as fenofibrate, which are known to decrease serum TG (37, 38, 39).

In spite of these early data, however, there was no clear agreement about how an apolipoprotein like ApoA5 that circulated at ng/ml levels could act so potently to decrease TG. As a result, the three main hypotheses that emerged over the ensuing years for its mechanism of action, including stimulating LPL directly, facilitating hepatic lipoprotein particle uptake, or decreasing the secretion of TG-containing lipoprotein particles by the liver, all proved rather unsatisfying (40, 41, 42, 43). Very recently, we were able to show that ApoA5 actually works by specifically suppressing the LPL-inhibitory activity of the ANGPT3/8 complex (44). This discovery, which provided the first direct connection between apolipoprotein biology and ANGPTL protein biology, had two important implications. The first was that ApoA5 lowers TG by binding to the ANGPTL3/8 complex and thus essentially acts as an endogenous inhibitor of ANGPTL3/8. The second was that if ApoA5 reduces TG levels by inhibiting ANGPTL3/8 but does so while circulating at molar concentrations 50-100-fold higher than those of ANGPTL3/8, then a neutralizing, high-affinity therapeutic anti-ANGPTL3/8 antibody should potently reduce TG levels.

In light of these implications, we had three main goals for our current study. Our first goal was to develop a novel therapeutic anti-ANGPTL3/8 antibody that would be capable of blocking the binding of the ANGPTL3/8 complex to LPL. We used ANGPTL3/8 as an immunogen to develop such an antibody that recognized only ANGPTL3/8 (but not ANGPTL3 or ANGPTL8) and bio-layer interferometry and surface plasmon resonance to select a high-affinity antibody binding the desired epitope. Our second goal was to understand the molecular mechanisms by which LPL, ApoA5, and the anti-ANGPTL3/8 antibody bind to ANGPTL3/8. In particular, we wanted to know what epitopes on the ANGPTL3/8 complex were bound by LPL, ApoA5, and the anti-ANGPTL3/8 antibody and whether or not these were distinct epitopes. To accomplish this goal, we used hydrogen-deuterium exchange mass spectrometry (HDXMS) and molecular modeling to map the exact site on the ANGPTL3/8 complex to which LPL, ApoA5, and the anti-ANGPTL3/8 antibody bind. Our third and final goal was to determine if our anti-ANGPTL3/8 antibody would block the inhibition of LPL by ANGPTL3/8 in vitro and potently lower circulating TG in vivo. To accomplish this goal, we characterized our antibody in LPL activity assays and administered it to hypertriglyceridemic mice (45) in order to determine its ability to reduce serum TG levels.

## Materials and methods

### Generation of recombinant proteins and complexes

Human ANGPTL3/8, ANGPTL3, ApoA5 (HSA-tagged), and mouse ANGPTL3/8 were generated as previously described (22, 44). Human ANGPTL8 was produced as HIS-HSA-ANGPTL8 transiently in HEK293 cells utilizing the same mammalian expression construct as the ANGPTL3/8 complex (22). The HIS-HSA-ANGPTL8 protein was purified through nickel-nitrilotriacetic acid affinity, followed by size-exclusion chromatography but not subjected to further proteolysis to remove the HSA fusion partner as was done when generating the ANGPTL3/8 complex. Mouse ANGPTL3 (NP_038941.1) was produced the same way as human ANGPTL3 (22). Cynomolgus monkey ANGPTL3/8 (XP_005543242.1/XP_005588064.1) was produced in the same way as human ANGPTL3/8 (22). Human GPIHBP1-LPL was produced in HEK293 cells through transient cotransfection. Nucleotide sequences encoding mouse IgG kappa signal peptide-HIS tag-mature human LPL (residues 28-475, NP_000228.1) were inserted into a mammalian expression vector containing a cytomegalovirus promoter, as were nucleotide sequences encoding GPIHBP1(1-151 R58G) (residues 1-151 with R58 changed to G58, NP_835466.2). This residue was changed to prevent the internal cleavage reported previously by Mysling et al (46). Protein expression was performed through transient cotransfection of both expression constructs in HEK293 cells cultured in serum-free media. Culture media were harvested 5 days posttransfection, and human LPL/GPIHBP1 complex was purified through nickel-nitrilotriacetic acid affinity, followed by size-exclusion chromatography. Each protein or complex was characterized using gradient gel electrophoresis with Bio-Rad 4%–20% Mini-Protean Tris-glycine gels, followed by Coomassie blue staining to ensure suitability for subsequent experiments. All proteins and complexes were maintained at a <0.01 EU/μg of endotoxin.

### Development of a therapeutic anti-ANGPTL3/8 antibody

Our goal was to develop an anti-ANGPTL3/8 antibody, superior to our previously described anti-ANGPTL3/8 antibody (44), that would be capable of binding ANGPTL3/8 complex with high affinity and that would also be able to completely block the ability of ANGPTL3/8 to bind to and inhibit LPL. To do this, multiple anti-ANGPTL3/8 antibodies were isolated following mouse immunizations with human recombinant ANGPTL3/8 complex, followed by the cloning of their anti-ANGPTL3/8 variable regions. In these experiments, transgenic mice expressing fully human immunoglobulin variable regions (AlivaMab) were immunized with the recombinant human ANGPTL3/8 complex using standard procedures. Three to five days after final nonadjuvant boost, lymph nodes and/or spleens were harvested, and single-cell suspensions were generated. Antigen-specific B-cells were enriched (and ANGPTL3-positive cells were depleted) by standard sorting methods using biotinylated or fluorophore-labeled ANGPTL3/8 (and ANGPTL3), and variable regions were cloned following single-cell PCR amplification. Antibodies were screened using an anti-ANGPTL3/8 capture ELISA. The IgG was captured with a goat anti-human kappa antibody and then screened for the ability to bind biotin-labeled antigen at a final concentration of 25 nM, which in turn was detected by alkaline phosphatase–labeled neutravidin. This enabled the final selection of an anti-ANGPTL3/8 antibody that demonstrated binding to the ANGPTL3/8 complex, with minimal binding to free ANGPTL3 or ANGPTL8. For comparison purposes, the anti-ANGPTL3 antibody evinacumab was produced based on sequences from the International Nonproprietary Names Recommended List 74, with F233A and L234A constant region substitutions to remove residual Fc gamma receptor binding.

### Bio-layer interferometry binding assessments

The interactions of the ANGPTL3/8 with LPL complexed with GPIHBP1 in the absence or presence of the anti-ANGPTL3/8 specific antibody were assessed with bio-layer interferometry using Octet RED96e® (Molecular Devices). To confirm first the specificity of the anti-ANGPTL3/8 antibody for the ANGPTL3/8 complex versus free ANGPTL3 or ANGPTL8, the antibody was immobilized on streptavidin biosensors, incubated with ANGPTL3, ANGPTL8, or ANGPTL3/8 (5 μg/ml of each) and transferred into buffer-only wells to monitor dissociation. To study the interaction of ANGPTL3/8 with GPIHBP1-LPL, avidin-tagged LPL copurified with GPIHBP1 was first immobilized on a streptavidin biosensor. After washing, the biosensor was incubated with ANGPTL3/8 complex that was preincubated in the absence or presence of either the anti-ANGPTL3/8-specific antibody or an irrelevant, isotype-matched control antibody. The ANGPTL3/8 complex and antibodies were preincubated for 30 min at room temperature at a molar ratio of 10:1 antibody:ANGPTL3/8. The association of ANGPTL3/8 with GPIHBP1-LPL was monitored. Following the association phase, the biosensors were transferred into buffer-only wells to monitor the dissociation of any bound ANGPTL3/8 from the GPIHBP1-LPL.

### HDXMS experiments

HDXMS experiments were performed using a decoupled system as previously described (47) with some modifications. Experiments were conducted at pH 7.4, in 10 mM sodium phosphate buffer, containing 150 mM NaCl (1x PBS buffer) in triplicate. Peptide identifications for all proteins were performed on a Waters Synapt G2Si instrument (Waters Corporation) at zero exchange (1:10 dilution in 1X PBS) using nepenthesin II (Nep II) for digestion. Final molar concentrations of the interacting proteins were optimized to provide the best conditions for the experiments and were as follows: ANGPTL3, 15 μM; ANGPTL8, 15 μM; ANGPTL3/8, 12 μM; LPL, 18 μM; ApoA5, 18 μM; and anti-ANGPTL3/8 antibody, 15 μM. Experiments were initiated by adding 25 μl of D_2_O buffer containing 1x PBS to 2.5 μl of protein at 15°C for various amounts of time (10 s, 2 min, 10 min, and 60 min) using a custom TECAN sample preparation system. The reaction was quenched using an equal volume of 0.32 M TCEP, 3 M guanidine HCl, 0.1 M phosphate pH 2.5 for two minutes at 4°C and immediately stored at −70°C. The sample injection system was comprised of a UR3 robot, a LEAP PAL3 HDX autosampler, and a HPLC system interfaced with a Waters Synapt G2Si. The LC mobile phases consisted of water (A) and acetonitrile (B), each containing 0.2% formic acid. Each sample was thawed using 50 μl of 1.5 M guanidine HCl, 0.1 M phosphate pH 2.5 for 1 min, and injected on to a Nep II column for digestion at 4°C with mobile phase A at a flow rate of 250 μl/min for 2.5 min. The resulting peptides were trapped on a Waters BEH Vanguard precolumn at 4°C, and chromatographically separated using a Waters Acquity UPLC BEH C18 analytical column at 4°C with a flow rate of 200 μl/min and a gradient of 3%–85% mobile phase B over 7 min and directed into the mass spectrometer for mass analysis. The Synapt G2Si was calibrated with Glu-fibrinopeptide (Waters Corporation) prior to use. Mass spectra were acquired over the m/z range of 255–1950, with the lock mass m/z of 556.2771 (Leucine Enkephalin, Waters Corporation). The relative deuterium incorporation for each peptide was determined by processing the MS data for deuterated samples along with an nondeuterated control using the identified peptide list in DynamX 3.0 (Waters Corporation). Using these methods, we compared the ANGPTL3/8 complex with ANGPTL3 and ANGPTL8 proteins. We also characterized binding of the anti-ANGPTL3/8 antibody, GPIHBP1-LPL, and ApoA5 to the ANGPTL3/8 complex.

### Negative stain transmission electron microscopy and molecular modeling

ANGPTL3, ANGPTL8, and an anti-ANGPTL3/8 antibody Fab were cotransfected in CHO cells to generate an ANGPTL3/8-Fab complex for negative stain transmission electron microscopy (TEM) experiments. Samples at 1 mg/l were diluted 100-fold into TBS and placed into grids for application and staining with 3 μl of uranyl formate. Negative stain TEM nanoimaging was performed at a magnification of 1,100,00× using a FEI Tecnai T12 electron microscope followed by 2D averaging analysis (NanoImaging Services, Inc, San Diego, CA). Images were subjected to 2D averaging in three rounds. In the process of 2D averaging, individual particles were selected from TEM images, aligned relative to each other, and then computationally classified based on apparent shape similarities. Images from negative stain TEM were used to build three-dimensional models for ANGPTL3, ANGPTL8, and the ANGPTL3/8 complex.

### Structural studies and modeling

For molecular modeling purposes (including minimization, dynamic simulations, and protein model building), we used a Molecular Operating Environment and Discovery Studio (BIOVIA). To build our structural models, we searched publicly available databases for possible similarities by sequence homology but did not find suitable templates. Therefore, we utilized secondary structure predictions to identify templates for modeling. We also reviewed the available literature (48, 49, 50), which indicated that the N-terminal regions of ANGPTL3 and ANGPTL8 both take on coiled coil domain (CCD) structures. We then used the Discovery Studio secondary structure prediction protocol to determine that our templates for both proteins should contain consecutive helices forming CCD structures spanning approximately 20 nm. In addition to the secondary structure predictions, we tested the ANGPTL3 sequence for the presence of CCDs characterized by a sequence motif of hydrophobic (leucine and isoleucine) and polar residues and used the LOGICOIL algorithm to aid in the prediction of multiple coiled-coil oligomeric states based on protein-sequence data (51). To develop models for the ANGPTL3 CCD and ANGPTL8 CCD, we used the free, online version of AI-based AlphaFold2, which utilizes computational methods that can predict protein structures with atomic accuracy even when no similar structure is known (52, 53, 54). Reassuringly, we found models created by AlphaFold2 contained CCD folds for the N-terminal domains of ANGPTL3 and ANGPTL8 and therefore used structures based on AlphaFold2 for our modeling.

ANGPTL3 is a 460-amino acid protein, with an N-terminal CCD consisting of amino acids 17–210 attached by a 30-amino acid linker to a C-terminal FLD consisting of amino acids 241–460 (55, 56, 57). The structure of the ANGPTL3 FLD has been solved by X-ray diffraction and previously described (55) as a FLD trimer, consistent with our own and other’s previous observations (22, 44, 56). Our secondary structure prediction for the N-terminal domain indicated a helical structure, consistent with reports that the N-terminal region forms a CCD containing the LPL-inhibitory motif (58, 59, 60). As there is no available crystal structure for the ANGPTL3 CCD, we developed a model for it using AlphaFold2. We then used the crystal structure of the ANGPTL3 FLD trimer with three modeled ANGPTL3 CCDs as the basis of our model for ANGPTL3 alone and ANGPTL3 in the ANGPTL3/8 complex.

ANGPTL8 is a 198-amino acid protein containing consecutive alpha helices forming a CCD that is comparable to the size of an ANGPTL3 CCD, according to secondary structure predictions by Discovery Studio. For the CCDs of ANGPTL3 and ANGPTL8, the AlphaFold2 model predicted a complex approximately 20 nm in length (after assembly with the ANGPTL3 FLD trimer). Analyses of the ANGPTL3/8 complex model revealed how the CCDs associate via a leucine zipper made from leucine and isoleucine residues present in the ANGPTL3 and ANGPTL8 N-terminal CCDs. Our subsequent HDXMS and negative stain TEM data then allowed us to verify the model generated by AlphaFold2 for ANGPTL3 and ANGPTL8 in an ANGPTL3/8 complex.

We found that the interacting areas of ANGPTL3 and ANGPTL8 CCDs as determined by HDXMS were in good agreement with our model and that all dimensions of the TEM-identified shapes corresponded well to the model. The next step was to develop a model for binding of our anti-ANGPTL3/8 antibody Fab (whose structure was internally available to a resolution of 1.8 Å) to the ANGPTL3/8 complex. Images obtained by negative stain TEM confirmed the location and the angle of the bound Fab versus the rod-like structure of the three ANGPTL3 CCDs and the ANGPTL8 CCD present in the ANGPTL3/8 complex. These images, together our HDXMS data, allowed us to build a comprehensive model for the anti-ANGPTL3/8 antibody Fab binding to ANGPTL3/8. Similarly, we utilized the recently described crystal structure of GPIHBP1-LPL (61) to develop a model for binding of the ANGPTL3/8 complex to GPIHBP1-LPL. Our HDXMS data indicated that the anti-ANGPTL3/8 antibody epitope is located at the interface of the ANGPTL3 and ANGPTL8 CCDs and overlaps with the anti-ANGPTL3/8 antibody epitope.

### Human and mouse LPL stable expression cell lines and LPL activity assays

Human LPL activity assays were performed as previously described with minor modifications (22). After overnight incubation of the human LPL stable expression cell line in growth medium, the medium was replaced with 80 μl of medium containing human ANGPTL3/8 complex or human ANGPTL3 at their respective IC_80_ concentrations (2.5 nM or 60 nM) that were each preincubated with increasing concentrations of the anti-ANGPTL3/8 antibody or evinacumab. Cells were incubated for 1-h at 37°C before 20 μl of 5X working solution, freshly prepared with 0.05% Zwittergent detergent 3-(N,N-dimethyl-octadecylammonio)-propanesulfonate (Sigma) and containing EnzChek lipase substrate BODIPY-dabcyl-labeled TG analog (Invitrogen), were added to achieve a final concentration of 1 μM. Fluorescence was monitored at 1 and 30 min at 37°C with a Synergy Neo2 plate reader with an excitation wavelength of 485 nm and emission wavelength of 516 nm. Readings were taken at 1 min and after 30 min, with the 1-min reading subtracted from the 30-min reading to correct for background.

Mouse LPL activity assays were performed as previously described with minor modifications (44). After overnight incubation of the mouse LPL stable expression cell line in growth medium, the medium was replaced with 80 μl of medium containing mouse ANGPTL3/8 complex or mouse ANGPTL3 at their respective IC_80_ concentrations (0.5 nM or 2 nM) that were each pre-incubated with increasing concentrations of the anti-ANGPTL3/8 antibody or evinacumab. Cells were incubated for 1-h at 37°C before 20 μl of 5X working solution, freshly prepared with 0.05% Zwittergent detergent 3-(N,N-dimethyl-octadecylammonio)-propanesulfonate (Sigma) and containing EnzChek lipase substrate BODIPY-dabcyl-labeled TG analog (Invitrogen), were added to achieve a final concentration of 1 μM. Fluorescence was monitored at 1 and 30 min at 37°C with a Synergy Neo2 plate reader with an excitation wavelength of 485 nm and emission wavelength of 516 nm. Readings were taken at 1 and 30 min, with the 1-min reading subtracted from the 30-min reading to correct for background.

### Endothelial lipase stable expression cell line and endothelial lipase activity assay

A human endothelial lipase (EL) activity assay was optimized to allow measurement of the phospholipase A1 activity of EL, which preferentially hydrolyzes phospholipids (PLs) at the *sn*-1 position and was performed as previously described (62), with some minor modifications. After overnight incubation of EL-stable expression cells at 37°C, the medium was replaced with 80 μl of medium (OptiMEM, Invitrogen) containing human ANGPTL3/8 complex or human ANGPTL3 at their respective IC_80_ concentrations (38 nM or 328 nM) that were each preincubated with increasing concentrations of either the anti-ANGPTL3/8 antibody or evinacumab. Cells were incubated for 1 h before adding 20 μl of 5X working solution that was freshly prepared with reaction buffer (50 mM Tris–HCl, 140 mM NaCl, 2 mM CaCl_2_) containing the EnzChek phospholipase A1 selective substrate PED-A1 (N-((6-(2,4-DNP)amino)hexanoyl)-1-BODIPYTM-FL-C5)-2-hexyl-sn-glycero-3-phosphoethanolamine) (Invitrogen), DOPC (1,2-dioleoyl-sn-glycero-3-phosphocholine), and DOPG (1,2-dioleoyl-sn-glycero-3-phospho-rac-(1-glycerol) sodium salt) (Sigma) in order to achieve final concentrations of 1, 10, and 10 μM respectively. The 5X working solution was prepared by mixing one part of 1 mM PED-A1, one part of 10 mM DOPC, and one part of 10 mM DOPG and adding the mixture drop by drop into 197 parts of reaction buffer while mixing in a glass tube. Cells were then incubated at 37°C, and fluorescence was monitored with a Synergy Neo2 plate reader with an excitation wavelength of 485 nm and an emission wavelength of 516 nm. Readings were taken at 1 and 30 min, with the 1-min reading subtracted from the 30-min reading to correct for background fluorescence.

### Evaluation of the anti-ANGPTL3/8 antibody in hypertriglyceridemic mice

All experiments involving animals were approved by the Institutional Animal Care and Use Committee of Eli Lilly and Company, Indianapolis, Indiana. Mice transgenic for human cholesterol ester transfer protein and human apolipoprotein A1 (CETP/ApoA1) (Taconic Cambridge City, IN) that have been described previously (45) were utilized for this study. The mice used were male and 16–20 weeks of age at the beginning of the study. Prior to the start of the study, nonfasted mice were anesthetized with isoflurane, and blood was collected from the retro-orbital sinus. After separation of the serum, TG were measured in serum samples using a Cobas® clinical chemistry analyzer (Roche Basel, Switzerland). Animals were assigned into 5 groups of 7–8 mice per group to yield groups with similar baseline serum TG levels. Either an irrelevant human IgG_4_ isotype-matched control antibody, dosed at 30 mg/kg (n=8), or the ANGPTL3/8 antibody, dosed at 1 mg/kg (n = 7), 3 mg/kg (n = 7), 10 mg/kg (n = 7), or 30 mg/kg (n = 7), was administered to the mice by a single subcutaneous injection. Blood was collected under isoflurane anesthesia from nonfasted animals 1 day, 7 days, 15 days, 21 days, and 28 days after antibody administration. Following blood collection, serum was separated, and TG concentrations were measured using a Roche Cobas® clinical chemistry analyzer.

### Data analysis

A four-parameter logistic nonlinear regression model was used to fit LPL activity assay curves in which the ability of the anti-ANGPTL3/8 antibody to block ANGPTL3/8-mediated LPL inhibition was examined. Significance for the effect of the anti-ANGPTL3/8 antibody or the irrelevant isotype-matched control antibody on circulating TG concentrations in the CETP/A1 mice was calculated using JMP 13 (SAS Cary, NC). A Dunnett’s test was used for each data set to compare each treatment group to the time-matched isotype control, and a *P*-value of less than 0.05 was considered to indicate statistical significance. For HDXMS experiments, relative deuterium uptake was calculated as previously described (63, 64, 65).

## Results

### Development of an anti-ANGPTL3/8 antibody that blocks binding of ANGPTL3/8 to LPL

**Fig. 1:**
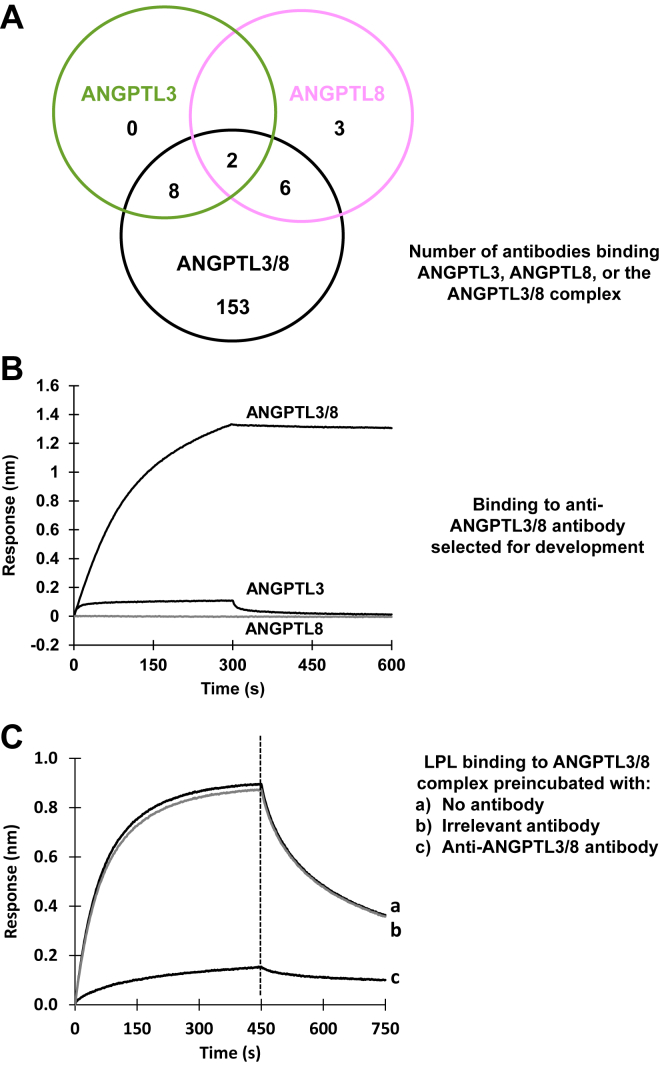
Discovery of an anti-ANGPTL3/8 therapeutic antibody that blocks the binding of ANGPTL3/8 to LPL. A: Anti-human ANGPTL3/8 antibodies isolated following mouse immunizations were profiled for binding to ANGPTL3/8 complex versus ANGPTL3 or ANGPTL8. A large number of individual clones bound specifically to the ANGPTL3/8 complex, with 153 out of the 172 clones tested exhibiting selectivity for ANGPTL3/8 compared to ANGPTL3 or ANGPTL8 alone. B: Biotinylated anti-ANGPTL3/8 antibody immobilized on streptavidin biosensors was incubated with ANGPTL3, ANGPTL8, or ANGPTL3/8 and transferred to buffer-only wells to monitor dissociation. The results obtained confirmed the specific binding of the antibody to ANGPTL3/8 and not ANGPTL3 or ANGPTL8. The results are representative of 3 independent experiments. C: Biolayer interferometry was used to examine the binding of ANGPTL3/8 to GPIHBP1-LPL in the absence or presence of the anti-ANGPTL3/8 antibody. Avidin-tagged LPL complexed with GPIHBP1 was first immobilized on streptavidin biosensors and incubated with ANGPTL3/8 that had been preincubated with no antibody (trace a), an irrelevant isotype-matched control antibody (trace b), or the anti-ANGPTL3/8 antibody (trace c). The biosensors were transferred to buffer-only wells to monitor dissociation. The anti-ANGPTL3/8 antibody almost completely blocked the binding of ANGPTL3/8 to GPIHBP1-LPL. The results are representative of 3 independent experiments.

To understand better the biology of the ANGPTL3/8 complex and identify potential therapeutically intervening molecules, we first aimed to generate an anti-ANGPTL3/8 antibody capable of selectively and potently blocking the binding of the ANGPTL3/8 complex to LPL. To do this, we used recombinant ANGPTL3/8 complex as an immunogen for mouse immunizations, enriched antigen-specific B-cells by FACS, and cloned the variable regions for CHO cell expression. We then screened the supernatants after transient transfection by single point ELISA for clones that specifically bound to the ANGPTL3/8 complex, but not free ANGPTL3 or ANGPTL8. Discovery of antibodies that bind specifically to protein complexes without any affinity to the parent proteins alone is notoriously difficult. This is because it requires complex-specific continuous epitopes formed by the joining proteins, while the majority of antibodies will bind to the more common epitopes presented by the individual, free proteins. Remarkably, however, in the initial single point ELISA screen, we found a large number of clones that appeared to bind exclusively to the complex. In fact, 153 of 172 clones tested by single point ELISA exhibited selectivity for the ANGPTL3/8 complex with no detectable binding to either ANGPTL3 or ANGPTL8 alone (Fig. 1A). Following sequence analysis and in vitro functional characterization (by LPL inhibition assays) of all of the possible antibodies, we selected the single best anti-ANGPTL3/8 antibody for further study.

To confirm that this antibody was specific for ANGPTL3/8, we immobilized the antibody on biosensors, incubated it with ANGPTL3, ANGPTL8, or ANGPTL3/8, and monitored the dissociation. These experiments, shown in Fig. 1B, confirmed the specific binding of the antibody to the ANGPTL3/8 complex, with negligible binding to ANGPTL3 or ANGPTL8 alone. We also examined the binding of the ANGPTL3/8 complex to LPL to determine if this binding could be blocked by the anti-ANGPTL3/8 antibody. To do this, avidin-tagged LPL copurified with GPIHBP1 was first immobilized on the streptavidin biosensor, and its association with the biosensor was monitored. The biosensor was then washed for a brief period of time to remove any unbound LPL. After this washing step, ANGPTL3/8 complex that had been previously preincubated with no antibody, the anti-ANGPTL3/8 antibody, or an irrelevant isotype-matched control antibody was added, and the association of the ANGPTL3/8 complex with GPIHBP1-LPL was monitored. The biosensor was then transferred into buffer-only wells to monitor the dissociation of any ANGPTL3/8 that had bound to the immobilized LPL. As Fig. 1C shows, the anti-ANGPTL3/8 antibody blocked the binding of ANGPTL3/8 to GPIHBP1-LPL while the irrelevant isotype-matched control antibody had no effect. As this figure shows, in the presence of no antibody or the irrelevant isotype-matched control antibody, ANGPTL3/8 showed a prominent association with LPL consistent with our previous observations (22). In contrast, after preincubation of ANGPTL3/8 with the anti-ANGPTL3/8 antibody, binding of ANGPTL3/8 to LPL was almost completely inhibited.

We also confirmed ANGPTL3/8-specific binding for our antibody in a concentration-dependent manner by ELISA, which demonstrated no detectable binding to free ANGPTL3 or ANGPTL8 up to antigen concentrations of 100 nM (Table 1). In addition, we determined the IC_50_ and maximal derepression capability for the anti-ANGPTL3/8 antibody against human, mouse, and cynomolgus monkey ANGPTL3/8 complex-mediated LPL inhibition. As Table 2 shows, the anti-ANGPTL3/8 antibody was a potent inhibitor of each species’ ANGPTL3/8 complex.

**Table 1:** Binding of the ANGPTL3/8 antibody to ANGPTL3/8 complex versus ANGPTL3 or ANGPTL8

Antigen (nM)	ANGPTL8 Alone		ANGPTL3 Alone		ANGPTL3/8 Complex	
	Antibody	Control	Antibody	Control	Antibody	Control
100	0.072	0.076	0.081	0.091	2.705	0.063
33.3	0.050	0.060	0.052	0.053	2.411	0.056
11.1	0.45	0.051	0.046	0.052	1.708	0.047
3.7	0.051	0.48	0.046	0.052	1.042	0.046
1.23	0.043	0.048	0.043	0.052	0.510	0.046
0.41	0.046	0.50	0.043	0.067	0.231	0.046
0.137	0.047	0.053	0.048	0.055	0.110	0.044
0.046	0.051	0.054	0.060	0.069	0.078	0.053

The ability of the anti-ANGPTL3/8 antibody to selectively bind ANGPTL3/8 complex versus ANGPTL3 or ANGPTL8 was determined using an ELISA in which the labeled antibody was tested for binding to increasing concentrations of the three different antigens. Units of measurement shown are absorbance values (A_560_).

**Table 2:** IC_50_ and maximal derepression data for the anti-ANGPTL3/8 antibody

LPL	ANGPTL3/8 Complex	IC_50_ (nM)	Maximal Derepression (%)
Mouse	Mouse	1.38	103
Monkey	Monkey	1.31	96
Human	Human	0.28	102

The anti-ANGPTL3/8 antibody was tested for its ability to derepress the inhibition of various species’ ANGPTL3/8-mediated LPL inhibition observed for each respective LPL.

### ANGPTL3/8 contains a neo-epitope that binds LPL, ApoA5, and the anti-ANGPTL3/8 antibody

**Fig. 2:**
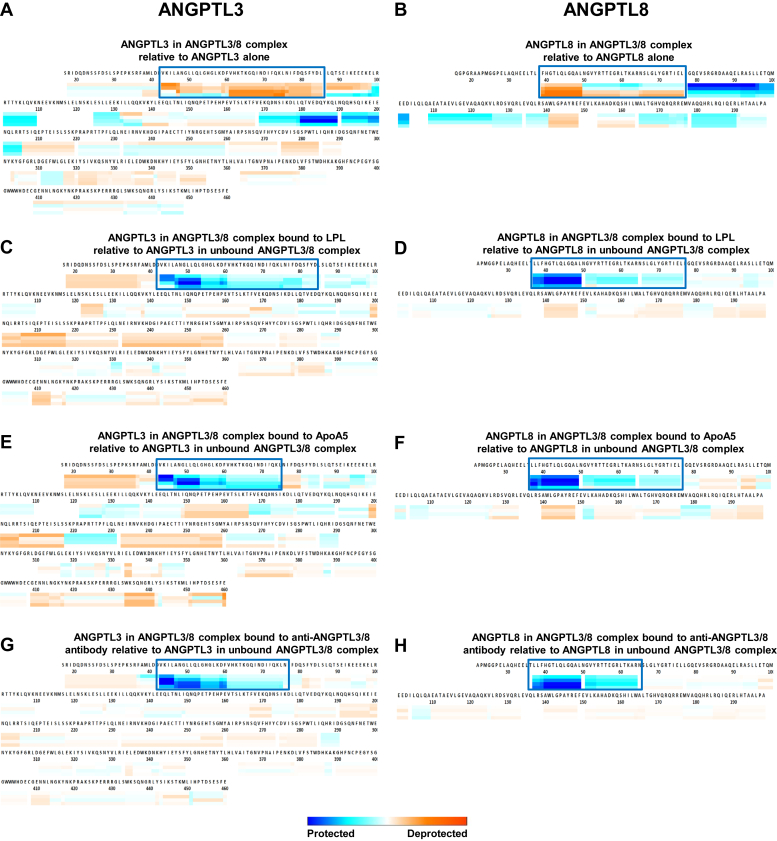
ANGPTL3/8 complex formation creates a neo-epitope recognized by LPL, ApoA5, and the anti-ANGPTL3/8 antibody. Deuterium uptake was measured after 10-s, 2-min, 10-min, and 1-h incubations in deuterated buffer. Boxed areas in Fig. 2A, B show increased HDX occurring in peptide regions comprising the unique epitope formed in the ANGPTL3/8 complex. Boxed areas in Fig. 2C–H show peptide regions with decreased HDX that are protected when the ANGPTL3/8 complex binds LPL, ApoA5, or the anti-ANGPTL3/8 antibody. A: Deuterium uptake into ANGPTL3 complexed with ANGPTL8 relative to deuterium uptake into ANGPTL3 alone. B: Deuterium uptake into ANGPTL8 complexed with ANGPTL3 relative to deuterium uptake into ANGPTL8 alone. C: Deuterium uptake into ANGPTL3 in ANGPTL3/8 complex bound by GPIHBP1-LPL relative to deuterium uptake into ANGPTL3 in unbound ANGPTL3/8 complex. D: Deuterium uptake into ANGPTL8 in ANGPTL3/8 complex bound by GPIHBP1-LPL relative to deuterium uptake into ANGPTL8 in unbound ANGPTL3/8 complex. E: Deuterium uptake into ANGPTL3 in ANGPTL3/8 complex bound by ApoA5 relative to deuterium uptake into ANGPTL3 in unbound ANGPTL3/8 complex. F: Deuterium uptake into ANGPTL8 in ANGPTL3/8 complex bound by ApoA5 relative to deuterium uptake into ANGPTL8 in unbound ANGPTL3/8 complex. G: Deuterium uptake into ANGPTL3 in ANGPTL3/8 complex bound by anti-ANGPTL3/8 antibody relative to deuterium uptake into ANGPTL3 in unbound ANGPTL3/8 complex. H: Deuterium uptake into ANGPTL8 in ANGPTL3/8 complex bound by anti-ANGPTL3/8 antibody relative to deuterium uptake into ANGPTL8 in unbound ANGPTL3/8 complex. ApoA5, apolipoprotein A5.

To understand the structural features of the ANGPTL3/8 complex and map the epitope of the anti-ANGPTL3/8 complex-specific antibody, HDXMS experiments were performed in which ANGPTL3 and ANGPTL8 proteins were exposed to deuterated buffer for short periods of time to map their surface-exposed amides both in isolation as well as in the ANGPTL3/8 complex. These experiments, which were performed in triplicate, revealed protection (decreased HD exchange) in residues 99–109, 124–131, and 170–205 in ANGPTL3, pointing to regions potentially involved in formation of the ANGPTL3/8 complex (Fig. 2A). Similarly, for ANGPTL8, reduced HD exchange was observed in regions 77–103 and 165–190, pointing to regions potentially involved in the interaction with ANGPTL3 during formation of the ANGPTL3/8 complex (Fig. 2B). In addition to the protection observed in the deuterium exchange experiments, both proteins also demonstrated an increase in deuterium exchange in certain regions. ANGPTL3 showed increased deuterium exchange at residues 41–85, as demonstrated in Fig. 2A, and ANGPTL8 showed increased deuterium exchange in residues 39–49 and residues 50–76 show increased exchange at longer exposure times, as shown in Fig. 2B. supplemental Fig. S1A–H show the complete HDXMS dataset from these experiments.

After obtaining these results, we characterized LPL binding to the ANGPTL3/8 complex using HDXMS. In these experiments, deuterium uptake into ANGPTL3 in the ANGPTL3/8 complex when the complex was bound to GPIHBP1-LPL was compared to deuterium uptake into ANGPTL3 in unbound ANGPTL3/8 complex (Fig. 2C). Similarly, deuterium uptake into ANGPTL8 in the ANGPTL3/8 complex when the complex was bound to GPIHBP1-LPL was compared to deuterium uptake into ANGPTL8 in unbound ANGPTL3/8 complex (Fig. 2D). For both proteins, the unique epitope that was exposed upon the formation of the ANGPTL3/8 complex (regions of increased deuterium exchange in Fig. 2A, B) was the same region that was protected from deuterium exchange when GPIHBP1-LPL bound the ANGPTL3/8 complex. supplemental Fig. S2A–H show the complete HDXMS dataset from these experiments.

Similar HDXMS experiments were performed to characterize ApoA5 binding to the ANGPTL3/8 complex. Deuterium uptake into ANGPTL3 in the ANGPTL3/8 complex when the complex was bound to ApoA5 was compared to deuterium uptake into ANGPTL3 in unbound ANGPTL3/8 complex (Fig. 2E). Likewise, deuterium uptake into ANGPTL8 in the ANGPTL3/8 complex when the complex was bound to ApoA5 was compared to deuterium uptake into ANGPTL8 in unbound ANGPTL3/8 complex (Fig. 2F). For both proteins, the unique epitope exposed upon the formation of the ANGPTL3/8 complex was again the same region that was protected from deuterium exchange when ApoA5 bound the ANGPTL3/8 complex. supplemental Fig. S3A–H show the complete HDXMS dataset from these experiments.

Further HDXMS experiments were performed to characterize the binding of the anti-ANGPTL3/8 antibody to the ANGPTL3/8 complex. Deuterium uptake into ANGPTL3 in the ANGPTL3/8 complex when the complex was bound to the anti-ANGPTL3/8 antibody was compared to deuterium uptake into ANGPTL3 in unbound ANGPTL3/8 complex (Fig. 2G). Likewise, deuterium uptake into ANGPTL8 in the ANGPTL3/8 complex when the complex was bound to the anti-ANGPTL3/8 antibody was compared to deuterium uptake into ANGPTL8 in unbound ANGPTL3/8 complex (Fig. 2H). For both proteins, the unique epitope exposed upon the formation of the ANGPTL3/8 complex was once again the same region that was protected from deuterium exchange when the anti-ANGPTL3/8 antibody bound the ANGPTL3/8 complex. supplemental Fig. S4A–H show complete HDXMS data from these experiments. Overall, in our HDXMS experiments, peptide coverage averaged 87% for both ANGPTL3 and ANGPTL8. Detailed coverage maps are shown in supplemental Figs. S1A, B, S2A, B, S3A, B, S4A, B.

For experiments described in supplemental Fig. S1, triplicate measurements at the 10-min exchange time were compared using Welch's *t* test, not requiring equal variance assumption, as previously described by Hageman and Weis (66). The correction method of Benjamini et al. (67) was then utilized to adjust for multiplicity, and significant differences were identified at a 10% false discovery rate, with the analyses performed using GraphPad Prism (supplemental Fig. S5). The results confirmed the previous analyses, as the peptides within the regions described above showed statistically significant differences in uptake between ANGPTL3 in the ANGPTL3/8 complex versus ANGPTL3 alone and ANGPTL8 in the ANGPTL3/8 complex versus ANGPTL8 alone. Furthermore, for all of the HDX experiments shown in supplemental Figs. S1–S4, we calculated the uncertainties for each individual peptide at every time point in order to determine if the peptide regions that we observed changes in were statistically significant as recommended by Weiss (68). supplemental Tables S1–S5 show the uncertainties, changes in deuterium uptake, and whether or not the changes were significant for each of the ANGPTL3 and ANGPTL8 peptides that were followed in all of the experiments. As these tables demonstrate, the uptake changes observed for each of the peptide regions described above were all confirmed to represent significant differences.

Together, these data showed that the ANGPTL3 and ANGPTL8 regions that demonstrated increased deuterium exchange upon formation of the ANGPTL3/8 complex were the same regions that showed protection in the ANGPTL3/8 complex when the complex was bound to GPIHBP1-LPL, ApoA5, or the anti-ANGPTL3/8 antibody. These observations therefore identified the formation of a unique epitope (consisting of ANGPTL3 and ANGPTL8 regions) that was unmasked only in the ANGPTL3/8 complex and which recognized LPL, ApoA5, and the anti-ANGPTL3/8 antibody. In so doing, these data thus also explained how it was possible to develop an anti-ANGPTL3/8 complex-specific antibody.

### Molecular modeling reveals novel leucine zipper-like motifs in the ANGTL3/8 complex

**Fig. 3:**
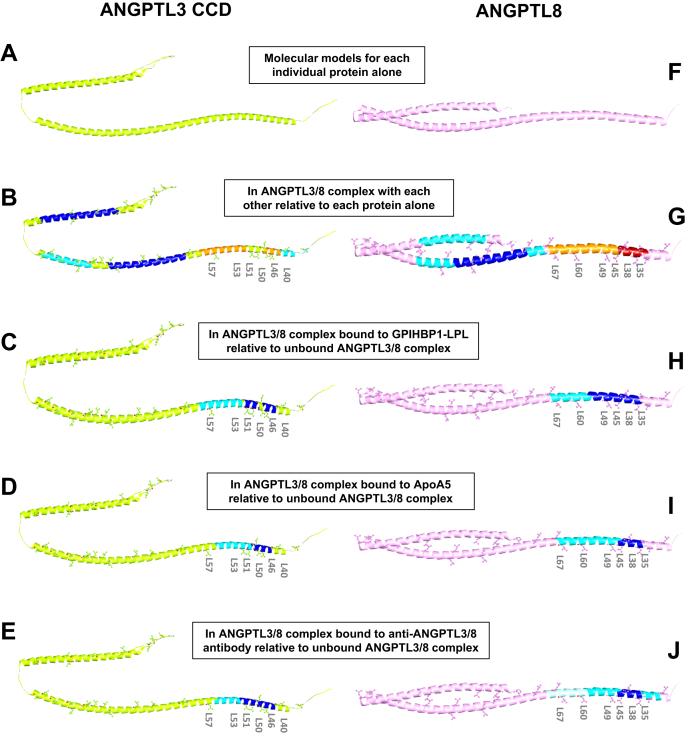
ANGPTL3 CCDs and the ANGPTL8 CCD that become unmasked in the ANGPTL3/8 complex bind LPL, ApoA5, and the anti-ANGPTL3/8 antibody. Blue regions show low HDX, while orange and red regions show high HDX. A: AlphaFold prediction of the ANGPTL3 CCD structure. B: AlphaFold model of the ANGPTL3 CCD in the ANGPTL3/8 complex with mapped HDX relative to ANGPTL3 alone, indicating the presence of a novel leucine zipper-like motif consisting of L40, L46, L50, L51, L53, and L57. C: AlphaFold model of the ANGPTL3 CCD in the ANGPTL3/8 complex bound to GPIHBP1-LPL with mapped HDX relative to unbound ANGPTL3/8 complex. D: AlphaFold model of the ANGPTL3 CCD in the ANGPTL3/8 complex bound to ApoA5 with mapped HDX relative to unbound ANGPTL3/8 complex. E: AlphaFold model of the ANGPTL3 CCD in the ANGPTL3/8 complex bound to the anti-ANGPTL3/8 antibody with mapped HDX relative to unbound ANGPTL3/8 complex. F: AlphaFold prediction of ANGPTL8 structure. G: AlphaFold model of ANGPTL8 in the ANGPTL3/8 complex with mapped HDX relative to ANGPTL8 alone, indicating the presence of a novel leucine zipper-like motif consisting of L35, L38, L45, L49, L60, and L67. H: AlphaFold model of ANGPTL8 in the ANGPTL3/8 complex bound to GPIHBP1-LPL with mapped HDX relative to unbound ANGPTL3/8 complex. I: AlphaFold model of ANGPTL8 in the ANGPTL3/8 complex bound to ApoA5 with mapped HDX relative to unbound ANGPTL3/8 complex. J: AlphaFold model of ANGPTL8 in the ANGPTL3/8 complex bound to the anti-ANGPTL3/8 antibody with mapped HDX relative to unbound ANGPTL3/8 complex. CCD, coiled coil domain.

Based on the LOGICOIL algorithm that predicted a trimer as the most probable ANGPTL3 17–240 sequence structure (51), we modeled the ANGPTL3 CCD trimer (supplemental Fig. S6) using AlphFold2 and observed a constellation of leucine amino acids forming a leucine zipper-like motif in the N-terminal portions of the ANGPTL3 CCDs consisting of L40, L46, L50, L51, L53, and L57 (Fig. 3A). Interestingly, our modeling predicted that these leucine zipper-like motifs are flanked by areas of increased deuterium uptake when ANGPTL3 was complexed with ANGPTL8 (Fig. 3B). Similarly, these same regions demonstrated decreased deuterium uptake when ANGPTL3 present in the ANGPTL3/8 complex was bound by LPL (Fig. 3C), ApoA5 (Fig. 3D), or the anti-ANGPTL3/8 antibody (Fig. 3E).

For the secondary structure prediction of ANGPTL8, we used Discovery Studio to confirm a coiled-coil structure (supplemental Fig. S7). For the ANGPTL8 22–198 sequence, the LOGICOIL algorithm predicted a tetramer as the most preferred assembly and a trimer as the second most likely assembly. As LOGICOIL predictions suggested a tetrameric assembly for ANGPTL8, we realized that the ANGPTL3/8 complex could consist of a tetramer comprised of one ANGPTL8 molecule and one ANGPTL3 trimer (supplemental Fig. S7). Taking into account that multimeric coiled-coil assembly is governed by the positions of the hydrophobic amino acids leucine and isoleucine and the ability of the structure to form conspicuous heptad repeat of hydrophobic and polar residues, we investigated the possibility of a leucine zipper-like motif, which in turn led us to the presence of a novel ANGPTL8 leucine zipper-like motif consisting of L35, L38, L45, L49, L60, and L67 (Fig. 3F). Interestingly, our modeling subsequently predicted that this ANGPTL8 leucine zipper-like was flanked by areas of increased deuterium uptake when ANGPTL8 was complexed with ANGPTL3 (Fig. 3G). Similarly, this same region demonstrated decreased deuterium uptake when ANGPTL8 present in the ANGPTL3/8 complex was bound by either LPL (Fig. 3H), ApoA5 (Fig. 3I), or the anti-ANGPTL3/8 antibody (Fig. 3J). Together, these models show that LPL, ApoA5, and the anti-ANGPTL3/8 antibody all bind to the same N-terminal regions in the CCDs of ANGPTL3 and ANGPTL8 that become unmasked when the ANGPTL3/8 complex is formed.

### Negative stain TEM and modeling ANGPTL3/8 binding the anti-ANGPTL3/8 antibody Fab and LPL

**Fig. 4:**
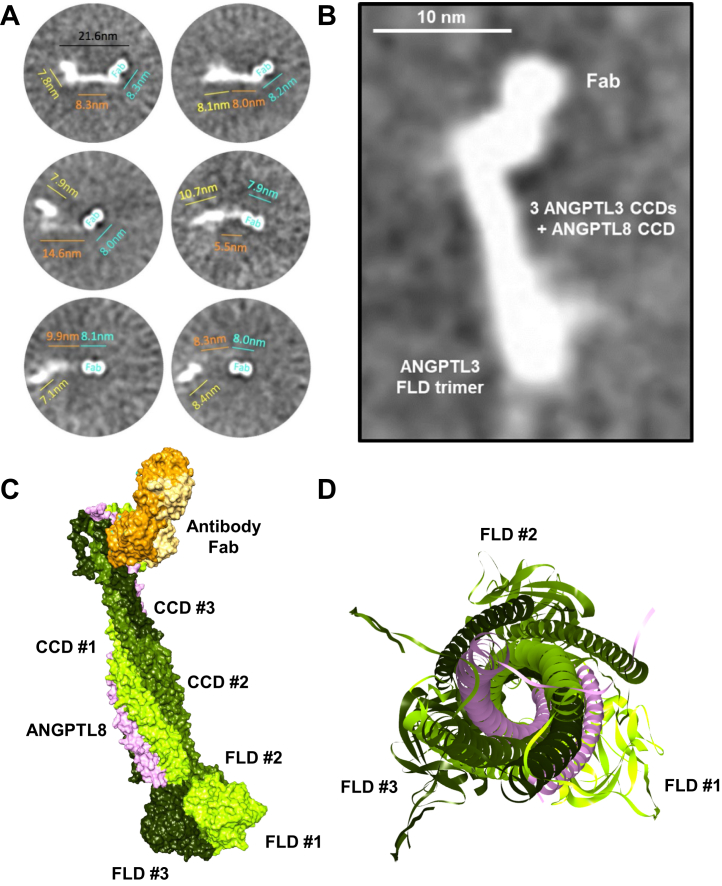
Binding of anti-ANGPTL3/8 antibody Fab to ANGPTL3/8 complex. A: Multiple 2D class averages for the anti-ANGPTL3/8 antibody Fab binding ANGPTL3/8 complex obtained from negative stain TEM experiments show a well-defined approximate 8 nm density with a bi-lobed structure typical for an IgG Fab (cyan). There is an elongated density about 6–15 nm long and approximately 3 nm wide, that is not well-defined (orange), likely consisting of three ANGPTL3 CCDs associated with the ANGPTL8 CCD. Located on the opposite end of the narrow, elongated density, there is a 7–11 nm less well-defined density (yellow), likely consisting of the three ANGPTL3 FLDs. B: Detailed view of a representative thumbnail class average image obtained from the third round of alignments obtained by negative stain TEM. All eight images from this round of alignments are shown in supplemental Fig. S11. The image shown here is that of the first of the eight images (top left image from supplemental Fig. S11). The image is consistent with an ANGPTL3:ANGPTL8 ratio of 3:1 in the ANGPTL3/8 complex. C: A molecular model shows the anti-ANGPTL3/8 antibody Fab binding the ANGPTL3/8 complex. D: Head-on view of the ANGPTL3/8 complex showing interaction of the ANGPTL3 and ANGPTL8 CCDs via leucine zipper motifs. The FLDs of the three ANGPTL3 molecules in the ANGPTL3/8 complex are shown in the background. TEM, transmission electron microscopy; FLDS, fibrinogen-like domains.

We performed negative stain TEM with ANGPTL3/8 in the presence of the anti-ANGPTL3/8 antibody Fab, and images obtained were subjected to 2D averaging in three rounds (supplemental Figs. S8–S11). In these experiments, we observed what appeared to be approximately 20 nm-long string-like particles that had bulky densities at both ends of a narrow center region whose width was roughly 3 nm (Fig. 4A). In some instances, one of the bulky densities had a bi-lobed structure resembling an IgG Fab, while the structure present on the opposite end resembled the trimer of the ANGPTL3 FLDs previously obtained by X-ray crystallography (55). Multiple 2D class averages showed elongated particles that had bulky densities at opposite ends of the rod-like center region. A representative image is shown in Fig. 4B. The smaller, better-defined, bi-lobed bulky density corresponded to the Fab, and the larger, less well-defined bulky density corresponded to the ANGPTL3 FLD trimer. Both densities extended outward and/or upward from the narrow central region. The central region was consistent with the presence of three ANGPTL3 CCDs and the ANGPTL8 CCD winding around each other, with the width of this rod-like structure being around 3 nm, which roughly corresponds to the width of four intertwined CCDs.

The negative stain TEM data thus showed the 55 kD Fab bound to the N-terminal CCDs of ANGPTL3 and ANGPTL8 in the complex. At the opposite end of the ANGPTL3/8 complex to where the Fab binds was a globular structure that was slightly larger than the 55 kD Fab. This represents the three ANGPTL3 FLDs present in the ANGPTL3/8 complex (which would be roughly 24 kD each, for a total of about 72 kD). These observations are thus consistent with our previous reports showing that the ratio of ANGPTL3:ANGPTL8 in the ANGPTL3/8 complex is 3:1 (22, 44). To rule out the possibility that any ANGPTL3 molecules present in the ANGPTL3/8 complex had their N-terminal CCD cleaved, we performed an additional experiment to analyze the ANGPTL3/8 complex via Western blotting with an anti-ANGPTL3 antibody that recognized the ANGPTL3 FLD. In this experiment, we observed only a single band corresponding to full-length ANGPTL3, thus confirming that the three ANGPTL3 molecules in the ANGPTL3/8 complex were intact (supplemental Fig. S12).

The above data enabled us to model the ANGPTL3/8 complex and position the anti-ANGPTL3/8 antibody Fab to match the shapes observed (Fig. 4C). It should be emphasized that we created the best model that we could with the available data, but that our model has inherent limitations based on what can be directly observed versus what is inferred. Despite these challenges, our molecular model showed good consistency with that obtained from the AlphaFold2 modeling for the binding of the anti-ANGPTL3/8 antibody Fab to ANGPTL3/8. This model was consistent with the idea that the anti-ANGPTL3/8 antibody Fab binds to coiled coil regions of ANGPTL3 and ANGPTL8 that become exposed when the ANGPTL3/8 complex forms. Figure 4D shows a head-on model for the ANGPTL3/8 complex demonstrating the interaction of the three CCDs of ANGPTL3 and the ANGPTL8 CCD.

**Fig. 5:**
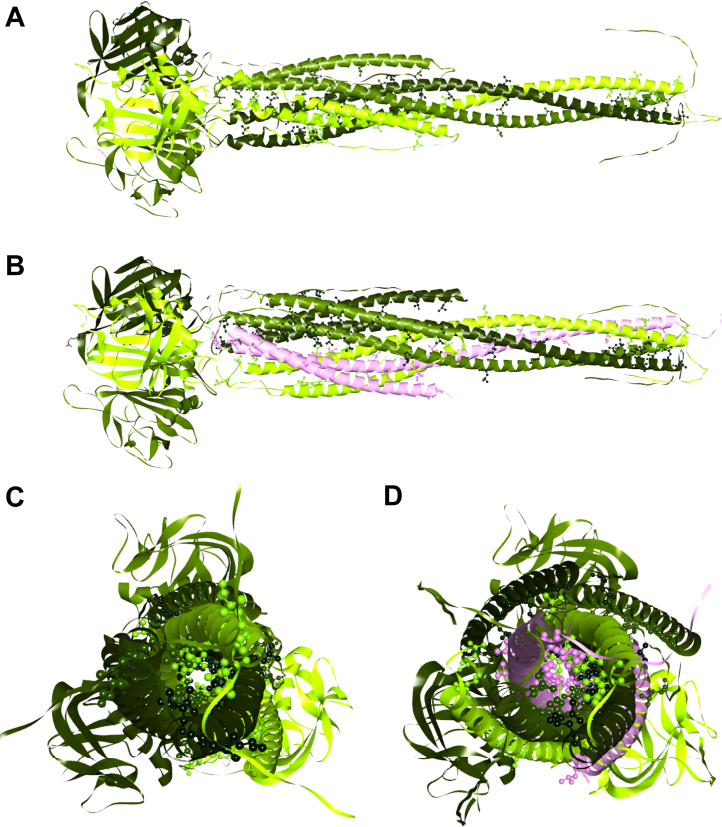
Modeling of ANGPTL3 and the ANGPTL3/8 complex. Leucine and isoleucine residues are highlighted. A: A model for the full-length ANGPTL3 structure made by joining three ANGPTL3 CCDs via amide bonds to the crystal structure of the FLD trimer. B: A model for the ANGPTL3/8 complex showing a tetrameric assembly containing the three CCDs of ANGPTL3 and the single ANGPTL8 CCD, revealing how the three ANGPTL3 CCDs and ANGPTL8 associate together, consistent with the rules of coiled coil multimeric states described by LOGICOIL. C: Head-on view of the ANGPTL3 trimer, showing leucine and isoleucine interactions amongst the three ANGPTL3 CCDs. D: Head-on view of the ANGPTL3/8 tetrameric complex, showing leucine and isoleucine interactions between the ANGPTL8 CCD and the three ANGPTL3 CCDs. CCDS, coiled coil domains.

To confirm the putative, novel leucine zipper-like motifs in the N-terminal regions of the CCDs of ANGPTL3 and ANGPTL8, we added one more constraint to analyze our final model in which the CCD interactions are supported via their leucine zipper-like motifs. We first modeled the full-length ANGPTL3 structure by joining three ANGPTL3 CCDs via amide bonds to the crystal structure of the FLD trimer (55, 56, 57) (Fig. 5A). We then included ANGPTL8 as modeled by AlphaFold2 in a tetrameric assembly with the three CCDs of ANGPTL3 (Fig. 5B). Analysis of the ANGPTL3/8 complex model revealed how the three ANGPTL3 CCDs and ANGPTL8 associate together according to the rules of coiled coil multimeric states as described by LOGICOIL (51). Figure 5C shows a head-on view of the ANGPTL3 trimer, highlighting the leucine and isoleucine interactions between the three ANGPTL3 CCDs. Figure 5D shows a head-on view of the ANGPTL3/8 tetrameric complex, highlighting the leucine and isoleucine interactions between the single ANGPTL8 CCD and the three ANGPTL3 CCDs.

**Fig. 6:**
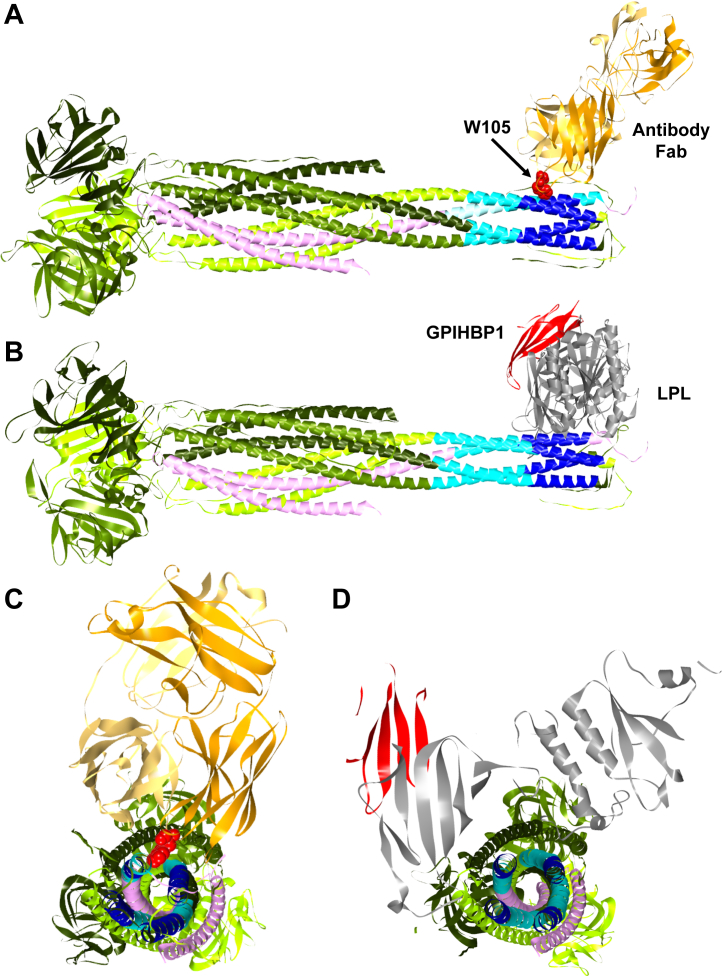
Models of the ANGPTL3/8 complex binding the anti-ANGPTL3/8 antibody Fab and LPL. Blue regions show the areas of decreased HDX. A: Model showing binding of the ANGPTL3/8 complex to the anti-ANGPTL3/8 antibody Fab with HDX relative to unbound ANGPTL3/8 complex. The location of W105 in the Fab heavy chain is highlighted in red. B: Model showing binding of the ANGPTL3/8 complex to GPIHBP1-LPL with HDX relative to unbound ANGPTL3/8 complex. C: Head-on view of the ANGPTL3/8 tetrameric complex bound to the antibody Fab, with the location of W105 in the Fab highlighted in red. D: Head-on view of the ANGPTL3/8 tetrameric complex bound to LPL, suggesting that LPL and the anti-ANGPTL3/8 antibody bind ANGPTL3/8 in a similar manner.

We next performed similar modeling for the ANGPTL3/8 complex when it was bound by the anti-ANGPTL3/8 antibody Fab. Figure 6A shows modeling of the ANGPTL3/8 complex bound by the anti-ANGPTL3/8 antibody Fab, with mapped HDXMS exchange relative to unbound ANGPTL3/8 complex. The three ANGPTL3 leucine zipper-like motifs and the ANGPTL8 leucine zipper-like motif were located within HDXMS-mapped sequences where increased deuterium-hydrogen exchange occurred when ANGPTL3 and ANGPTL8 form the ANGPTL3/8 complex.

For comparison, Fig. 6B shows a model for how the ANGPTL3/8 complex engages LPL present in the GPIHBP1-LPL complex. The same regions that were mapped by HDXMS as the interacting sequences with the antibody Fab were also identified as the interacting regions with GPIHBP1-LPL, whose structure has been previously reported (61). This model suggested that LPL binds to the ANGPTL3/8 complex in a very similar manner to how the anti-ANGPTL3/8 antibody binds the ANGPTL3/8 complex.

Figure 6C shows a head-on view of the ANGPTL3/8 tetrameric complex bound to the antibody Fab, indicating that W105 in the Fab heavy chain may be important for binding of the Fab to the ANGPTL3/8 complex. Figure 6D shows a head-on view of the ANGPTL3/8 tetrameric complex bound to LPL-GPIHBP1, indicating the similarity with which both LPL and the antibody Fab bind ANGPTL3/8. Based on our HDXMS data and modeling of the CCDs, we hypothesize that a leucine zipper enables formation of the novel, unmasked LPL-inhibitory epitope of the ANGPTL3/8 complex where LPL, ApoA5, and the anti-ANGPTL3/8 antibody compete for binding.

The molecular modeling performed for the binding of the Fab to ANGPTL3/8 suggested that W105 in CDR3 of the heavy chain of the anti-ANGPTL3/8 antibody Fab may be potentially critical for Fab binding to the ANGPTL3/8 complex, as it was located precisely between the ANGPTL3 and ANGPTL8 proteins in the ANGPTL3/8 complex. To confirm this possibility, we performed saturated mutagenesis for W105 in the anti-ANGPTL3/8 antibody heavy chain and converted this residue to every other amino acid (except cysteine). In each case, binding to the ANGPTL3/8 complex was markedly reduced. The results for each substituted amino acid in the ANGPTL3/8 binding ELISA are shown compared to W105 in supplemental Table S6. Additional antigen titration binding studies confirmed loss of binding for variant W105A, in which the aromatic side chain at position 105 of the heavy chain was removed (supplemental Fig. S13).

Based on our combined HDXMS and modeling data, we propose that the three ANGPTL3 CCDs and the ANGPTL8 CCD come together via interdigitation of their respective leucine zipper-like motifs to form the ANGPTL3/8 complex. Formation of the leucine zipper between ANGPTL3 and ANGPTL8 unmasks the ANGPTL3 LPL-inhibitory sequence while ANGPTL8 provides structural support and reduces the number of conformations, therefore making the ANGPTL3/8 complex a potent LPL inhibitor. The formation of this leucine zipper also buries hydrophobic leucine residues away from the interface of the coiled-coil domains, thus exposing neighboring hydrophilic amino acids that ensure better solubility of the ANGPTL3/8 complex and confer its ability to bind to and inhibit LPL. As interesting as our model may be, however, it has inherent limitations based on what could observe versus what the model infers. It should therefore be viewed as a preliminary model, with the possibility for further modification when or if the crystal structure of the ANGPTL3/8 complex becomes available.

### The anti-ANGPTL3/8 antibody selectively blocks the ability of ANGPTL3/8 to inhibit LPL

**Fig. 7:**
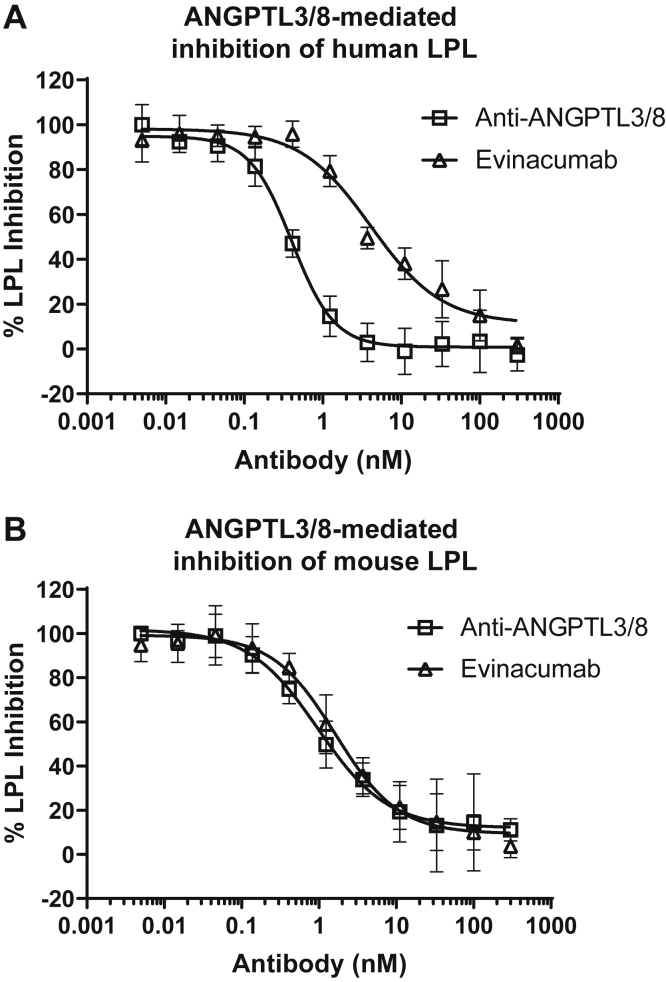
The anti-ANGPTL3/8 antibody blocks the ability of ANGPTL3/8 to inhibit LPL. A: The ability of the anti-ANGPTL3/8 antibody or evinacumab to block human ANGPTL3/8-mediated inhibition of human LPL activity was assessed using human LPL-stable expression cells with fluorescent lipase substrate. Results are shown as the mean ± SD (n = 6 from 3 independent experiments). B: The ability of the anti-ANGPTL3/8 antibody or evinacumab to block mouse ANGPTL3/8-mediated inhibition of mouse LPL activity was assessed using mouse LPL-stable expression cells with fluorescent lipase substrate. Results are shown as the mean ± SD (n = 6 from 3 independent experiments).

In order to characterize the ability of the anti-ANGPTL3/8 antibody to suppress ANGPTL3/8-mediated inhibition of human LPL activity, we assessed the effect of increasing concentrations of the ANGPTL3/8 antibody on the ability of human ANGPTL3/8 complex to inhibit human LPL. The anti-ANGPTL3 antibody evinacumab was also assessed in these assays. Figure 7A shows the results of these experiments, in which the anti-ANGPTL3/8 antibody potently decreased the ability of human ANGPTL3/8 to inhibit human LPL activity (IC_50_ = 0.28 nM). Interestingly, evinacumab also suppressed the ability of human ANGPTL3/8 to inhibit human LPL, albeit less potently than the anti-ANGPTL3/8 antibody (IC_50_ = 4.13 nM). To characterize the ability of the anti-ANGPTL3/8 antibody to suppress mouse ANGPTL3/8-mediated inhibition of mouse LPL activity, we assessed the effect of increasing concentrations of the ANGPTL3/8 antibody on the ability of mouse ANGPTL3/8 complex to inhibit mouse LPL activity (with evinacumab again used as a comparator). Figure 7B shows the results of these experiments, in which the anti-ANGPTL3/8 antibody and evinacumab each decreased the ability of mouse ANGPTL3/8 complex to inhibit mouse LPL enzymatic activity with similar potencies (IC_50_ values of 1.38 and 1.6 nM, respectively).

**Fig. 8:**
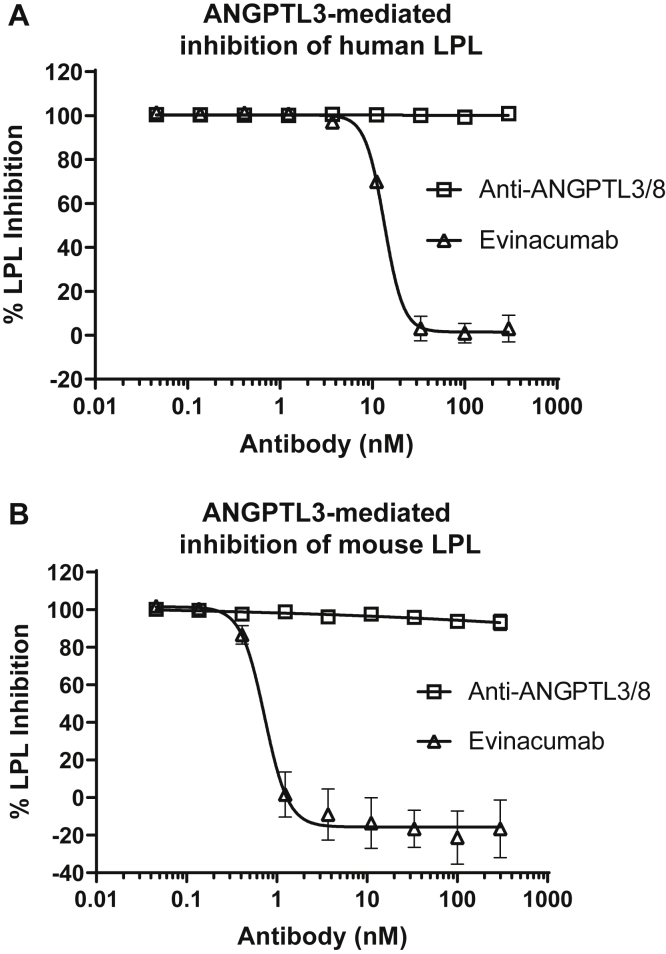
The anti-ANGPTL3/8 antibody does not block ANGPTL3-mediated LPL inhibition. A: The ability of the anti-ANGPTL3/8 antibody or evinacumab to block human ANGPTL3-mediated inhibition of human LPL activity was assessed using human LPL-stable expression cells with fluorescent lipase substrate. Results are shown as the mean ± SD (n = 6 from 2 independent experiments). B: The ability of the anti-ANGPTL3/8 antibody or evinacumab to block mouse ANGPTL3-mediated inhibition of mouse LPL activity was assessed using mouse LPL-stable expression cells with fluorescent lipase substrate. Results are shown as the mean ± SD (n = 6 from 2 independent experiments).

Because ANGPTL3 alone can inhibit LPL (although much less potently than the ANGPTL3/8 complex), we wanted to ensure that our anti-ANGPTL3/8 antibody was specific for ANGPTL3/8 and did not block the ability of ANGPTL3 to inhibit LPL. To confirm that this was the case, we first assessed the ability of increasing concentrations of the ANGPTL3/8 antibody to block human ANGPTL3-mediated inhibition of human LPL activity. Figure 8A shows the results of these experiments, in which the anti-ANGPTL3/8 antibody did not block the ability of human ANGPTL3 to inhibit human LPL activity. In contrast, the anti-ANGPTL3 antibody evinacumab dose-dependently blocked the ability of human ANGPTL3 to inhibit human LPL activity (IC_50_ = 13.5 nM). Similar experiments were also performed with mouse ANGPTL3. Figure 8B shows the results, in which the anti-ANGPTL3/8 antibody showed no suppression of the mouse ANGPTL3-mediated LPL inhibition, while evinacumab demonstrated dose-dependent suppression of the ability of mouse ANGPTL3 to inhibit mouse LPL enzymatic activity (IC_50_ = 0.73 nM).

**Fig. 9:**
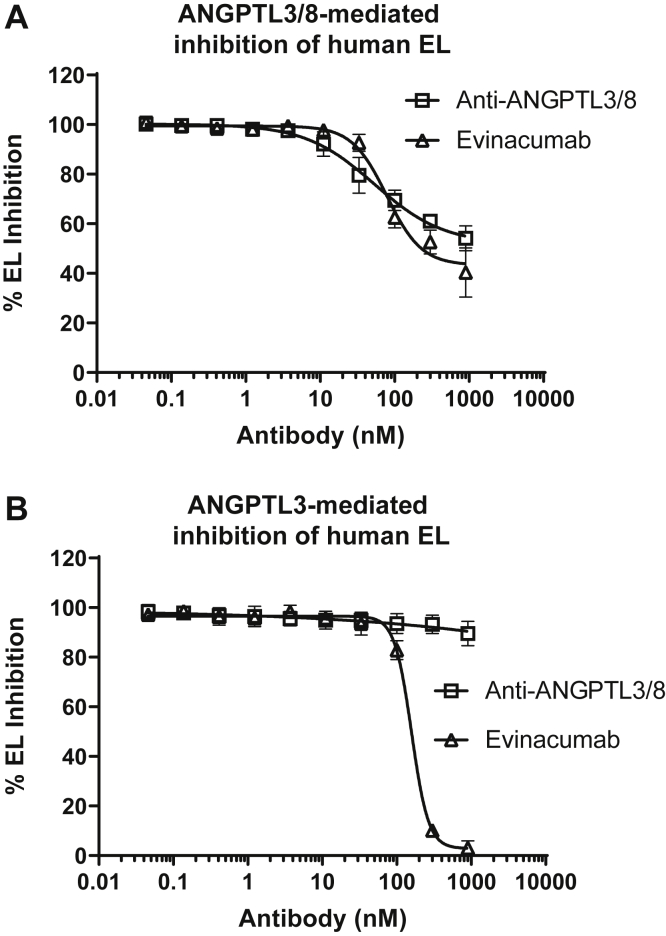
The anti-ANGPTL3/8 antibody blocks the ability of ANGPTL3/8 to inhibit EL much less potently than it blocks the ability of ANGPTL3/8 to inhibit LPL. A: The ability of the anti-ANGPTL3/8 antibody or evinacumab to block ANGPTL3/8-mediated inhibition of human EL activity was assessed using EL-stable expression cells with fluorescent PLA1 substrate. Results are shown as the mean ± SD (n = 4 from 3 independent experiments). B: The ability of the anti-ANGPTL3/8 antibody or evinacumab to block ANGPTL3-mediated inhibition of human EL activity was assessed using EL-stable expression cells with fluorescent PLA1 substrate. Results are shown as the mean ± SD (n = 4 from 3 independent experiments). EL, endothelial lipase.

In light of our recent observations that ANGPTL3/8 can also inhibit EL activity and do so more potently than ANGPTL3 alone (62), we performed additional experiments to determine the effect of the anti-ANGPTL3/8 antibody and evinacumab on ANGPTL3/8-mediated inhibition of EL. Interestingly, we observed that the anti-ANGPTL3/8 antibody and evinacumab both comparably blocked ANGPTL3/8-mediated EL inhibition and did so much less completely and potently (IC_50_ values of 53 nM and 78 nM, respectively) than they blocked ANGPTL3/8-mediated LPL inhibition (Fig. 9A). As expected, only evinacumab was observed to block ANGPTL3-mediated EL inhibition (IC_50_ value of 158 nM), while the anti-ANGPTL3/8 antibody had no effect on ANGPTL3-mediated EL inhibition (Fig. 9B). Taken together, the results from these experiments demonstrated that the anti-ANGPTL3/8 antibody had no effect on ANGPTL3-mediated LPL or EL inhibition and preferentially suppressed human ANGPTL3/8-mediated LPL inhibition versus ANGPTL3/8-mediated EL inhibition. The anti-ANGPTL3/8 antibody also suppressed human ANGPTL3/8-mediated inhibition of LPL more potently than did evinacumab. Our data showing that the anti-ANGPTL3/8 antibody was able to cross-react with murine ANGPTL3/8 also indicated that it should be suitable for in vivo experiments in mice.

### The anti-ANGPTL3/8 antibody potently lowers TG in mice

**Fig. 10:**
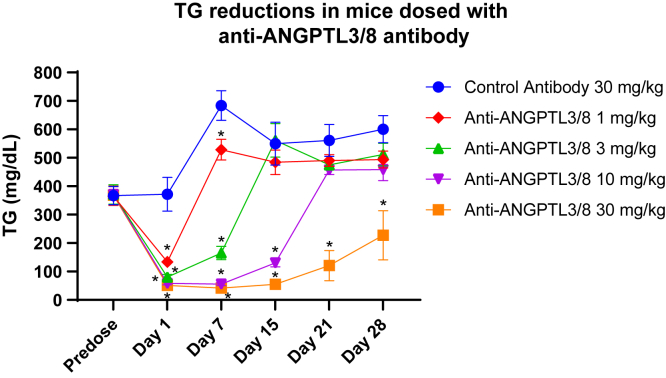
The anti-ANGPTL3/8 antibody potently lowers TG in vivo. Hypertriglyceridemic CETP/ApoA1 transgenic mice were administered a single dose of the anti-ANGPTL3/8 antibody (at 1, 3, 10, or 30 mg/kg) or an irrelevant isotype-matched control antibody (at 30 mg/kg). Blood was collected predose and 1 day, 7 days, 15 days, 21 days, and 28 days following administration of the single dose of the antibody. Serum TG levels were measured using a Roche Cobas assay. Results are shown as the mean ± SEM for 7 mice for each antibody group for each time point. Asterisks denote *p*-values of < 0.05. ApoA1, apolipoprotein A1.

The results described above indicated that the anti-ANGPTL3/8 antibody potently and selectively suppressed the ability of human ANGPTL3/8 to inhibit human LPL activity as well as the ability of mouse ANGPTL3/8 to inhibit mouse LPL activity. To determine if the anti-ANGPTL3/8 antibody would also potently decrease serum TG levels in vivo, we administered the anti-ANGPTL3/8 antibody to transgenic CETP/ApoA1 hypertriglyceridemic mice (45) at doses ranging from 1 mg/kg to 30 mg/kg. In this experiment, groups of 7 nonfasted mice with comparable baseline TG levels were each administered a single dose of the anti-ANGPTL3/8 antibody, with administration of a single 30 mg/kg dose of an irrelevant isotype-matched antibody serving as the control. Serum TG levels were then measured 1 day, 7 days, 14 days, 21 days, and 28 days after the single dose of the anti-ANGPTL3/8 antibody or isotype-matched control antibody was administered. Figure 10 shows the results of this experiment, in which the anti-ANGPTL3/8 antibody rapidly and dose-dependently reduced circulating TG in the nonfasted mice, with the reduction in circulating TG being sustained even after 28 days in the highest dose group. This experiment thus clearly demonstrated that a single dose of the anti-ANGPTL3/8 antibody could lower serum TG dose-dependently for at least 28 days.

## Discussion

Data from our current study reveal that a novel epitope forms when ANGPTL8 combines with ANGPTL3 to create the ANGPTL3/8 complex. This novel conformational epitope includes N-terminal regions of both proteins that are involved in the inhibition of LPL activity. Both our HDXMS data and modeling studies support the possibility that the N-terminal CCDs of ANGPTL3 and ANGPTL8 come together via interdigitation of their respective leucine zipper-like motifs when the ANGPTL3/8 complex forms. The formation of this leucine zipper then buries the hydrophobic leucine residues away from the interface of the two coiled-coil domains, with the result being that neighboring hydrophilic amino acids are outwardly exposed to ensure solubility of the ANGPTL3/8 complex and its ability to bind to and potently inhibit LPL.

Our findings build upon the previous observations of several groups. In 2009, Yau et al. found that a 12-amino acid consensus motif in the CCD of ANGPTL4 was critical for its binding to and inhibition of LPL (48). That same year, Lee et al. demonstrated that the specific epitope 1 region in the CCD of ANGPTL3 and ANGPTL4 was crucial for the binding of both proteins to LPL as well as their ability to inhibit LPL activity (58). In 2017, Haller et al. showed that ANGPTL8 itself also possesses LPL inhibitory activity, but that it was only able to inhibit LPL and increase circulating TG levels in vivo in the presence of ANGPTL3. (19).

Interestingly, as we show in our current study, both LPL and the TG-lowering apolipoprotein ApoA5 recognize the same epitope present in the ANGPTL3/8 complex, suggesting that ApoA5 acts like an endogenous ANGPTL3/8 inhibitor in order to protect LPL from inhibition by the ANGPTL3/8 complex and preserve its activity. To achieve this ANGPTL3/8 inhibition, however, ApoA5 must circulate at molar levels roughly 50-100-fold greater than those of ANGPTL3/8 (25). The anti-ANGPTL3/8 antibody that we developed also targeted this same region of the ANGPTL3/8 complex that was bound by ApoA5 and LPL and almost completely blocked the binding of ANGPTL3/8 to LPL. In so doing, the anti-ANGPTL3/8 antibody was also found to suppress potently and selectively the ability of ANGPTL3/8 to inhibit the enzymatic activity of both mouse and human LPL. Furthermore, when this antibody was administered to hypertriglyceridemic mice, it markedly decreased their circulating TG concentrations, thus confirming that specific inhibition of the ANGPTL3/8 complex in vivo represents a novel approach for lowering serum TG.

Together, these findings open up a new possibility for the treatment of hyperlipidemia. Already, inhibition of ANGPTL3 is under intense investigation for this indication, with therapeutic antibodies such as evinacumab (which as recently approved) under development in the clinic (69, 70, 71, 72). Based on the results of our current study, as well as those of our previous studies in which we showed that ANGPTL3/8 is secreted by hepatocytes in an insulin-dependent manner (22, 44), we would predict that both of these modalities actually work by targeting the ANGPTL3/8 complex secreted by the liver. In the case of ANGPTL3 siRNA molecules (which specifically target the liver), decreased hepatic expression of ANGPTL3 would be expected to result in decreased formation of the ANGPTL3/8 complex intracellularly within hepatocytes, thus decreasing release of the ANGPTL3/8 complex into the bloodstream. In the case of evinacumab, our data suggest that although it was developed as an anti-ANGPTL3 antibody (which it appears to be based on our results in this study), it also recognizes ANGPTL3 in such a way that it is able to target the ANGPTL3/8 complex. Interestingly, a recent report suggested that this might be the case (73).

While targeting ANGPTL3 has been shown to lower serum TG concentrations, it also appears to carry the unwanted property of decreasing HDL levels. This is likely due to the fact that in addition to inhibiting LPL, ANGPTL3 is also an inhibitor of EL, the enzyme that hydrolyzes PL in HDL, particularly PL-rich HDL (74, 75, 76, 77, 78). There is currently some uncertainty regarding the ability of ANGPTL3/8 to inhibit EL compared to the ability of ANGPTL3 to inhibit EL. We recently demonstrated that ANGPTL3/8 inhibited EL more potently than does ANGPTL3, although the effect was not nearly as dramatic as what we had previously observed for LPL (62). In contrast, Sylvers-Davie et al. recently found that ANGPTL8 did not significantly alter the binding to or the inhibition of EL by ANGPTL3 (79). The reasons for these differing findings are unclear and could possibly be related to differences in how the ANGPTL3 and ANGPTL8 proteins were expressed, suggesting that further investigation will be required to understand better the effect of ANGPTL8 on the EL-inhibitory activity of ANGPTL3.

Along these lines, it has previously been reported that knockout of the EL gene results in increased HDL levels while elevated EL activity results in decreased circulating HDL due to the loss of PL from HDL, which subsequently results in relatively PL-poor HDL particles that are then cleared more readily from the circulation (74, 75, 76, 77, 78). In addition to this effect on HDL, by targeting ANGPTL3 which circulates at roughly 200 ng/ml (vs. approximately 20 ng/ml for the ANGPTL3/8 complex), evinacumab would be expected to be affected by target-mediated drug disposition. This may explain why it must be administered intravenously at high doses (15 mg/kg) in the clinic (69, 70, 71, 72). Interestingly, we observed that both our anti-ANGPTL3/8 antibody and evinacumab blocked ANGPTL3/8-mediated EL inhibition much less potently and completely than they blocked ANGPTL3/8-mediated LPL inhibition. In contrast, only evinacumab was observed to block ANGPTL3-mediated EL inhibition and it did so only at relatively high concentrations.

The approach of targeting ANGPTL3/8 with an anti-ANGPTL3/8-specific antibody might have some particular advantages. Because such an antibody would target only the ANGPTL3/8 complex, and not ANGPTL3, lower doses may be possible, thus opening the door for subcutaneous rather than intravenous administration. With regard to the effect on HDL, our previous data, which correlated the circulating levels of ANGPTL3/8 with markers of metabolic syndrome in human control subjects, demonstrated that ANGPTL3/8 was significantly inversely correlated with HDL, while being significantly directly correlated with all other markers of metabolic syndrome (22). The significant inverse correlation of ANGPTL3/8 with HDL would thus suggest that targeting the ANGPTL3/8 complex should increase rather than decrease circulating HDL levels. Of course, human clinical trial data will have to be generated in order to show whether or not this is actually the case. One patient population that might be particularly interesting to study would be hyperlipidemic patients with type 2 diabetes, who can often present with increased TG and decreased HDL (80).

Hyperlipidemia associated with elevated TG and an increased TG/HDL ratio is being increasingly recognized as a significant atherosclerotic CVD risk, beyond the risk posed by elevated LDL-C and ApoB concentrations (81, 82, 83). Over the past several years, mouse knockout studies of ANGPTL3 and ANGPTL8 as well as studies examining anti-ANGPTL3 antibodies and human ANGPTL3 and ANGPTL8 knockout mutations have increasingly suggested that targeting ANGPTL3 and/or ANGPTL8 might represent the next frontier of lipid lowering and CVD risk reduction. In the case of ANGPTL3, knockout mice have been shown to have markedly decreased circulating TG, and targeting ANGPTL3 with an anti-ANGPTL3 antibody has been shown to decrease TG concentrations (84, 85, 86, 87, 88, 89, 90). In humans, ANGPTL3 knockout mutations have been associated with decreased TG and LDL-C (as well as decreased HDL) along with a significantly reduced risk of CVD while targeting ANGPTL3 with an anti-ANGPTL3 antibody has been demonstrated to reduce TG levels (84, 85, 86, 87, 88, 89, 90).

In the case of ANGPTL8, whole body knockout mice manifested a favorable phenotype that included dramatically reduced TG levels, especially in the feeding state, and decreased fat mass with preserved lean body mass (17). This phenotype is mimicked in liver-specific ANGPTL8 knockout mice, whereas adipose-specific ANGPTL8 knockout mice were actually found to have increased postprandial TG, likely due to decreased ANGPTL4/8 in the fat (23). Based on our previous study, localized ANGPTL4/8 in the adipose tissue is essential in preserving LPL activity so that FA from postprandial TG can be directed for storage in the fat rather than being deposited ectopically (22). Together, these observations suggest that reduction of the ANGPTL3/8 complex made and secreted by the liver may be the mechanism by which ANGPTL8 whole body knockout mice have a beneficial phenotype.

Understanding the effects of ANGPTL8 knockout mutations in humans has proved much more difficult, likely due to the rarity of these mutations. Until recently, the key finding in this area was a 2014 report by Peloso et al. that examined the effects of the ANGPTL8 121X heterozygous knockout mutation (91). These authors found that this mutation was associated with a 15% decrease in circulating TG, an absolute 10 mg/dl increase in HDL (both of which were highly statistically significant) and a 5.8 mg/dl decrease in LDL-C (91). Unfortunately, because the 121X mutation is very rare (only about a 0.1% frequency in Europeans), the study was not sufficiently powered to assess CVD protection, so no definitive conclusions could be drawn about whether or not this mutation conferred a decreased risk of atherosclerotic CVD (91).

For the next several years, this remained an open question until the publication of a very recent report by Helkkula et al. that described the effects of a novel ANGPTL8 131X knockout mutation in humans (92). These authors found that subjects heterozygous for this mutation had a 24 mg/dl decrease in TG, a 16.8 mg/dl decrease in LDL-C, a 14.2 mg/dl decrease in TC, and a 9.1 mg/dl increase in HDL, all of which were highly statistically significant (92). Importantly, the carriers of this mutation also had a highly statistically significant 47% decreased risk of CVD compared to noncarriers. This reduction in CVD risk was made even more dramatic by the fact that carriers of the mutation were only about half as likely to be using statins compared to noncarriers (92). In addition, data from the same study also suggested that this mutation may confer protection against type 2 diabetes, although an even more highly powered study would be needed to address this question definitively (92).

Not surprisingly, Helkkula et al. suggested that ANGPTL8 could be an intriguing target for treating dyslipidemia and reducing CVD risk (92). When these human genetic data are viewed in the context of our current study, it would seem that targeting ANGPTL8 via inhibiting the ANGPTL3/8 complex may be an attractive approach. Evidence supporting this approach includes our observation that the TG-lowering apolipoprotein ApoA5 acts as an endogenous inhibitor of the ANGPTL3/8 complex by binding to the same site on the complex to which LPL binds, thus protecting LPL from inhibition by ANGPTL3/8. In addition, we also now show that it is possible to target this unique ANGPTL3/8 epitope with a therapeutic monoclonal antibody and that such an antibody is capable of potently blocking ANGPTL3/8-mediated inhibition of LPL enzymatic activity in vitro and markedly lowering TG in vivo. Another piece of data suggesting that specifically targeting the ANGPTL3/8 complex (rather than taking a pan anti-ANGPTL8 approach) may be advantageous is the recent observation by Oldoni et al. that mice with an adipose-specific ANGPTL8 knockout (which would affect mainly ANGPTL4/8 in the fat) actually presented with increased postprandial TG levels, while liver-specific ANGPTL8 knockout mice had decreased circulating TG concentrations (23).

A final question that arises is what effect an anti-ANGPTL3/8 antibody might have on LDL-C levels in humans. In our previous study, we demonstrated that ANGPTL3/8 (but not ANGPTL4/8) was significantly positively correlated with LDL-C in human subjects (22). This would suggest that inhibition of ANGPTL3/8 should result in decreased circulating LDL-C levels. Our observation that the anti-ANGPTL3 antibody evinacumab inhibits the ANGPTL3/8 complex (in addition to inhibiting ANGPTL3) combined with the fact that evinacumab significantly lowers LDL-C in human patients (69, 70, 71, 72) would further suggest that targeting the ANGPTL3/8 complex might reduce circulating LDL-C levels. Along these lines, however, consideration should also be given to a previous report that inhibition of EL may be an important mechanism for the LDL-C lowering observed with ANGPTL3 inactivation, even in the absence of the LDL receptor, thus suggesting the possibility that ANGPTL3 may modulate LDL-C concentrations through an EL-dependent clearance mechanism (78). Clearly, further investigation will be required to assess the effect of ANGPTL3/8 inhibition versus ANGPTL3 inhibition on LDL-C levels.

In summary, our data cast important new light on the regulation of serum TG levels and answer some of the key emerging questions that have been posed about how ApoA5 and LPL interact with ANGPTL3/8 (93). While many uncertainties remain, our observation that the CCDs of ANGPTL3 and ANGPTL8 come together via interactions of their respective leucine zipper-like motifs to form an LPL-inhibitory epitope is an important step forward in our understanding of the ANGPTL3/8 complex. Similarly, our observation that ApoA5 and LPL bind to the same unique leucine zipper epitope on the ANGPTL3/8 complex and that an anti-ANGPTL3/8 antibody targeting this epitope markedly decreases ANGPTL3/8-mediated inhibition of LPL in vitro and dramatically lowers TG in vivo all point to ANGPTL3/8 as a critical intersection where multiple proteins of interest converge (94, 95). Taken together, our findings provide insights into the mechanisms regulating lipid metabolism while at the same time indicating that additional investigation will be required to understand more fully how these findings might be able to be translated into novel therapeutic approaches for treating hyperlipidemia.

## Data availability

All study data are contained within the manuscript and the accompanying Supplementary Data File.

## Supplemental data

This article contains supplemental data

## Conflict of interest

The authors declare that they have no conflicts of interest with the contents of this article.
