# Supplementary material for: An anti-ANGPTL3/8 antibody decreases circulating triglycerides by binding to a LPL-inhibitory leucine zipper-like motif
Source: J Lipid Res. 2022 Mar 17;63(5):100198. doi: 10.1016/j.jlr.2022.100198 (PMC9036128; doi:10.1016/j.jlr.2022.100198)
Supplement: Supplemental Figures S1–S13 and Tables S1–S5 [file mmc1.pdf]

SUPPLEMENTAL INFORMATION

An anti-ANGPTL3/8 antibody decreases circulating triglycerides by binding to a LPL-inhibitory leucine zipper-like motif

Deepa Balasubramaniam*, Oliver Schroeder*, Anna M. Russell, Jonathan R. Fitchett, Aaron K. Austin, Thomas P. Beyer, Yan Q. Chen, Jonathan W. Day, Mariam Ehsani, Aik Roy Heng, Eugene Y. Zhen, Julian Davies, Wolfgang Glaesner, Bryan E. Jones, Robert W. Siegel, Yue-Wei Qian, and Robert J. Konrad**

Lilly Research Laboratories, Eli Lilly and Company

*These authors contributed equally to this work

**Author for correspondence

Figure Legends for Figures S1-S5

Figure S1: HDXMS data show the ANGPTL3/8 complex contains a neo-epitope. (A) Sequence coverage showing ANGPTL3 peptides that were followed in the experiment. (B) Sequence coverage showing ANGPTL8 peptides that were followed in the experiment. (C) Difference plot comparing changes in deuterium uptake of ANGPTL3 peptides in ANGPTL3/8 versus ANGPTL3 alone. (D) Difference plot comparing changes in deuterium uptake of ANGPTL8 peptides in ANGPTL3/8 versus ANGPTL8 alone. For C and D, peptides that exhibited differences in deuterium exchange that exceeded their individual uncertainty values (Tables S1 and S2) were considered significantly different. (E) Heat map of the relative deuterium uptake comparing ANGPTL3 in ANGPTL3/8 versus ANGPTL3 alone. Orange areas show increased deuterium uptake while blue areas show decreased uptake. (F) Heat map of relative deuterium uptake comparing ANGPTL8 in ANGPTL3/8 versus ANGPTL8 alone. Orange areas show increased deuterium uptake while blue areas show decreased uptake. (G) Individual uptake plots for ANGPTL3 peptides in ANGPTL3/8 compared to ANGPTL3 alone. (F) Individual uptake plots for ANGPTL8 peptides in ANGPTL3/8 compared to ANGPTL8 alone.

Figure S2: HDXMS data show that the ANGPTL3/8 neo-epitope is recognized by LPL. (A) Sequence coverage for ANGPTL3 peptides that were followed in the experiment. (B) Sequence coverage for ANGPTL8 peptides that were followed in the experiment. (C) Difference plot comparing changes in deuterium uptake of ANGPTL3 peptides in ANGPTL3/8 complex bound to LPL relative to ANGPTL3 in unbound ANGPTL3/8 complex. (D) Difference plot comparing changes in deuterium uptake of ANGPTL8 peptides in ANGPTL3/8 complex bound to LPL relative to ANGPTL8 in unbound ANGPTL3/8 complex. For C and D, peptides that exhibited differences in deuterium exchange that exceeded their individual uncertainty values (Table S3) were considered significantly different (E) Heat map of the relative deuterium uptake for ANGPTL3 in ANGPTL3/8 complex bound to LPL relative to ANGPTL3 in unbound ANGPTL3/8 complex. Orange areas show regions of increased deuterium uptake while blue areas show regions of decreased uptake. (F) Heat map of the relative deuterium uptake ANGPTL8 in ANGPTL3/8 complex bound to LPL relative to ANGPTL8 in unbound ANGPTL3/8 complex. Orange areas show increased deuterium uptake while blue areas show decreased uptake. (G) Individual uptake plots for ANGPTL3 peptides in ANGPTL3/8 bound to LPL compared to unbound ANGPTL3/8. (H) Individual uptake plots for ANGPTL8 peptides in ANGPTL3/8 bound to LPL compared to unbound ANGPTL3/8.

Figure S3: HDXMS data show that the ANGPTL3/8 neo-epitope is recognized by ApoA5. (A) Sequence coverage for ANGPTL3 peptides that were followed in the experiment. (B) Sequence coverage for ANGPTL8 peptides that were followed in the experiment. (C) Difference plot comparing changes in deuterium uptake of ANGPTL3 peptides in ANGPTL3/8 complex bound to ApoA5 relative to ANGPTL3 in unbound ANGPTL3/8 complex. (D) Difference plot comparing changes in deuterium uptake of ANGPTL8 peptides in ANGPTL3/8 complex bound to ApoA5 relative to ANGPTL8 in unbound ANGPTL3/8 complex. For C and D, peptides that exhibited differences in deuterium exchange that exceeded their individual uncertainty values (Table S4) were considered significantly different. (E) Heat map of the relative deuterium uptake for ANGPTL3 peptides in ANGPTL3/8 complex bound to ApoA5 relative to ANGPTL3 in

unbound ANGPTL3/8 complex. Orange areas show regions of increased deuterium uptake while blue areas show regions of decreased uptake. (F) Heat map of the relative deuterium uptake ANGPTL8 peptides in ANGPTL3/8 complex bound to ApoA5 relative to ANGPTL8 in unbound ANGPTL3/8 complex. Orange areas show increased deuterium uptake while blue areas show decreased uptake. (G) Individual uptake plots for ANGPTL3 peptides in ANGPTL3/8 bound to ApoA5 compared to unbound ANGPTL3/8. (H) Individual uptake plots for ANGPTL8 peptides in ANGPTL3/8 bound to ApoA5 compared to unbound ANGPTL3/8.

Figure S4: HDXMS data show that the ANGPTL3/8 neo-epitope is recognized by the anti-ANGPTL3/8 antibody. (A) Sequence coverage for ANGPTL3 peptides that were followed in the experiment. (B) Sequence coverage for ANGPTL8 peptides that were followed in the experiment. (C) Difference plot comparing changes in deuterium uptake of ANGPTL3 peptides in ANGPTL3/8 complex bound to antibody relative to ANGPTL3 in unbound ANGPTL3/8 complex. (D) Difference plot comparing changes in deuterium uptake of ANGPTL8 peptides in ANGPTL3/8 complex bound to antibody relative to ANGPTL8 in unbound ANGPTL3/8 complex. For C and D, peptides that exhibited differences in deuterium exchange that exceeded their individual uncertainty values (Table S5) were considered significantly different. (E) Heat map of the relative deuterium uptake for ANGPTL3 peptides in ANGPTL3/8 complex bound to the antibody relative to ANGPTL3 in unbound ANGPTL3/8 complex. Orange areas show regions of increased deuterium uptake while blue areas show regions of decreased uptake. (F) Heat map of the relative deuterium uptake for ANGPTL8 peptides in ANGPTL3/8 complex bound to the antibody relative to ANGPTL8 in unbound ANGPTL3/8 complex. Orange areas show increased deuterium uptake while blue areas show decreased uptake. (G) Individual uptake plots for ANGPTL3 peptides in ANGPTL3/8 bound to the antibody compared to unbound ANGPTL3/8. (H) Individual uptake plots for ANGPTL8 peptides in ANGPTL3/8 complex bound to the antibody compared to unbound ANGPTL3/8.

Figure S5: Representative volcano plots for ANGPTL3 and ANGPTL8 peptides. Observed changes in deuterium exchange Δ(HX) at 10-min are plotted against Welch's t-test q-values. The horizontal q-value significance is set at a 10% false discovery rate (FDR). (A) ANGPTL3 in the ANGPTL3/8 complex versus ANGPTL3 alone. ANGPTL3 peptides showing increased uptake in the ANGPTL3/8 complex compared to ANGPTL3 alone correspond to the same peptides showing decreased uptake when the ANGPTL3/8 complex is bound to LPL, ApoA5, or the anti-ANGPTL3/8 antibody. (B) ANGPTL8 in the ANGPTL3/8 complex versus ANGPTL8 alone. ANGPTL8 peptides showing increased uptake in the ANGPTL3/8 complex compared to ANGPTL8 alone correspond to the same peptides showing decreased uptake when the ANGPTL3/8 complex is bound to LPL, ApoA5, or the anti-ANGPTL3/8 antibody.

Figure S1. HDXMS comparing the ANGPTL3/8 complex to ANGPTL3 and ANGPTL8

Figure S1A. Sequence coverage for ANGPTL3 (deuterated peptides followed in the experiment)

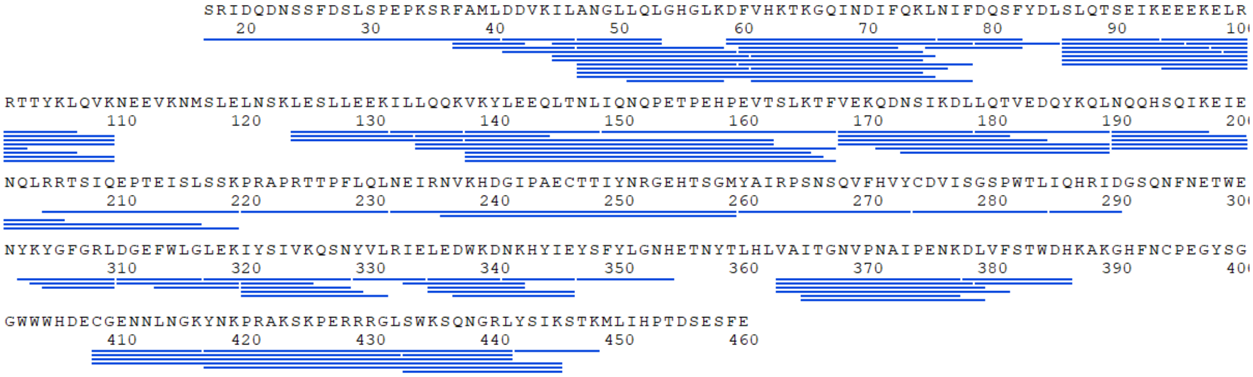

Total: 106 Peptides, 85.1% Coverage, 4.20 Redundancy

Figure S1B. Sequence coverage for ANGPTL8 (deuterated peptides followed in the experiment)

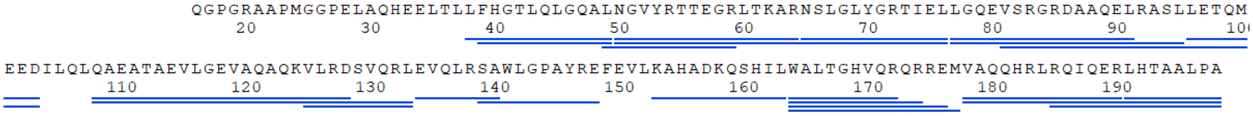

Total: 24 Peptides, 83.6% Coverage, 2.22 Redundancy

Figure S1C. Difference plot comparing the changes in deuterium uptake of ANGPTL3 in the ANGPTL3/8 complex versus ANGPTL3 alone

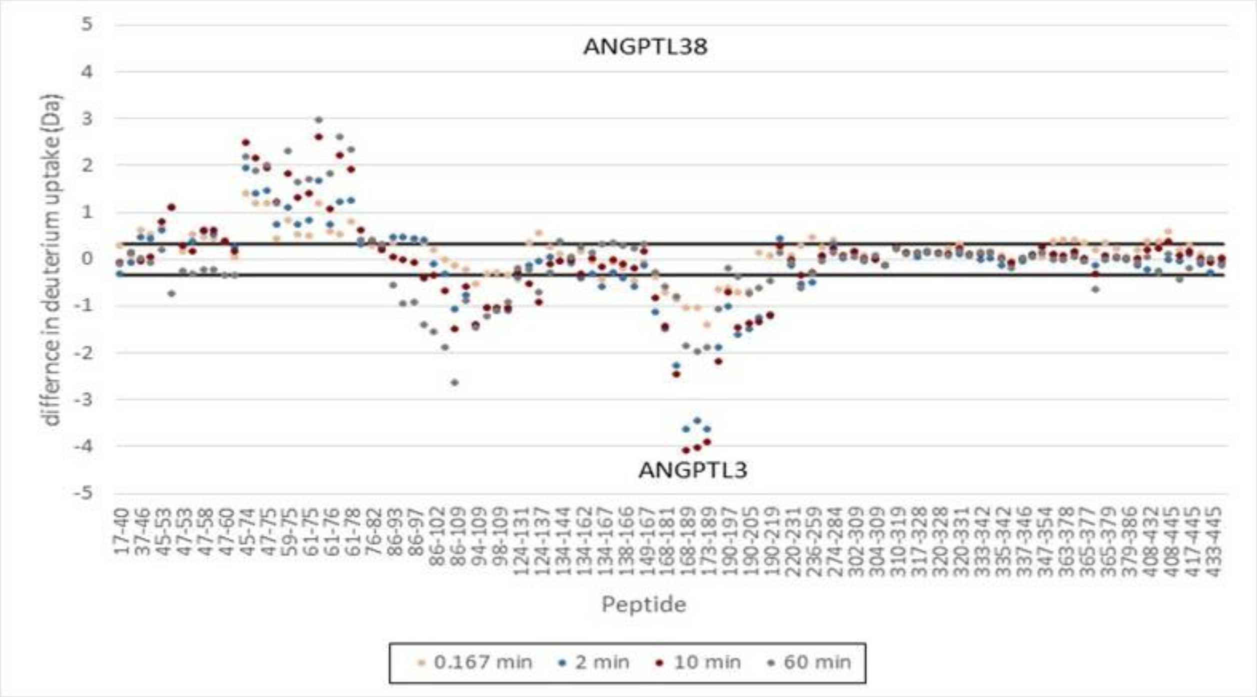

Figure S1D. Difference plot comparing the changes in deuterium uptake of ANGPTL8 in the ANGPTL3/8 complex versus ANGPTL8 alone

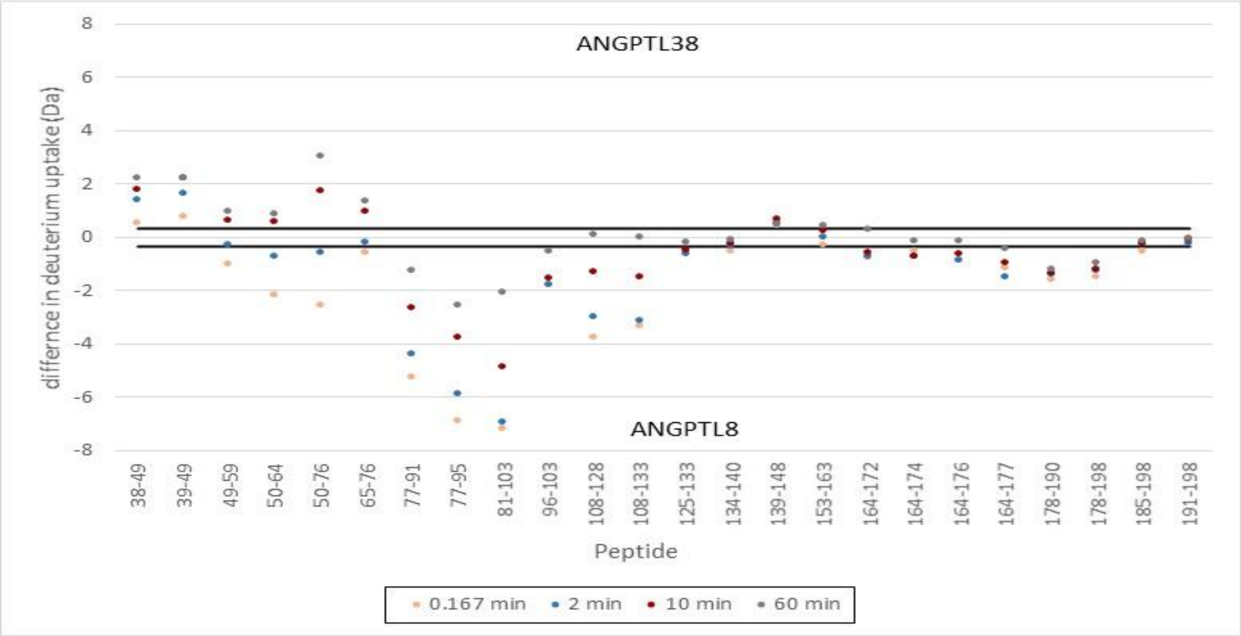

Figure S1E. Heat map of the relative deuterium uptake comparing ANGPTL3 in the ANGPTL3/8 complex versus ANGPTL3 alone

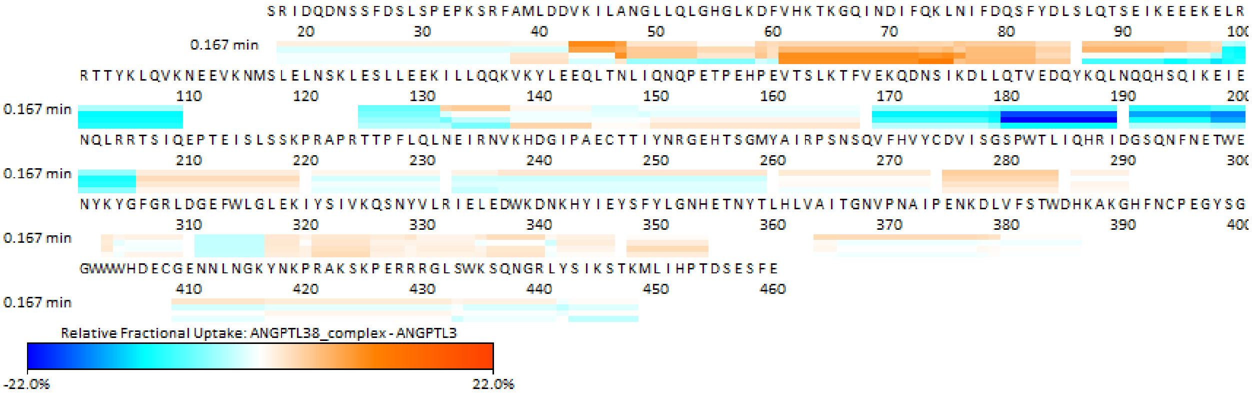

Figure S1F. Heat map of the relative deuterium uptake comparing ANGPTL8 in the ANGPTL3/8 complex versus ANGPTL8 alone

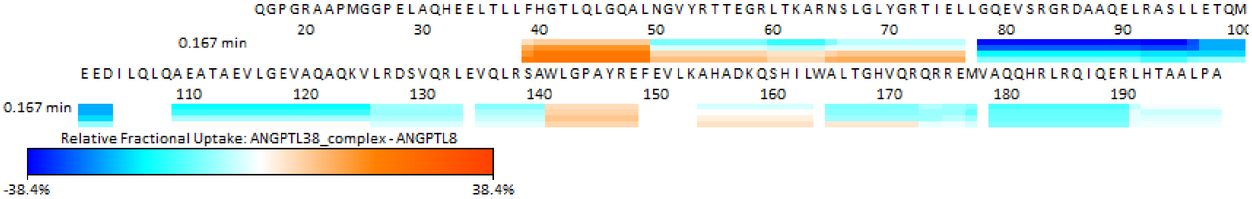

Figure S1G. Uptake plots for ANGPTL3 in ANGPTL3/8 complex compared to ANGPTL3 alone: Standard deviation in uptake difference is 0.11 Da (Experiment performed in triplicate with error bars shown).

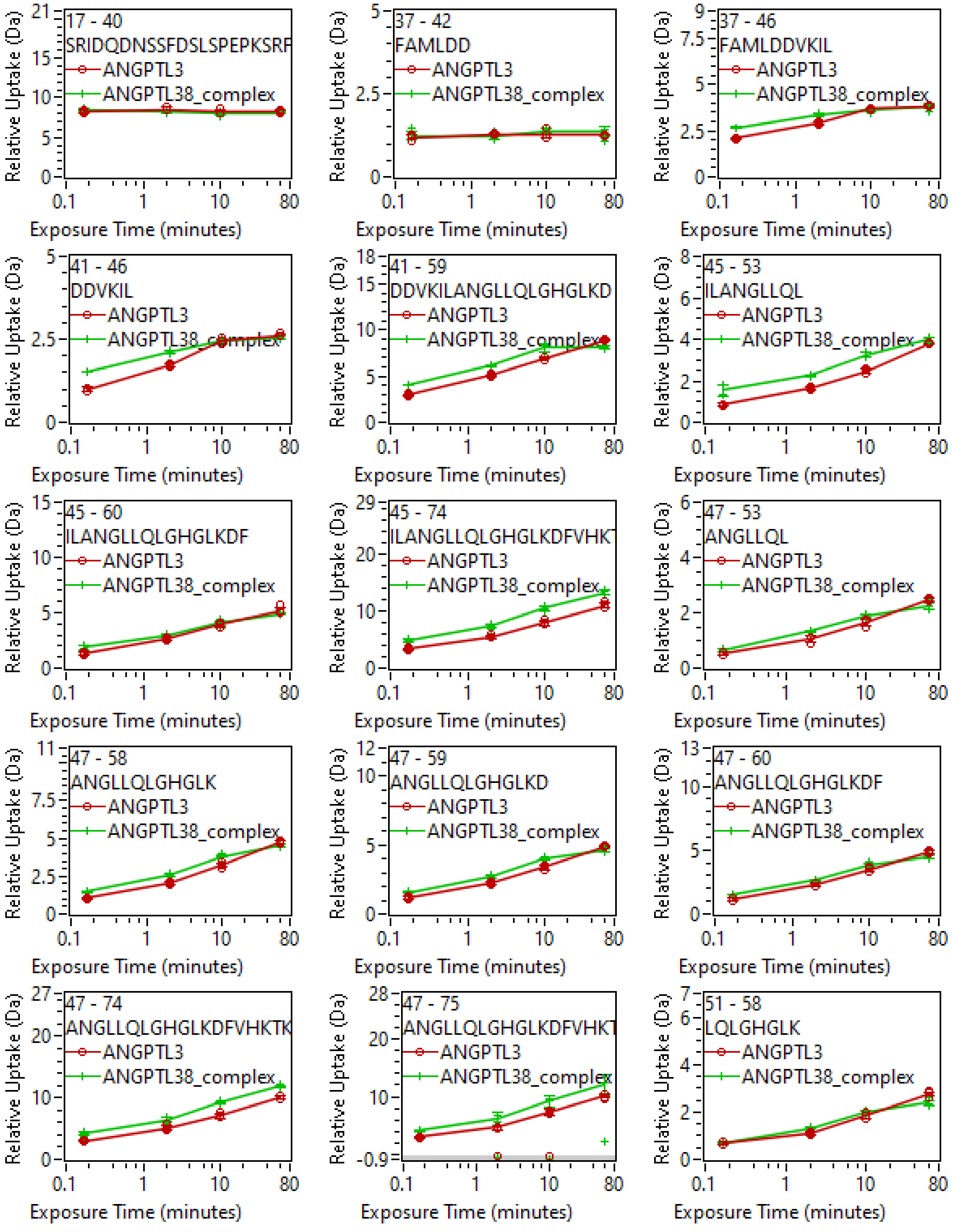

Figure S1G Continued

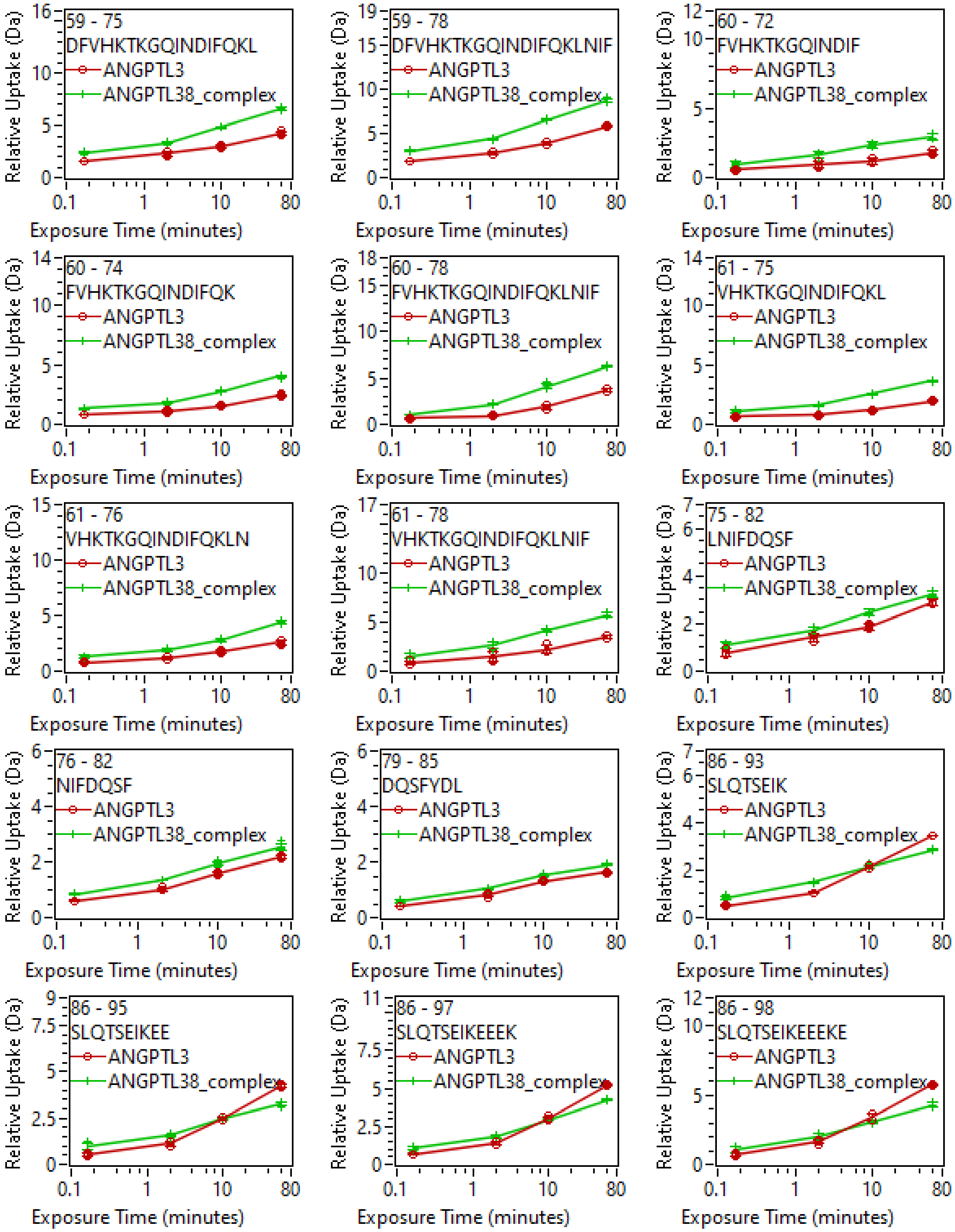

Figure S1G Continued

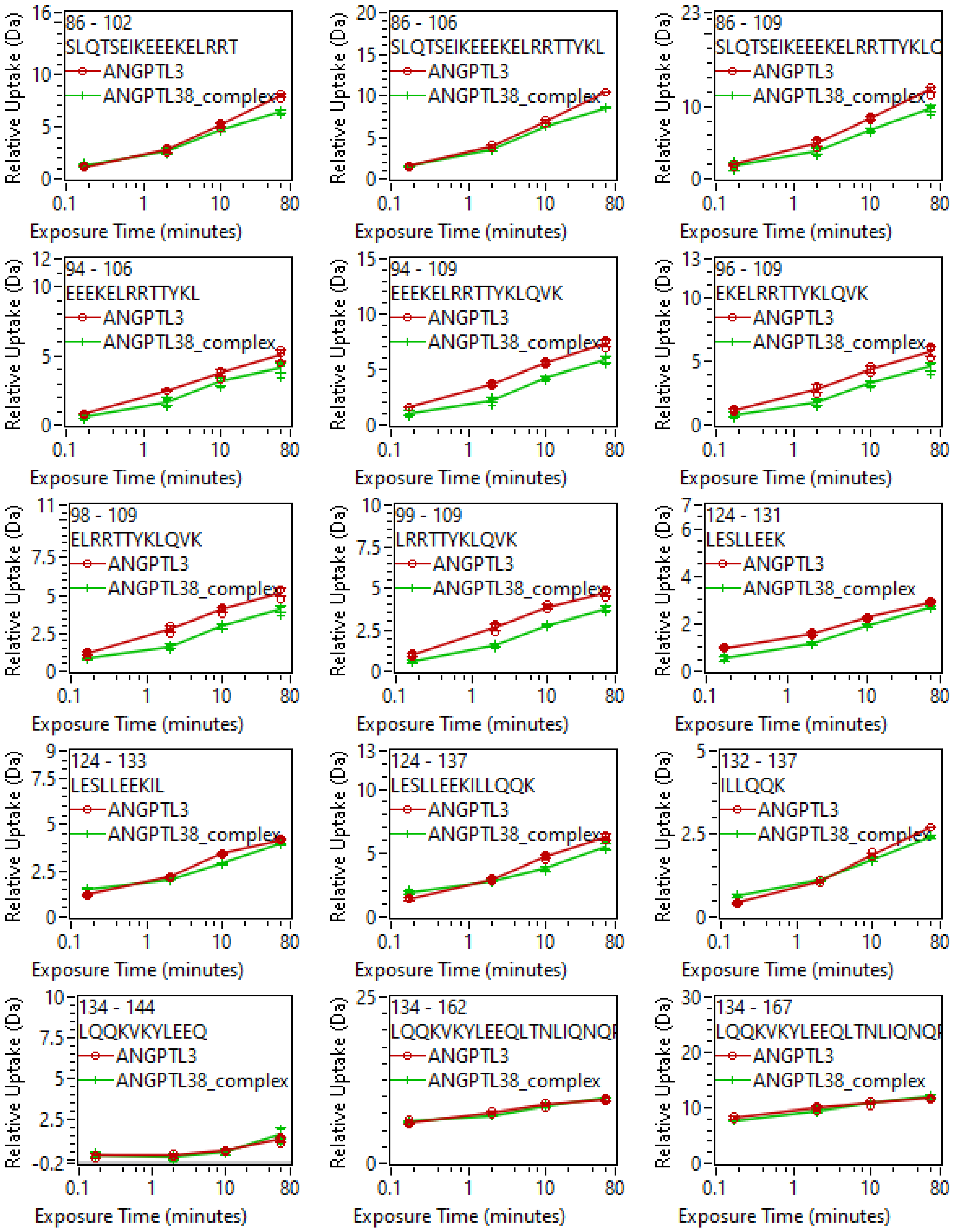

Figure S1G Continued

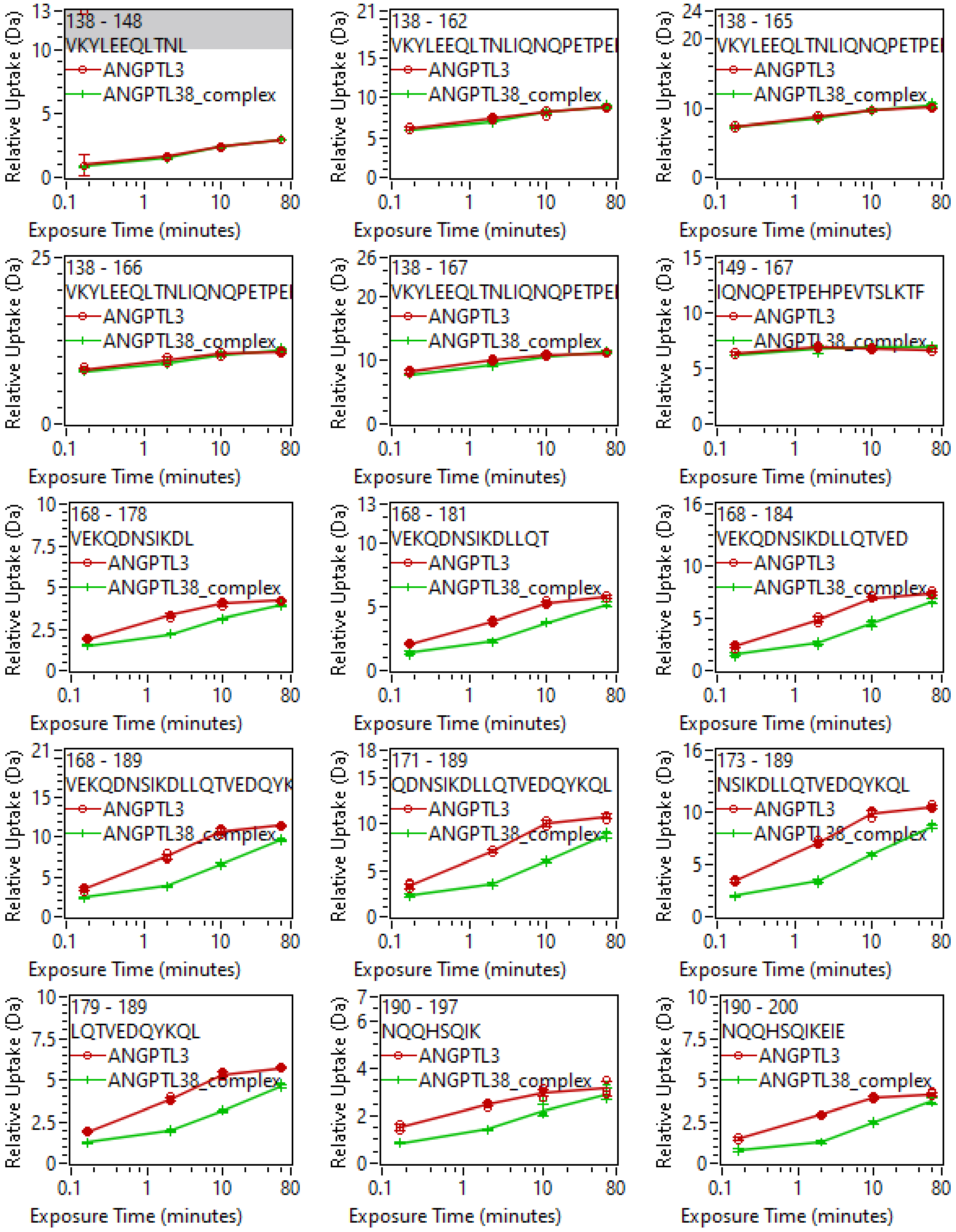

Figure S1G Continued

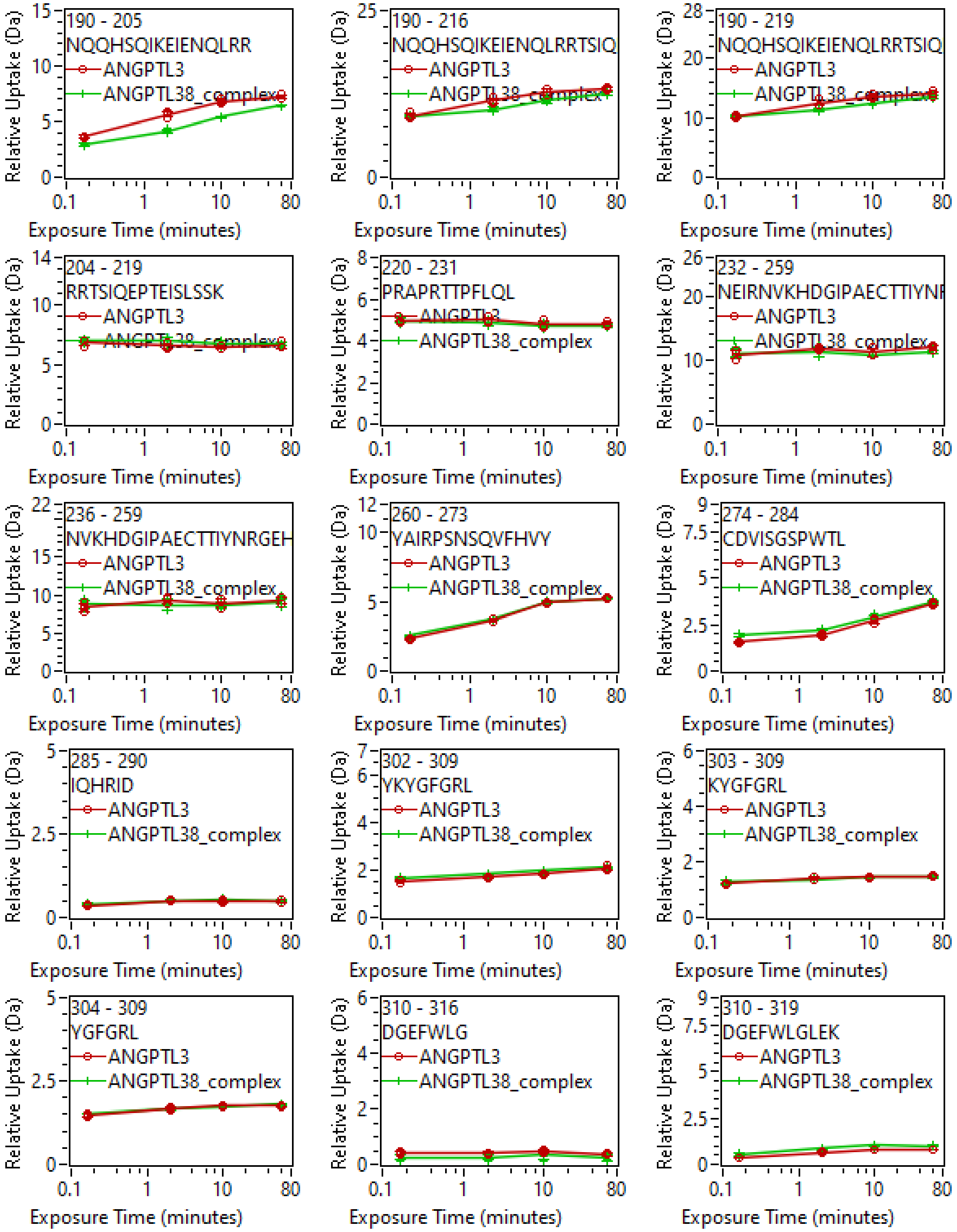

Figure S1G Continued

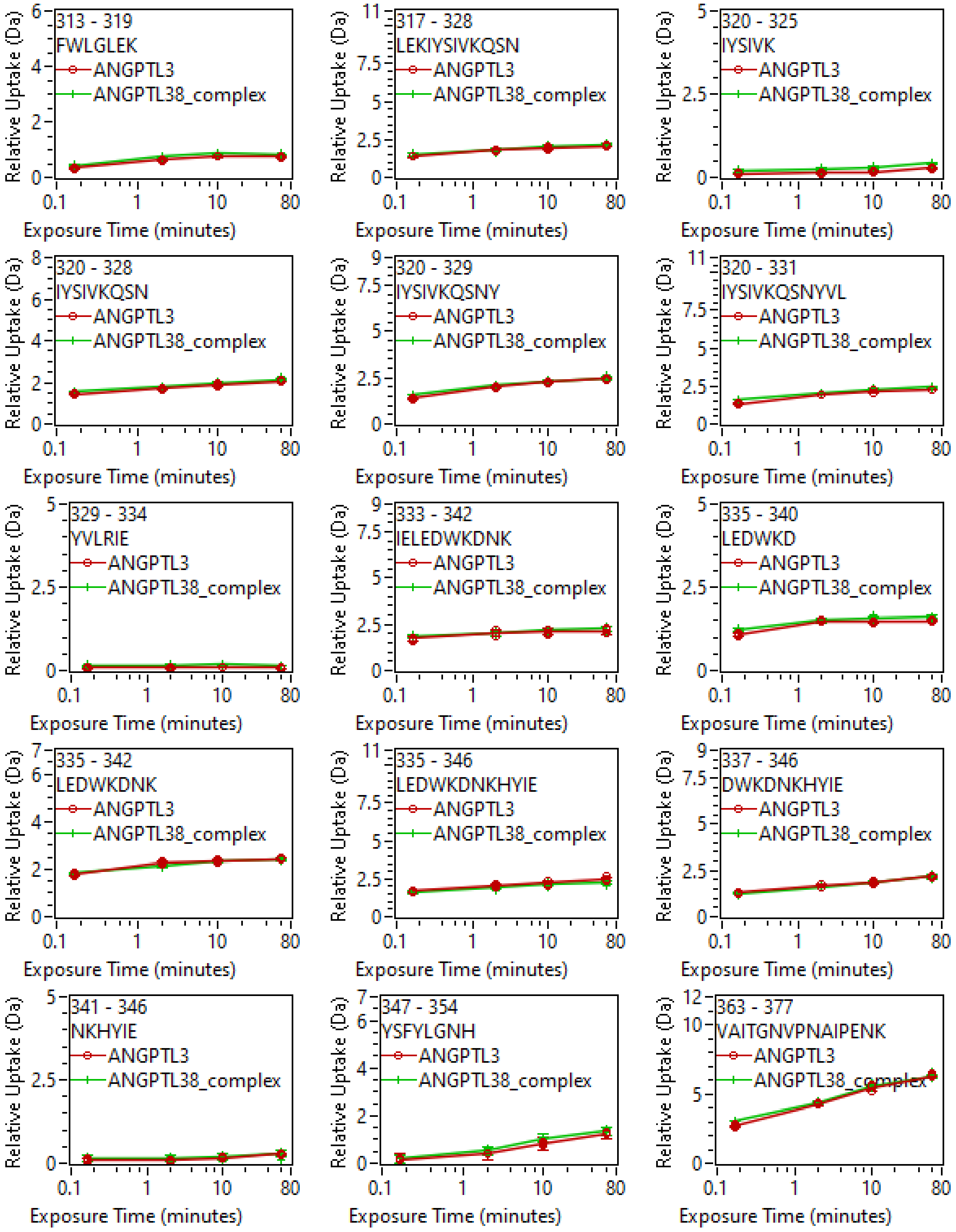

Figure S1G Continued

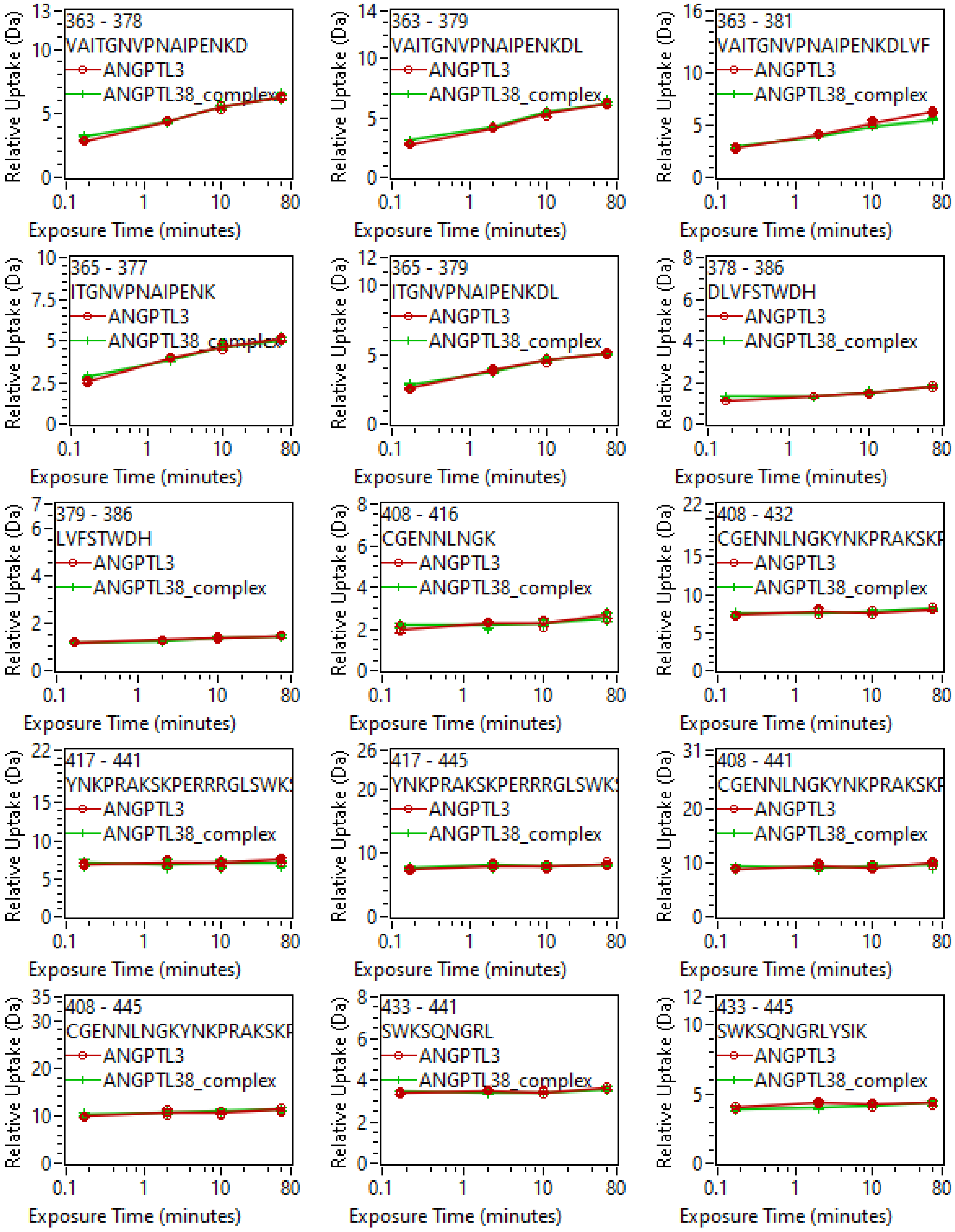

Figure S1G Continued

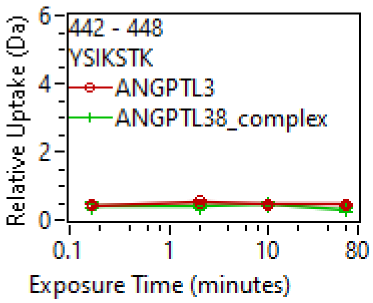

Figure S1H. Uptake plots for ANGPTL8 in ANGPTL3/8 complex compared to ANGPTL8 alone. Standard deviation in uptake difference is 0.11 Da (Experiment performed in triplicate with error bars shown).

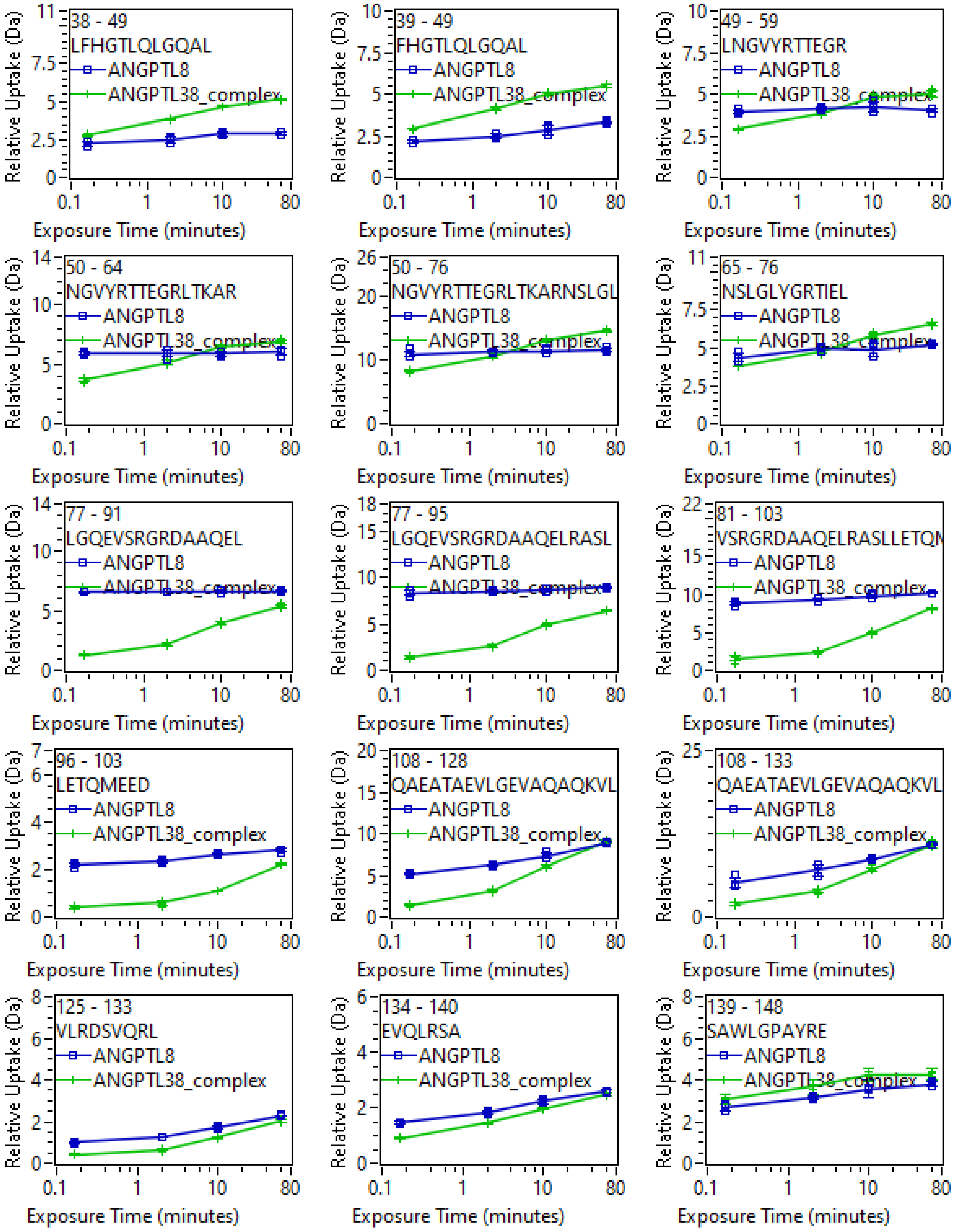

Figure S1H Continued

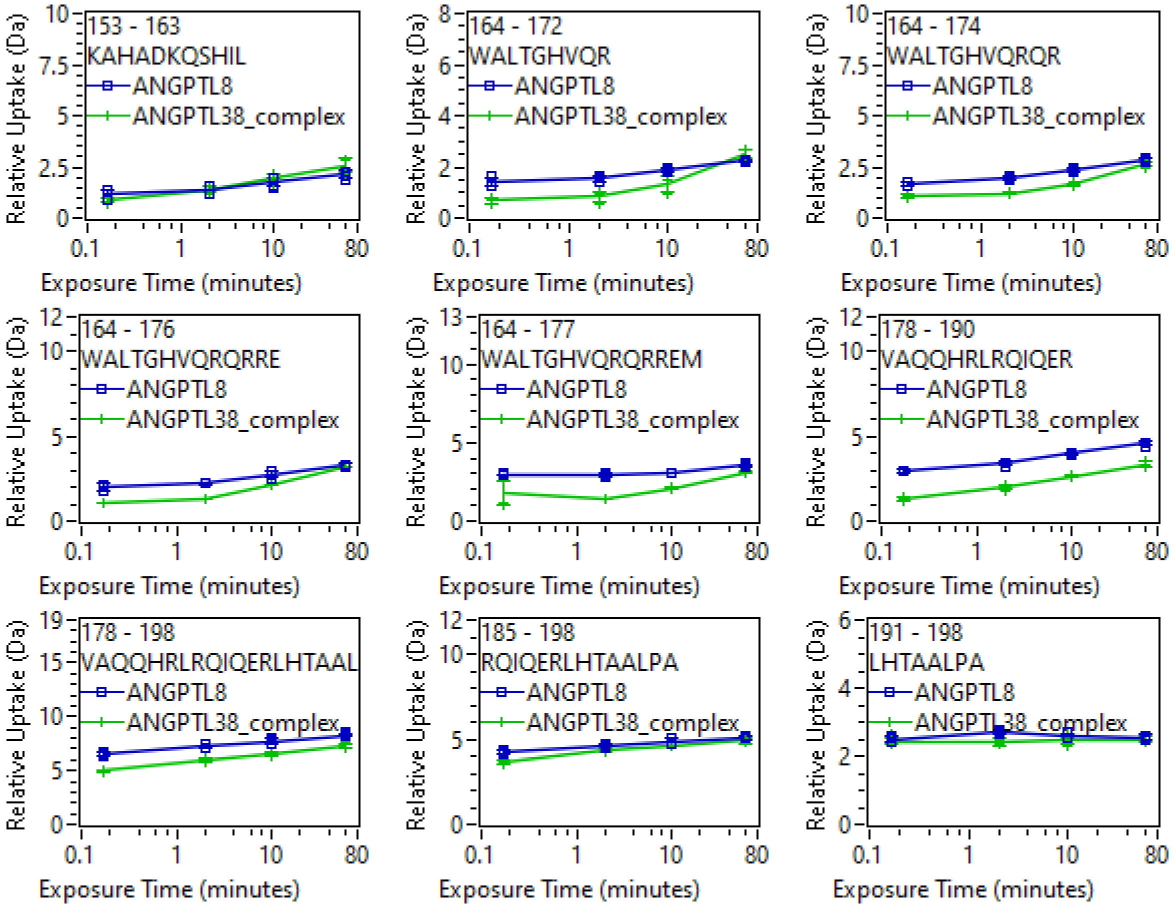

Figure S2. HDXMS comparing ANGPTL3/8 bound to LPL versus unbound ANGPTL3/8

Figure S2A. Sequence coverage for ANGPTL3 (deuterated peptides followed in the experiment)

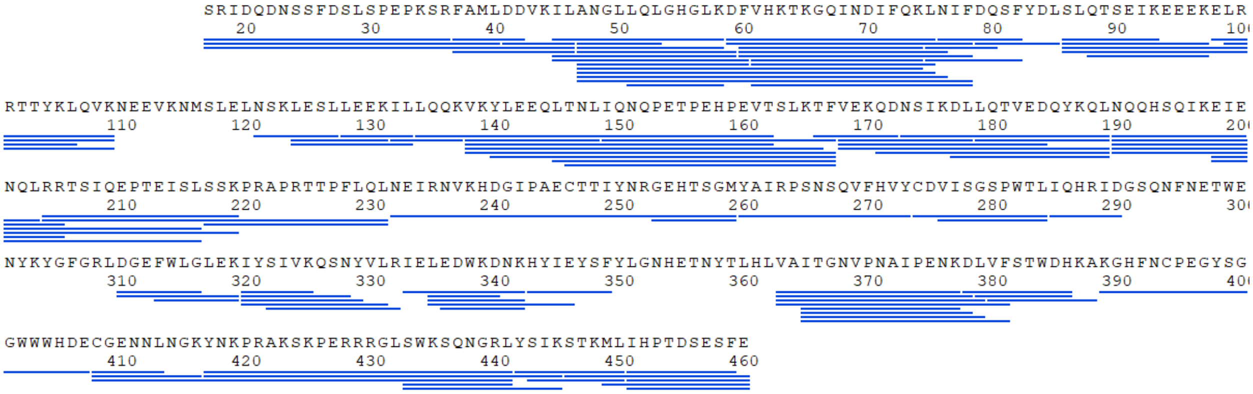

Total: 117 Peptides, 90.3% Coverage, 4.23 Redundancy

Figure S2B. Sequence coverage for ANGPTL8 (deuterated peptides followed in the experiment)

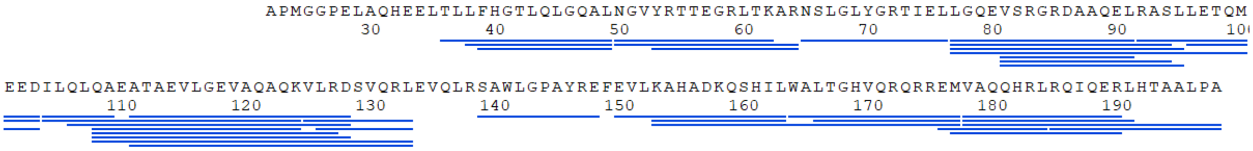

Total: 39 Peptides, 88.7% Coverage, 3.76 Redundancy

Figure S2C. Difference plot comparing the changes in deuterium uptake of ANGPTL3 in ANGPTL3/8 complex bound to LPL relative to ANGPTL3 in unbound ANGPTL3/8 complex

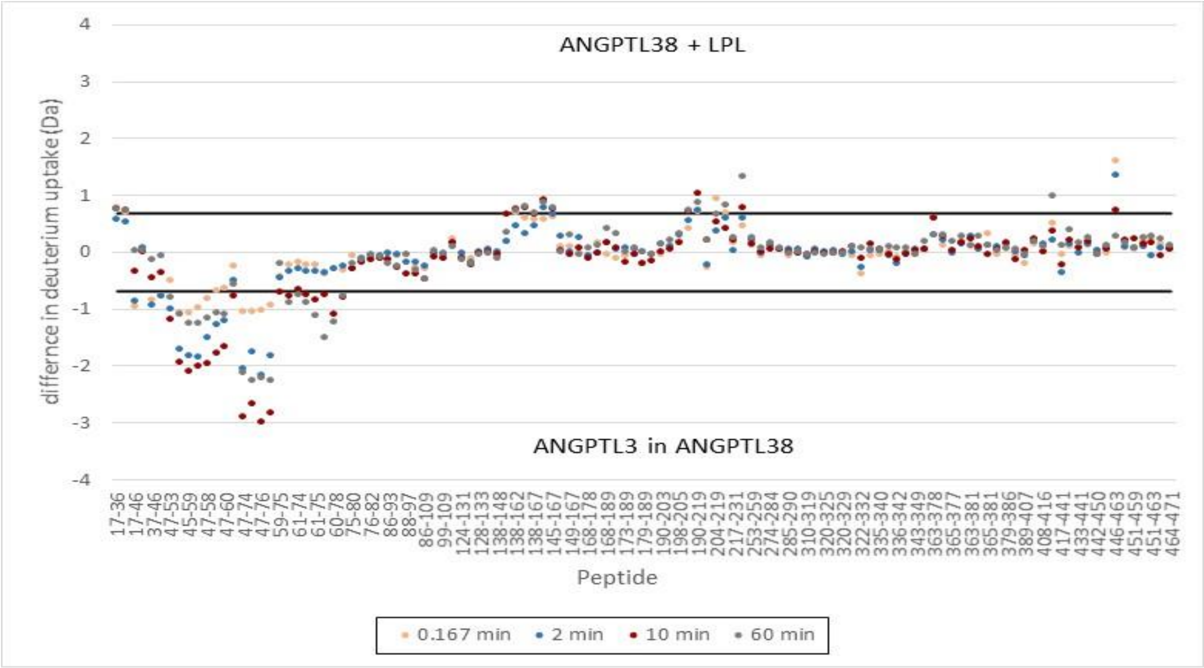

Figure S2D. Difference plot comparing the changes in deuterium uptake of ANGPTL8 in ANGPTL3/8 complex bound to LPL relative to ANGPTL8 in unbound ANGPTL3/8 complex

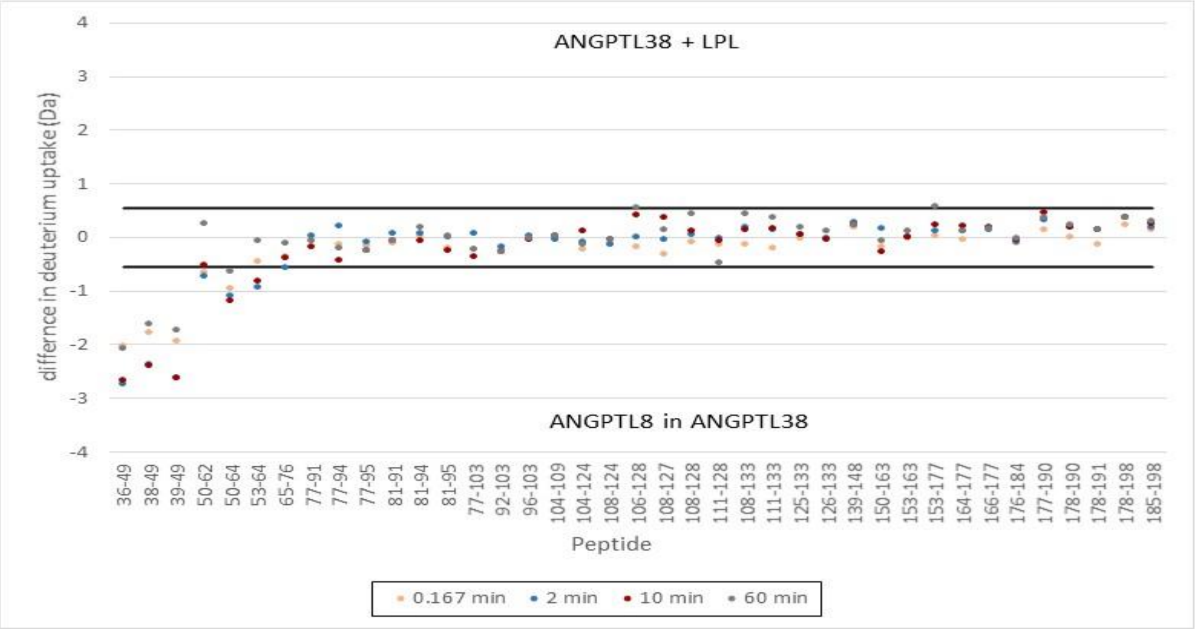

Figure S2E. Heat map of the relative deuterium uptake ANGPTL3 in ANGPTL3/8 complex bound to LPL relative to ANGPTL3 in unbound ANGPTL3/8 complex

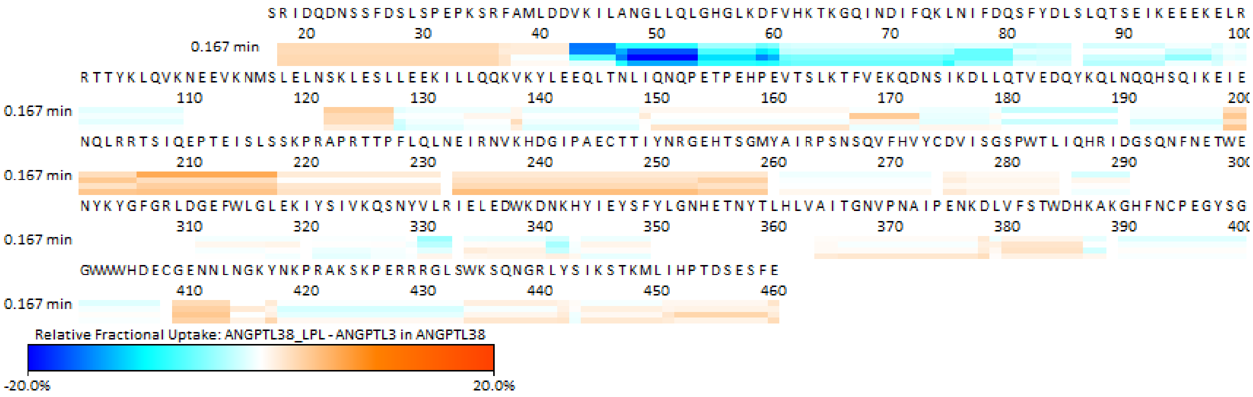

Figure S2F. Heat map of the relative deuterium uptake of ANGPTL8 in ANGPTL3/8 complex bound to LPL relative to ANGPTL8 in unbound ANGPTL3/8 complex

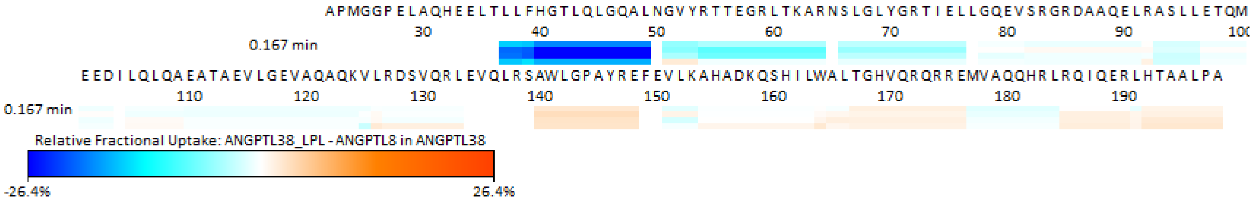

Figure S2G. ANGPTL3 uptake plots for ANGPTL3/8 bound to LPL compared to unbound ANGPTL3/8. Standard deviation in uptake difference is 0.23 Da (Experiment performed in triplicate with error bars shown).

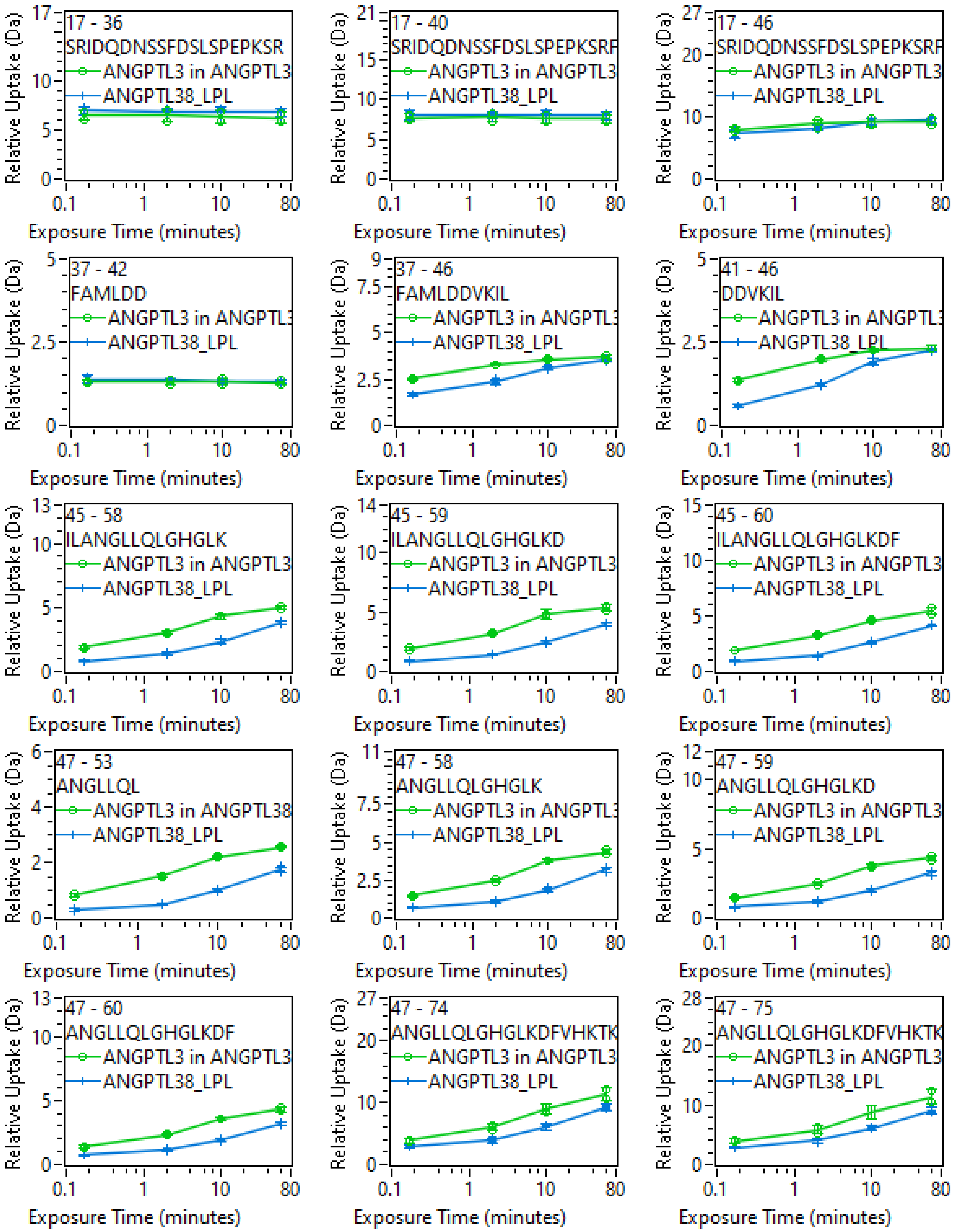

Figure S2G continued

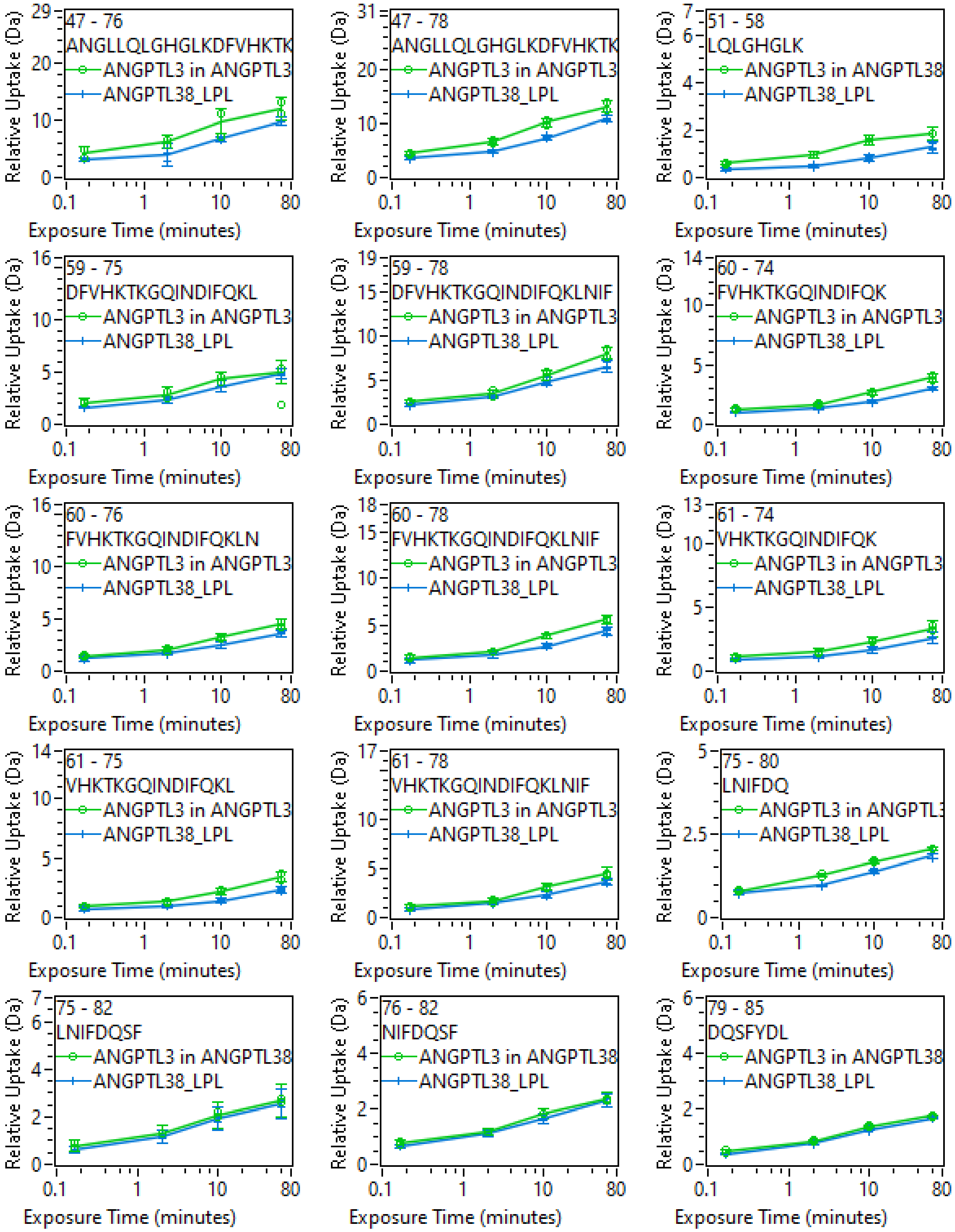

Figure S2G continued

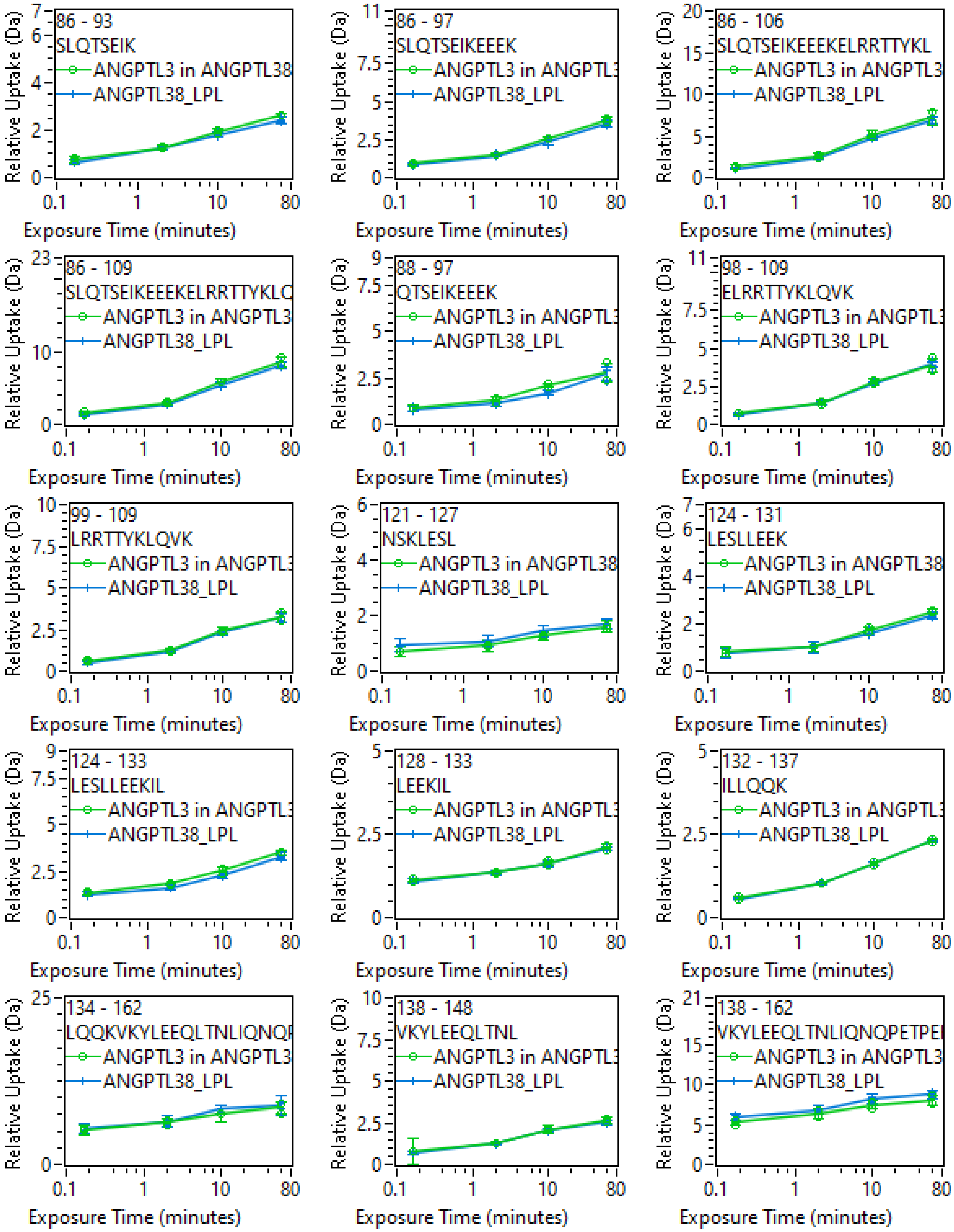

Figure S2G continued

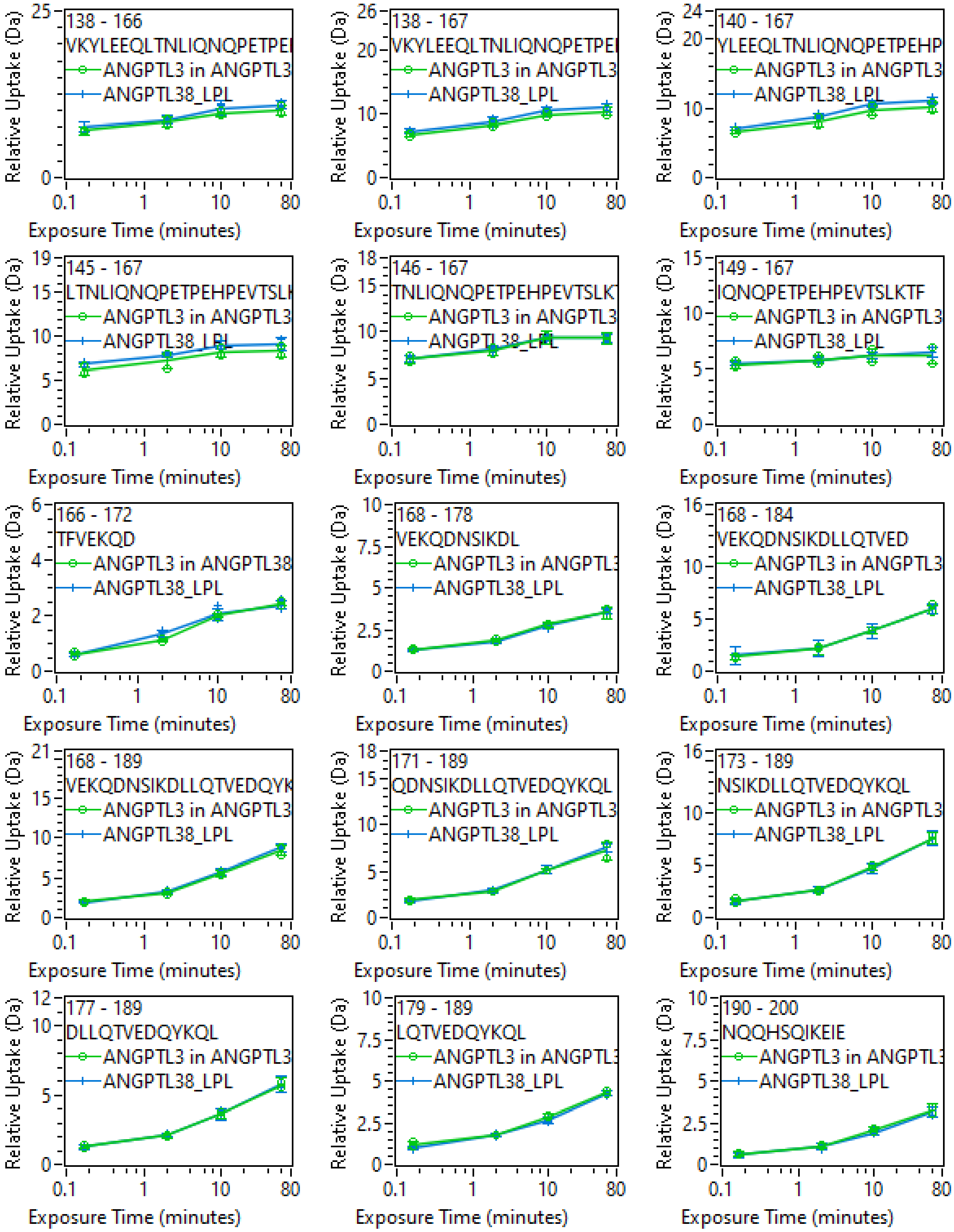

Figure S2G continued

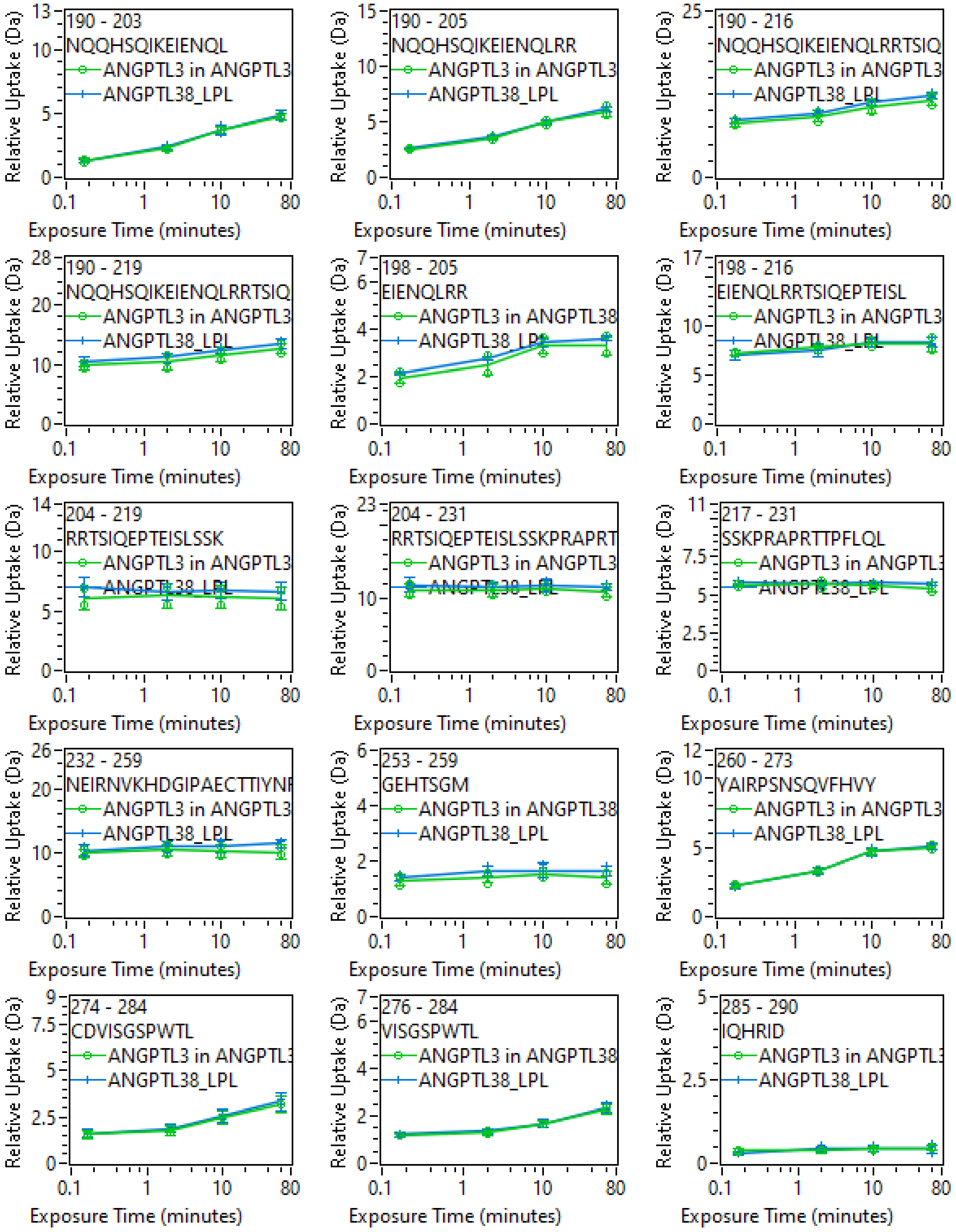

Figure S2G continued

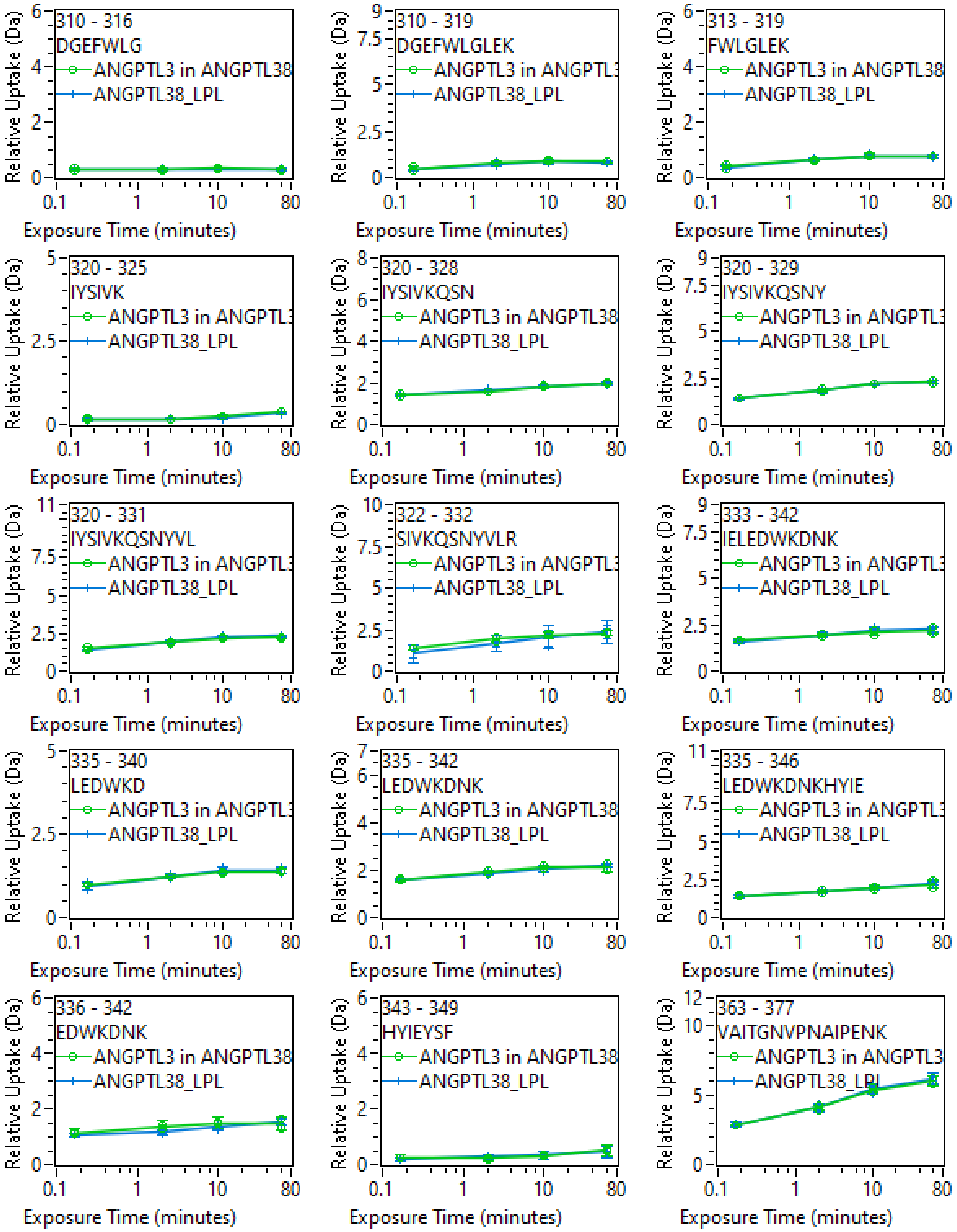

Figure S2G continued

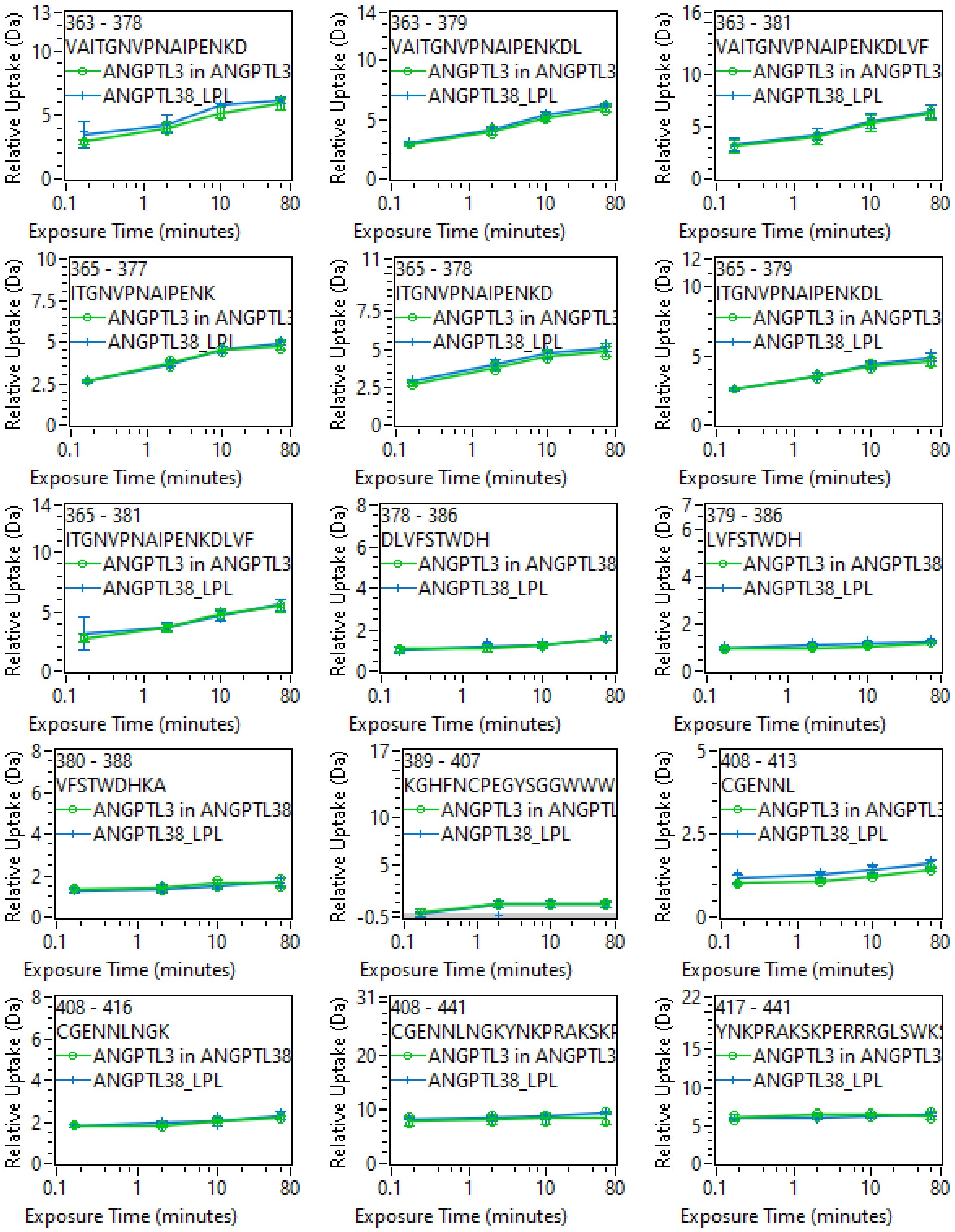

Figure S2G continued

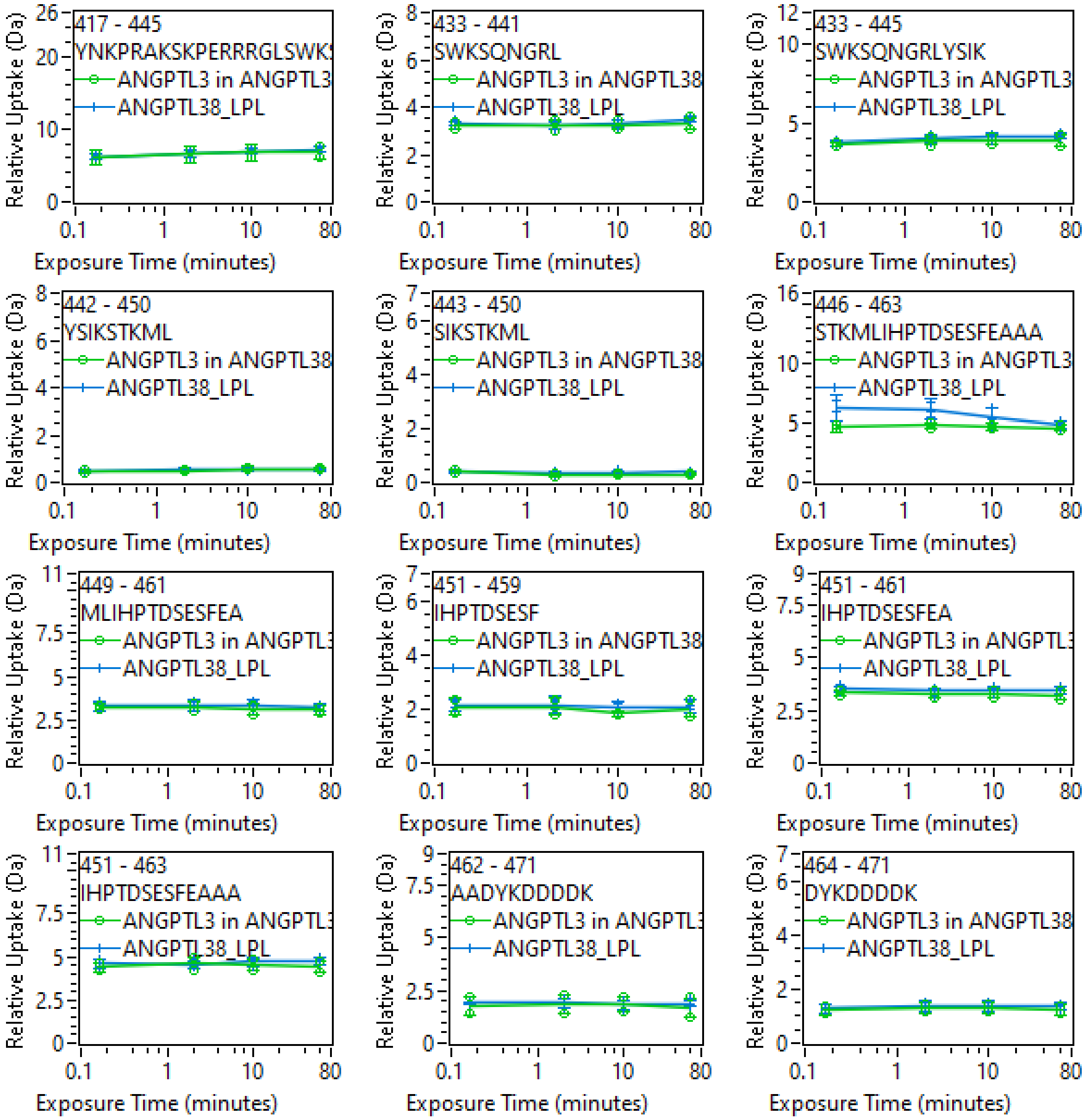

Figure S2H. ANGPTL8 uptake plots for ANGPTL3/8 bound to LPL compared to unbound ANGPTL3/8. Standard deviation in uptake difference is 0.18 Da (Experiment performed in triplicate with error bars shown).

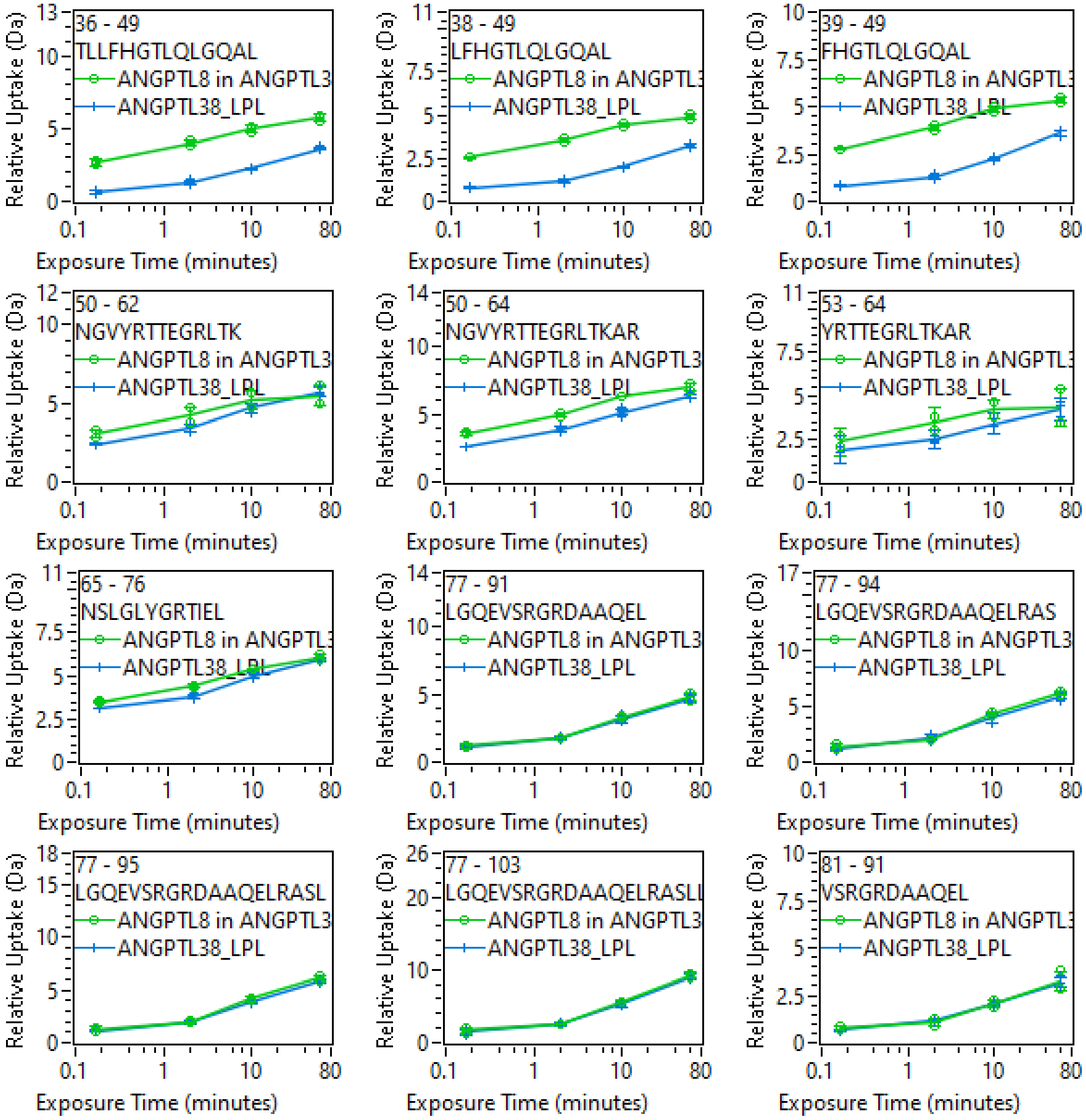

Figure S2H continued

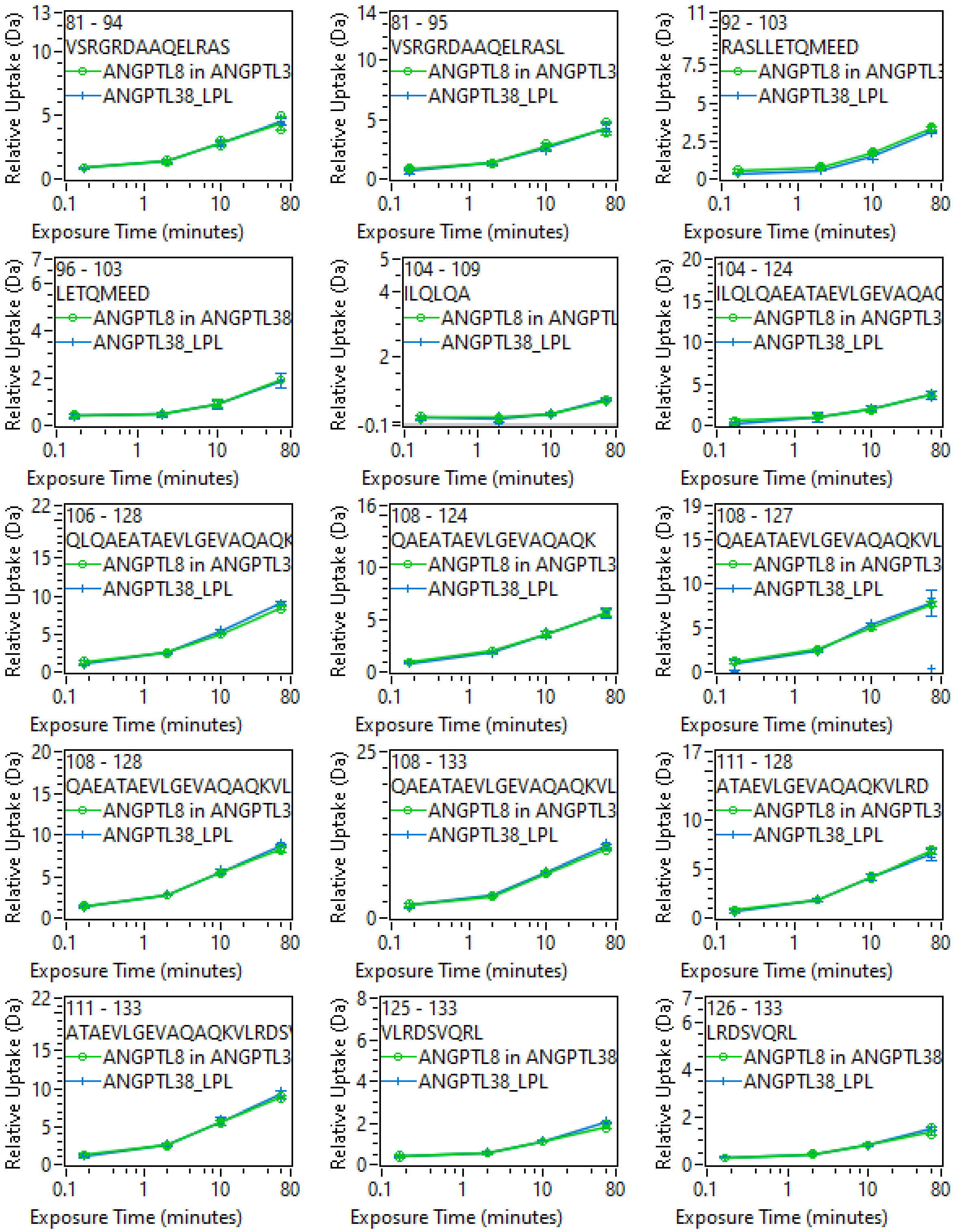

Figure S2H continued

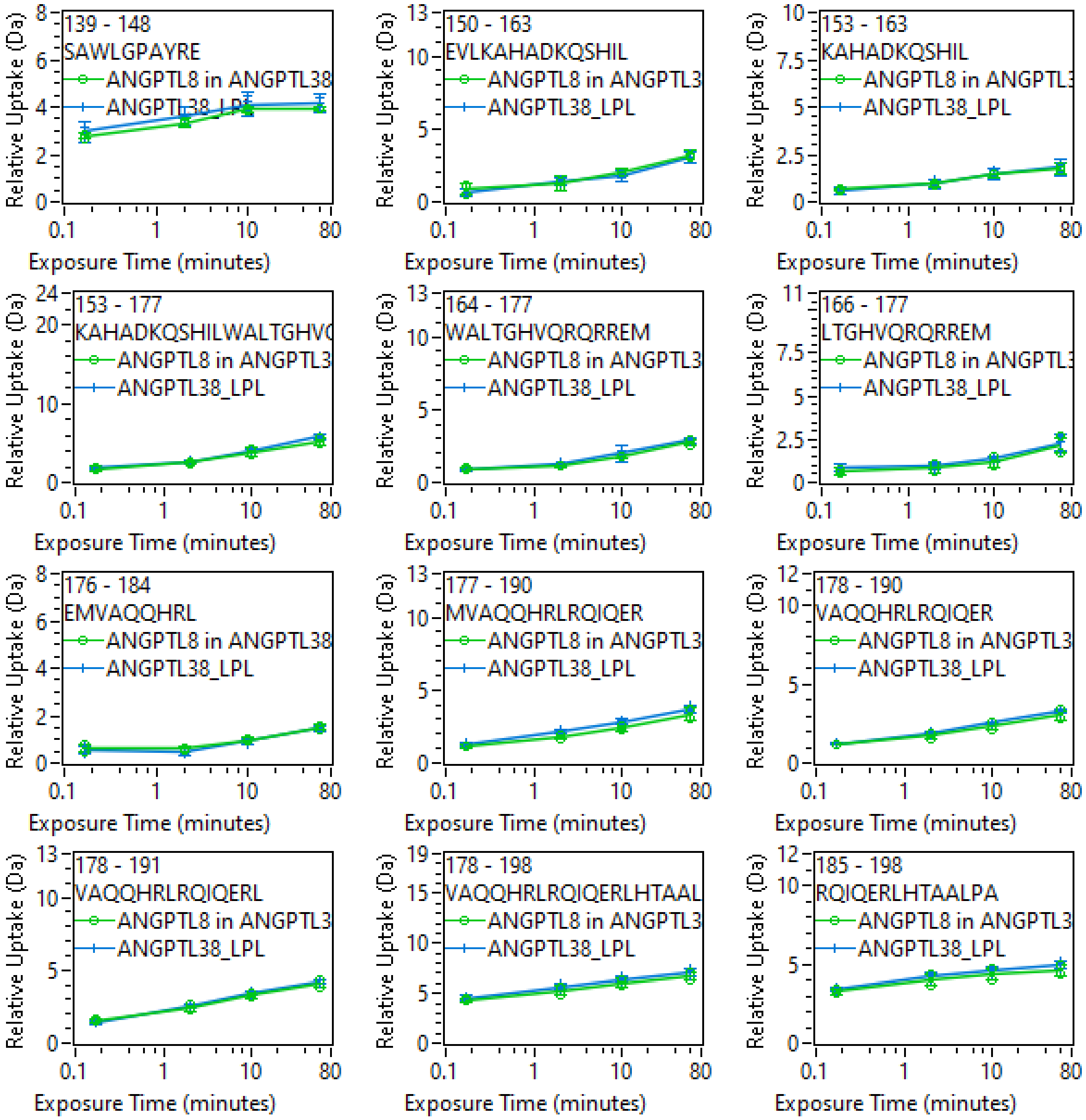

Figure S3. HDXMS comparing ANGPTL3/8 bound to ApoA5 to unbound ANGPTL3/8

Figure S3A. Sequence coverage for ANGPTL3 (deuterated peptides followed in the experiment)

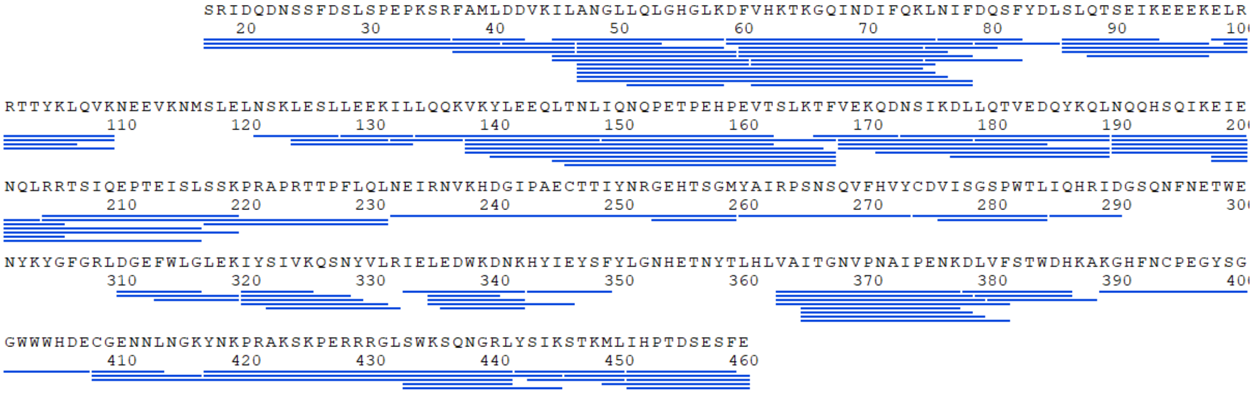

Total: 117 Peptides, 90.3% Coverage, 4.23 Redundancy

Figure S3B. Sequence coverage for ANGPTL8 (deuterated peptides followed in the experiment)

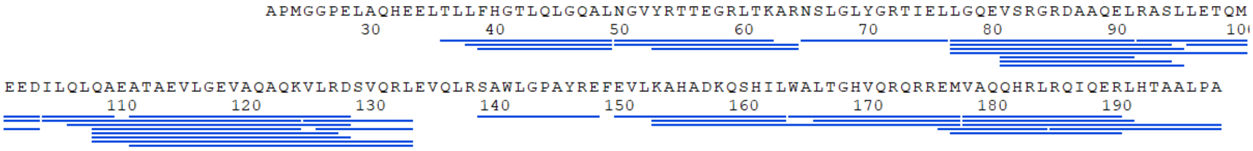

Total: 39 Peptides, 88.7% Coverage, 3.76 Redundancy

Figure S3C. Difference plot comparing the changes in deuterium uptake of ANGPTL3 in ANGPTL3/8 complex bound to ApoA5 relative to ANGPTL3 in unbound ANGPTL3/8 complex

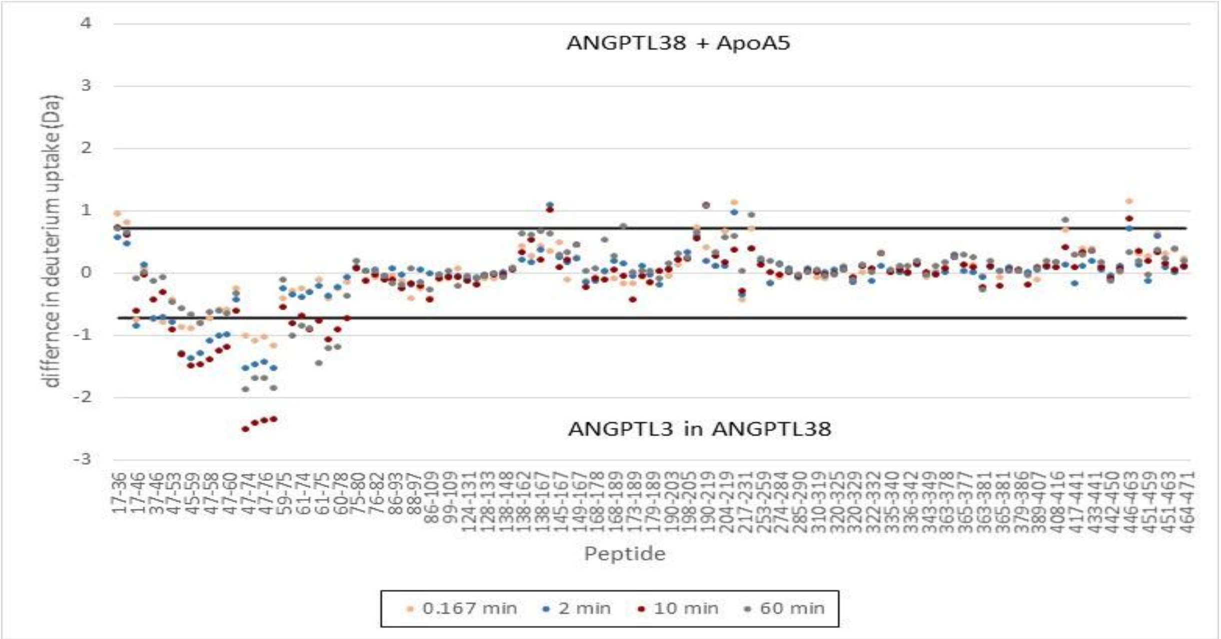

Figure S3D. Difference plot comparing the changes in deuterium uptake of ANGPTL8 in ANGPTL3/8 complex bound to ApoA5 relative to ANGPTL8 in unbound ANGPTL3/8 complex

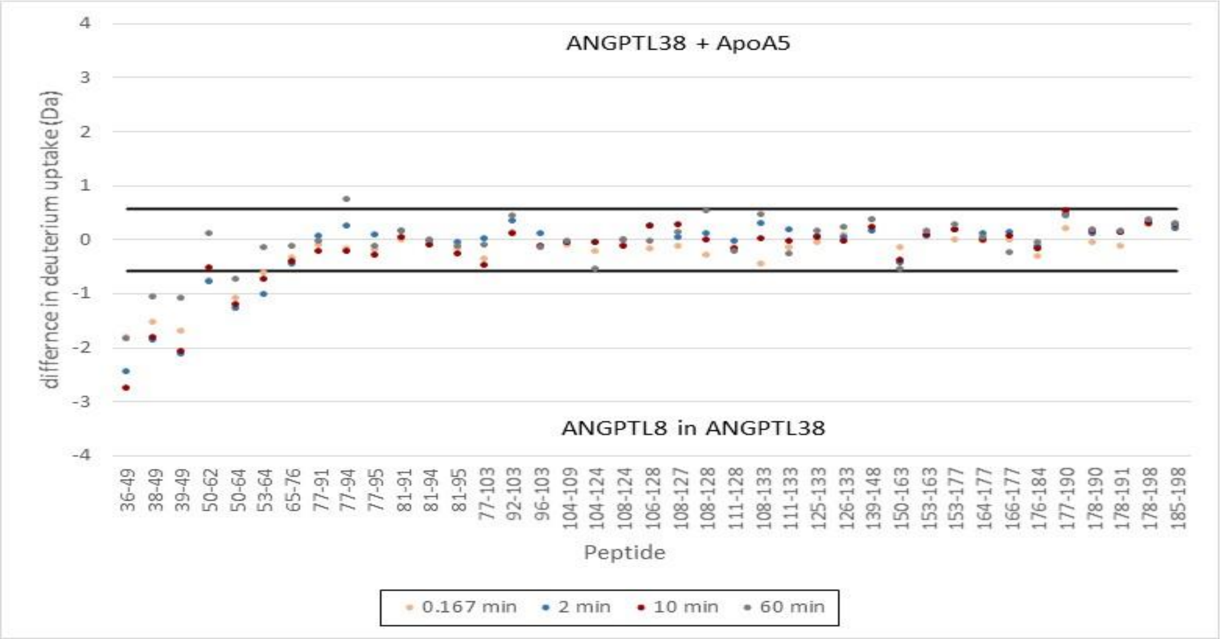

Figure S3E. Heat map of the relative deuterium uptake of ANGPTL3 in ANGPTL3/8 complex bound to ApoA5 relative to ANGPTL3 in unbound ANGPTL3/8 complex

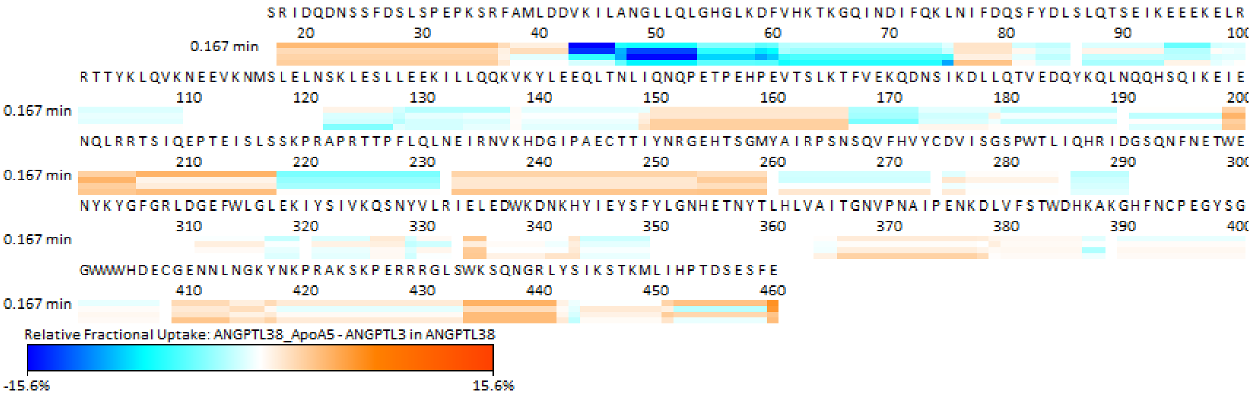

Figure S3F. Heat map of the relative deuterium uptake ANGPTL8 in ANGPTL3/8 complex bound to ApoA5 relative to ANGPTL8 in unbound ANGPTL3/8 complex

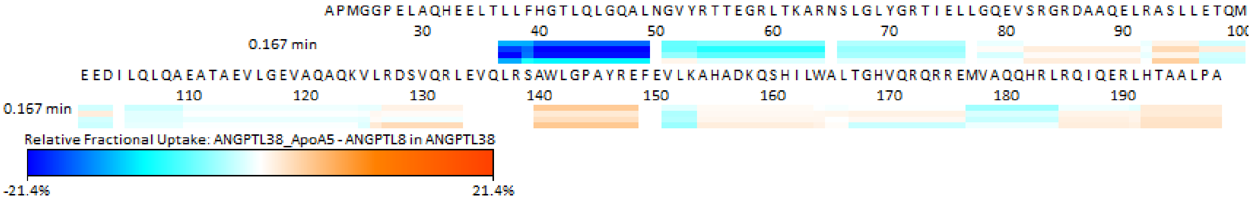

Figure S3G. ANGPTL3 uptake plots for ANGPTL3/8 bound to ApoA5 compared to unbound ANGPTL3/8. Standard deviation in uptake difference is 0.24 Da (Experiment performed in triplicate with error bars shown).

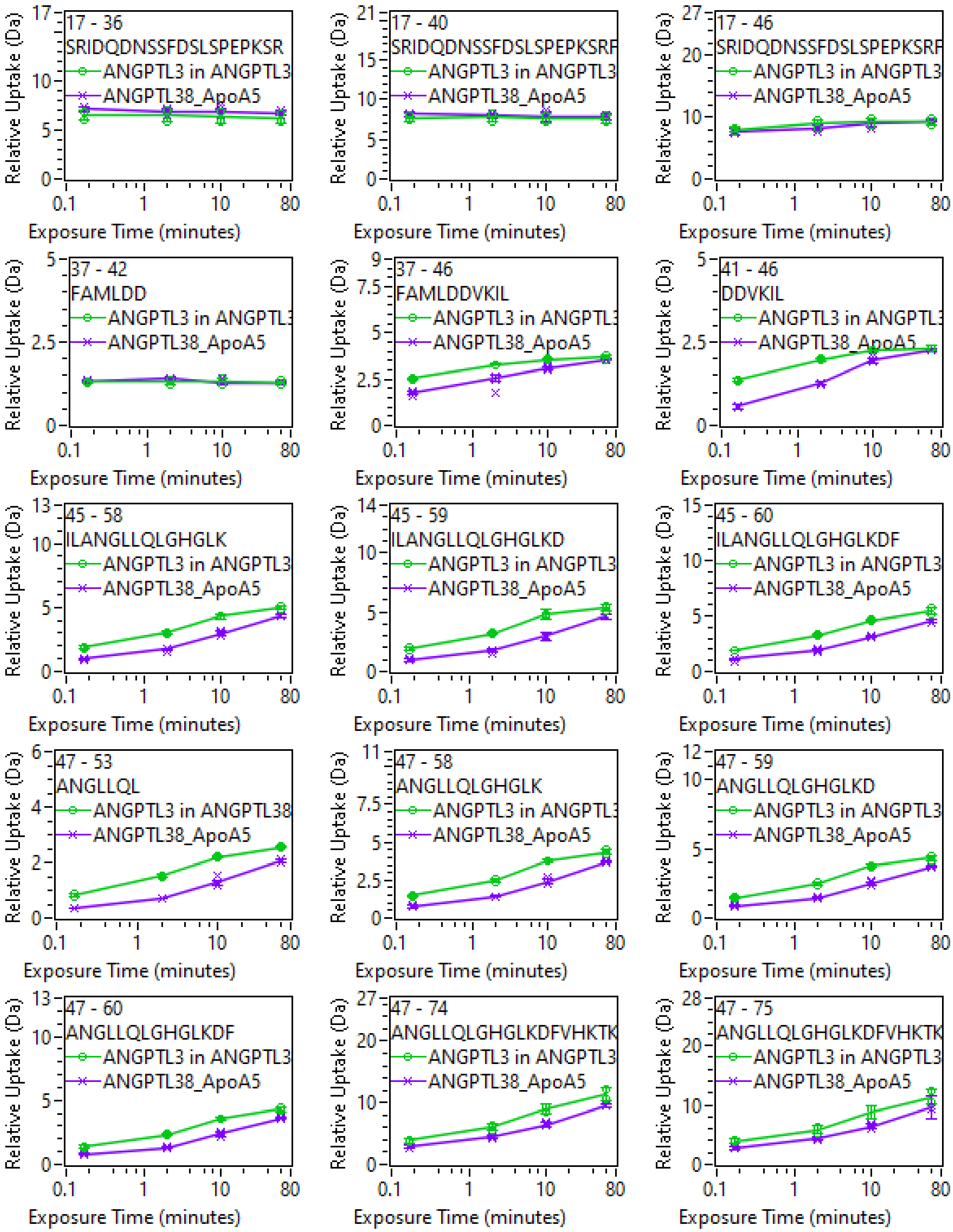

Figure S3G continued

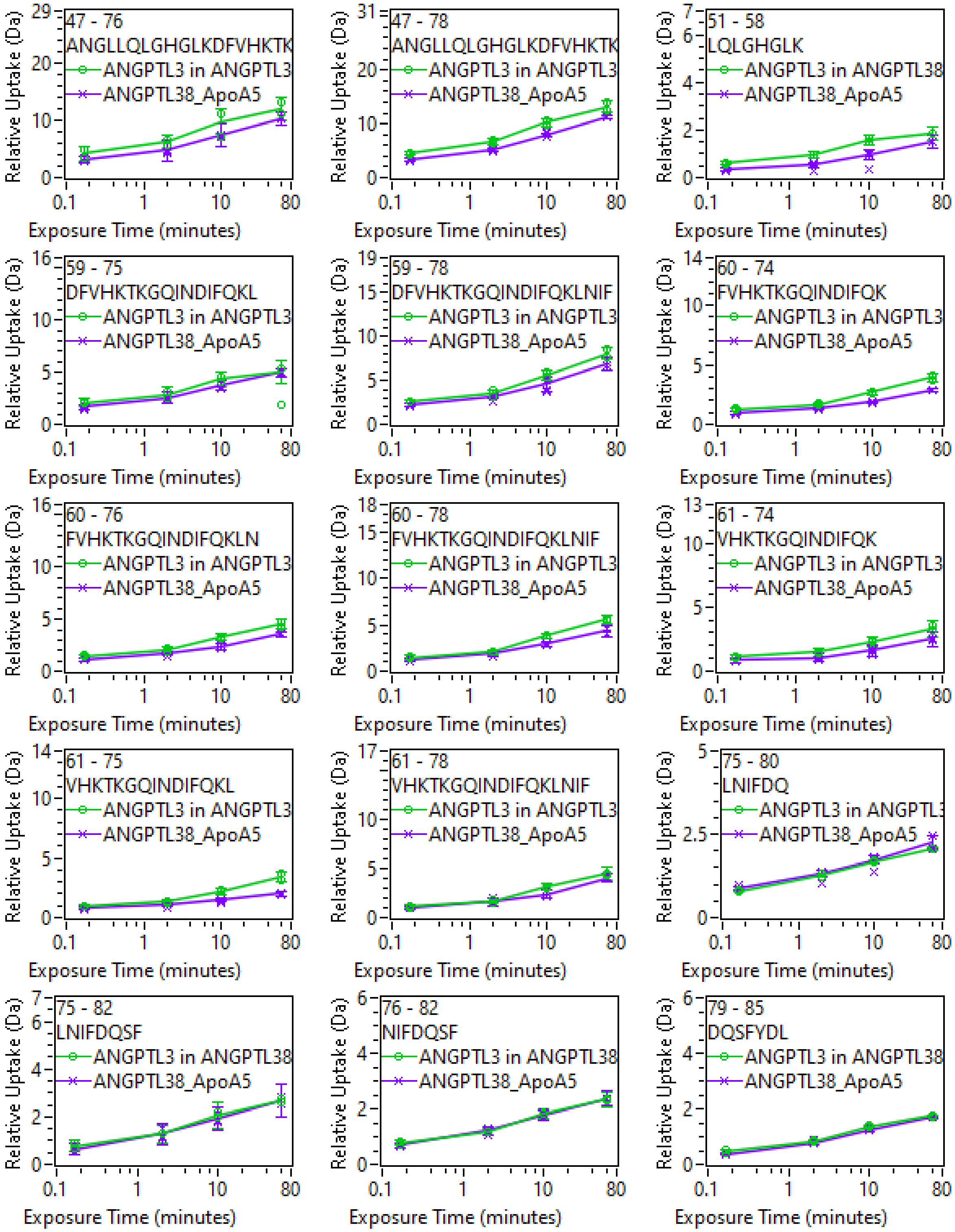

Figure S3G continued

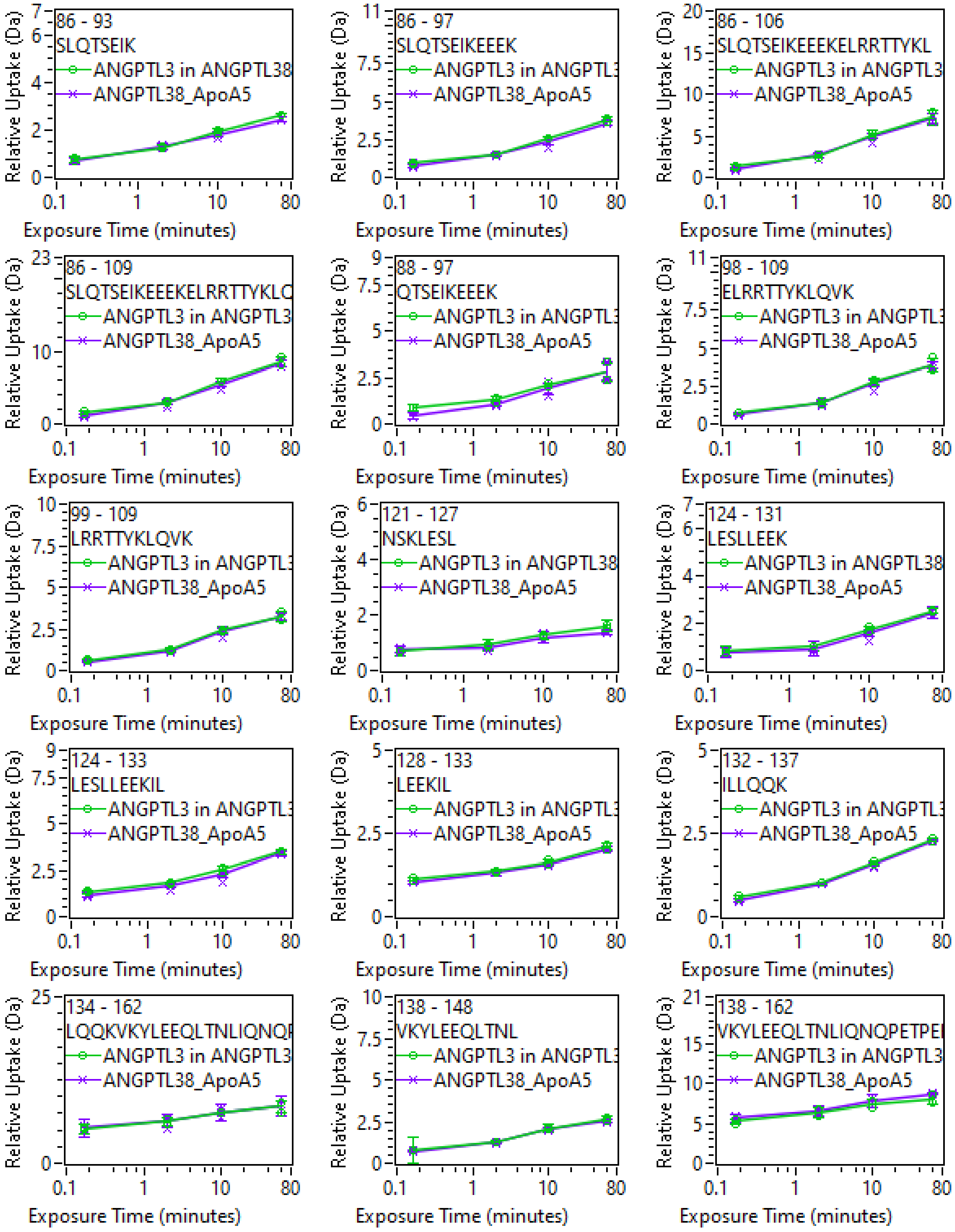

Figure S3G continued

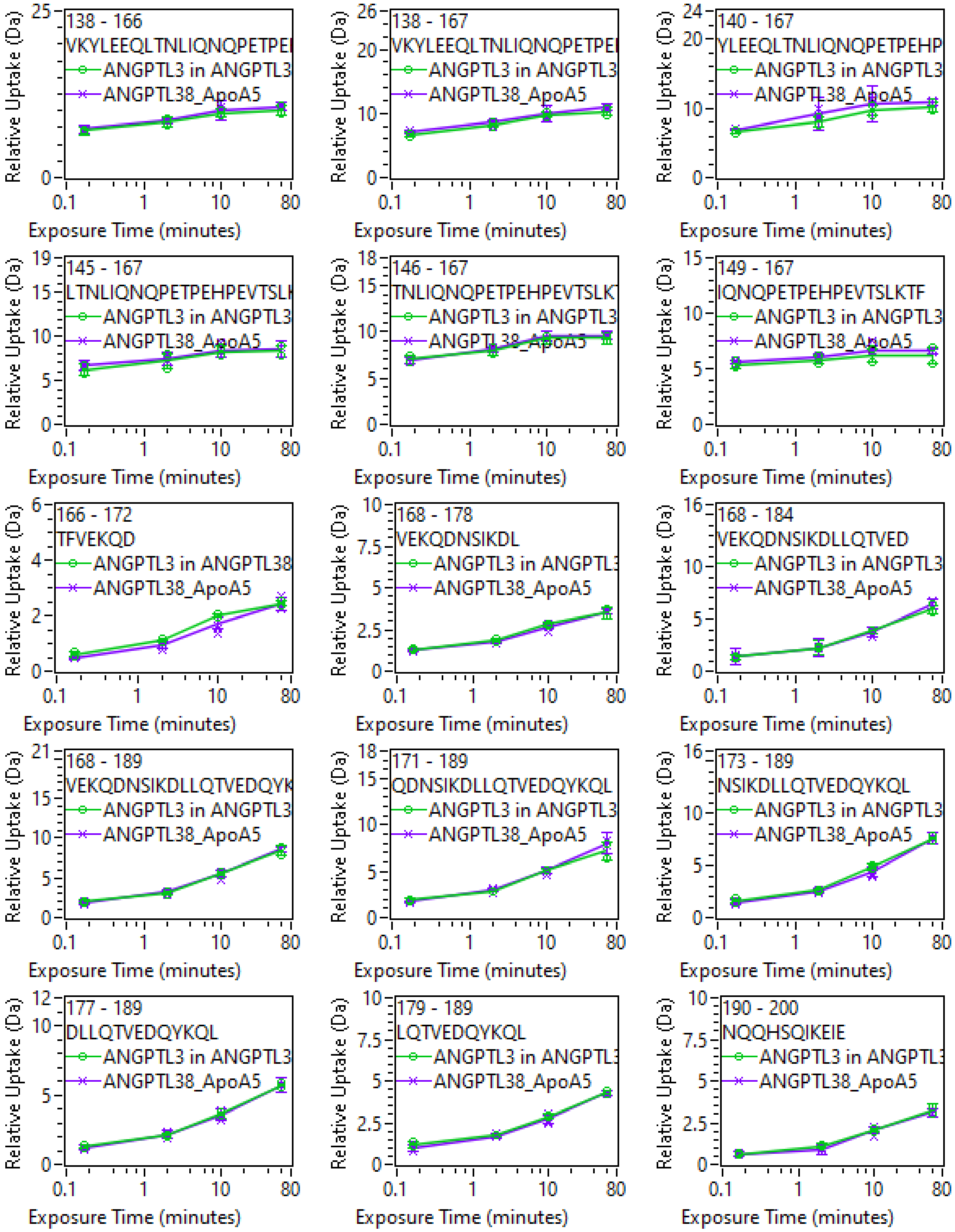

Figure S3G continued

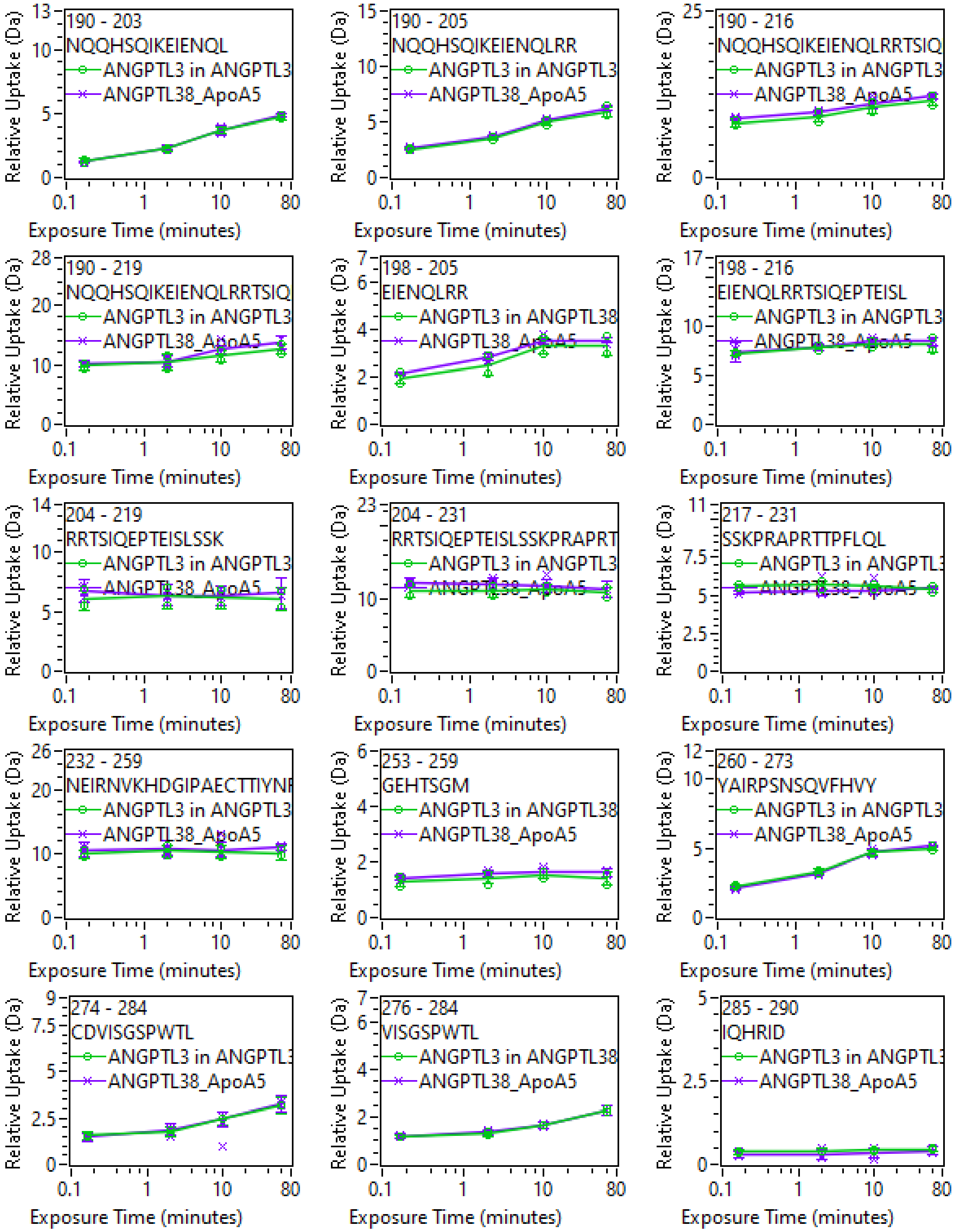

Figure S3G continued

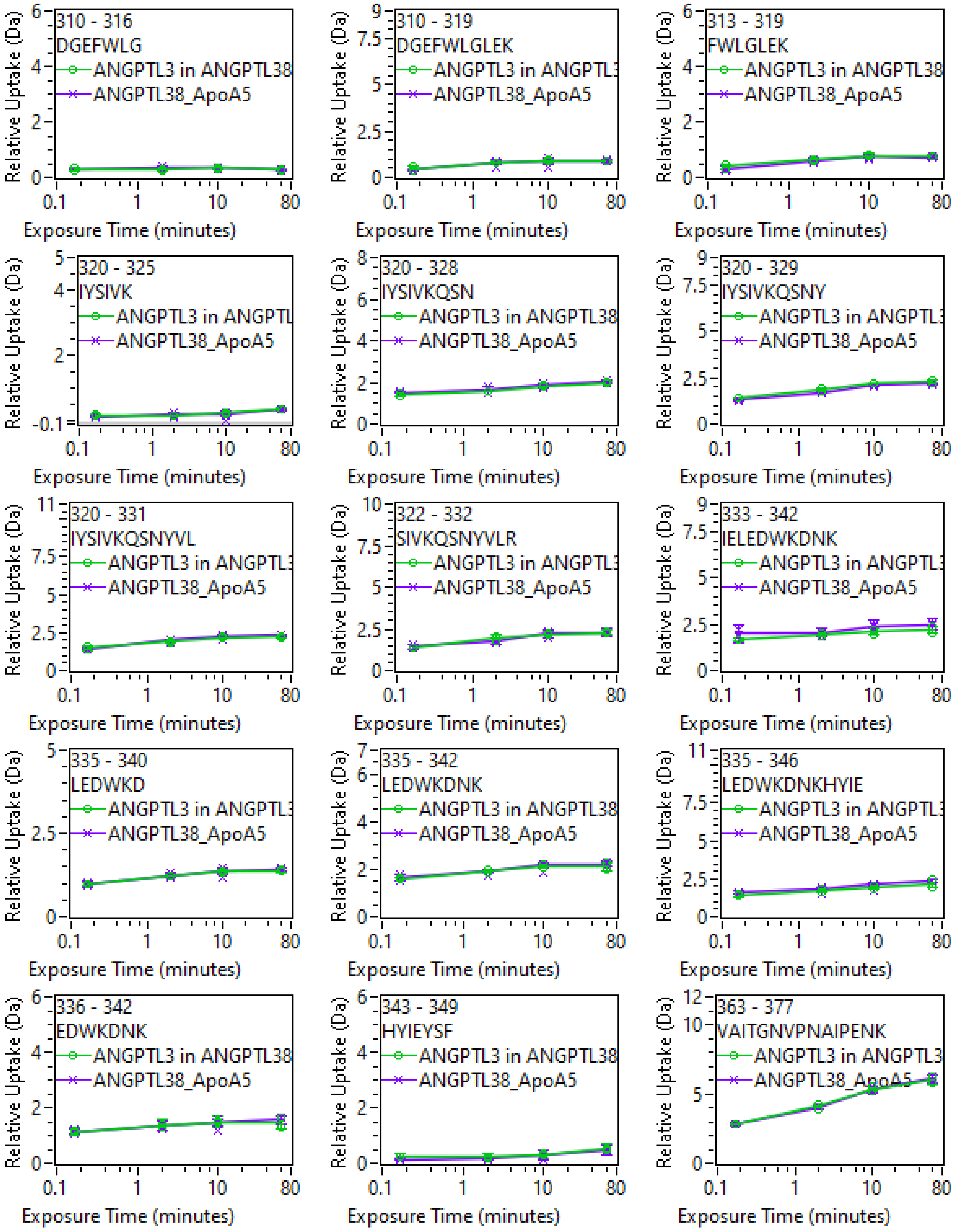

Figure S3G continued

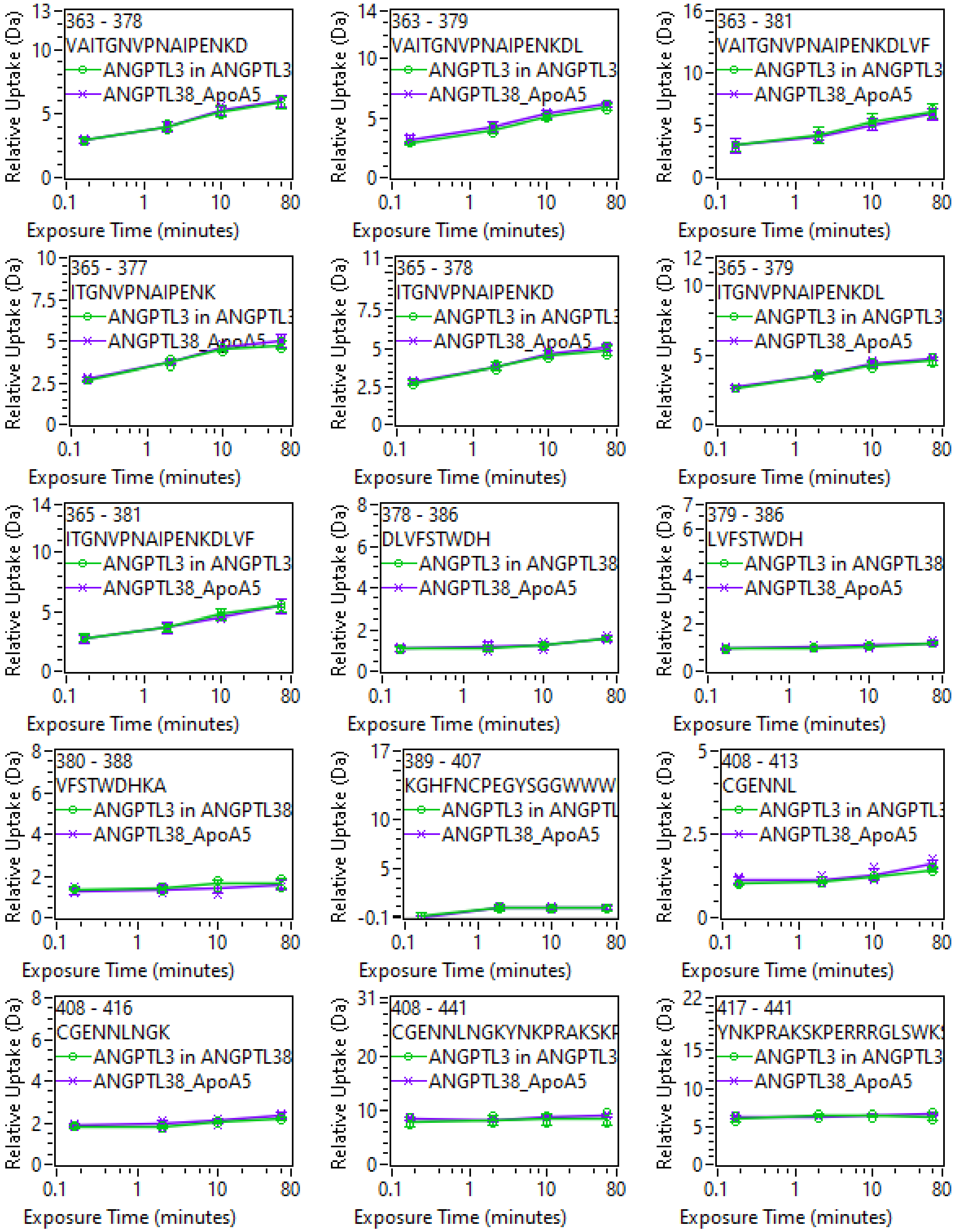

Figure S3G continued

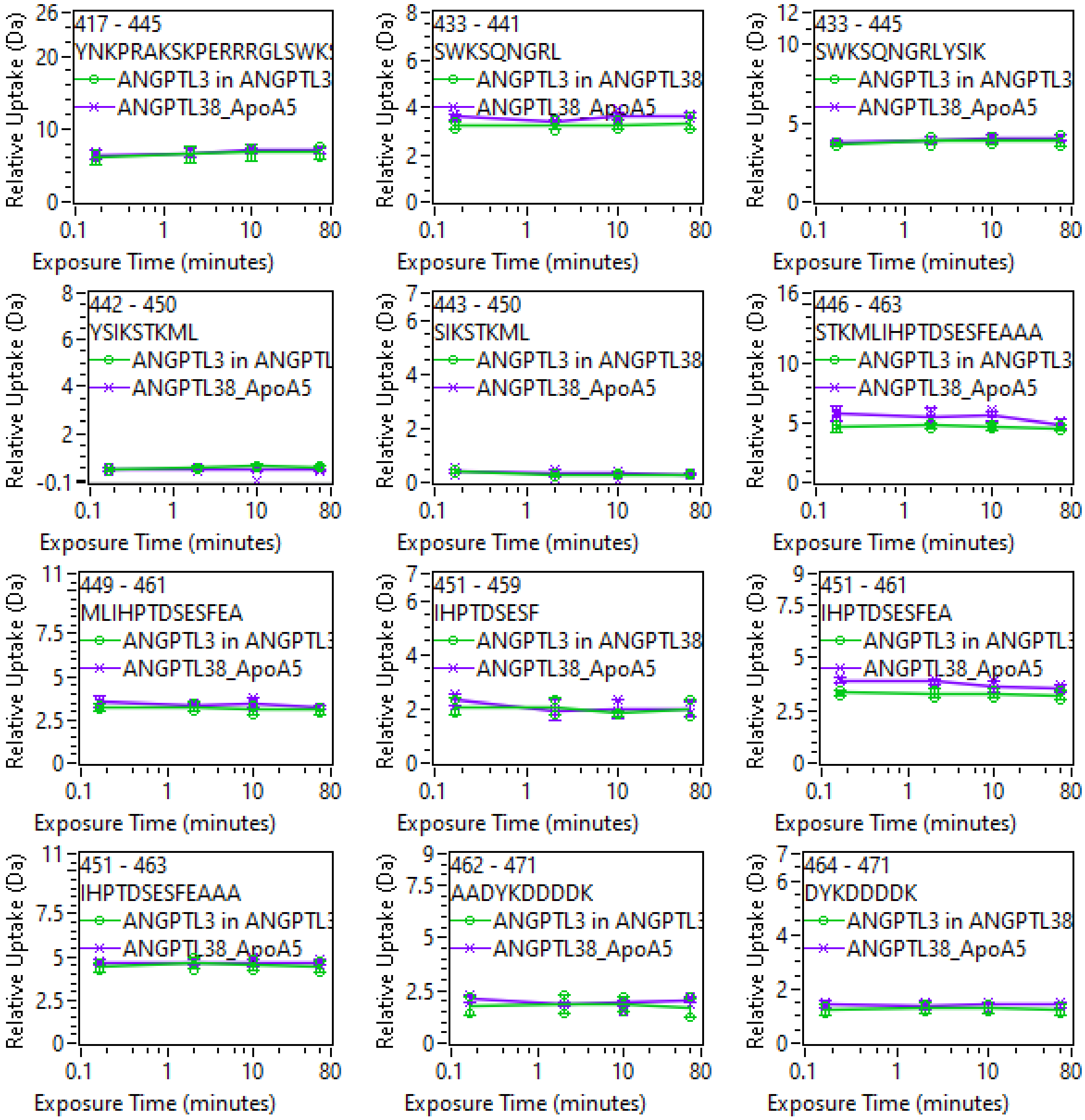

Figure S3H. ANGPTL8 uptake plots for ANGPTL3/8 bound to ApoA5 compared to unbound ANGPTL3/8. Standard deviation in uptake difference is 0.19 Da (Experiment performed in triplicate with error bars shown).

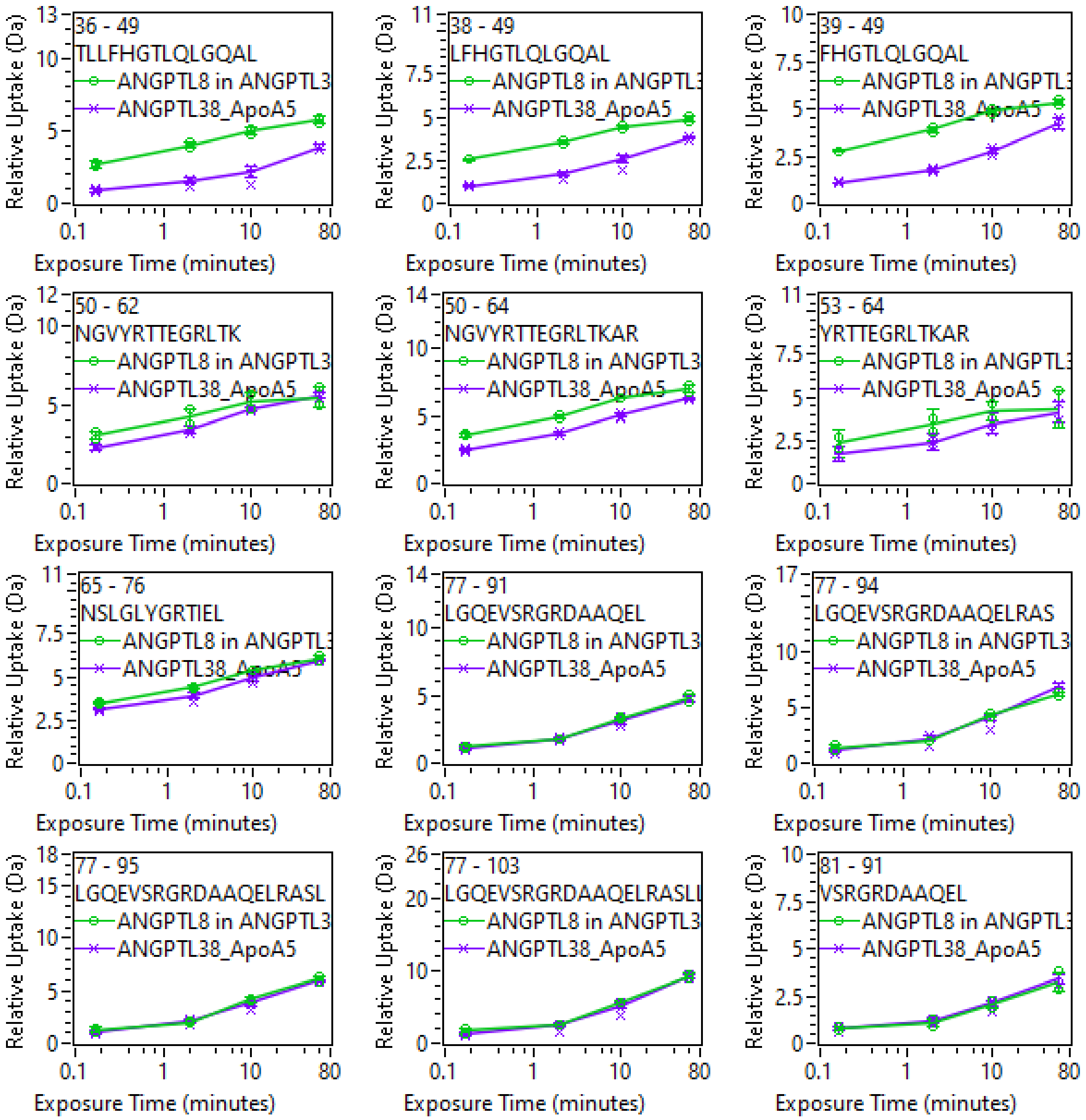

Figure S3H continued

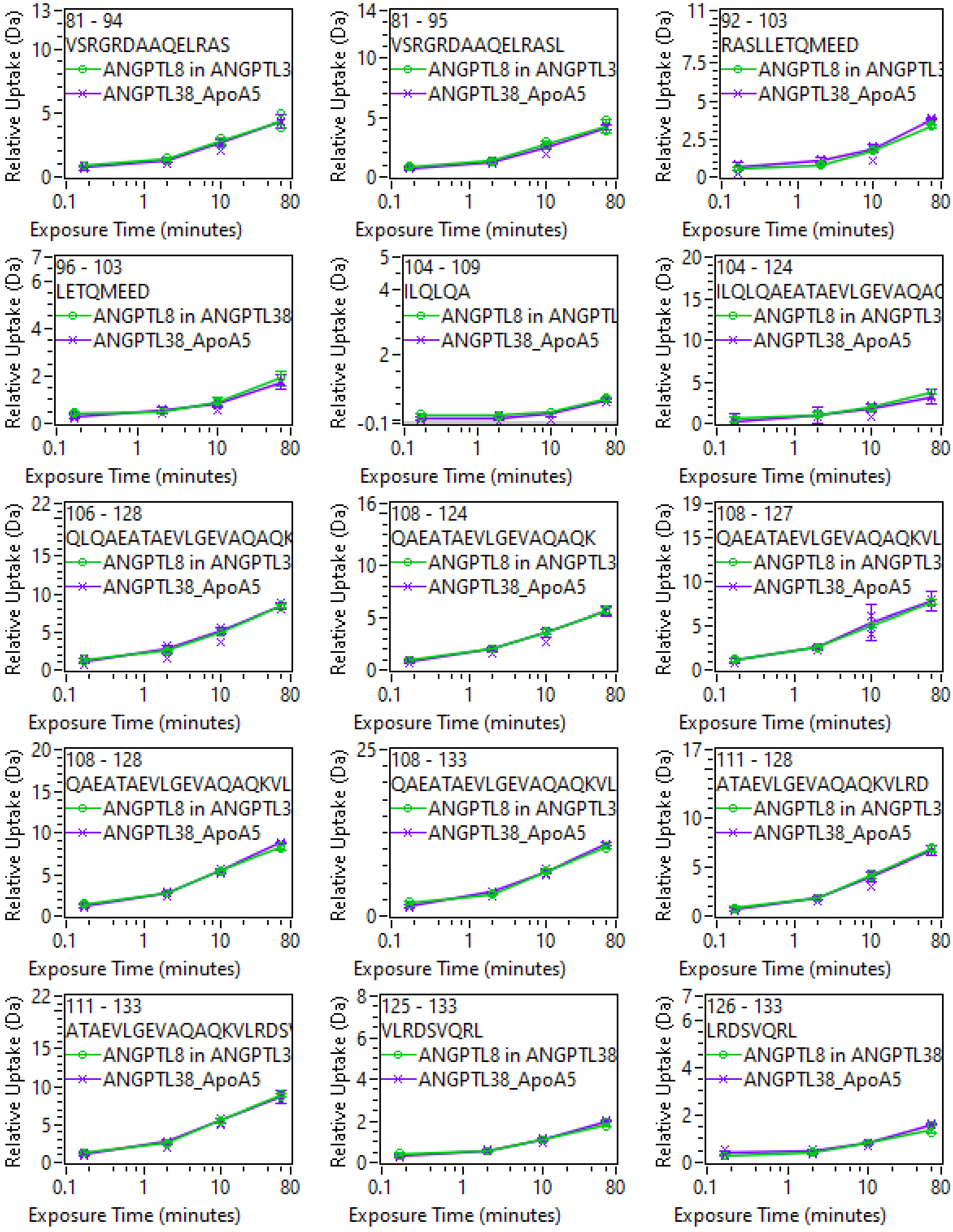

Figure S3H continued

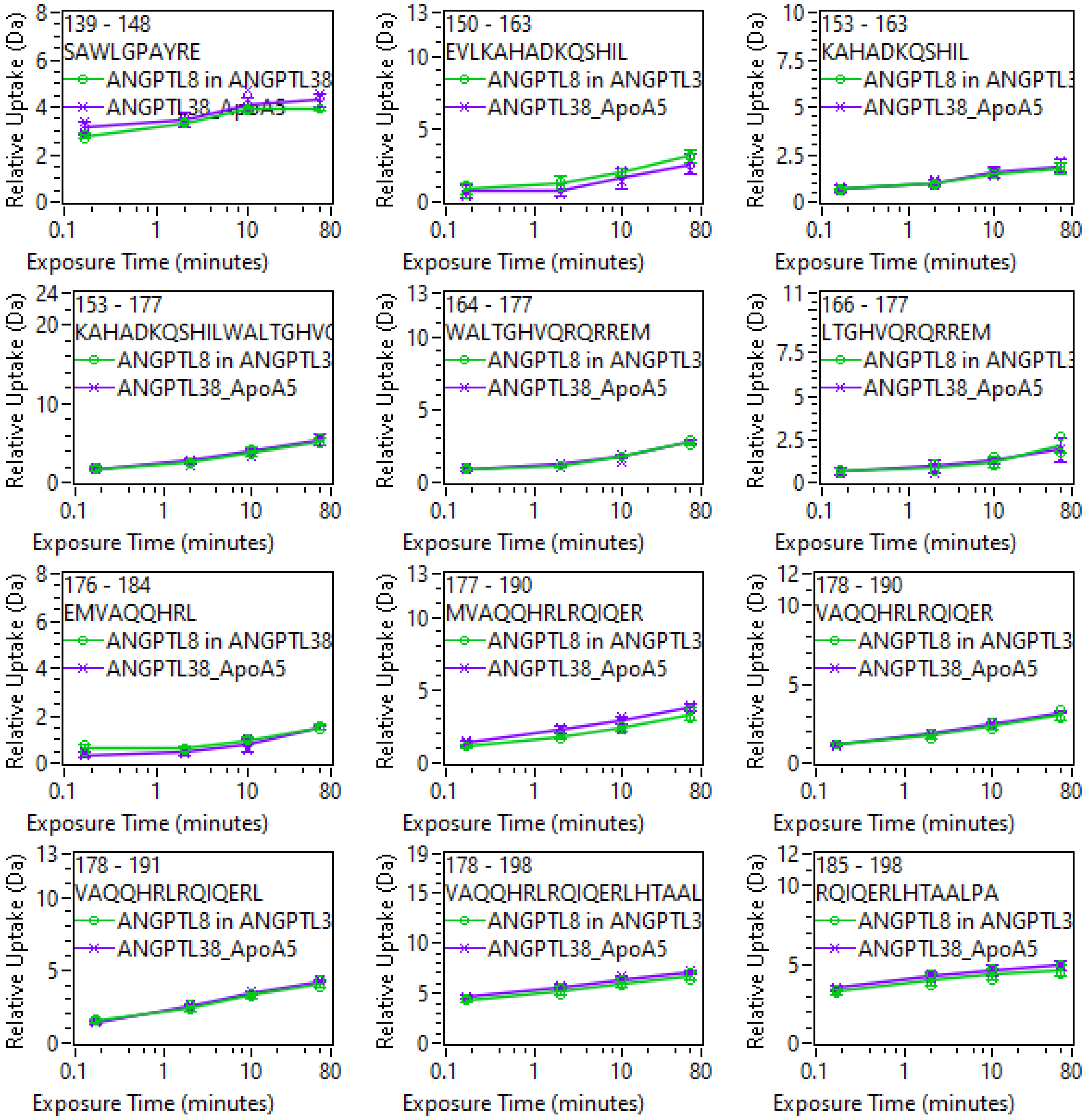

Figure S4. HDXMS comparing ANGPTL3/8 bound to anti-ANGPTL3/8 antibody to unbound ANGPTL3/8

Figure S4A. Sequence coverage for ANGPTL3 (deuterated peptides followed in the experiment)

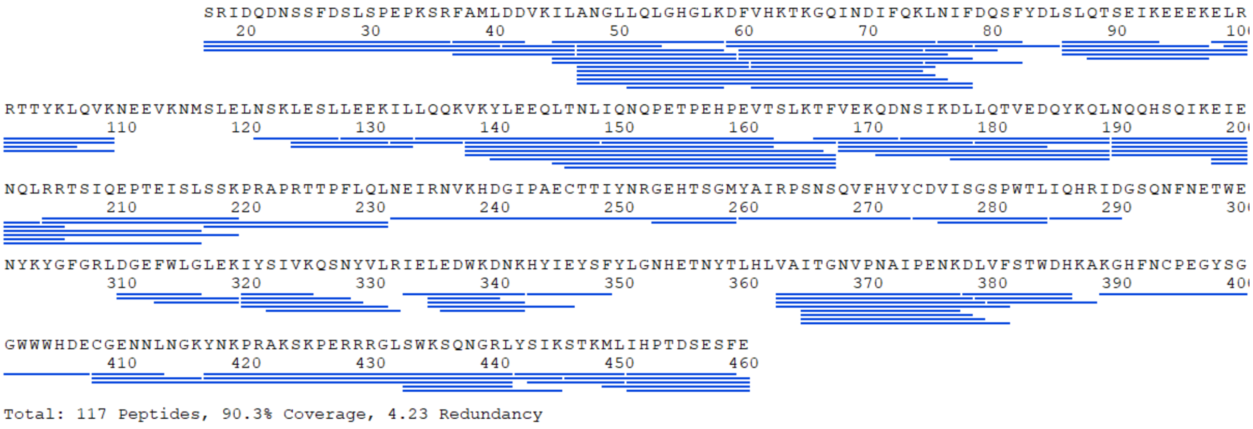

Figure S4B. Sequence coverage for ANGPTL8 (deuterated peptides followed in the experiment)

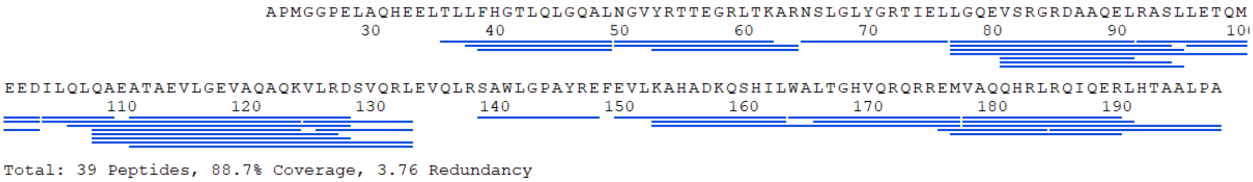

Figure S4C. Difference plot comparing the changes in deuterium uptake of ANGPTL3 in ANGPTL3/8 complex bound to anti-ANGPTL3/8 antibody relative to ANGPTL3 in unbound ANGPTL3/8 complex

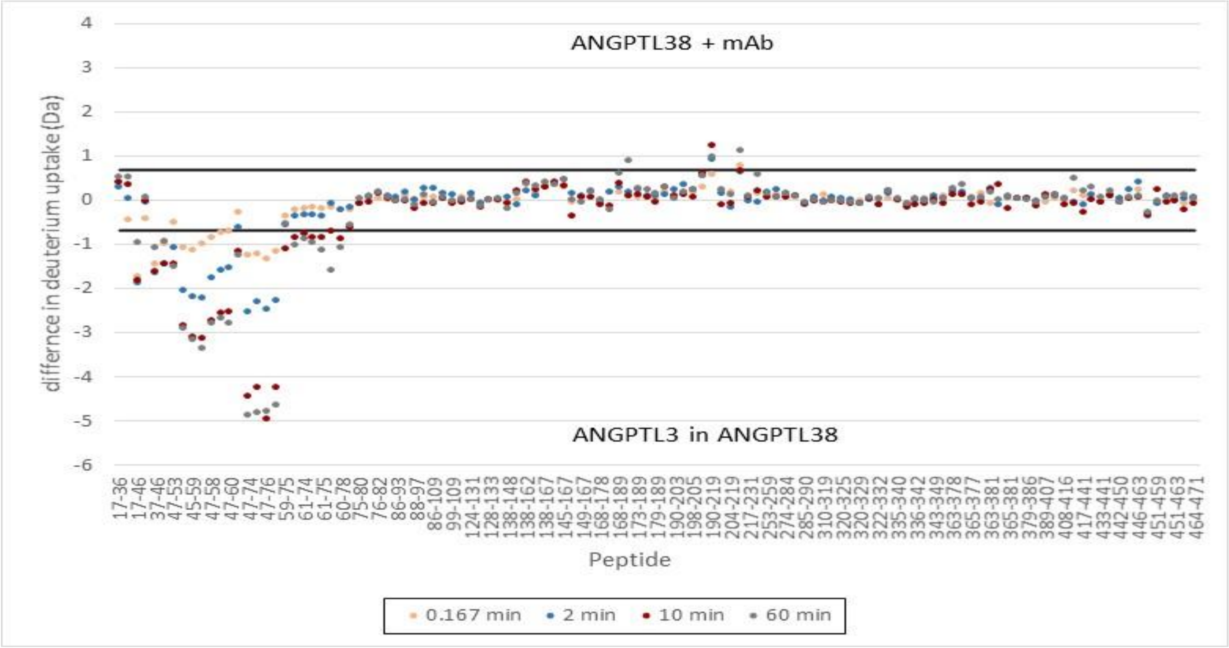

Figure S4D. Difference plot comparing the changes in deuterium uptake of ANGPTL8 in ANGPTL3/8 complex bound to anti-ANGPTL3/8 antibody relative to ANGPTL8 in unbound ANGPTL3/8 complex

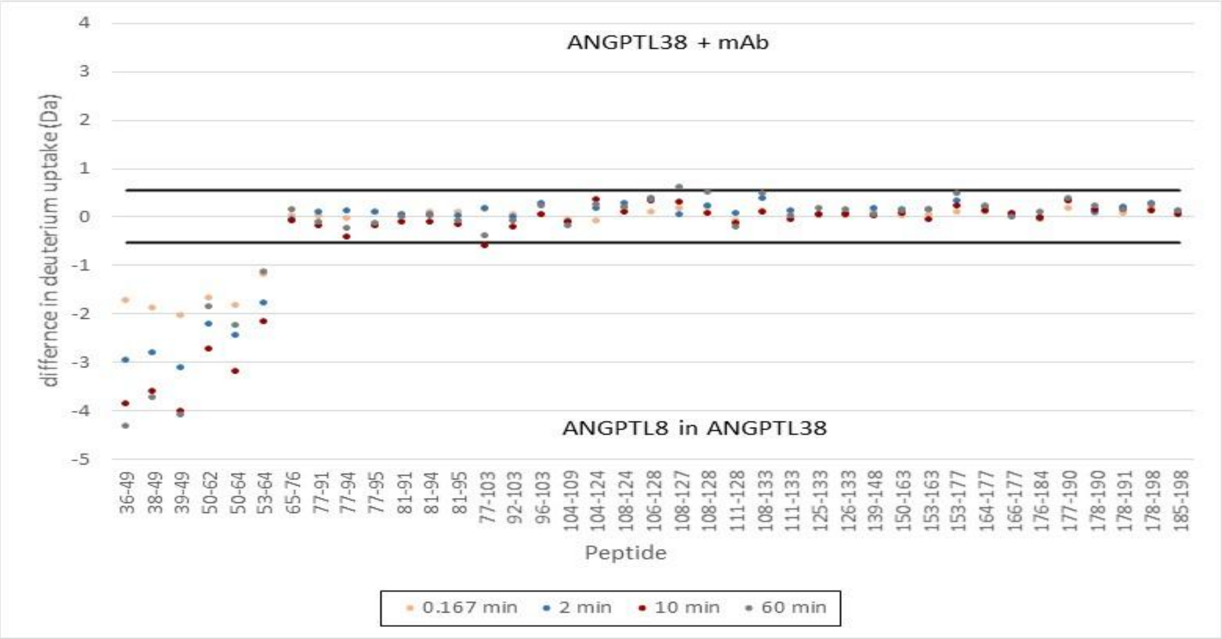

Figure S4E. Heat map of the relative deuterium uptake of ANGPTL3 in ANGPTL3/8 complex bound to anti-ANGPTL3/8 antibody relative to ANGPTL3 in unbound ANGPTL3/8 complex

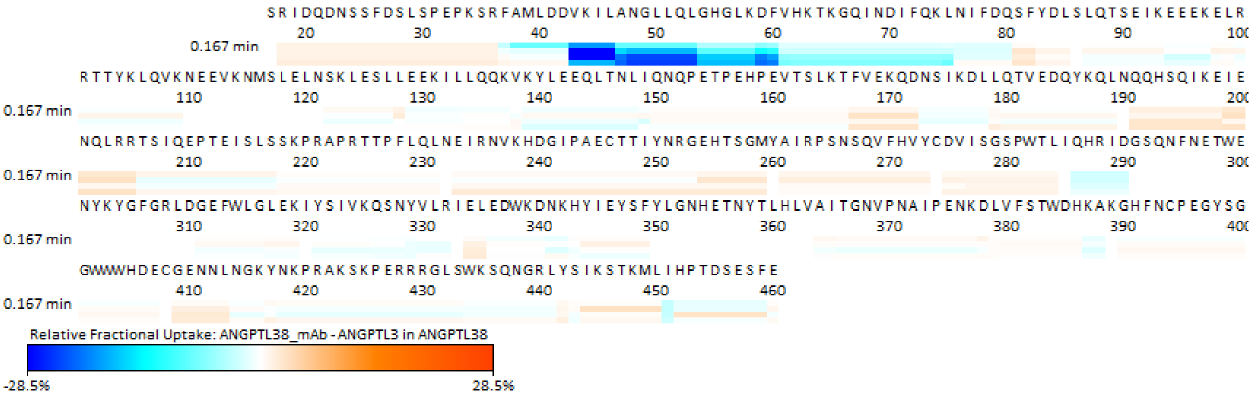

Figure S4F. Heat map of the relative deuterium uptake of ANGPTL3 in ANGPTL3/8 complex bound to anti-ANGPTL3/8 antibody relative to ANGPTL3 in unbound ANGPTL3/8 complex

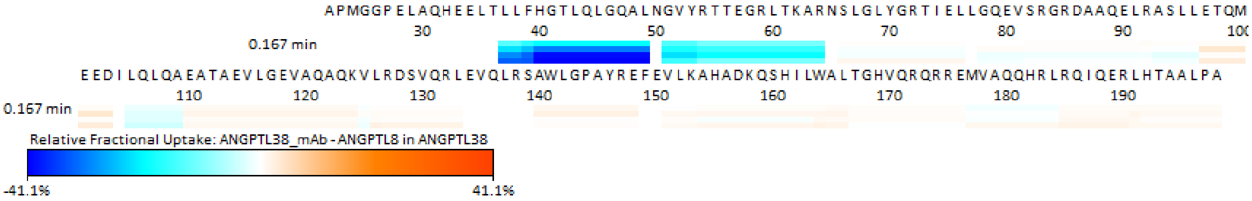

Figure S4G. ANGPTL3 uptake plots for ANGPTL3/8 bound to anti-ANGPTL3/8 antibody compared to unbound ANGPTL3/8. Standard deviation in uptake difference is 0.23 Da (Experiment performed in triplicate with error bars shown).

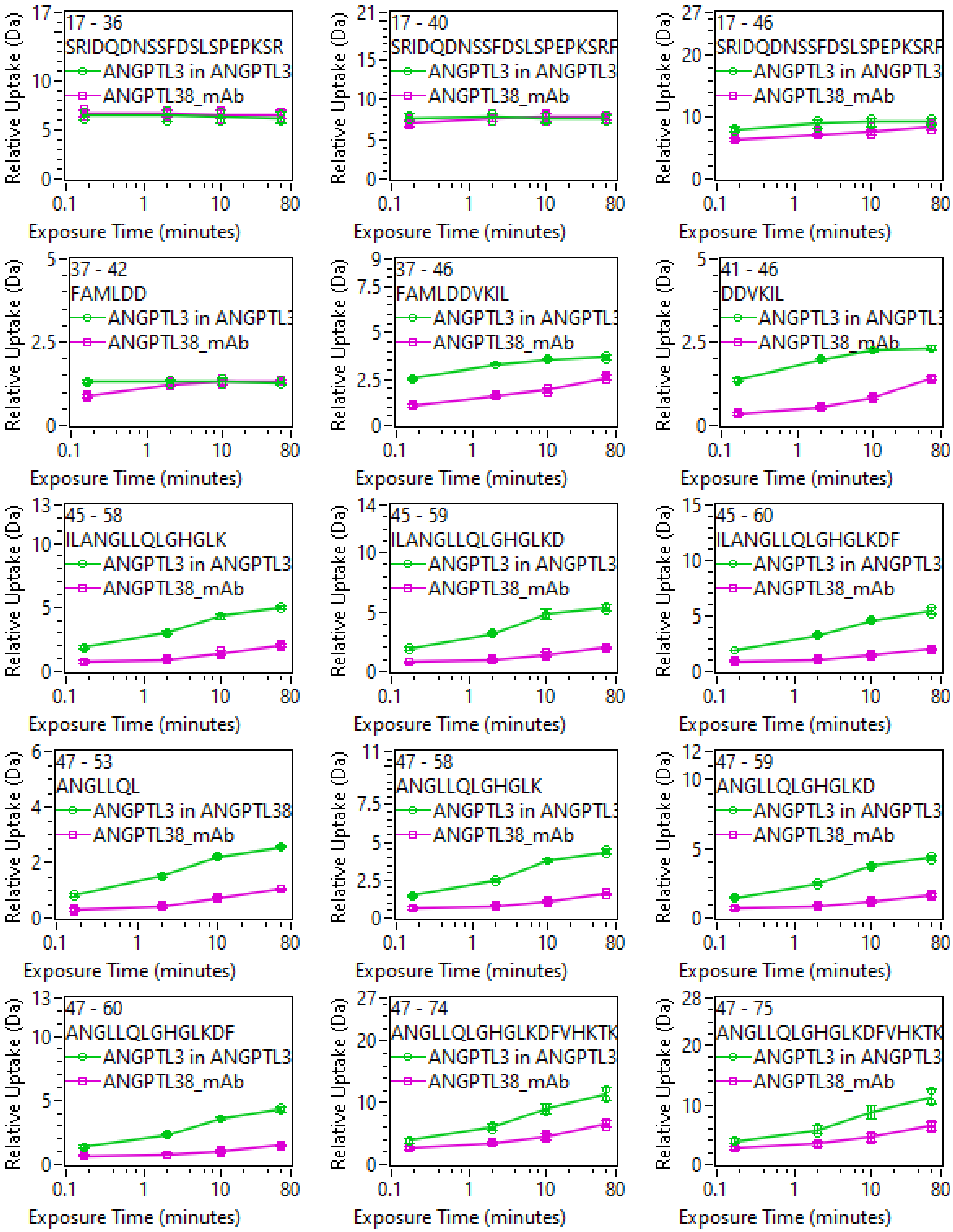

Figure S4G continued

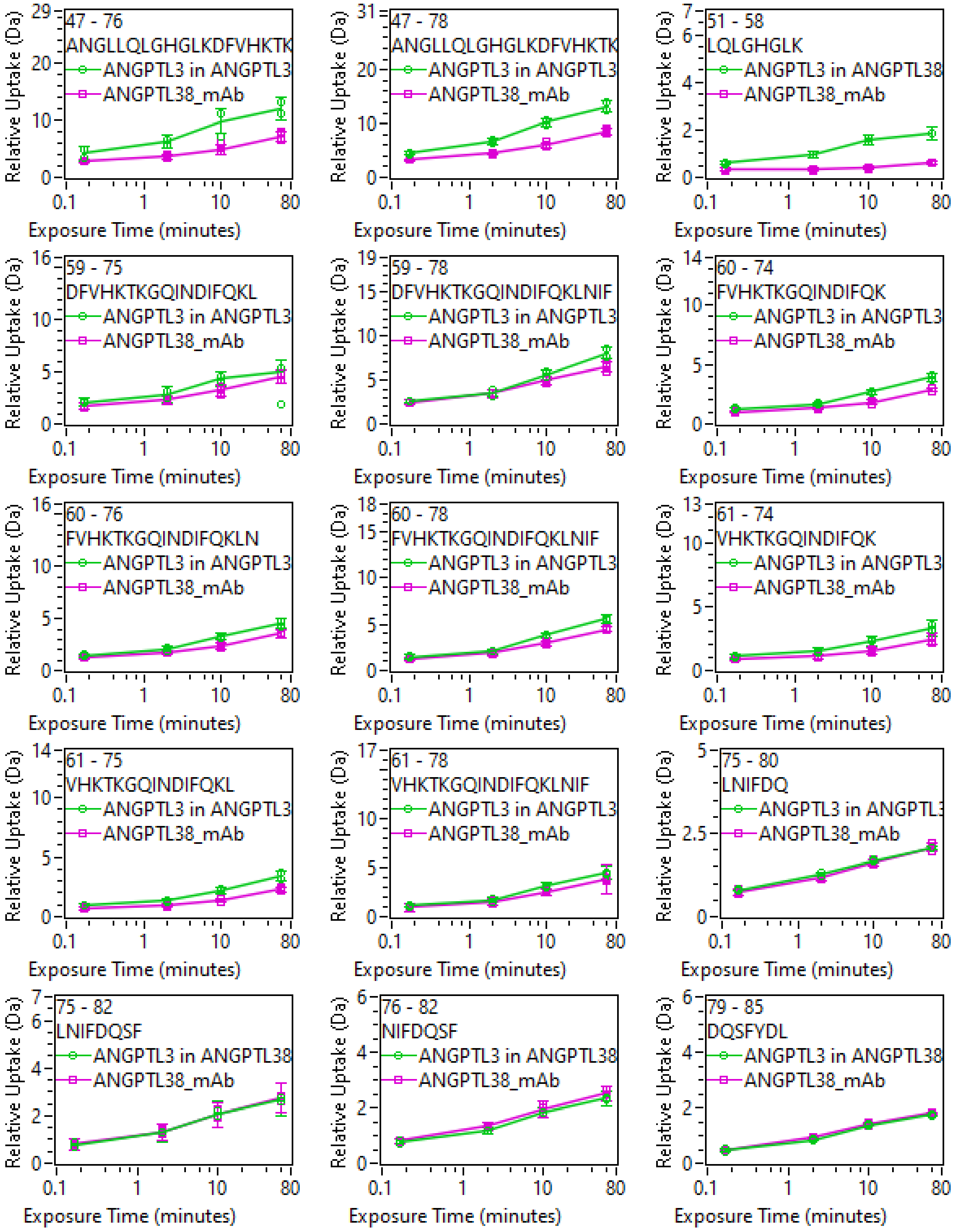

Figure S4G continued

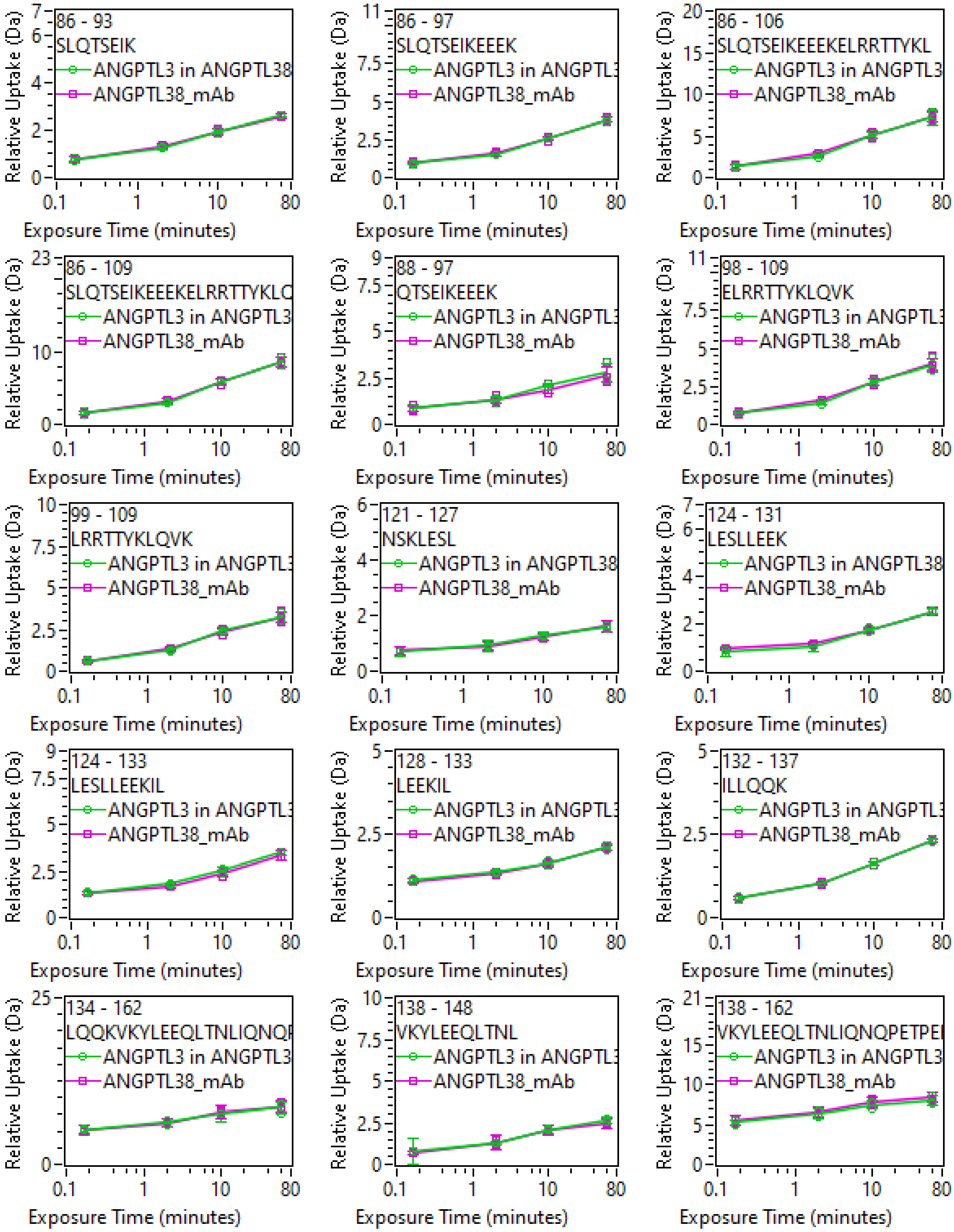

Figure S4G continued

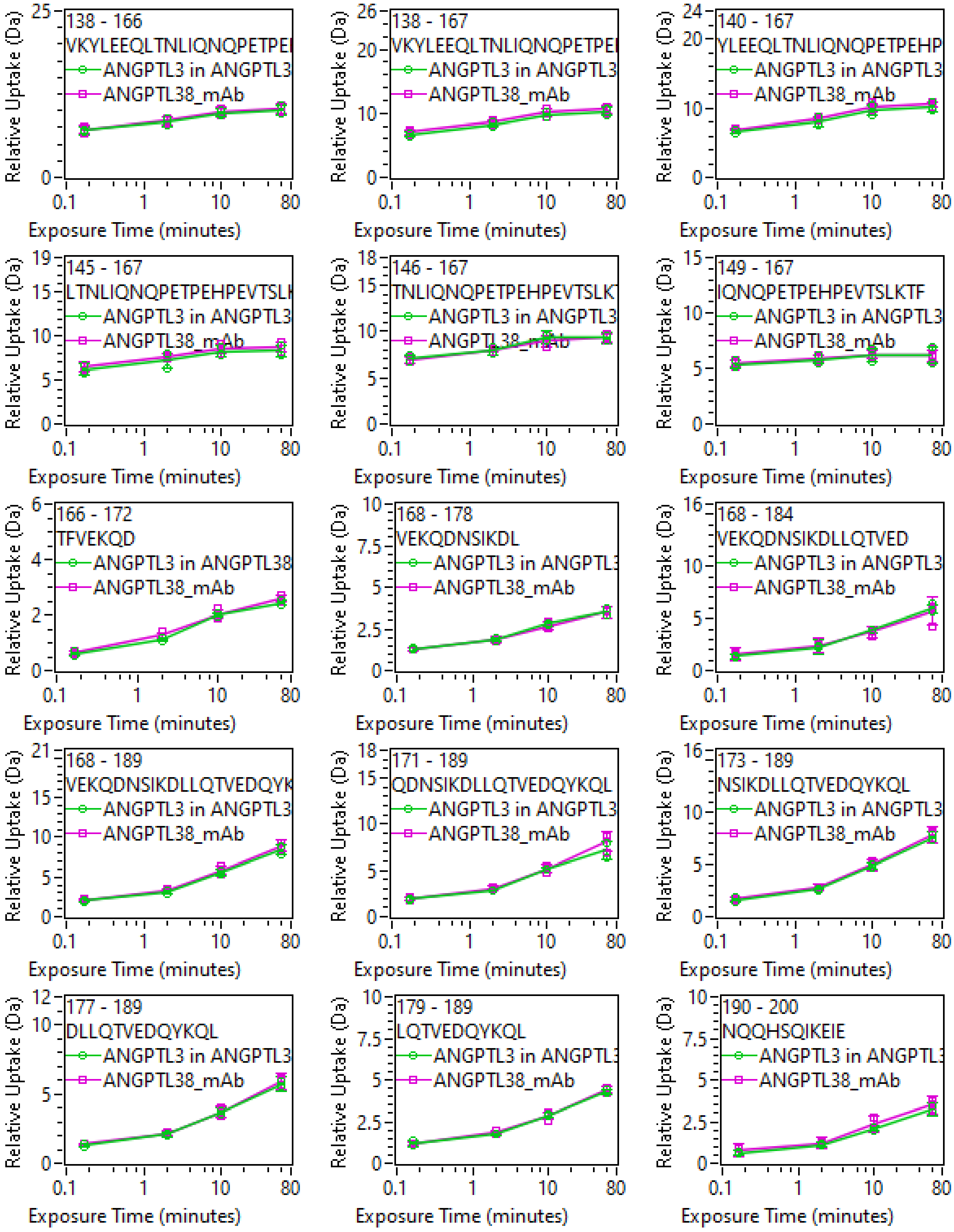

Figure S4G continued

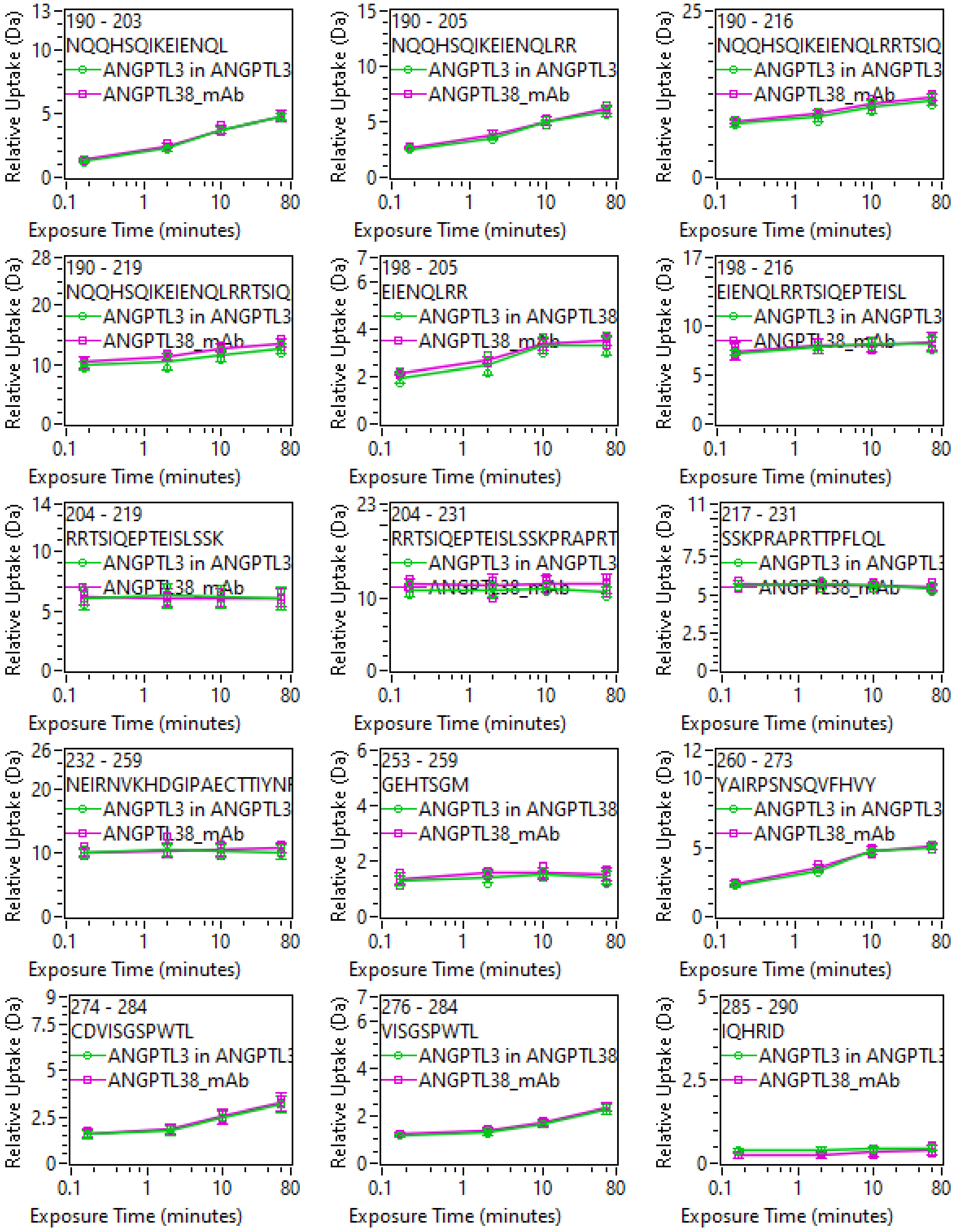

Figure S4G continued

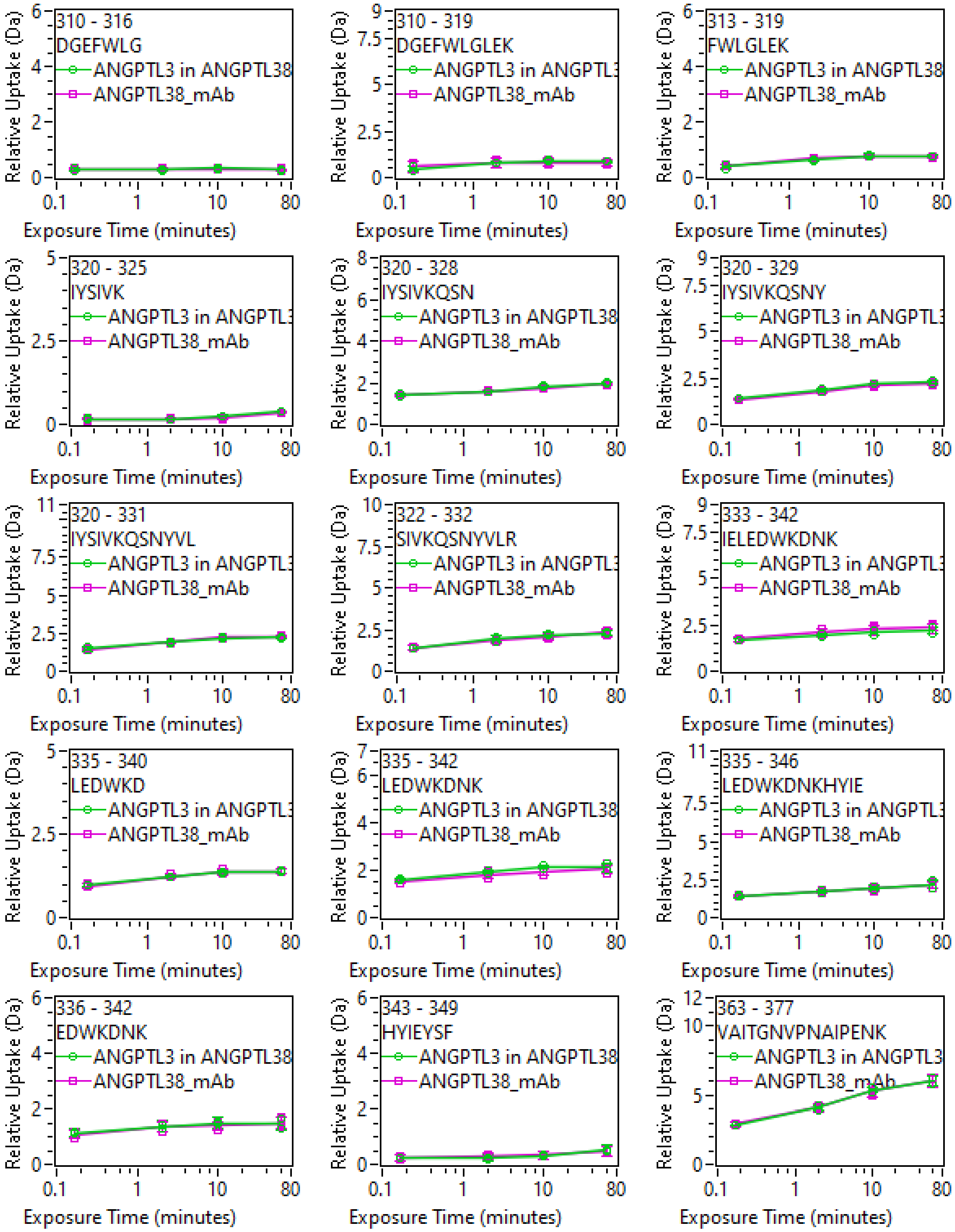

Figure S4G continued

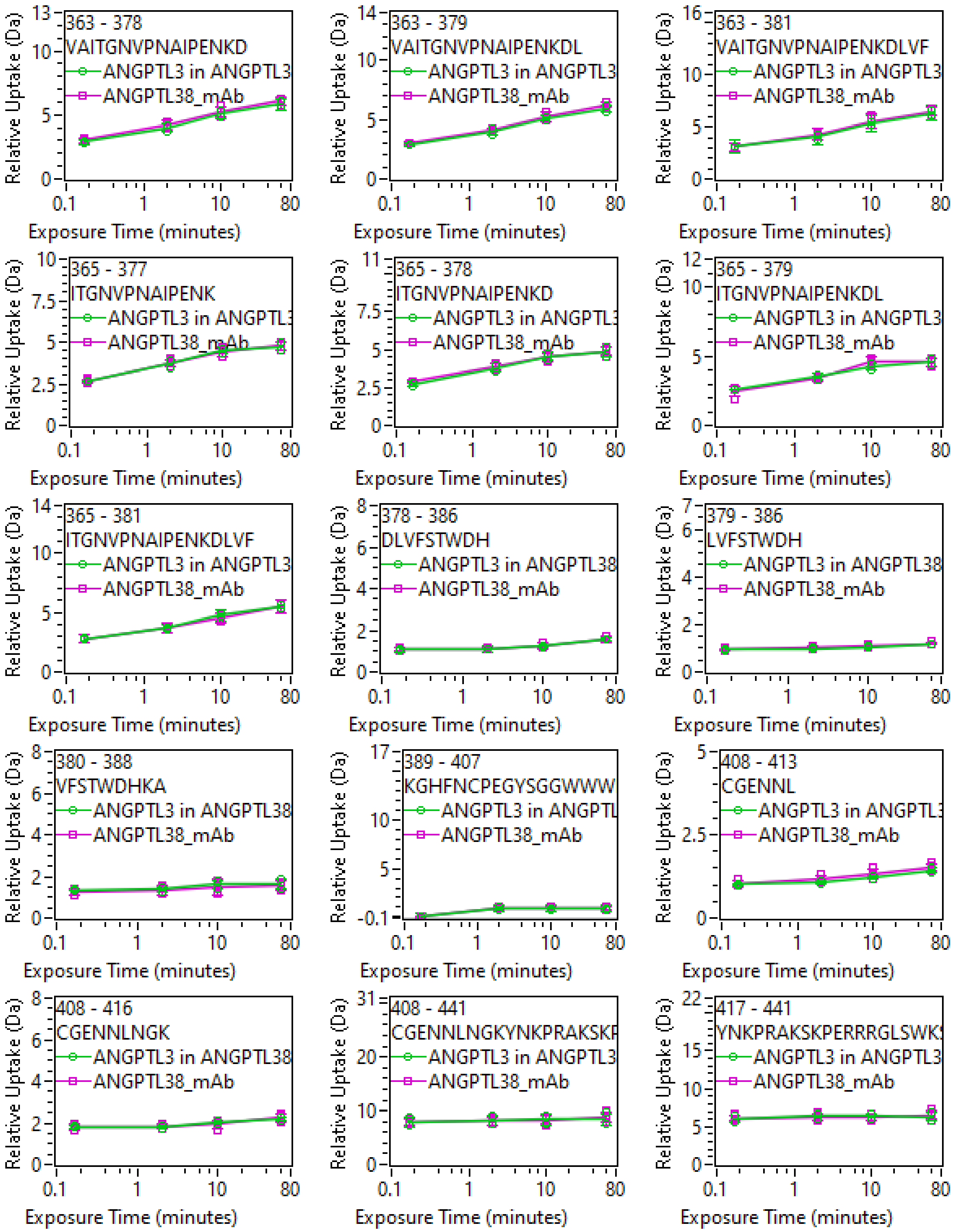

Figure S4G continued

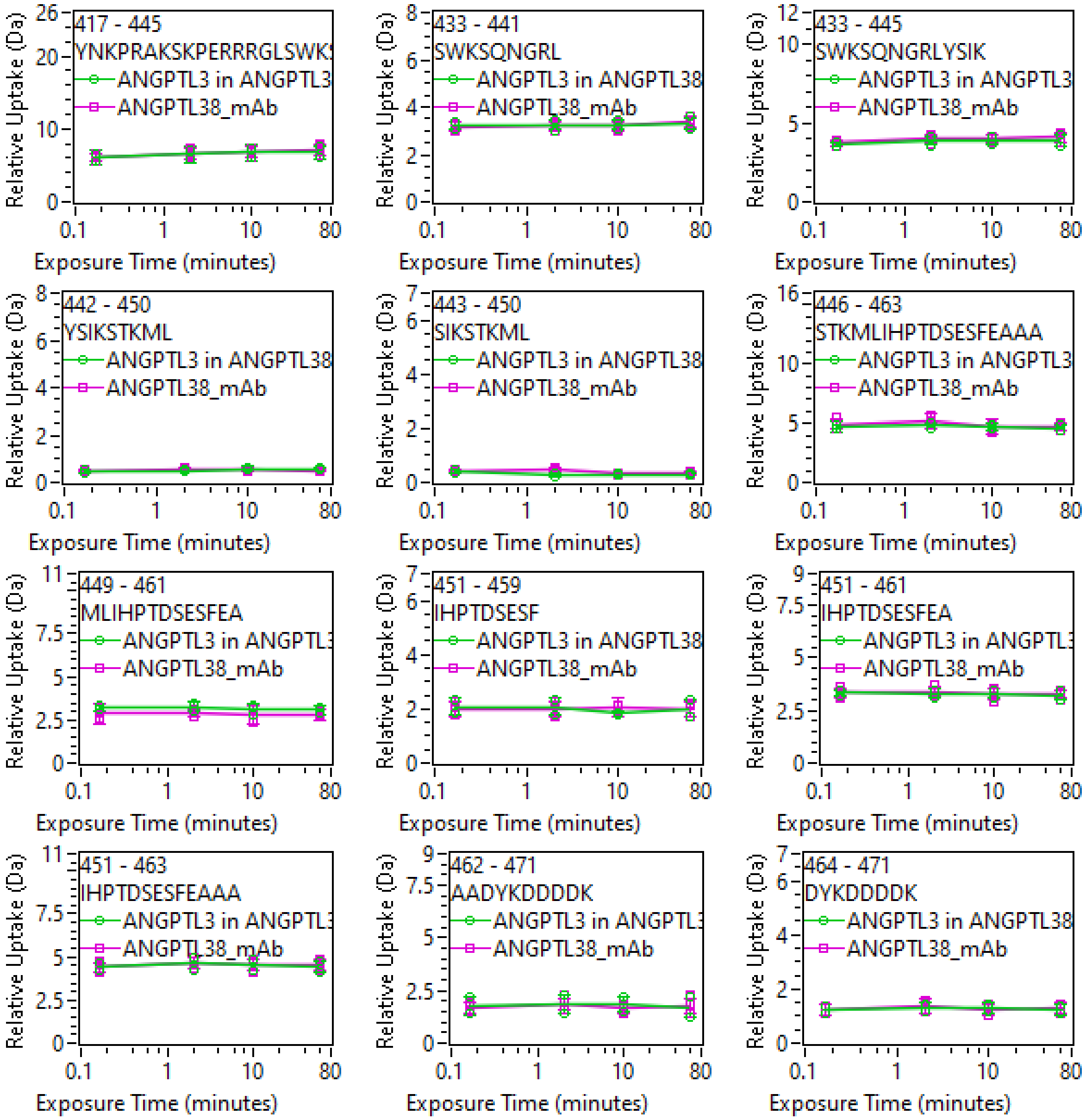

Figure S4H. ANGPTL8 uptake plots for ANGPTL3/8 bound to anti-ANGPTL3/8 antibody compared to unbound ANGPTL3/8. Standard deviation in uptake difference is 0.18 Da (Experiment performed in triplicate with error bars shown).

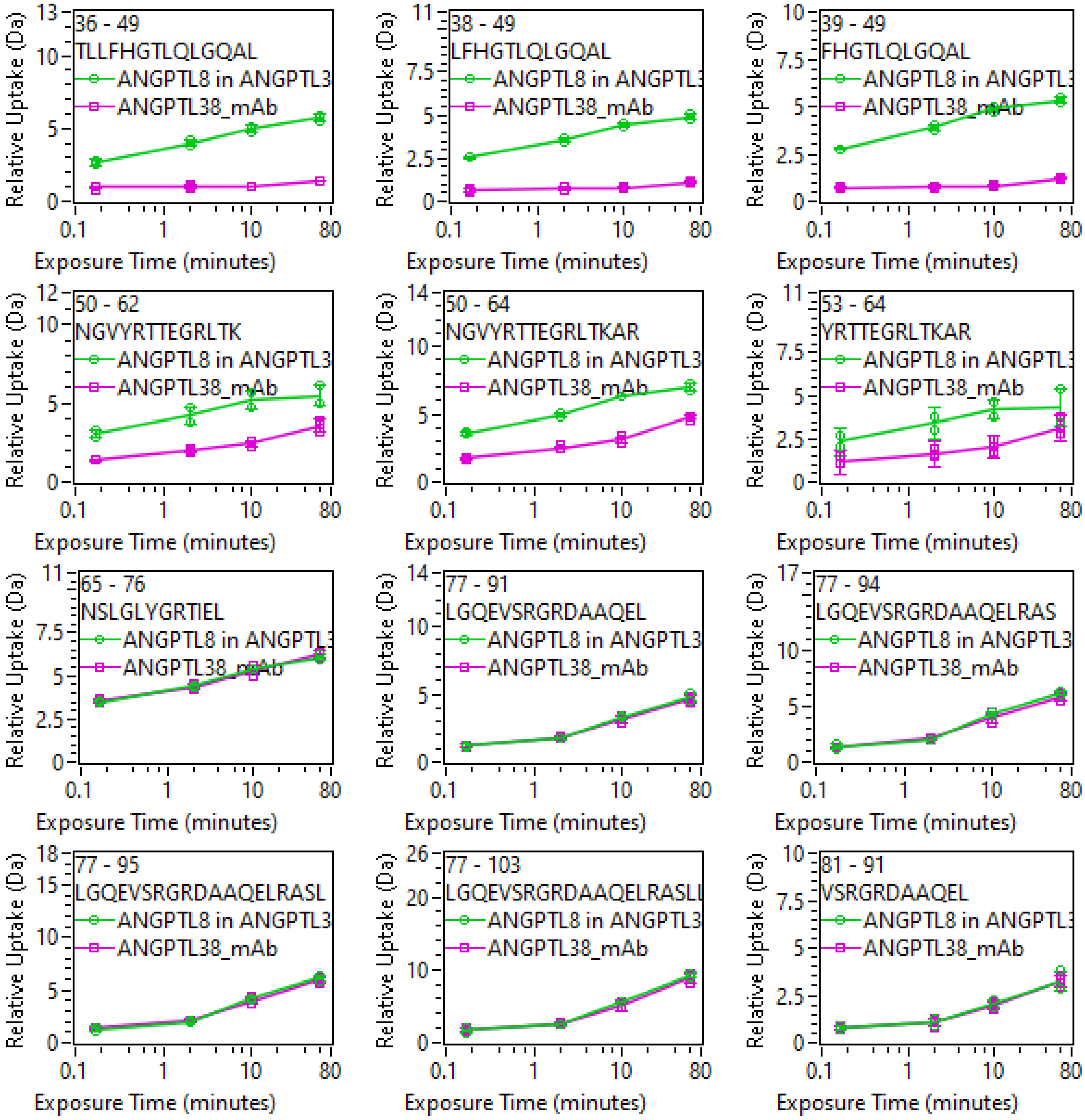

Figure S4H continued

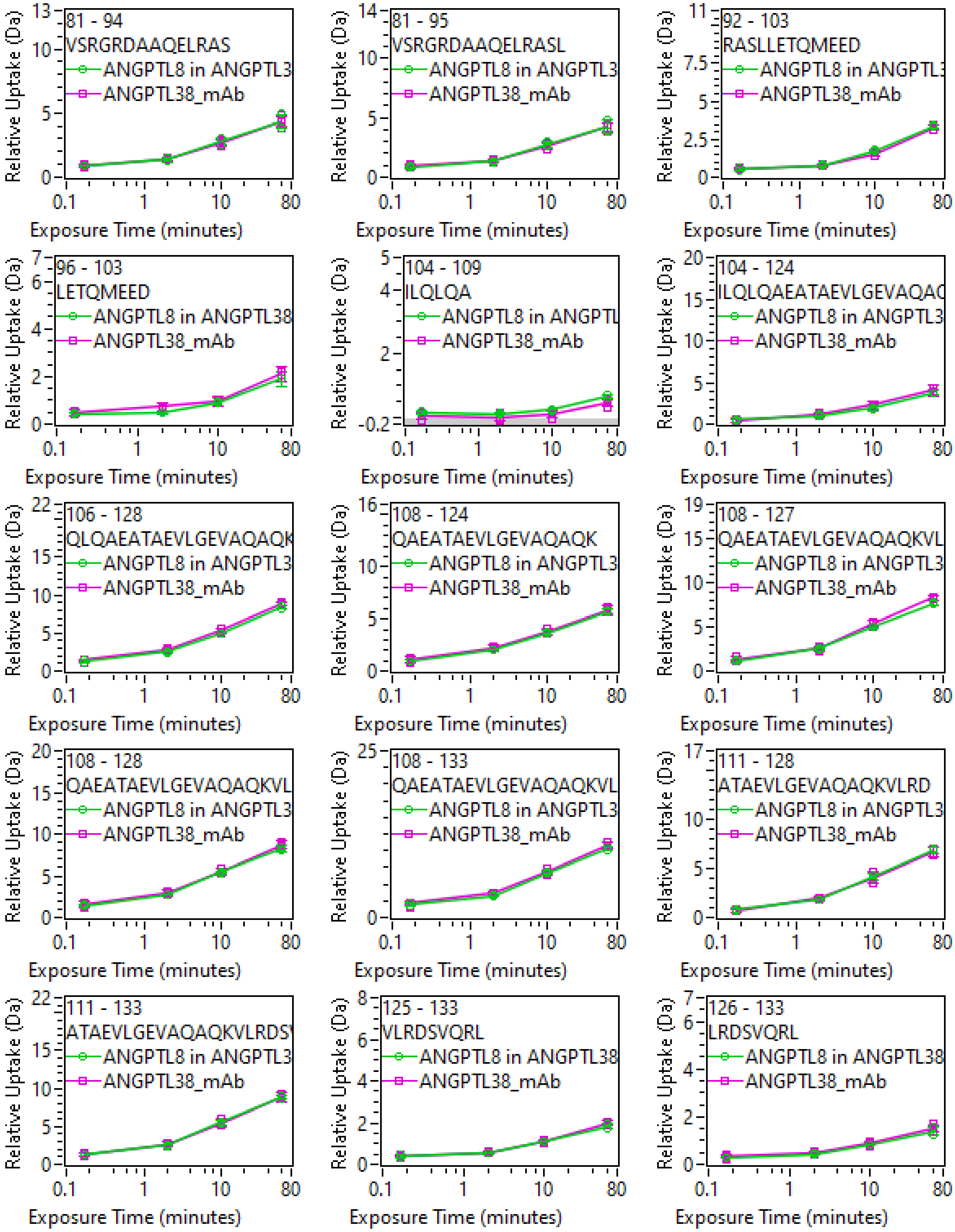

Figure S4H continued

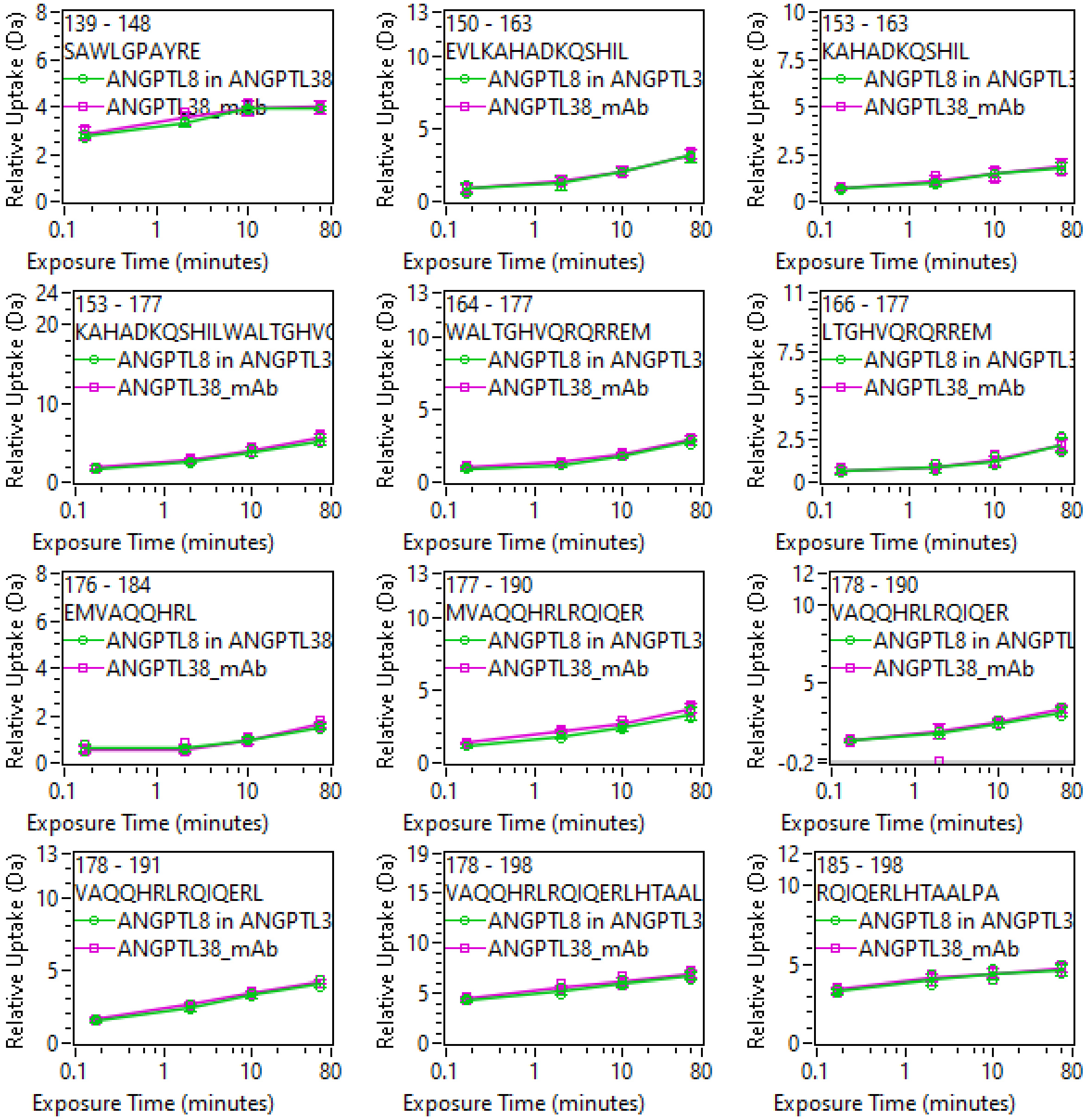

Figure S5: Volcano plot analyses at the 10-min exchange time for ANGPTL3 and ANGPTL8 peptides.

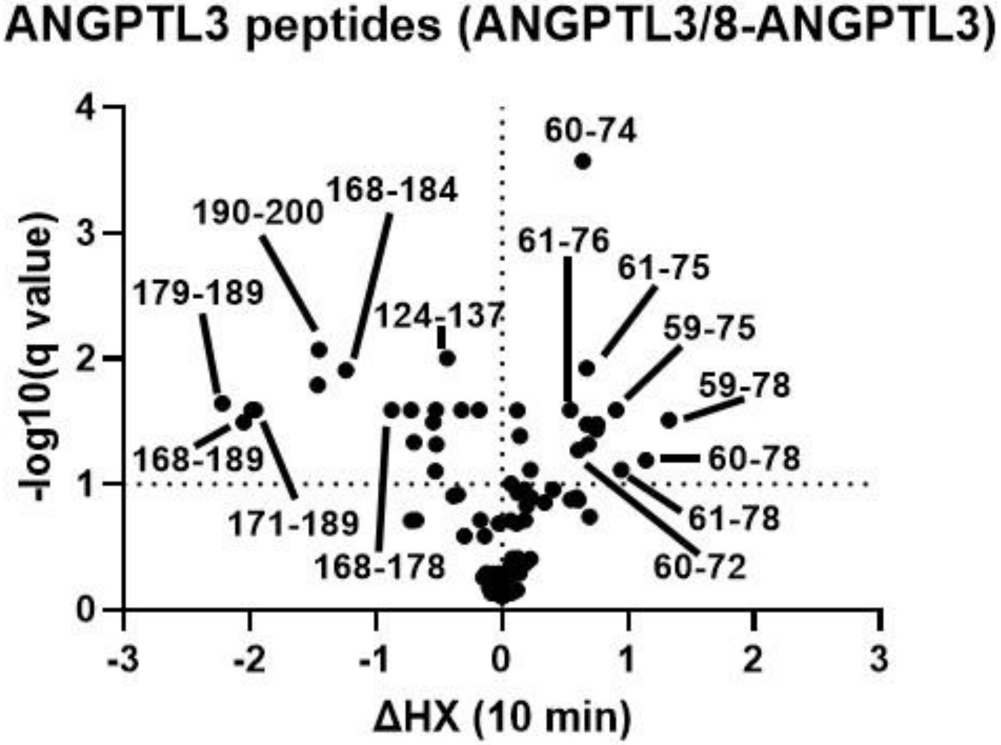

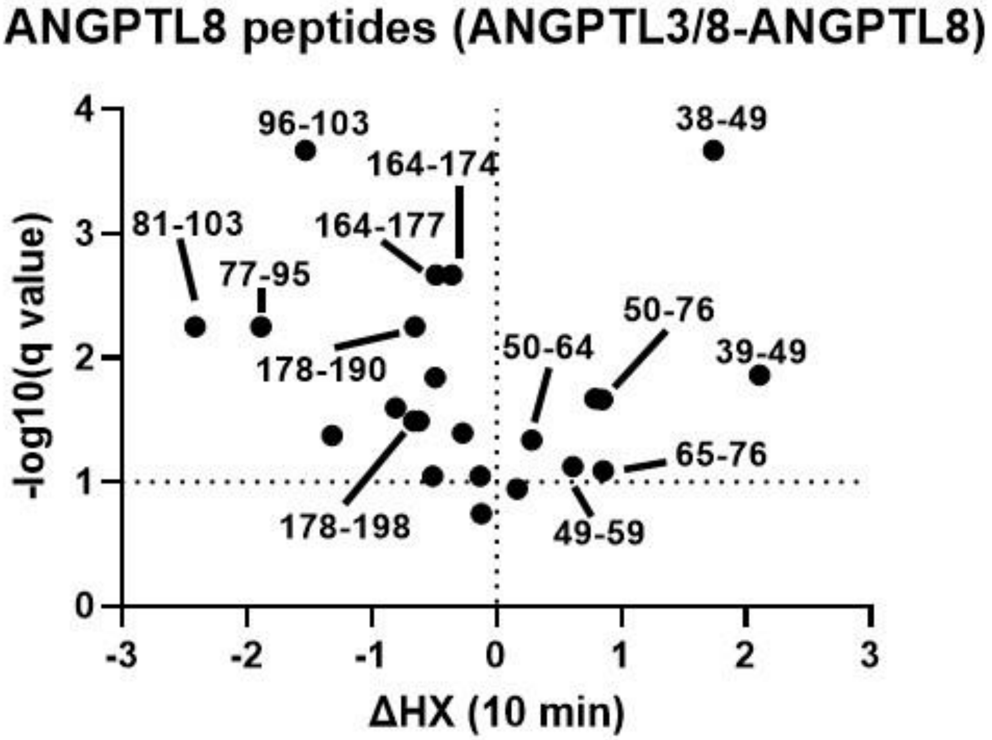

Table S1: Differential HX, combined uncertainty, and significance testing for individual HX times for ANGPTL3 peptides in the ANGPTL3/8 complex compared to ANGPTL3 alone

Protein	Peptide	Exposure	U=ku k=2	Δ (HX)t	ABS(Δ (HX)t)	True = significant difference, reject Null (no difference)
Angptl3	17-40	10 s	0.302	0.261	0.261	FALSE
Angptl3	17-40	2 min	0.394	-0.332	0.332	FALSE
Angptl3	17-40	10 min	0.400	-0.102	0.102	FALSE
Angptl3	17-40	60 min	0.195	-0.138	0.138	FALSE
Angptl3	37-42	10 s	0.158	0.049	0.049	FALSE
Angptl3	37-42	2 min	0.107	-0.084	0.084	FALSE
Angptl3	37-42	10 min	0.133	0.110	0.110	FALSE
Angptl3	37-42	60 min	0.170	0.125	0.125	FALSE
Angptl3	37-46	10 s	0.094	0.590	0.590	TRUE
Angptl3	37-46	2 min	0.136	0.454	0.454	TRUE
Angptl3	37-46	10 min	0.148	-0.062	0.062	FALSE
Angptl3	37-46	60 min	0.164	-0.006	0.006	FALSE
Angptl3	41-46	10 s	0.067	0.511	0.511	TRUE
Angptl3	41-46	2 min	0.081	0.411	0.411	TRUE
Angptl3	41-46	10 min	0.092	0.010	0.010	FALSE
Angptl3	41-46	60 min	0.068	-0.090	0.090	TRUE
Angptl3	41-59	10 s	0.135	1.106	1.106	TRUE
Angptl3	41-59	2 min	0.159	1.068	1.068	TRUE
Angptl3	41-59	10 min	0.481	1.070	1.070	TRUE
Angptl3	41-59	60 min	0.198	-0.754	0.754	TRUE
Angptl3	45-53	10 s	0.311	0.669	0.669	TRUE
Angptl3	45-53	2 min	0.101	0.588	0.588	TRUE
Angptl3	45-53	10 min	0.174	0.769	0.769	TRUE
Angptl3	45-53	60 min	0.104	0.167	0.167	TRUE
Angptl3	45-60	10 s	0.289	0.496	0.496	TRUE
Angptl3	45-60	2 min	0.199	0.364	0.364	TRUE
Angptl3	45-60	10 min	0.370	0.132	0.132	FALSE
Angptl3	45-60	60 min	0.352	-0.327	0.327	FALSE
Angptl3	45-74	10 s	0.378	1.379	1.379	TRUE
Angptl3	45-74	2 min	0.534	1.910	1.910	TRUE
Angptl3	45-74	10 min	0.652	2.464	2.464	TRUE
Angptl3	45-74	60 min	0.742	2.164	2.164	TRUE
Angptl3	47-53	10 s	0.082	0.133	0.133	TRUE
Angptl3	47-53	2 min	0.117	0.264	0.264	TRUE
Angptl3	47-53	10 min	0.157	0.277	0.277	TRUE
Angptl3	47-53	60 min	0.162	-0.269	0.269	TRUE
Angptl3	47-58	10 s	0.078	0.439	0.439	TRUE
Angptl3	47-58	2 min	0.136	0.566	0.566	TRUE
Angptl3	47-58	10 min	0.203	0.597	0.597	TRUE
Angptl3	47-58	60 min	0.141	-0.238	0.238	TRUE
Angptl3	47-59	10 s	0.106	0.422	0.422	TRUE
Angptl3	47-59	2 min	0.147	0.497	0.497	TRUE
Angptl3	47-59	10 min	0.189	0.585	0.585	TRUE
Angptl3	47-59	60 min	0.178	-0.236	0.236	TRUE
Angptl3	47-60	10 s	0.141	0.386	0.386	TRUE
Angptl3	47-60	2 min	0.153	0.343	0.343	TRUE
Angptl3	47-60	10 min	0.216	0.354	0.354	TRUE
Angptl3	47-60	60 min	0.227	-0.383	0.383	TRUE
Angptl3	47-74	10 s	0.278	1.165	1.165	TRUE
Angptl3	47-74	2 min	0.579	1.389	1.389	TRUE
Angptl3	47-74	10 min	0.490	2.143	2.143	TRUE
Angptl3	47-74	60 min	0.370	1.862	1.862	TRUE
Angptl3	47-75	10 s	0.231	1.154	1.154	TRUE
Angptl3	47-75	2 min	1.406	1.434	1.434	TRUE
Angptl3	47-75	10 min	1.337	1.915	1.915	TRUE

Protein	Peptide	Exposure	U=ku k=2	Δ (HX)t	ABS(Δ (HX)t)	True = significant difference, reject Null (no difference)
Angptl3	51-58	10 s	0.070	0.031	0.031	FALSE
Angptl3	51-58	2 min	0.098	0.232	0.232	TRUE
Angptl3	51-58	10 min	0.145	0.146	0.146	TRUE
Angptl3	51-58	60 min	0.178	-0.380	0.380	TRUE
Angptl3	59-75	10 s	0.147	0.816	0.816	TRUE
Angptl3	59-75	2 min	0.256	1.068	1.068	TRUE
Angptl3	59-75	10 min	0.170	1.800	1.800	TRUE
Angptl3	59-75	60 min	0.255	2.269	2.269	TRUE
Angptl3	59-78	10 s	0.126	1.163	1.163	TRUE
Angptl3	59-78	2 min	0.257	1.660	1.660	TRUE
Angptl3	59-78	10 min	0.259	2.593	2.593	TRUE
Angptl3	59-78	60 min	0.284	2.940	2.940	TRUE
Angptl3	60-72	10 s	0.235	0.404	0.404	TRUE
Angptl3	60-72	2 min	0.358	0.705	0.705	TRUE
Angptl3	60-72	10 min	0.341	1.183	1.183	TRUE
Angptl3	60-72	60 min	0.363	1.153	1.153	TRUE
Angptl3	60-74	10 s	0.090	0.493	0.493	TRUE
Angptl3	60-74	2 min	0.152	0.710	0.710	TRUE
Angptl3	60-74	10 min	0.092	1.275	1.275	TRUE
Angptl3	60-74	60 min	0.169	1.605	1.605	TRUE
Angptl3	60-78	10 s	0.057	0.497	0.497	TRUE
Angptl3	60-78	2 min	0.170	1.212	1.212	TRUE
Angptl3	60-78	10 min	0.406	2.200	2.200	TRUE
Angptl3	60-78	60 min	0.251	2.580	2.580	TRUE
Angptl3	61-75	10 s	0.152	0.462	0.462	TRUE
Angptl3	61-75	2 min	0.129	0.804	0.804	TRUE
Angptl3	61-75	10 min	0.082	1.377	1.377	TRUE
Angptl3	61-75	60 min	0.117	1.686	1.686	TRUE
Angptl3	61-76	10 s	0.185	0.563	0.563	TRUE
Angptl3	61-76	2 min	0.257	0.712	0.712	TRUE
Angptl3	61-76	10 min	0.230	1.054	1.054	TRUE
Angptl3	61-76	60 min	0.274	1.799	1.799	TRUE
Angptl3	61-78	10 s	0.351	0.765	0.765	TRUE
Angptl3	61-78	2 min	0.688	1.226	1.226	TRUE
Angptl3	61-78	10 min	0.537	1.883	1.883	TRUE
Angptl3	61-78	60 min	0.296	2.300	2.300	TRUE
Angptl3	75-82	10 s	0.235	0.326	0.326	TRUE
Angptl3	75-82	2 min	0.279	0.284	0.284	TRUE
Angptl3	75-82	10 min	0.211	0.603	0.603	TRUE
Angptl3	75-82	60 min	0.215	0.373	0.373	TRUE
Angptl3	76-82	10 s	0.062	0.258	0.258	TRUE
Angptl3	76-82	2 min	0.078	0.344	0.344	TRUE
Angptl3	76-82	10 min	0.107	0.351	0.351	TRUE
Angptl3	76-82	60 min	0.133	0.369	0.369	TRUE
Angptl3	79-85	10 s	0.053	0.160	0.160	TRUE
Angptl3	79-85	2 min	0.088	0.230	0.230	TRUE
Angptl3	79-85	10 min	0.061	0.186	0.186	TRUE
Angptl3	79-85	60 min	0.053	0.280	0.280	TRUE
Angptl3	86-93	10 s	0.089	0.335	0.335	TRUE
Angptl3	86-93	2 min	0.044	0.446	0.446	TRUE
Angptl3	86-93	10 min	0.104	0.032	0.032	FALSE
Angptl3	86-93	60 min	0.061	-0.572	0.572	TRUE
Angptl3	86-95	10 s	0.236	0.439	0.439	TRUE
Angptl3	86-95	2 min	0.159	0.435	0.435	TRUE
Angptl3	86-95	10 min	0.127	-0.026	0.026	FALSE
Angptl3	86-95	60 min	0.154	-0.980	0.980	TRUE
Angptl3	86-97	10 s	0.163	0.396	0.396	TRUE
Angptl3	86-97	2 min	0.153	0.404	0.404	TRUE
Angptl3	86-97	10 min	0.161	-0.091	0.091	FALSE
Angptl3	86-97	60 min	0.067	-0.945	0.945	TRUE

Protein	Peptide	Exposure	U=ku k=2	Δ (HX)t	ABS(Δ (HX)t)	True = significant difference, reject Null (no difference)
Angptl3	86-98	2 min	0.279	0.388	0.388	TRUE
Angptl3	86-98	10 min	0.267	-0.435	0.435	TRUE
Angptl3	86-98	60 min	0.243	-1.435	1.435	TRUE
Angptl3	86-102	10 s	0.222	0.158	0.158	FALSE
Angptl3	86-102	2 min	0.383	-0.127	0.127	FALSE
Angptl3	86-102	10 min	0.230	-0.378	0.378	TRUE
Angptl3	86-102	60 min	0.321	-1.576	1.576	TRUE
Angptl3	86-106	10 s	0.114	-0.034	0.034	FALSE
Angptl3	86-106	2 min	0.299	-0.353	0.353	TRUE
Angptl3	86-106	10 min	0.225	-0.688	0.688	TRUE
Angptl3	86-106	60 min	0.181	-1.892	1.892	TRUE
Angptl3	86-109	10 s	0.639	-0.166	0.166	FALSE
Angptl3	86-109	2 min	0.687	-1.078	1.078	TRUE
Angptl3	86-109	10 min	0.365	-1.528	1.528	TRUE
Angptl3	86-109	60 min	0.734	-2.658	2.658	TRUE
Angptl3	94-106	10 s	0.161	-0.237	0.237	TRUE
Angptl3	94-106	2 min	0.286	-0.805	0.805	TRUE
Angptl3	94-106	10 min	0.424	-0.618	0.618	TRUE
Angptl3	94-106	60 min	0.600	-0.926	0.926	TRUE
Angptl3	94-109	10 s	0.249	-0.560	0.560	TRUE
Angptl3	94-109	2 min	0.354	-1.410	1.410	TRUE
Angptl3	94-109	10 min	0.226	-1.421	1.421	TRUE
Angptl3	94-109	60 min	0.450	-1.474	1.474	TRUE
Angptl3	96-109	10 s	0.219	-0.348	0.348	TRUE
Angptl3	96-109	2 min	0.467	-1.074	1.074	TRUE
Angptl3	96-109	10 min	0.359	-1.074	1.074	TRUE
Angptl3	96-109	60 min	0.597	-1.250	1.250	TRUE
Angptl3	98-109	10 s	0.145	-0.319	0.319	TRUE
Angptl3	98-109	2 min	0.293	-1.136	1.136	TRUE
Angptl3	98-109	10 min	0.258	-1.057	1.057	TRUE
Angptl3	98-109	60 min	0.449	-1.130	1.130	TRUE
Angptl3	99-109	10 s	0.147	-0.357	0.357	TRUE
Angptl3	99-109	2 min	0.276	-1.114	1.114	TRUE
Angptl3	99-109	10 min	0.184	-1.064	1.064	TRUE
Angptl3	99-109	60 min	0.279	-0.950	0.950	TRUE
Angptl3	124-131	10 s	0.124	-0.459	0.459	TRUE
Angptl3	124-131	2 min	0.097	-0.404	0.404	TRUE
Angptl3	124-131	10 min	0.064	-0.315	0.315	TRUE
Angptl3	124-131	60 min	0.072	-0.223	0.223	TRUE
Angptl3	124-133	10 s	0.090	0.338	0.338	TRUE
Angptl3	124-133	2 min	0.079	-0.149	0.149	TRUE
Angptl3	124-133	10 min	0.059	-0.544	0.544	TRUE
Angptl3	124-133	60 min	0.051	-0.257	0.257	TRUE
Angptl3	124-137	10 s	0.203	0.534	0.534	TRUE
Angptl3	124-137	2 min	0.211	-0.073	0.073	FALSE
Angptl3	124-137	10 min	0.246	-0.948	0.948	TRUE
Angptl3	124-137	60 min	0.386	-0.718	0.718	TRUE
Angptl3	132-137	10 s	0.054	0.226	0.226	TRUE
Angptl3	132-137	2 min	0.049	0.018	0.018	FALSE
Angptl3	132-137	10 min	0.078	-0.137	0.137	TRUE
Angptl3	132-137	60 min	0.037	-0.297	0.297	TRUE
Angptl3	134-144	10 s	0.242	0.076	0.076	FALSE
Angptl3	134-144	2 min	0.284	-0.057	0.057	FALSE
Angptl3	134-144	10 min	0.228	-0.053	0.053	FALSE
Angptl3	134-144	60 min	0.438	0.367	0.367	FALSE
Angptl3	134-162	10 s	0.299	0.131	0.131	FALSE
Angptl3	134-162	2 min	0.300	-0.442	0.442	TRUE
Angptl3	134-162	10 min	0.426	-0.341	0.341	FALSE
Angptl3	134-162	60 min	0.308	0.237	0.237	FALSE
Angptl3	134-167	10 s	0.298	-0.467	0.467	TRUE

Protein	Peptide	Exposure	U=ku k=2	Δ (HX)t	ABS(Δ (HX)t)	True = significant difference, reject Null (no difference)
Angptl3	134-167	10 min	0.410	-0.176	0.176	FALSE
Angptl3	134-167	60 min	0.456	0.297	0.297	FALSE
Angptl3	138-148	10 s	0.951	-0.139	0.139	FALSE
Angptl3	138-148	2 min	0.096	-0.110	0.110	TRUE
Angptl3	138-148	10 min	0.074	-0.027	0.027	FALSE
Angptl3	138-148	60 min	0.065	0.035	0.035	FALSE
Angptl3	138-162	10 s	0.256	-0.074	0.074	FALSE
Angptl3	138-162	2 min	0.271	-0.348	0.348	TRUE
Angptl3	138-162	10 min	0.460	0.002	0.002	FALSE
Angptl3	138-162	60 min	0.237	0.114	0.114	FALSE
Angptl3	138-165	10 s	0.203	-0.113	0.113	FALSE
Angptl3	138-165	2 min	0.259	-0.304	0.304	TRUE
Angptl3	138-165	10 min	0.203	-0.042	0.042	FALSE
Angptl3	138-165	60 min	0.282	0.331	0.331	TRUE
Angptl3	138-166	10 s	0.274	-0.220	0.220	FALSE
Angptl3	138-166	2 min	0.490	-0.439	0.439	FALSE
Angptl3	138-166	10 min	0.440	-0.115	0.115	FALSE
Angptl3	138-166	60 min	0.291	0.268	0.268	FALSE
Angptl3	138-167	10 s	0.228	-0.480	0.480	TRUE
Angptl3	138-167	2 min	0.341	-0.613	0.613	TRUE
Angptl3	138-167	10 min	0.242	-0.215	0.215	FALSE
Angptl3	138-167	60 min	0.239	0.196	0.196	FALSE
Angptl3	149-167	10 s	0.199	-0.094	0.094	FALSE
Angptl3	149-167	2 min	0.373	-0.157	0.157	FALSE
Angptl3	149-167	10 min	0.174	0.143	0.143	FALSE
Angptl3	149-167	60 min	0.176	0.309	0.309	TRUE
Angptl3	168-178	10 s	0.082	-0.394	0.394	TRUE
Angptl3	168-178	2 min	0.126	-1.145	1.145	TRUE
Angptl3	168-178	10 min	0.124	-0.856	0.856	TRUE
Angptl3	168-178	60 min	0.106	-0.299	0.299	TRUE
Angptl3	168-181	10 s	0.196	-0.716	0.716	TRUE
Angptl3	168-181	2 min	0.195	-1.521	1.521	TRUE
Angptl3	168-181	10 min	0.217	-1.453	1.453	TRUE
Angptl3	168-181	60 min	0.239	-0.615	0.615	TRUE
Angptl3	168-184	10 s	0.258	-0.870	0.870	TRUE
Angptl3	168-184	2 min	0.351	-2.287	2.287	TRUE
Angptl3	168-184	10 min	0.328	-2.466	2.466	TRUE
Angptl3	168-184	60 min	0.351	-0.808	0.808	TRUE
Angptl3	168-189	10 s	0.295	-1.077	1.077	TRUE
Angptl3	168-189	2 min	0.323	-3.660	3.660	TRUE
Angptl3	168-189	10 min	0.454	-4.116	4.116	TRUE
Angptl3	168-189	60 min	0.268	-1.878	1.878	TRUE
Angptl3	171-189	10 s	0.338	-1.072	1.072	TRUE
Angptl3	171-189	2 min	0.264	-3.475	3.475	TRUE
Angptl3	171-189	10 min	0.371	-4.045	4.045	TRUE
Angptl3	171-189	60 min	0.462	-1.997	1.997	TRUE
Angptl3	173-189	10 s	0.212	-1.438	1.438	TRUE
Angptl3	173-189	2 min	0.289	-3.667	3.667	TRUE
Angptl3	173-189	10 min	0.392	-3.916	3.916	TRUE
Angptl3	173-189	60 min	0.295	-1.899	1.899	TRUE
Angptl3	179-189	10 s	0.080	-0.678	0.678	TRUE
Angptl3	179-189	2 min	0.145	-1.897	1.897	TRUE
Angptl3	179-189	10 min	0.133	-2.205	2.205	TRUE
Angptl3	179-189	60 min	0.155	-1.096	1.096	TRUE
Angptl3	190-197	10 s	0.145	-0.655	0.655	TRUE
Angptl3	190-197	2 min	0.095	-1.044	1.044	TRUE
Angptl3	190-197	10 min	0.317	-0.730	0.730	TRUE
Angptl3	190-197	60 min	0.442	-0.220	0.220	FALSE
Angptl3	190-200	10 s	0.188	-0.730	0.730	TRUE
Angptl3	190-200	2 min	0.126	-1.645	1.645	TRUE

Protein	Peptide	Exposure	U=ku k=2	Δ (HX)t	ABS(Δ (HX)t)	True = significant difference, reject Null (no difference)
Angptl3	190-200	60 min	0.254	-0.401	0.401	TRUE
Angptl3	190-205	10 s	0.176	-0.708	0.708	TRUE
Angptl3	190-205	2 min	0.309	-1.511	1.511	TRUE
Angptl3	190-205	10 min	0.235	-1.384	1.384	TRUE
Angptl3	190-205	60 min	0.223	-0.754	0.754	TRUE
Angptl3	190-216	10 s	0.424	0.099	0.099	FALSE
Angptl3	190-216	2 min	0.631	-1.288	1.288	TRUE
Angptl3	190-216	10 min	0.583	-1.378	1.378	TRUE
Angptl3	190-216	60 min	0.428	-0.626	0.626	TRUE
Angptl3	190-219	10 s	0.341	0.063	0.063	FALSE
Angptl3	190-219	2 min	0.632	-1.235	1.235	TRUE
Angptl3	190-219	10 min	0.427	-1.199	1.199	TRUE
Angptl3	190-219	60 min	0.598	-0.482	0.482	FALSE
Angptl3	204-219	10 s	0.337	0.216	0.216	FALSE
Angptl3	204-219	2 min	0.394	0.426	0.426	TRUE
Angptl3	204-219	10 min	0.337	0.269	0.269	FALSE
Angptl3	204-219	60 min	0.360	0.102	0.102	FALSE
Angptl3	220-231	10 s	0.138	0.060	0.060	FALSE
Angptl3	220-231	2 min	0.202	-0.149	0.149	FALSE
Angptl3	220-231	10 min	0.252	-0.049	0.049	FALSE
Angptl3	220-231	60 min	0.143	-0.087	0.087	FALSE
Angptl3	232-259	10 s	0.990	0.263	0.263	FALSE
Angptl3	232-259	2 min	0.904	-0.550	0.550	FALSE
Angptl3	232-259	10 min	0.826	-0.369	0.369	FALSE
Angptl3	232-259	60 min	0.501	-0.635	0.635	TRUE
Angptl3	236-259	10 s	0.862	0.459	0.459	FALSE
Angptl3	236-259	2 min	1.015	-0.511	0.511	FALSE
Angptl3	236-259	10 min	0.823	-0.298	0.298	FALSE
Angptl3	236-259	60 min	0.832	-0.332	0.332	FALSE
Angptl3	260-273	10 s	0.106	0.221	0.221	TRUE
Angptl3	260-273	2 min	0.124	0.024	0.024	FALSE
Angptl3	260-273	10 min	0.147	0.052	0.052	FALSE
Angptl3	260-273	60 min	0.056	-0.070	0.070	TRUE
Angptl3	274-284	10 s	0.099	0.374	0.374	TRUE
Angptl3	274-284	2 min	0.107	0.304	0.304	TRUE
Angptl3	274-284	10 min	0.163	0.212	0.212	TRUE
Angptl3	274-284	60 min	0.129	0.118	0.118	FALSE
Angptl3	285-290	10 s	0.038	0.060	0.060	TRUE
Angptl3	285-290	2 min	0.048	-0.008	0.008	FALSE
Angptl3	285-290	10 min	0.076	0.042	0.042	FALSE
Angptl3	285-290	60 min	0.075	0.025	0.025	FALSE
Angptl3	302-309	10 s	0.076	0.130	0.130	TRUE
Angptl3	302-309	2 min	0.069	0.115	0.115	TRUE
Angptl3	302-309	10 min	0.059	0.134	0.134	TRUE
Angptl3	302-309	60 min	0.098	0.032	0.032	FALSE
Angptl3	303-309	10 s	0.088	0.051	0.051	FALSE
Angptl3	303-309	2 min	0.069	-0.067	0.067	FALSE
Angptl3	303-309	10 min	0.062	-0.010	0.010	FALSE
Angptl3	303-309	60 min	0.048	-0.032	0.032	FALSE
Angptl3	304-309	10 s	0.040	0.056	0.056	TRUE
Angptl3	304-309	2 min	0.049	-0.016	0.016	FALSE
Angptl3	304-309	10 min	0.045	-0.025	0.025	FALSE
Angptl3	304-309	60 min	0.034	0.047	0.047	TRUE
Angptl3	310-316	10 s	0.134	-0.157	0.157	TRUE
Angptl3	310-316	2 min	0.130	-0.154	0.154	TRUE
Angptl3	310-316	10 min	0.183	-0.156	0.156	FALSE
Angptl3	310-316	60 min	0.140	-0.145	0.145	TRUE
Angptl3	310-319	10 s	0.084	0.170	0.170	TRUE
Angptl3	310-319	2 min	0.072	0.200	0.200	TRUE
Angptl3	310-319	10 min	0.089	0.234	0.234	TRUE

Protein	Peptide	Exposure	U=ku k=2	Δ (HX)t	ABS(Δ (HX)t)	True = significant difference, reject Null (no difference)
Angptl3	313-319	10 s	0.061	0.117	0.117	TRUE
Angptl3	313-319	2 min	0.057	0.119	0.119	TRUE
Angptl3	313-319	10 min	0.042	0.111	0.111	TRUE
Angptl3	313-319	60 min	0.035	0.093	0.093	TRUE
Angptl3	317-328	10 s	0.121	0.105	0.105	FALSE
Angptl3	317-328	2 min	0.114	0.019	0.019	FALSE
Angptl3	317-328	10 min	0.134	0.122	0.122	FALSE
Angptl3	317-328	60 min	0.088	0.126	0.126	TRUE
Angptl3	320-325	10 s	0.051	0.102	0.102	TRUE
Angptl3	320-325	2 min	0.051	0.111	0.111	TRUE
Angptl3	320-325	10 min	0.051	0.131	0.131	TRUE
Angptl3	320-325	60 min	0.040	0.154	0.154	TRUE
Angptl3	320-328	10 s	0.037	0.125	0.125	TRUE
Angptl3	320-328	2 min	0.043	0.082	0.082	TRUE
Angptl3	320-328	10 min	0.060	0.118	0.118	TRUE
Angptl3	320-328	60 min	0.064	0.097	0.097	TRUE
Angptl3	320-329	10 s	0.028	0.190	0.190	TRUE
Angptl3	320-329	2 min	0.059	0.093	0.093	TRUE
Angptl3	320-329	10 min	0.083	0.068	0.068	FALSE
Angptl3	320-329	60 min	0.103	0.066	0.066	FALSE
Angptl3	320-331	10 s	0.047	0.290	0.290	TRUE
Angptl3	320-331	2 min	0.038	0.098	0.098	TRUE
Angptl3	320-331	10 min	0.093	0.198	0.198	TRUE
Angptl3	320-331	60 min	0.063	0.147	0.147	TRUE
Angptl3	329-334	10 s	0.057	0.046	0.046	FALSE
Angptl3	329-334	2 min	0.042	0.054	0.054	TRUE
Angptl3	329-334	10 min	0.034	0.070	0.070	TRUE
Angptl3	329-334	60 min	0.032	0.054	0.054	TRUE
Angptl3	333-342	10 s	0.217	0.156	0.156	FALSE
Angptl3	333-342	2 min	0.284	-0.042	0.042	FALSE
Angptl3	333-342	10 min	0.225	0.085	0.085	FALSE
Angptl3	333-342	60 min	0.264	0.101	0.101	FALSE
Angptl3	335-340	10 s	0.090	0.144	0.144	TRUE
Angptl3	335-340	2 min	0.078	0.005	0.005	FALSE
Angptl3	335-340	10 min	0.113	0.101	0.101	FALSE
Angptl3	335-340	60 min	0.084	0.113	0.113	TRUE
Angptl3	335-342	10 s	0.072	0.045	0.045	FALSE
Angptl3	335-342	2 min	0.094	-0.146	0.146	TRUE
Angptl3	335-342	10 min	0.085	-0.015	0.015	FALSE
Angptl3	335-342	60 min	0.075	-0.038	0.038	FALSE
Angptl3	335-346	10 s	0.114	-0.083	0.083	FALSE
Angptl3	335-346	2 min	0.167	-0.124	0.124	FALSE
Angptl3	335-346	10 min	0.198	-0.085	0.085	FALSE
Angptl3	335-346	60 min	0.197	-0.224	0.224	TRUE
Angptl3	337-346	10 s	0.091	-0.022	0.022	FALSE
Angptl3	337-346	2 min	0.102	-0.057	0.057	FALSE
Angptl3	337-346	10 min	0.115	0.004	0.004	FALSE
Angptl3	337-346	60 min	0.145	0.002	0.002	FALSE
Angptl3	341-346	10 s	0.126	0.057	0.057	FALSE
Angptl3	341-346	2 min	0.123	0.078	0.078	FALSE
Angptl3	341-346	10 min	0.118	0.046	0.046	FALSE
Angptl3	341-346	60 min	0.137	0.019	0.019	FALSE
Angptl3	347-354	10 s	0.353	0.018	0.018	FALSE
Angptl3	347-354	2 min	0.358	0.113	0.113	FALSE
Angptl3	347-354	10 min	0.356	0.221	0.221	FALSE
Angptl3	347-354	60 min	0.356	0.107	0.107	FALSE
Angptl3	363-377	10 s	0.093	0.369	0.369	TRUE
Angptl3	363-377	2 min	0.155	0.058	0.058	FALSE
Angptl3	363-377	10 min	0.260	0.093	0.093	FALSE
Angptl3	363-377	60 min	0.202	-0.024	0.024	FALSE

Protein	Peptide	Exposure	U=ku k=2	Δ (HX)t	ABS(Δ (HX)t)	True = significant difference, reject Null (no difference)
Angptl3	363-378	2 min	0.190	-0.051	0.051	FALSE
Angptl3	363-378	10 min	0.291	0.046	0.046	FALSE
Angptl3	363-378	60 min	0.293	-0.028	0.028	FALSE
Angptl3	363-379	10 s	0.098	0.391	0.391	TRUE
Angptl3	363-379	2 min	0.111	0.080	0.080	FALSE
Angptl3	363-379	10 min	0.274	0.130	0.130	FALSE
Angptl3	363-379	60 min	0.214	0.036	0.036	FALSE
Angptl3	363-381	10 s	0.188	0.162	0.162	FALSE
Angptl3	363-381	2 min	0.169	-0.170	0.170	TRUE
Angptl3	363-381	10 min	0.301	-0.349	0.349	TRUE
Angptl3	363-381	60 min	0.201	-0.669	0.669	TRUE
Angptl3	365-377	10 s	0.115	0.313	0.313	TRUE
Angptl3	365-377	2 min	0.153	-0.067	0.067	FALSE
Angptl3	365-377	10 min	0.259	-0.001	0.001	FALSE
Angptl3	365-377	60 min	0.206	-0.055	0.055	FALSE
Angptl3	365-379	10 s	0.115	0.335	0.335	TRUE
Angptl3	365-379	2 min	0.168	-0.040	0.040	FALSE
Angptl3	365-379	10 min	0.240	0.067	0.067	FALSE
Angptl3	365-379	60 min	0.142	-0.049	0.049	FALSE
Angptl3	378-386	10 s	0.033	0.189	0.189	TRUE
Angptl3	378-386	2 min	0.045	-0.020	0.020	FALSE
Angptl3	378-386	10 min	0.112	0.027	0.027	FALSE
Angptl3	378-386	60 min	0.081	0.019	0.019	FALSE
Angptl3	379-386	10 s	0.040	0.015	0.015	FALSE
Angptl3	379-386	2 min	0.063	-0.046	0.046	FALSE
Angptl3	379-386	10 min	0.102	0.005	0.005	FALSE
Angptl3	379-386	60 min	0.080	-0.013	0.013	FALSE
Angptl3	408-416	10 s	0.189	0.180	0.180	FALSE
Angptl3	408-416	2 min	0.170	-0.153	0.153	FALSE
Angptl3	408-416	10 min	0.252	-0.001	0.001	FALSE
Angptl3	408-416	60 min	0.272	-0.085	0.085	FALSE
Angptl3	408-432	10 s	0.287	0.366	0.366	TRUE
Angptl3	408-432	2 min	0.399	-0.238	0.238	FALSE
Angptl3	408-432	10 min	0.392	0.169	0.169	FALSE
Angptl3	408-432	60 min	0.298	0.018	0.018	FALSE
Angptl3	408-441	10 s	0.532	0.363	0.363	FALSE
Angptl3	408-441	2 min	0.715	-0.289	0.289	FALSE
Angptl3	408-441	10 min	0.673	0.217	0.217	FALSE
Angptl3	408-441	60 min	0.584	-0.317	0.317	FALSE
Angptl3	408-445	10 s	0.516	0.550	0.550	TRUE
Angptl3	408-445	2 min	0.736	-0.051	0.051	FALSE
Angptl3	408-445	10 min	0.565	0.355	0.355	FALSE
Angptl3	408-445	60 min	0.562	0.081	0.081	FALSE
Angptl3	417-441	10 s	0.526	0.185	0.185	FALSE
Angptl3	417-441	2 min	0.691	-0.063	0.063	FALSE
Angptl3	417-441	10 min	0.671	0.045	0.045	FALSE
Angptl3	417-441	60 min	0.440	-0.457	0.457	TRUE
Angptl3	417-445	10 s	0.434	0.265	0.265	FALSE
Angptl3	417-445	2 min	0.556	0.078	0.078	FALSE
Angptl3	417-445	10 min	0.549	0.134	0.134	FALSE
Angptl3	417-445	60 min	0.381	-0.229	0.229	FALSE
Angptl3	433-441	10 s	0.081	0.097	0.097	TRUE
Angptl3	433-441	2 min	0.106	-0.132	0.132	TRUE
Angptl3	433-441	10 min	0.131	-0.022	0.022	FALSE
Angptl3	433-441	60 min	0.125	-0.063	0.063	FALSE
Angptl3	433-445	10 s	0.243	-0.075	0.075	FALSE
Angptl3	433-445	2 min	0.257	-0.319	0.319	TRUE
Angptl3	433-445	10 min	0.249	-0.112	0.112	FALSE
Angptl3	433-445	60 min	0.248	0.004	0.004	FALSE
Angptl3	442-448	10 s	0.099	0.019	0.019	FALSE

Protein	Peptide	Exposure	U=ku k=2	Δ (HX)t	ABS(Δ (HX)t)	True = significant difference, reject Null (no difference)
Angptl3	442-448	10 min	0.086	0.006	0.006	FALSE
Angptl3	442-448	60 min	0.067	-0.166	0.166	TRUE

Table S2: Differential HX, combined uncertainty, and significance testing for individual HX times for ANGPTL8 peptides in the ANGPTL3/8 complex compared to ANGPTL8 alone

Protein	peptide	Exposure	U=ku k=2	Δ (HX)t	ABS(Δ (HX)t)	True = significant difference, reject Null (no difference)
ANGPTL8	38-49	10 s	0.183	-0.537	0.537	TRUE
ANGPTL8	38-49	2 min	0.259	-1.383	1.383	TRUE
ANGPTL8	38-49	10 min	0.175	-1.768	1.768	TRUE
ANGPTL8	38-49	60 min	0.139	-2.218	2.218	TRUE
ANGPTL8	39-49	10 s	0.097	-0.764	0.764	TRUE
ANGPTL8	39-49	2 min	0.178	-1.639	1.639	TRUE
ANGPTL8	39-49	10 min	0.329	-2.192	2.192	TRUE
ANGPTL8	39-49	60 min	0.138	-2.191	2.191	TRUE
ANGPTL8	49-59	10 s	0.132	1.031	1.031	TRUE
ANGPTL8	49-59	2 min	0.190	0.305	0.305	TRUE
ANGPTL8	49-59	10 min	0.359	-0.636	0.636	TRUE
ANGPTL8	49-59	60 min	0.248	-0.969	0.969	TRUE
ANGPTL8	50-64	10 s	0.210	2.173	2.173	TRUE
ANGPTL8	50-64	2 min	0.408	0.759	0.759	TRUE
ANGPTL8	50-64	10 min	0.244	-0.578	0.578	TRUE
ANGPTL8	50-64	60 min	0.330	-0.877	0.877	TRUE
ANGPTL8	50-76	10 s	0.595	2.588	2.588	TRUE
ANGPTL8	50-76	2 min	0.427	0.575	0.575	TRUE
ANGPTL8	50-76	10 min	0.369	-1.726	1.726	TRUE
ANGPTL8	50-76	60 min	0.374	-3.027	3.027	TRUE
ANGPTL8	65-76	10 s	0.316	0.573	0.573	TRUE
ANGPTL8	65-76	2 min	0.184	0.221	0.221	TRUE
ANGPTL8	65-76	10 min	0.526	-0.938	0.938	TRUE
ANGPTL8	65-76	60 min	0.142	-1.347	1.347	TRUE
ANGPTL8	77-91	10 s	0.088	5.280	5.280	TRUE
ANGPTL8	77-91	2 min	0.119	4.405	4.405	TRUE
ANGPTL8	77-91	10 min	0.248	2.657	2.657	TRUE
ANGPTL8	77-91	60 min	0.178	1.270	1.270	TRUE
ANGPTL8	77-95	10 s	0.304	6.914	6.914	TRUE
ANGPTL8	77-95	2 min	0.170	5.916	5.916	TRUE
ANGPTL8	77-95	10 min	0.310	3.776	3.776	TRUE
ANGPTL8	77-95	60 min	0.176	2.563	2.563	TRUE
ANGPTL8	81-103	10 s	0.481	7.177	7.177	TRUE
ANGPTL8	81-103	2 min	0.229	6.940	6.940	TRUE
ANGPTL8	81-103	10 min	0.358	4.862	4.862	TRUE
ANGPTL8	81-103	60 min	0.157	2.063	2.063	TRUE
ANGPTL8	96-103	10 s	0.108	1.775	1.775	TRUE
ANGPTL8	96-103	2 min	0.141	1.776	1.776	TRUE
ANGPTL8	96-103	10 min	0.068	1.534	1.534	TRUE
ANGPTL8	96-103	60 min	0.088	0.559	0.559	TRUE
ANGPTL8	108-128	10 s	0.195	3.757	3.757	TRUE
ANGPTL8	108-128	2 min	0.144	2.992	2.992	TRUE
ANGPTL8	108-128	10 min	0.387	1.317	1.317	TRUE
ANGPTL8	108-128	60 min	0.288	-0.107	0.107	FALSE
ANGPTL8	108-133	10 s	0.887	3.320	3.320	TRUE
ANGPTL8	108-133	2 min	1.011	3.156	3.156	TRUE
ANGPTL8	108-133	10 min	0.289	1.527	1.527	TRUE
ANGPTL8	108-133	60 min	0.572	0.032	0.032	FALSE
ANGPTL8	125-133	2 min	0.058	0.624	0.624	TRUE
ANGPTL8	125-133	10 min	0.084	0.480	0.480	TRUE
ANGPTL8	125-133	60 min	0.072	0.222	0.222	TRUE
ANGPTL8	134-140	10 s	0.066	0.552	0.552	TRUE
ANGPTL8	134-140	2 min	0.086	0.372	0.372	TRUE
ANGPTL8	134-140	10 min	0.103	0.256	0.256	TRUE
ANGPTL8	134-140	60 min	0.096	0.114	0.114	TRUE

Protein	peptide	Exposure	U=ku k=2	Δ (HX)t	ABS(Δ (HX)t)	True = significant difference, reject Null (no difference)
ANGPTL8	139-148	2 min	0.424	-0.517	0.517	TRUE
ANGPTL8	139-148	10 min	0.568	-0.680	0.680	TRUE
ANGPTL8	139-148	60 min	0.331	-0.486	0.486	TRUE
ANGPTL8	153-163	10 s	0.302	0.313	0.313	TRUE
ANGPTL8	153-163	2 min	0.285	-0.012	0.012	FALSE
ANGPTL8	153-163	10 min	0.349	-0.233	0.233	FALSE
ANGPTL8	153-163	60 min	0.428	-0.442	0.442	TRUE
ANGPTL8	164-172	10 s	0.238	0.769	0.769	TRUE
ANGPTL8	164-172	2 min	0.250	0.712	0.712	TRUE
ANGPTL8	164-172	10 min	0.364	0.584	0.584	TRUE
ANGPTL8	164-172	60 min	0.166	-0.270	0.270	TRUE
ANGPTL8	164-174	10 s	0.145	0.566	0.566	TRUE
ANGPTL8	164-174	2 min	0.131	0.743	0.743	TRUE
ANGPTL8	164-174	10 min	0.140	0.725	0.725	TRUE
ANGPTL8	164-174	60 min	0.164	0.176	0.176	TRUE
ANGPTL8	164-176	10 s	0.242	0.897	0.897	TRUE
ANGPTL8	164-176	2 min	0.054	0.899	0.899	TRUE
ANGPTL8	164-176	10 min	0.211	0.641	0.641	TRUE
ANGPTL8	164-176	60 min	0.095	0.150	0.150	TRUE
ANGPTL8	164-177	10 s	0.877	1.155	1.155	TRUE
ANGPTL8	164-177	2 min	0.137	1.520	1.520	TRUE
ANGPTL8	164-177	10 min	0.075	0.997	0.997	TRUE
ANGPTL8	164-177	60 min	0.091	0.451	0.451	TRUE
ANGPTL8	178-190	10 s	0.135	1.615	1.615	TRUE
ANGPTL8	178-190	2 min	0.180	1.408	1.408	TRUE
ANGPTL8	178-190	10 min	0.129	1.345	1.345	TRUE
ANGPTL8	178-190	60 min	0.203	1.195	1.195	TRUE
ANGPTL8	178-198	10 s	0.260	1.527	1.527	TRUE
ANGPTL8	178-198	2 min	0.295	1.244	1.244	TRUE
ANGPTL8	178-198	10 min	0.348	1.207	1.207	TRUE
ANGPTL8	178-198	60 min	0.285	0.974	0.974	TRUE
ANGPTL8	185-198	10 s	0.204	0.541	0.541	TRUE
ANGPTL8	185-198	2 min	0.232	0.232	0.232	FALSE
ANGPTL8	185-198	10 min	0.227	0.237	0.237	TRUE
ANGPTL8	185-198	60 min	0.193	0.165	0.165	FALSE
ANGPTL8	191-198	10 s	0.158	0.043	0.043	FALSE
ANGPTL8	191-198	2 min	0.150	0.245	0.245	TRUE
ANGPTL8	191-198	10 min	0.162	0.130	0.130	FALSE
ANGPTL8	191-198	60 min	0.133	0.066	0.066	FALSE

Table S3: Differential HX, combined uncertainty, and significance testing for individual HX times for ANGPTL3 and ANGPTL8 peptides in ANGPTL3/8 + LPL compared to unbound ANGPTL3/8

Protein	peptide	Exposure	U=ku k=2	Δ (HX)t	ABS(Δ (HX)t)	True = significant difference, reject Null (no difference)
Angptl3	17-36	10 s	0.789	-0.483	0.483	FALSE
Angptl3	17-36	2 min	0.794	-0.464	0.464	FALSE
Angptl3	17-36	10 min	0.887	-0.512	0.512	FALSE
Angptl3	17-36	60 min	0.778	-0.523	0.523	FALSE
Angptl3	17-40	10 s	0.841	-0.351	0.351	FALSE
Angptl3	17-40	2 min	0.840	-0.294	0.294	FALSE
Angptl3	17-40	10 min	0.905	-0.411	0.411	FALSE
Angptl3	17-40	60 min	0.759	-0.418	0.418	FALSE
Angptl3	17-46	10 s	0.800	0.662	0.662	FALSE
Angptl3	17-46	2 min	0.883	0.595	0.595	FALSE
Angptl3	17-46	10 min	0.910	-0.061	0.061	FALSE
Angptl3	17-46	60 min	0.736	-0.300	0.300	FALSE
Angptl3	37-42	10 s	0.151	-0.073	0.073	FALSE
Angptl3	37-42	2 min	0.118	-0.071	0.071	FALSE
Angptl3	37-42	10 min	0.125	0.012	0.012	FALSE
Angptl3	37-42	60 min	0.109	-0.023	0.023	FALSE
Angptl3	37-46	10 s	0.087	0.854	0.854	TRUE
Angptl3	37-46	2 min	0.129	0.931	0.931	TRUE
Angptl3	37-46	10 min	0.121	0.452	0.452	TRUE
Angptl3	37-46	60 min	0.177	0.144	0.144	FALSE
Angptl3	41-46	10 s	0.074	0.769	0.769	TRUE
Angptl3	41-46	2 min	0.082	0.754	0.754	TRUE
Angptl3	41-46	10 min	0.106	0.349	0.349	TRUE
Angptl3	41-46	60 min	0.103	0.056	0.056	FALSE
Angptl3	45-58	10 s	0.147	1.092	1.092	TRUE
Angptl3	45-58	2 min	0.192	1.710	1.710	TRUE
Angptl3	45-58	10 min	0.279	1.971	1.971	TRUE
Angptl3	45-58	60 min	0.265	1.128	1.128	TRUE
Angptl3	45-59	10 s	0.165	1.080	1.080	TRUE
Angptl3	45-59	2 min	0.156	1.815	1.815	TRUE
Angptl3	45-59	10 min	0.503	2.337	2.337	TRUE
Angptl3	45-59	60 min	0.396	1.320	1.320	TRUE
Angptl3	45-60	10 s	0.098	0.975	0.975	TRUE
Angptl3	45-60	2 min	0.083	1.858	1.858	TRUE
Angptl3	45-60	10 min	0.142	2.020	2.020	TRUE
Angptl3	45-60	60 min	0.320	1.261	1.261	TRUE
Angptl3	47-53	10 s	0.088	0.503	0.503	TRUE
Angptl3	47-53	2 min	0.074	1.008	1.008	TRUE
Angptl3	47-53	10 min	0.067	1.199	1.199	TRUE
Angptl3	47-53	60 min	0.114	0.800	0.800	TRUE
Angptl3	47-58	10 s	0.131	0.786	0.786	TRUE
Angptl3	47-58	2 min	0.166	1.457	1.457	TRUE
Angptl3	47-58	10 min	0.178	1.904	1.904	TRUE
Angptl3	47-58	60 min	0.257	1.155	1.155	TRUE
Angptl3	47-59	10 s	0.119	0.691	0.691	TRUE
Angptl3	47-59	2 min	0.153	1.291	1.291	TRUE
Angptl3	47-59	10 min	0.177	1.778	1.778	TRUE
Angptl3	47-59	60 min	0.280	1.065	1.065	TRUE
Angptl3	47-60	10 s	0.123	0.651	0.651	TRUE
Angptl3	47-60	2 min	0.135	1.203	1.203	TRUE
Angptl3	47-60	10 min	0.203	1.669	1.669	TRUE
Angptl3	47-60	60 min	0.274	1.104	1.104	TRUE
Angptl3	47-74	10 s	0.491	1.052	1.052	TRUE
Angptl3	47-74	2 min	0.788	2.061	2.061	TRUE
Angptl3	47-74	10 min	1.036	2.899	2.899	TRUE
Angptl3	47-74	60 min	1.363	2.128	2.128	TRUE

Protein	peptide	Exposure	U=ku k=2	Δ (HX)t	ABS(Δ (HX)t)	True = significant difference, reject Null (no difference)
Angptl3	47-75	2 min	0.959	1.758	1.758	TRUE
Angptl3	47-75	10 min	1.281	2.683	2.683	TRUE
Angptl3	47-75	60 min	1.575	2.256	2.256	TRUE
Angptl3	47-76	10 s	1.389	1.031	1.031	FALSE
Angptl3	47-76	2 min	2.727	2.164	2.164	FALSE
Angptl3	47-76	10 min	2.398	3.005	3.005	TRUE
Angptl3	47-76	60 min	2.417	2.207	2.207	FALSE
Angptl3	47-78	10 s	0.467	0.931	0.931	TRUE
Angptl3	47-78	2 min	0.735	1.830	1.830	TRUE
Angptl3	47-78	10 min	0.963	2.845	2.845	TRUE
Angptl3	47-78	60 min	1.226	2.257	2.257	TRUE
Angptl3	51-58	10 s	0.092	0.259	0.259	TRUE
Angptl3	51-58	2 min	0.182	0.495	0.495	TRUE
Angptl3	51-58	10 min	0.319	0.779	0.779	TRUE
Angptl3	51-58	60 min	0.422	0.581	0.581	TRUE
Angptl3	59-75	10 s	0.482	0.433	0.433	FALSE
Angptl3	59-75	2 min	0.987	0.469	0.469	FALSE
Angptl3	59-75	10 min	1.014	0.716	0.716	FALSE
Angptl3	59-75	60 min	1.404	0.218	0.218	FALSE
Angptl3	59-78	10 s	0.269	0.400	0.400	TRUE
Angptl3	59-78	2 min	0.509	0.363	0.363	FALSE
Angptl3	59-78	10 min	0.798	0.746	0.746	FALSE
Angptl3	59-78	60 min	1.024	1.518	1.518	TRUE
Angptl3	60-74	10 s	0.147	0.240	0.240	TRUE
Angptl3	60-74	2 min	0.183	0.350	0.350	TRUE
Angptl3	60-74	10 min	0.272	0.771	0.771	TRUE
Angptl3	60-74	60 min	0.406	0.894	0.894	TRUE
Angptl3	60-76	10 s	0.330	0.224	0.224	FALSE
Angptl3	60-76	2 min	0.403	0.339	0.339	FALSE
Angptl3	60-76	10 min	0.486	0.750	0.750	TRUE
Angptl3	60-76	60 min	0.642	0.894	0.894	TRUE
Angptl3	60-78	10 s	0.431	0.306	0.306	FALSE
Angptl3	60-78	2 min	0.471	0.308	0.308	FALSE
Angptl3	60-78	10 min	0.435	1.110	1.110	TRUE
Angptl3	60-78	60 min	0.640	1.236	1.236	TRUE
Angptl3	61-74	10 s	0.326	0.190	0.190	FALSE
Angptl3	61-74	2 min	0.392	0.300	0.300	FALSE
Angptl3	61-74	10 min	0.571	0.667	0.667	TRUE
Angptl3	61-74	60 min	0.948	0.749	0.749	FALSE
Angptl3	61-75	10 s	0.290	0.225	0.225	FALSE
Angptl3	61-75	2 min	0.326	0.346	0.346	TRUE
Angptl3	61-75	10 min	0.396	0.847	0.847	TRUE
Angptl3	61-75	60 min	0.611	1.129	1.129	TRUE
Angptl3	61-78	10 s	0.194	0.314	0.314	TRUE
Angptl3	61-78	2 min	0.339	0.263	0.263	FALSE
Angptl3	61-78	10 min	0.523	0.794	0.794	TRUE
Angptl3	61-78	60 min	0.898	0.773	0.773	FALSE
Angptl3	75-80	10 s	0.099	0.062	0.062	FALSE
Angptl3	75-80	2 min	0.069	0.301	0.301	TRUE
Angptl3	75-80	10 min	0.080	0.303	0.303	TRUE
Angptl3	75-80	60 min	0.091	0.204	0.204	TRUE
Angptl3	75-82	10 s	0.337	0.165	0.165	FALSE
Angptl3	75-82	2 min	0.562	0.122	0.122	FALSE
Angptl3	75-82	10 min	0.842	0.175	0.175	FALSE
Angptl3	75-82	60 min	1.056	0.135	0.135	FALSE
Angptl3	76-82	10 s	0.139	0.133	0.133	FALSE
Angptl3	76-82	2 min	0.187	0.060	0.060	FALSE
Angptl3	76-82	10 min	0.291	0.150	0.150	FALSE
Angptl3	76-82	60 min	0.390	0.054	0.054	FALSE
Angptl3	79-85	10 s	0.087	0.111	0.111	TRUE

Protein	peptide	Exposure	U=ku k=2	Δ (HX)t	ABS(Δ (HX)t)	True = significant difference, reject Null (no difference)
Angptl3	79-85	10 min	0.080	0.111	0.111	TRUE
Angptl3	79-85	60 min	0.048	0.083	0.083	TRUE
Angptl3	86-93	10 s	0.160	0.114	0.114	FALSE
Angptl3	86-93	2 min	0.130	0.015	0.015	FALSE
Angptl3	86-93	10 min	0.159	0.139	0.139	FALSE
Angptl3	86-93	60 min	0.174	0.199	0.199	TRUE
Angptl3	86-97	10 s	0.210	0.066	0.066	FALSE
Angptl3	86-97	2 min	0.143	0.057	0.057	FALSE
Angptl3	86-97	10 min	0.237	0.283	0.283	TRUE
Angptl3	86-97	60 min	0.317	0.243	0.243	FALSE
Angptl3	86-106	10 s	0.274	0.274	0.274	FALSE
Angptl3	86-106	2 min	0.433	0.189	0.189	FALSE
Angptl3	86-106	10 min	0.689	0.398	0.398	FALSE
Angptl3	86-106	60 min	1.117	0.318	0.318	FALSE
Angptl3	86-109	10 s	0.396	0.307	0.307	FALSE
Angptl3	86-109	2 min	0.377	0.258	0.258	FALSE
Angptl3	86-109	10 min	0.556	0.490	0.490	FALSE
Angptl3	86-109	60 min	0.966	0.483	0.483	FALSE
Angptl3	88-97	10 s	0.250	0.043	0.043	FALSE
Angptl3	88-97	2 min	0.260	0.180	0.180	FALSE
Angptl3	88-97	10 min	0.185	0.384	0.384	TRUE
Angptl3	88-97	60 min	0.667	0.058	0.058	FALSE
Angptl3	98-109	10 s	0.139	0.049	0.049	FALSE
Angptl3	98-109	2 min	0.174	0.032	0.032	FALSE
Angptl3	98-109	10 min	0.306	0.085	0.085	FALSE
Angptl3	98-109	60 min	0.564	-0.028	0.028	FALSE
Angptl3	99-109	10 s	0.111	0.107	0.107	FALSE
Angptl3	99-109	2 min	0.153	0.047	0.047	FALSE
Angptl3	99-109	10 min	0.284	0.108	0.108	FALSE
Angptl3	99-109	60 min	0.469	0.025	0.025	FALSE
Angptl3	121-127	10 s	0.313	-0.228	0.228	FALSE
Angptl3	121-127	2 min	0.310	-0.169	0.169	FALSE
Angptl3	121-127	10 min	0.293	-0.169	0.169	FALSE
Angptl3	121-127	60 min	0.329	-0.085	0.085	FALSE
Angptl3	124-131	10 s	0.326	0.073	0.073	FALSE
Angptl3	124-131	2 min	0.341	0.017	0.017	FALSE
Angptl3	124-131	10 min	0.195	0.123	0.123	FALSE
Angptl3	124-131	60 min	0.191	0.149	0.149	FALSE
Angptl3	124-133	10 s	0.185	0.106	0.106	FALSE
Angptl3	124-133	2 min	0.171	0.182	0.182	TRUE
Angptl3	124-133	10 min	0.264	0.226	0.226	FALSE
Angptl3	124-133	60 min	0.269	0.215	0.215	FALSE
Angptl3	128-133	10 s	0.117	0.032	0.032	FALSE
Angptl3	128-133	2 min	0.106	0.008	0.008	FALSE
Angptl3	128-133	10 min	0.115	0.028	0.028	FALSE
Angptl3	128-133	60 min	0.116	0.053	0.053	FALSE
Angptl3	132-137	10 s	0.077	0.031	0.031	FALSE
Angptl3	132-137	2 min	0.069	-0.034	0.034	FALSE
Angptl3	132-137	10 min	0.081	0.005	0.005	FALSE
Angptl3	132-137	60 min	0.069	0.019	0.019	FALSE
Angptl3	134-162	10 s	1.209	-0.178	0.178	FALSE
Angptl3	134-162	2 min	1.272	-0.180	0.180	FALSE
Angptl3	134-162	10 min	1.527	-0.661	0.661	FALSE
Angptl3	134-162	60 min	1.952	-0.333	0.333	FALSE
Angptl3	138-148	10 s	0.954	0.088	0.088	FALSE
Angptl3	138-148	2 min	0.139	0.013	0.013	FALSE
Angptl3	138-148	10 min	0.277	0.057	0.057	FALSE
Angptl3	138-148	60 min	0.259	0.126	0.126	FALSE
Angptl3	138-162	10 s	0.665	-0.675	0.675	TRUE
Angptl3	138-162	2 min	0.853	-0.466	0.466	FALSE

Protein	peptide	Exposure	U=ku k=2	Δ (HX)t	ABS(Δ (HX)t)	True = significant difference, reject Null (no difference)
Angptl3	138-162	60 min	0.921	-0.719	0.719	FALSE
Angptl3	138-166	10 s	0.997	-0.601	0.601	FALSE
Angptl3	138-166	2 min	1.101	-0.313	0.313	FALSE
Angptl3	138-166	10 min	1.352	-0.769	0.769	FALSE
Angptl3	138-166	60 min	1.160	-0.788	0.788	FALSE
Angptl3	138-167	10 s	0.641	-0.571	0.571	FALSE
Angptl3	138-167	2 min	0.964	-0.465	0.465	FALSE
Angptl3	138-167	10 min	0.891	-0.663	0.663	FALSE
Angptl3	138-167	60 min	1.050	-0.673	0.673	FALSE
Angptl3	140-167	10 s	0.473	-0.562	0.562	TRUE
Angptl3	140-167	2 min	0.840	-0.770	0.770	FALSE
Angptl3	140-167	10 min	0.962	-0.921	0.921	FALSE
Angptl3	140-167	60 min	0.883	-0.876	0.876	FALSE
Angptl3	145-167	10 s	0.742	-0.606	0.606	FALSE
Angptl3	145-167	2 min	1.055	-0.654	0.654	FALSE
Angptl3	145-167	10 min	0.925	-0.763	0.763	FALSE
Angptl3	145-167	60 min	1.138	-0.781	0.781	FALSE
Angptl3	146-167	10 s	0.638	-0.098	0.098	FALSE
Angptl3	146-167	2 min	0.627	-0.275	0.275	FALSE
Angptl3	146-167	10 min	0.888	-0.013	0.013	FALSE
Angptl3	146-167	60 min	0.853	-0.005	0.005	FALSE
Angptl3	149-167	10 s	0.419	-0.082	0.082	FALSE
Angptl3	149-167	2 min	0.499	-0.005	0.005	FALSE
Angptl3	149-167	10 min	0.752	0.058	0.058	FALSE
Angptl3	149-167	60 min	0.944	-0.306	0.306	FALSE
Angptl3	166-172	10 s	0.128	0.014	0.014	FALSE
Angptl3	166-172	2 min	0.157	-0.246	0.246	TRUE
Angptl3	166-172	10 min	0.230	-0.056	0.056	FALSE
Angptl3	166-172	60 min	0.198	0.044	0.044	FALSE
Angptl3	168-178	10 s	0.126	0.063	0.063	FALSE
Angptl3	168-178	2 min	0.190	0.081	0.081	FALSE
Angptl3	168-178	10 min	0.265	0.105	0.105	FALSE
Angptl3	168-178	60 min	0.409	-0.068	0.068	FALSE
Angptl3	168-184	10 s	1.022	-0.160	0.160	FALSE
Angptl3	168-184	2 min	1.218	0.015	0.015	FALSE
Angptl3	168-184	10 min	0.865	0.032	0.032	FALSE
Angptl3	168-184	60 min	0.734	-0.103	0.103	FALSE
Angptl3	168-189	10 s	0.288	0.037	0.037	FALSE
Angptl3	168-189	2 min	0.431	-0.158	0.158	FALSE
Angptl3	168-189	10 min	0.694	-0.166	0.166	FALSE
Angptl3	168-189	60 min	0.937	-0.411	0.411	FALSE
Angptl3	171-189	10 s	0.324	0.109	0.109	FALSE
Angptl3	171-189	2 min	0.275	-0.043	0.043	FALSE
Angptl3	171-189	10 min	0.576	-0.063	0.063	FALSE
Angptl3	171-189	60 min	1.250	-0.315	0.315	FALSE
Angptl3	173-189	10 s	0.399	0.081	0.081	FALSE
Angptl3	173-189	2 min	0.381	-0.065	0.065	FALSE
Angptl3	173-189	10 min	0.675	0.189	0.189	FALSE
Angptl3	173-189	60 min	0.968	0.001	0.001	FALSE
Angptl3	177-189	10 s	0.244	0.048	0.048	FALSE
Angptl3	177-189	2 min	0.294	-0.049	0.049	FALSE
Angptl3	177-189	10 min	0.561	0.055	0.055	FALSE
Angptl3	177-189	60 min	0.912	-0.059	0.059	FALSE
Angptl3	179-189	10 s	0.229	0.218	0.218	FALSE
Angptl3	179-189	2 min	0.168	0.007	0.007	FALSE
Angptl3	179-189	10 min	0.255	0.206	0.206	FALSE
Angptl3	179-189	60 min	0.290	0.012	0.012	FALSE
Angptl3	190-200	10 s	0.231	0.038	0.038	FALSE
Angptl3	190-200	2 min	0.258	0.042	0.042	FALSE
Angptl3	190-200	10 min	0.260	0.165	0.165	FALSE

Protein	peptide	Exposure	U=ku k=2	Δ (HX)t	ABS(Δ (HX)t)	True = significant difference, reject Null (no difference)
Angptl3	190-203	10 s	0.243	0.040	0.040	FALSE
Angptl3	190-203	2 min	0.262	-0.125	0.125	FALSE
Angptl3	190-203	10 min	0.480	-0.007	0.007	FALSE
Angptl3	190-203	60 min	0.487	-0.134	0.134	FALSE
Angptl3	190-205	10 s	0.083	-0.108	0.108	TRUE
Angptl3	190-205	2 min	0.260	-0.088	0.088	FALSE
Angptl3	190-205	10 min	0.309	-0.046	0.046	FALSE
Angptl3	190-205	60 min	0.554	-0.208	0.208	FALSE
Angptl3	190-216	10 s	0.828	-0.400	0.400	FALSE
Angptl3	190-216	2 min	1.071	-0.545	0.545	FALSE
Angptl3	190-216	10 min	1.238	-0.696	0.696	FALSE
Angptl3	190-216	60 min	1.145	-0.734	0.734	FALSE
Angptl3	190-219	10 s	1.221	-0.700	0.700	FALSE
Angptl3	190-219	2 min	1.762	-0.740	0.740	FALSE
Angptl3	190-219	10 min	1.264	-1.020	1.020	FALSE
Angptl3	190-219	60 min	1.257	-0.859	0.859	FALSE
Angptl3	198-205	10 s	0.262	-0.180	0.180	FALSE
Angptl3	198-205	2 min	0.452	-0.299	0.299	FALSE
Angptl3	198-205	10 min	0.449	-0.161	0.161	FALSE
Angptl3	198-205	60 min	0.446	-0.308	0.308	FALSE
Angptl3	198-216	10 s	0.650	0.267	0.267	FALSE
Angptl3	198-216	2 min	0.852	0.226	0.226	FALSE
Angptl3	198-216	10 min	0.774	-0.202	0.202	FALSE
Angptl3	198-216	60 min	1.050	-0.208	0.208	FALSE
Angptl3	204-219	10 s	1.418	-0.937	0.937	FALSE
Angptl3	204-219	2 min	1.444	-0.364	0.364	FALSE
Angptl3	204-219	10 min	1.374	-0.525	0.525	FALSE
Angptl3	204-219	60 min	1.386	-0.661	0.661	FALSE
Angptl3	204-231	10 s	1.606	-0.691	0.691	FALSE
Angptl3	204-231	2 min	1.297	-0.596	0.596	FALSE
Angptl3	204-231	10 min	1.267	-0.420	0.420	FALSE
Angptl3	204-231	60 min	0.945	-0.827	0.827	FALSE
Angptl3	217-231	10 s	0.186	-0.168	0.168	FALSE
Angptl3	217-231	2 min	0.339	-0.013	0.013	FALSE
Angptl3	217-231	10 min	0.212	-0.205	0.205	FALSE
Angptl3	217-231	60 min	0.283	-0.249	0.249	FALSE
Angptl3	232-259	10 s	1.216	-0.451	0.451	FALSE
Angptl3	232-259	2 min	1.457	-0.584	0.584	FALSE
Angptl3	232-259	10 min	1.521	-0.766	0.766	FALSE
Angptl3	232-259	60 min	1.431	-1.312	1.312	FALSE
Angptl3	253-259	10 s	0.241	-0.110	0.110	FALSE
Angptl3	253-259	2 min	0.313	-0.212	0.212	FALSE
Angptl3	253-259	10 min	0.324	-0.137	0.137	FALSE
Angptl3	253-259	60 min	0.321	-0.238	0.238	FALSE
Angptl3	260-273	10 s	0.279	0.072	0.072	FALSE
Angptl3	260-273	2 min	0.342	0.024	0.024	FALSE
Angptl3	260-273	10 min	0.406	0.009	0.009	FALSE
Angptl3	260-273	60 min	0.330	-0.078	0.078	FALSE
Angptl3	274-284	10 s	0.370	-0.079	0.079	FALSE
Angptl3	274-284	2 min	0.416	-0.119	0.119	FALSE
Angptl3	274-284	10 min	0.578	-0.053	0.053	FALSE
Angptl3	274-284	60 min	0.771	-0.152	0.152	FALSE
Angptl3	276-284	10 s	0.148	-0.033	0.033	FALSE
Angptl3	276-284	2 min	0.188	-0.052	0.052	FALSE
Angptl3	276-284	10 min	0.246	-0.033	0.033	FALSE
Angptl3	276-284	60 min	0.343	-0.068	0.068	FALSE
Angptl3	285-290	10 s	0.110	0.078	0.078	FALSE
Angptl3	285-290	2 min	0.140	-0.035	0.035	FALSE
Angptl3	285-290	10 min	0.155	-0.006	0.006	FALSE
Angptl3	285-290	60 min	0.176	0.027	0.027	FALSE

Protein	peptide	Exposure	U=ku k=2	Δ (HX)t	ABS(Δ (HX)t)	True = significant difference, reject Null (no difference)
Angptl3	310-316	2 min	0.052	-0.037	0.037	FALSE
Angptl3	310-316	10 min	0.043	0.019	0.019	FALSE
Angptl3	310-316	60 min	0.059	-0.005	0.005	FALSE
Angptl3	310-319	10 s	0.144	0.075	0.075	FALSE
Angptl3	310-319	2 min	0.134	0.085	0.085	FALSE
Angptl3	310-319	10 min	0.167	0.041	0.041	FALSE
Angptl3	310-319	60 min	0.110	0.046	0.046	FALSE
Angptl3	313-319	10 s	0.122	0.025	0.025	FALSE
Angptl3	313-319	2 min	0.087	-0.048	0.048	FALSE
Angptl3	313-319	10 min	0.108	-0.004	0.004	FALSE
Angptl3	313-319	60 min	0.099	0.027	0.027	FALSE
Angptl3	320-325	10 s	0.059	0.009	0.009	FALSE
Angptl3	320-325	2 min	0.052	-0.004	0.004	FALSE
Angptl3	320-325	10 min	0.071	0.035	0.035	FALSE
Angptl3	320-325	60 min	0.057	0.050	0.050	FALSE
Angptl3	320-328	10 s	0.082	0.019	0.019	FALSE
Angptl3	320-328	2 min	0.091	-0.010	0.010	FALSE
Angptl3	320-328	10 min	0.119	0.027	0.027	FALSE
Angptl3	320-328	60 min	0.095	0.034	0.034	FALSE
Angptl3	320-329	10 s	0.088	-0.003	0.003	FALSE
Angptl3	320-329	2 min	0.137	0.045	0.045	FALSE
Angptl3	320-329	10 min	0.112	0.000	0.000	FALSE
Angptl3	320-329	60 min	0.148	0.019	0.019	FALSE
Angptl3	320-331	10 s	0.143	0.061	0.061	FALSE
Angptl3	320-331	2 min	0.168	0.009	0.009	FALSE
Angptl3	320-331	10 min	0.090	-0.093	0.093	TRUE
Angptl3	320-331	60 min	0.095	-0.078	0.078	FALSE
Angptl3	322-332	10 s	0.618	0.382	0.382	FALSE
Angptl3	322-332	2 min	0.567	0.271	0.271	FALSE
Angptl3	322-332	10 min	0.788	0.113	0.113	FALSE
Angptl3	322-332	60 min	0.838	-0.075	0.075	FALSE
Angptl3	333-342	10 s	0.143	0.069	0.069	FALSE
Angptl3	333-342	2 min	0.175	-0.021	0.021	FALSE
Angptl3	333-342	10 min	0.293	-0.125	0.125	FALSE
Angptl3	333-342	60 min	0.272	-0.055	0.055	FALSE
Angptl3	335-340	10 s	0.145	0.039	0.039	FALSE
Angptl3	335-340	2 min	0.106	-0.016	0.016	FALSE
Angptl3	335-340	10 min	0.135	-0.050	0.050	FALSE
Angptl3	335-340	60 min	0.132	-0.041	0.041	FALSE
Angptl3	335-342	10 s	0.092	0.003	0.003	FALSE
Angptl3	335-342	2 min	0.165	0.069	0.069	FALSE
Angptl3	335-342	10 min	0.185	0.043	0.043	FALSE
Angptl3	335-342	60 min	0.214	-0.094	0.094	FALSE
Angptl3	335-346	10 s	0.161	0.001	0.001	FALSE
Angptl3	335-346	2 min	0.205	0.034	0.034	FALSE
Angptl3	335-346	10 min	0.237	0.047	0.047	FALSE
Angptl3	335-346	60 min	0.331	-0.072	0.072	FALSE
Angptl3	336-342	10 s	0.168	0.100	0.100	FALSE
Angptl3	336-342	2 min	0.269	0.206	0.206	FALSE
Angptl3	336-342	10 min	0.281	0.135	0.135	FALSE
Angptl3	336-342	60 min	0.301	-0.074	0.074	FALSE
Angptl3	343-349	10 s	0.134	0.040	0.040	FALSE
Angptl3	343-349	2 min	0.176	-0.047	0.047	FALSE
Angptl3	343-349	10 min	0.231	-0.014	0.014	FALSE
Angptl3	343-349	60 min	0.340	0.054	0.054	FALSE
Angptl3	363-377	10 s	0.204	-0.032	0.032	FALSE
Angptl3	363-377	2 min	0.415	-0.046	0.046	FALSE
Angptl3	363-377	10 min	0.482	-0.049	0.049	FALSE
Angptl3	363-377	60 min	0.552	-0.188	0.188	FALSE
Angptl3	363-378	10 s	1.175	-0.598	0.598	FALSE

Protein	peptide	Exposure	U=ku k=2	Δ (HX)t	ABS(Δ (HX)t)	True = significant difference, reject Null (no difference)
Angptl3	363-378	10 min	0.536	-0.601	0.601	TRUE
Angptl3	363-378	60 min	0.628	-0.298	0.298	FALSE
Angptl3	363-379	10 s	0.256	-0.115	0.115	FALSE
Angptl3	363-379	2 min	0.348	-0.195	0.195	FALSE
Angptl3	363-379	10 min	0.447	-0.267	0.267	FALSE
Angptl3	363-379	60 min	0.378	-0.284	0.284	FALSE
Angptl3	363-381	10 s	1.071	-0.089	0.089	FALSE
Angptl3	363-381	2 min	1.072	-0.267	0.267	FALSE
Angptl3	363-381	10 min	1.225	-0.225	0.225	FALSE
Angptl3	363-381	60 min	1.100	-0.104	0.104	FALSE
Angptl3	365-377	10 s	0.077	-0.030	0.030	FALSE
Angptl3	365-377	2 min	0.325	0.030	0.030	FALSE
Angptl3	365-377	10 min	0.308	-0.032	0.032	FALSE
Angptl3	365-377	60 min	0.328	-0.188	0.188	FALSE
Angptl3	365-378	10 s	0.167	-0.182	0.182	TRUE
Angptl3	365-378	2 min	0.459	-0.130	0.130	FALSE
Angptl3	365-378	10 min	0.440	-0.160	0.160	FALSE
Angptl3	365-378	60 min	0.510	-0.280	0.280	FALSE
Angptl3	365-379	10 s	0.149	-0.050	0.050	FALSE
Angptl3	365-379	2 min	0.343	-0.052	0.052	FALSE
Angptl3	365-379	10 min	0.385	-0.085	0.085	FALSE
Angptl3	365-379	60 min	0.471	-0.281	0.281	FALSE
Angptl3	365-381	10 s	1.618	-0.313	0.313	FALSE
Angptl3	365-381	2 min	0.590	-0.107	0.107	FALSE
Angptl3	365-381	10 min	0.723	0.048	0.048	FALSE
Angptl3	365-381	60 min	0.863	-0.112	0.112	FALSE
Angptl3	378-386	10 s	0.207	0.046	0.046	FALSE
Angptl3	378-386	2 min	0.216	-0.086	0.086	FALSE
Angptl3	378-386	10 min	0.166	-0.044	0.044	FALSE
Angptl3	378-386	60 min	0.171	-0.016	0.016	FALSE
Angptl3	379-386	10 s	0.111	-0.054	0.054	FALSE
Angptl3	379-386	2 min	0.105	-0.141	0.141	TRUE
Angptl3	379-386	10 min	0.088	-0.160	0.160	TRUE
Angptl3	379-386	60 min	0.092	-0.076	0.076	FALSE
Angptl3	380-388	10 s	0.125	0.076	0.076	FALSE
Angptl3	380-388	2 min	0.229	0.011	0.011	FALSE
Angptl3	380-388	10 min	0.283	0.139	0.139	FALSE
Angptl3	380-388	60 min	0.344	-0.049	0.049	FALSE
Angptl3	389-407	10 s	0.446	0.213	0.213	FALSE
Angptl3	389-407	2 min	0.520	0.070	0.070	FALSE
Angptl3	389-407	10 min	0.504	-0.031	0.031	FALSE
Angptl3	389-407	60 min	0.500	0.043	0.043	FALSE
Angptl3	408-413	10 s	0.152	-0.143	0.143	FALSE
Angptl3	408-413	2 min	0.138	-0.184	0.184	TRUE
Angptl3	408-413	10 min	0.151	-0.217	0.217	TRUE
Angptl3	408-413	60 min	0.168	-0.173	0.173	TRUE
Angptl3	408-416	10 s	0.124	0.000	0.000	FALSE
Angptl3	408-416	2 min	0.188	-0.131	0.131	FALSE
Angptl3	408-416	10 min	0.238	0.007	0.007	FALSE
Angptl3	408-416	60 min	0.237	-0.084	0.084	FALSE
Angptl3	408-441	10 s	1.010	-0.494	0.494	FALSE
Angptl3	408-441	2 min	1.203	-0.214	0.214	FALSE
Angptl3	408-441	10 min	1.480	-0.358	0.358	FALSE
Angptl3	408-441	60 min	1.381	-0.978	0.978	FALSE
Angptl3	417-441	10 s	0.469	0.047	0.047	FALSE
Angptl3	417-441	2 min	0.348	0.364	0.364	TRUE
Angptl3	417-441	10 min	0.424	0.222	0.222	FALSE
Angptl3	417-441	60 min	0.737	-0.115	0.115	FALSE
Angptl3	417-445	10 s	1.336	-0.088	0.088	FALSE
Angptl3	417-445	2 min	1.391	-0.129	0.129	FALSE

Protein	peptide	Exposure	U=ku k=2	Δ (HX)t	ABS(Δ (HX)t)	True = significant difference, reject Null (no difference)
Angptl3	417-445	60 min	1.111	-0.397	0.397	FALSE
Angptl3	433-441	10 s	0.219	-0.098	0.098	FALSE
Angptl3	433-441	2 min	0.299	0.025	0.025	FALSE
Angptl3	433-441	10 min	0.278	-0.057	0.057	FALSE
Angptl3	433-441	60 min	0.317	-0.151	0.151	FALSE
Angptl3	433-445	10 s	0.279	-0.106	0.106	FALSE
Angptl3	433-445	2 min	0.403	-0.129	0.129	FALSE
Angptl3	433-445	10 min	0.386	-0.181	0.181	FALSE
Angptl3	433-445	60 min	0.454	-0.245	0.245	FALSE
Angptl3	442-450	10 s	0.121	-0.025	0.025	FALSE
Angptl3	442-450	2 min	0.110	-0.016	0.016	FALSE
Angptl3	442-450	10 min	0.139	0.046	0.046	FALSE
Angptl3	442-450	60 min	0.120	0.056	0.056	FALSE
Angptl3	443-450	10 s	0.088	0.024	0.024	FALSE
Angptl3	443-450	2 min	0.090	-0.085	0.085	FALSE
Angptl3	443-450	10 min	0.112	-0.037	0.037	FALSE
Angptl3	443-450	60 min	0.041	-0.123	0.123	TRUE
Angptl3	446-463	10 s	1.317	-1.592	1.592	TRUE
Angptl3	446-463	2 min	1.043	-1.351	1.351	TRUE
Angptl3	446-463	10 min	0.965	-0.721	0.721	FALSE
Angptl3	446-463	60 min	0.467	-0.271	0.271	FALSE
Angptl3	449-461	10 s	0.379	-0.068	0.068	FALSE
Angptl3	449-461	2 min	0.466	-0.083	0.083	FALSE
Angptl3	449-461	10 min	0.452	-0.213	0.213	FALSE
Angptl3	449-461	60 min	0.388	-0.168	0.168	FALSE
Angptl3	451-459	10 s	0.448	-0.042	0.042	FALSE
Angptl3	451-459	2 min	0.495	-0.068	0.068	FALSE
Angptl3	451-459	10 min	0.285	-0.228	0.228	FALSE
Angptl3	451-459	60 min	0.467	-0.068	0.068	FALSE
Angptl3	451-461	10 s	0.266	-0.182	0.182	FALSE
Angptl3	451-461	2 min	0.295	-0.095	0.095	FALSE
Angptl3	451-461	10 min	0.299	-0.138	0.138	FALSE
Angptl3	451-461	60 min	0.294	-0.249	0.249	FALSE
Angptl3	451-463	10 s	0.394	-0.223	0.223	FALSE
Angptl3	451-463	2 min	0.453	0.066	0.066	FALSE
Angptl3	451-463	10 min	0.445	-0.156	0.156	FALSE
Angptl3	451-463	60 min	0.440	-0.283	0.283	FALSE
Angptl3	462-471	10 s	0.474	-0.145	0.145	FALSE
Angptl3	462-471	2 min	0.528	-0.061	0.061	FALSE
Angptl3	462-471	10 min	0.491	0.065	0.065	FALSE
Angptl3	462-471	60 min	0.548	-0.226	0.226	FALSE
Angptl3	464-471	10 s	0.280	-0.052	0.052	FALSE
Angptl3	464-471	2 min	0.338	-0.073	0.073	FALSE
Angptl3	464-471	10 min	0.340	-0.049	0.049	FALSE
Angptl3	464-471	60 min	0.306	-0.109	0.109	FALSE
Angptl8	36-49	10 s	0.300	2.033	2.033	TRUE
Angptl8	36-49	2 min	0.335	2.735	2.735	TRUE
Angptl8	36-49	10 min	0.302	2.668	2.668	TRUE
Angptl8	36-49	60 min	0.338	2.087	2.087	TRUE
Angptl8	38-49	10 s	0.087	1.778	1.778	TRUE
Angptl8	38-49	2 min	0.200	2.388	2.388	TRUE
Angptl8	38-49	10 min	0.143	2.402	2.402	TRUE
Angptl8	38-49	60 min	0.232	1.624	1.624	TRUE
Angptl8	39-49	10 s	0.050	1.947	1.947	TRUE
Angptl8	39-49	2 min	0.221	2.636	2.636	TRUE
Angptl8	39-49	10 min	0.192	2.640	2.640	TRUE
Angptl8	39-49	60 min	0.249	1.737	1.737	TRUE
Angptl8	50-62	10 s	0.287	0.664	0.664	TRUE
Angptl8	50-62	2 min	0.647	0.744	0.744	TRUE
Angptl8	50-62	10 min	0.746	0.519	0.519	FALSE

Protein	peptide	Exposure	U=ku k=2	Δ (HX)t	ABS(Δ (HX)t)	True = significant difference, reject Null (no difference)
Angptl8	50-64	10 s	0.114	0.955	0.955	TRUE
Angptl8	50-64	2 min	0.277	1.088	1.088	TRUE
Angptl8	50-64	10 min	0.306	1.185	1.185	TRUE
Angptl8	50-64	60 min	0.348	0.645	0.645	TRUE
Angptl8	53-64	10 s	1.323	0.465	0.465	FALSE
Angptl8	53-64	2 min	1.242	0.931	0.931	FALSE
Angptl8	53-64	10 min	0.886	0.834	0.834	FALSE
Angptl8	53-64	60 min	1.428	0.072	0.072	FALSE
Angptl8	65-76	10 s	0.057	0.378	0.378	TRUE
Angptl8	65-76	2 min	0.162	0.584	0.584	TRUE
Angptl8	65-76	10 min	0.178	0.381	0.381	TRUE
Angptl8	65-76	60 min	0.193	0.127	0.127	FALSE
Angptl8	77-91	10 s	0.202	0.063	0.063	FALSE
Angptl8	77-91	2 min	0.167	-0.012	0.012	FALSE
Angptl8	77-91	10 min	0.352	0.181	0.181	FALSE
Angptl8	77-91	60 min	0.471	0.076	0.076	FALSE
Angptl8	77-94	10 s	0.331	0.134	0.134	FALSE
Angptl8	77-94	2 min	0.263	-0.202	0.202	FALSE
Angptl8	77-94	10 min	0.413	0.425	0.425	TRUE
Angptl8	77-94	60 min	0.355	0.212	0.212	FALSE
Angptl8	77-95	10 s	0.278	0.163	0.163	FALSE
Angptl8	77-95	2 min	0.191	0.088	0.088	FALSE
Angptl8	77-95	10 min	0.418	0.261	0.261	FALSE
Angptl8	77-95	60 min	0.461	0.253	0.253	FALSE
Angptl8	77-103	10 s	0.594	0.221	0.221	FALSE
Angptl8	77-103	2 min	0.184	-0.061	0.061	FALSE
Angptl8	77-103	10 min	0.405	0.371	0.371	FALSE
Angptl8	77-103	60 min	0.689	0.231	0.231	FALSE
Angptl8	81-91	10 s	0.086	0.106	0.106	TRUE
Angptl8	81-91	2 min	0.205	-0.061	0.061	FALSE
Angptl8	81-91	10 min	0.250	0.073	0.073	FALSE
Angptl8	81-91	60 min	0.629	0.072	0.072	FALSE
Angptl8	81-94	10 s	0.163	-0.017	0.017	FALSE
Angptl8	81-94	2 min	0.258	-0.075	0.075	FALSE
Angptl8	81-94	10 min	0.361	0.065	0.065	FALSE
Angptl8	81-94	60 min	0.686	-0.181	0.181	FALSE
Angptl8	81-95	10 s	0.302	0.207	0.207	FALSE
Angptl8	81-95	2 min	0.251	0.007	0.007	FALSE
Angptl8	81-95	10 min	0.389	0.244	0.244	FALSE
Angptl8	81-95	60 min	0.758	-0.027	0.027	FALSE
Angptl8	92-103	10 s	0.118	0.229	0.229	TRUE
Angptl8	92-103	2 min	0.084	0.194	0.194	TRUE
Angptl8	92-103	10 min	0.188	0.287	0.287	TRUE
Angptl8	92-103	60 min	0.114	0.267	0.267	TRUE
Angptl8	96-103	10 s	0.122	0.056	0.056	FALSE
Angptl8	96-103	2 min	0.148	-0.013	0.013	FALSE
Angptl8	96-103	10 min	0.277	0.053	0.053	FALSE
Angptl8	96-103	60 min	0.517	0.032	0.032	FALSE
Angptl8	104-109	10 s	0.077	0.023	0.023	FALSE
Angptl8	104-109	2 min	0.103	0.040	0.040	FALSE
Angptl8	104-109	10 min	0.056	-0.024	0.024	FALSE
Angptl8	104-109	60 min	0.065	-0.030	0.030	FALSE
Angptl8	104-124	10 s	0.442	0.241	0.241	FALSE
Angptl8	104-124	2 min	0.828	0.092	0.092	FALSE
Angptl8	104-124	10 min	0.555	-0.108	0.108	FALSE
Angptl8	104-124	60 min	0.616	0.147	0.147	FALSE
Angptl8	106-128	10 s	0.465	0.175	0.175	FALSE
Angptl8	106-128	2 min	0.217	0.012	0.012	FALSE
Angptl8	106-128	10 min	0.246	-0.409	0.409	TRUE
Angptl8	106-128	60 min	0.348	-0.539	0.539	TRUE

Protein	peptide	Exposure	U=ku k=2	Δ (HX)t	ABS(Δ (HX)t)	True = significant difference, reject Null (no difference)
Angptl8	108-124	2 min	0.194	0.141	0.141	FALSE
Angptl8	108-124	10 min	0.417	0.047	0.047	FALSE
Angptl8	108-124	60 min	0.609	0.051	0.051	FALSE
Angptl8	108-127	10 s	0.768	0.317	0.317	FALSE
Angptl8	108-127	2 min	0.286	0.044	0.044	FALSE
Angptl8	108-127	10 min	0.313	-0.362	0.362	TRUE
Angptl8	108-127	60 min	1.782	-0.132	0.132	FALSE
Angptl8	108-128	10 s	0.213	0.098	0.098	FALSE
Angptl8	108-128	2 min	0.101	-0.041	0.041	FALSE
Angptl8	108-128	10 min	0.293	-0.117	0.117	FALSE
Angptl8	108-128	60 min	0.558	-0.437	0.437	FALSE
Angptl8	108-133	10 s	0.601	0.138	0.138	FALSE
Angptl8	108-133	2 min	0.138	-0.183	0.183	TRUE
Angptl8	108-133	10 min	0.311	-0.127	0.127	FALSE
Angptl8	108-133	60 min	0.485	-0.443	0.443	FALSE
Angptl8	111-128	10 s	0.347	0.145	0.145	FALSE
Angptl8	111-128	2 min	0.301	0.028	0.028	FALSE
Angptl8	111-128	10 min	0.510	0.067	0.067	FALSE
Angptl8	111-128	60 min	0.765	0.473	0.473	FALSE
Angptl8	111-133	10 s	0.243	0.199	0.199	FALSE
Angptl8	111-133	2 min	0.281	-0.124	0.124	FALSE
Angptl8	111-133	10 min	0.634	-0.165	0.165	FALSE
Angptl8	111-133	60 min	0.663	-0.356	0.356	FALSE
Angptl8	125-133	10 s	0.071	0.022	0.022	FALSE
Angptl8	125-133	2 min	0.044	-0.046	0.046	TRUE
Angptl8	125-133	10 min	0.058	-0.048	0.048	FALSE
Angptl8	125-133	60 min	0.174	-0.175	0.175	TRUE
Angptl8	126-133	10 s	0.041	0.025	0.025	FALSE
Angptl8	126-133	2 min	0.055	0.015	0.015	FALSE
Angptl8	126-133	10 min	0.045	0.039	0.039	FALSE
Angptl8	126-133	60 min	0.205	-0.112	0.112	FALSE
Angptl8	139-148	10 s	0.510	-0.191	0.191	FALSE
Angptl8	139-148	2 min	0.499	-0.275	0.275	FALSE
Angptl8	139-148	10 min	0.576	-0.226	0.226	FALSE
Angptl8	139-148	60 min	0.494	-0.219	0.219	FALSE
Angptl8	150-163	10 s	0.526	0.185	0.185	FALSE
Angptl8	150-163	2 min	0.599	-0.154	0.154	FALSE
Angptl8	150-163	10 min	0.598	0.282	0.282	FALSE
Angptl8	150-163	60 min	0.645	0.060	0.060	FALSE
Angptl8	153-163	10 s	0.242	0.023	0.023	FALSE
Angptl8	153-163	2 min	0.353	0.009	0.009	FALSE
Angptl8	153-163	10 min	0.450	-0.005	0.005	FALSE
Angptl8	153-163	60 min	0.608	-0.105	0.105	FALSE
Angptl8	153-177	10 s	0.406	-0.029	0.029	FALSE
Angptl8	153-177	2 min	0.322	-0.118	0.118	FALSE
Angptl8	153-177	10 min	0.728	-0.237	0.237	FALSE
Angptl8	153-177	60 min	0.692	-0.558	0.558	FALSE
Angptl8	164-177	10 s	0.166	0.055	0.055	FALSE
Angptl8	164-177	2 min	0.157	-0.106	0.106	FALSE
Angptl8	164-177	10 min	0.643	-0.213	0.213	FALSE
Angptl8	164-177	60 min	0.309	-0.117	0.117	FALSE
Angptl8	166-177	10 s	0.330	-0.177	0.177	FALSE
Angptl8	166-177	2 min	0.404	-0.131	0.131	FALSE
Angptl8	166-177	10 min	0.429	-0.172	0.172	FALSE
Angptl8	166-177	60 min	0.779	-0.149	0.149	FALSE
Angptl8	176-184	10 s	0.273	0.123	0.123	FALSE
Angptl8	176-184	2 min	0.247	0.091	0.091	FALSE
Angptl8	176-184	10 min	0.227	0.051	0.051	FALSE
Angptl8	176-184	60 min	0.175	0.034	0.034	FALSE
Angptl8	177-190	10 s	0.190	-0.141	0.141	FALSE

Protein	peptide	Exposure	U=ku k=2	Δ (HX)t	ABS(Δ (HX)t)	True = significant difference, reject Null (no difference)
Angptl8	177-190	10 min	0.366	-0.449	0.449	TRUE
Angptl8	177-190	60 min	0.561	-0.354	0.354	FALSE
Angptl8	178-190	10 s	0.061	-0.008	0.008	FALSE
Angptl8	178-190	2 min	0.231	-0.184	0.184	FALSE
Angptl8	178-190	10 min	0.263	-0.191	0.191	FALSE
Angptl8	178-190	60 min	0.367	-0.216	0.216	FALSE
Angptl8	178-191	10 s	0.201	0.147	0.147	FALSE
Angptl8	178-191	2 min	0.319	-0.125	0.125	FALSE
Angptl8	178-191	10 min	0.313	-0.134	0.134	FALSE
Angptl8	178-191	60 min	0.328	-0.143	0.143	FALSE
Angptl8	178-198	10 s	0.323	-0.218	0.218	FALSE
Angptl8	178-198	2 min	0.591	-0.371	0.371	FALSE
Angptl8	178-198	10 min	0.586	-0.353	0.353	FALSE
Angptl8	178-198	60 min	0.647	-0.372	0.372	FALSE
Angptl8	185-198	10 s	0.247	-0.141	0.141	FALSE
Angptl8	185-198	2 min	0.483	-0.176	0.176	FALSE
Angptl8	185-198	10 min	0.504	-0.260	0.260	FALSE
Angptl8	185-198	60 min	0.512	-0.290	0.290	FALSE

Table S4: Differential HX, combined uncertainty, and significance testing for individual HX times for ANGPTL3 and ANGPTL8 peptides in ANGPTL3/8 + ApoA5 compared to unbound ANGPTL3/8

Protein	peptide	Exposure	U=ku k=2	Δ (HX)t	ABS(T)	True = significant difference, reject Null (no difference)
Angptl3	17-36	10 s	0.658	-0.722	0.722	TRUE
Angptl3	17-36	2 min	0.812	-0.374	0.374	FALSE
Angptl3	17-36	10 min	0.882	-0.454	0.454	FALSE
Angptl3	17-36	60 min	0.699	-0.499	0.499	FALSE
Angptl3	17-40	10 s	0.678	-0.438	0.438	FALSE
Angptl3	17-40	2 min	0.775	-0.185	0.185	FALSE
Angptl3	17-40	10 min	0.957	-0.157	0.157	FALSE
Angptl3	17-40	60 min	0.705	-0.268	0.268	FALSE
Angptl3	17-46	10 s	0.708	0.294	0.294	FALSE
Angptl3	17-46	2 min	0.877	0.591	0.591	FALSE
Angptl3	17-46	10 min	0.806	0.282	0.282	FALSE
Angptl3	17-46	60 min	0.604	-0.128	0.128	FALSE
Angptl3	37-42	10 s	0.071	-0.047	0.047	FALSE
Angptl3	37-42	2 min	0.115	-0.105	0.105	FALSE
Angptl3	37-42	10 min	0.118	0.042	0.042	FALSE
Angptl3	37-42	60 min	0.086	-0.002	0.002	FALSE
Angptl3	37-46	10 s	0.119	0.764	0.764	TRUE
Angptl3	37-46	2 min	0.217	0.753	0.753	TRUE
Angptl3	37-46	10 min	0.119	0.454	0.454	TRUE
Angptl3	37-46	60 min	0.168	0.140	0.140	FALSE
Angptl3	41-46	10 s	0.088	0.781	0.781	TRUE
Angptl3	41-46	2 min	0.085	0.705	0.705	TRUE
Angptl3	41-46	10 min	0.087	0.317	0.317	TRUE
Angptl3	41-46	60 min	0.105	0.063	0.063	FALSE
Angptl3	45-58	10 s	0.170	0.901	0.901	TRUE
Angptl3	45-58	2 min	0.166	1.317	1.317	TRUE
Angptl3	45-58	10 min	0.299	1.353	1.353	TRUE
Angptl3	45-58	60 min	0.225	0.617	0.617	TRUE
Angptl3	45-59	10 s	0.228	0.960	0.960	TRUE
Angptl3	45-59	2 min	0.209	1.457	1.457	TRUE
Angptl3	45-59	10 min	0.643	1.780	1.780	TRUE
Angptl3	45-59	60 min	0.404	0.702	0.702	TRUE
Angptl3	45-60	10 s	0.150	0.813	0.813	TRUE
Angptl3	45-60	2 min	0.134	1.308	1.308	TRUE
Angptl3	45-60	10 min	0.091	1.486	1.486	TRUE
Angptl3	45-60	60 min	0.339	0.820	0.820	TRUE
Angptl3	47-53	10 s	0.071	0.440	0.440	TRUE
Angptl3	47-53	2 min	0.067	0.813	0.813	TRUE
Angptl3	47-53	10 min	0.124	0.924	0.924	TRUE
Angptl3	47-53	60 min	0.096	0.485	0.485	TRUE
Angptl3	47-58	10 s	0.134	0.713	0.713	TRUE
Angptl3	47-58	2 min	0.144	1.106	1.106	TRUE
Angptl3	47-58	10 min	0.198	1.363	1.363	TRUE
Angptl3	47-58	60 min	0.222	0.647	0.647	TRUE
Angptl3	47-59	10 s	0.123	0.599	0.599	TRUE
Angptl3	47-59	2 min	0.134	1.020	1.020	TRUE
Angptl3	47-59	10 min	0.202	1.261	1.261	TRUE
Angptl3	47-59	60 min	0.231	0.624	0.624	TRUE
Angptl3	47-60	10 s	0.137	0.600	0.600	TRUE
Angptl3	47-60	2 min	0.143	1.014	1.014	TRUE
Angptl3	47-60	10 min	0.230	1.213	1.213	TRUE
Angptl3	47-60	60 min	0.231	0.677	0.677	TRUE
Angptl3	47-74	10 s	0.528	1.035	1.035	TRUE
Angptl3	47-74	2 min	0.673	1.547	1.547	TRUE
Angptl3	47-74	10 min	0.922	2.522	2.522	TRUE
Angptl3	47-74	60 min	1.277	1.882	1.882	TRUE

Protein	peptide	Exposure	U=ku k=2	Δ (HX)t	ABS(T)	True = significant difference, reject Null (no difference)
Angptl3	47-75	2 min	0.839	1.493	1.493	TRUE
Angptl3	47-75	10 min	1.321	2.425	2.425	TRUE
Angptl3	47-75	60 min	2.687	1.703	1.703	FALSE
Angptl3	47-76	10 s	1.460	1.040	1.040	FALSE
Angptl3	47-76	2 min	2.577	1.446	1.446	FALSE
Angptl3	47-76	10 min	3.195	2.394	2.394	FALSE
Angptl3	47-76	60 min	2.647	1.711	1.711	FALSE
Angptl3	47-78	10 s	0.442	1.179	1.179	TRUE
Angptl3	47-78	2 min	0.633	1.557	1.557	TRUE
Angptl3	47-78	10 min	0.987	2.361	2.361	TRUE
Angptl3	47-78	60 min	1.170	1.876	1.876	TRUE
Angptl3	51-58	10 s	0.119	0.270	0.270	TRUE
Angptl3	51-58	2 min	0.201	0.454	0.454	TRUE
Angptl3	51-58	10 min	0.374	0.634	0.634	TRUE
Angptl3	51-58	60 min	0.451	0.349	0.349	FALSE
Angptl3	59-75	10 s	0.514	0.420	0.420	FALSE
Angptl3	59-75	2 min	1.097	0.275	0.275	FALSE
Angptl3	59-75	10 min	0.946	0.566	0.566	FALSE
Angptl3	59-75	60 min	1.369	0.118	0.118	FALSE
Angptl3	59-78	10 s	0.274	0.418	0.418	TRUE
Angptl3	59-78	2 min	0.548	0.393	0.393	FALSE
Angptl3	59-78	10 min	1.178	1.086	1.086	FALSE
Angptl3	59-78	60 min	1.123	1.236	1.236	TRUE
Angptl3	60-74	10 s	0.163	0.309	0.309	TRUE
Angptl3	60-74	2 min	0.167	0.365	0.365	TRUE
Angptl3	60-74	10 min	0.272	0.833	0.833	TRUE
Angptl3	60-74	60 min	0.396	1.018	1.018	TRUE
Angptl3	60-76	10 s	0.302	0.327	0.327	TRUE
Angptl3	60-76	2 min	0.405	0.320	0.320	FALSE
Angptl3	60-76	10 min	0.507	0.919	0.919	TRUE
Angptl3	60-76	60 min	0.573	0.912	0.912	TRUE
Angptl3	60-78	10 s	0.418	0.261	0.261	FALSE
Angptl3	60-78	2 min	0.507	0.243	0.243	FALSE
Angptl3	60-78	10 min	0.469	0.920	0.920	TRUE
Angptl3	60-78	60 min	0.878	1.197	1.197	TRUE
Angptl3	61-74	10 s	0.383	0.267	0.267	FALSE
Angptl3	61-74	2 min	0.501	0.402	0.402	FALSE
Angptl3	61-74	10 min	0.678	0.712	0.712	TRUE
Angptl3	61-74	60 min	1.026	0.872	0.872	FALSE
Angptl3	61-75	10 s	0.243	0.133	0.133	FALSE
Angptl3	61-75	2 min	0.320	0.232	0.232	FALSE
Angptl3	61-75	10 min	0.356	0.795	0.795	TRUE
Angptl3	61-75	60 min	0.555	1.464	1.464	TRUE
Angptl3	61-78	10 s	0.319	0.163	0.163	FALSE
Angptl3	61-78	2 min	0.519	0.093	0.093	FALSE
Angptl3	61-78	10 min	0.640	0.750	0.750	TRUE
Angptl3	61-78	60 min	0.935	0.377	0.377	FALSE
Angptl3	75-80	10 s	0.083	-0.083	0.083	TRUE
Angptl3	75-80	2 min	0.133	-0.081	0.081	FALSE
Angptl3	75-80	10 min	0.140	-0.045	0.045	FALSE
Angptl3	75-80	60 min	0.223	-0.183	0.183	FALSE
Angptl3	75-82	10 s	0.392	0.126	0.126	FALSE
Angptl3	75-82	2 min	0.675	-0.007	0.007	FALSE
Angptl3	75-82	10 min	0.847	0.141	0.141	FALSE
Angptl3	75-82	60 min	1.125	-0.007	0.007	FALSE
Angptl3	76-82	10 s	0.161	0.083	0.083	FALSE
Angptl3	76-82	2 min	0.236	-0.027	0.027	FALSE
Angptl3	76-82	10 min	0.299	0.046	0.046	FALSE
Angptl3	76-82	60 min	0.426	-0.017	0.017	FALSE
Angptl3	79-85	10 s	0.092	0.109	0.109	TRUE

Protein	peptide	Exposure	U=ku k=2	Δ (HX)t	ABS(T)	True = significant difference, reject Null (no difference)
Angptl3	79-85	10 min	0.081	0.117	0.117	TRUE
Angptl3	79-85	60 min	0.034	0.064	0.064	TRUE
Angptl3	86-93	10 s	0.212	0.075	0.075	FALSE
Angptl3	86-93	2 min	0.182	-0.046	0.046	FALSE
Angptl3	86-93	10 min	0.184	0.127	0.127	FALSE
Angptl3	86-93	60 min	0.161	0.184	0.184	TRUE
Angptl3	86-97	10 s	0.183	0.148	0.148	FALSE
Angptl3	86-97	2 min	0.146	0.039	0.039	FALSE
Angptl3	86-97	10 min	0.238	0.269	0.269	TRUE
Angptl3	86-97	60 min	0.256	0.197	0.197	FALSE
Angptl3	86-106	10 s	0.300	0.266	0.266	FALSE
Angptl3	86-106	2 min	0.503	-0.042	0.042	FALSE
Angptl3	86-106	10 min	0.704	0.229	0.229	FALSE
Angptl3	86-106	60 min	1.219	0.159	0.159	FALSE
Angptl3	86-109	10 s	0.383	0.430	0.430	TRUE
Angptl3	86-109	2 min	0.509	0.035	0.035	FALSE
Angptl3	86-109	10 min	0.548	0.452	0.452	FALSE
Angptl3	86-109	60 min	1.002	0.287	0.287	FALSE
Angptl3	88-97	10 s	0.251	0.419	0.419	TRUE
Angptl3	88-97	2 min	0.257	0.207	0.207	FALSE
Angptl3	88-97	10 min	0.401	0.181	0.181	FALSE
Angptl3	88-97	60 min	0.776	-0.063	0.063	FALSE
Angptl3	98-109	10 s	0.136	0.126	0.126	FALSE
Angptl3	98-109	2 min	0.216	0.048	0.048	FALSE
Angptl3	98-109	10 min	0.366	0.101	0.101	FALSE
Angptl3	98-109	60 min	0.577	0.042	0.042	FALSE
Angptl3	99-109	10 s	0.072	0.108	0.108	TRUE
Angptl3	99-109	2 min	0.150	0.067	0.067	FALSE
Angptl3	99-109	10 min	0.309	0.089	0.089	FALSE
Angptl3	99-109	60 min	0.434	-0.009	0.009	FALSE
Angptl3	121-127	10 s	0.223	-0.045	0.045	FALSE
Angptl3	121-127	2 min	0.257	0.069	0.069	FALSE
Angptl3	121-127	10 min	0.297	0.080	0.080	FALSE
Angptl3	121-127	60 min	0.262	0.229	0.229	FALSE
Angptl3	124-131	10 s	0.315	0.092	0.092	FALSE
Angptl3	124-131	2 min	0.422	0.113	0.113	FALSE
Angptl3	124-131	10 min	0.187	0.153	0.153	FALSE
Angptl3	124-131	60 min	0.286	0.065	0.065	FALSE
Angptl3	124-133	10 s	0.163	0.188	0.188	TRUE
Angptl3	124-133	2 min	0.187	0.105	0.105	FALSE
Angptl3	124-133	10 min	0.281	0.216	0.216	FALSE
Angptl3	124-133	60 min	0.227	0.094	0.094	FALSE
Angptl3	128-133	10 s	0.099	0.111	0.111	TRUE
Angptl3	128-133	2 min	0.104	0.039	0.039	FALSE
Angptl3	128-133	10 min	0.116	0.066	0.066	FALSE
Angptl3	128-133	60 min	0.122	0.066	0.066	FALSE
Angptl3	132-137	10 s	0.083	0.109	0.109	TRUE
Angptl3	132-137	2 min	0.059	0.023	0.023	FALSE
Angptl3	132-137	10 min	0.088	0.054	0.054	FALSE
Angptl3	132-137	60 min	0.070	0.036	0.036	FALSE
Angptl3	134-162	10 s	1.835	-0.065	0.065	FALSE
Angptl3	134-162	2 min	1.421	-0.052	0.052	FALSE
Angptl3	134-162	10 min	1.984	-0.039	0.039	FALSE
Angptl3	134-162	60 min	1.994	-0.055	0.055	FALSE
Angptl3	138-148	10 s	0.952	0.080	0.080	FALSE
Angptl3	138-148	2 min	0.135	0.012	0.012	FALSE
Angptl3	138-148	10 min	0.288	0.054	0.054	FALSE
Angptl3	138-148	60 min	0.236	0.085	0.085	FALSE
Angptl3	138-162	10 s	0.692	-0.409	0.409	FALSE
Angptl3	138-162	2 min	0.970	-0.184	0.184	FALSE

Protein	peptide	Exposure	U=ku k=2	Δ (HX)t	ABS(T)	True = significant difference, reject Null (no difference)
Angptl3	138-162	60 min	0.690	-0.616	0.616	FALSE
Angptl3	138-166	10 s	0.838	-0.245	0.245	FALSE
Angptl3	138-166	2 min	0.977	-0.163	0.163	FALSE
Angptl3	138-166	10 min	1.835	-0.519	0.519	FALSE
Angptl3	138-166	60 min	1.083	-0.602	0.602	FALSE
Angptl3	138-167	10 s	0.534	-0.412	0.412	FALSE
Angptl3	138-167	2 min	0.689	-0.363	0.363	FALSE
Angptl3	138-167	10 min	1.593	-0.195	0.195	FALSE
Angptl3	138-167	60 min	0.881	-0.652	0.652	FALSE
Angptl3	140-167	10 s	0.406	-0.340	0.340	FALSE
Angptl3	140-167	2 min	2.828	-1.066	1.066	FALSE
Angptl3	140-167	10 min	3.056	-0.994	0.994	FALSE
Angptl3	140-167	60 min	0.936	-0.617	0.617	FALSE
Angptl3	145-167	10 s	0.905	-0.483	0.483	FALSE
Angptl3	145-167	2 min	1.314	-0.232	0.232	FALSE
Angptl3	145-167	10 min	1.095	-0.079	0.079	FALSE
Angptl3	145-167	60 min	1.317	-0.276	0.276	FALSE
Angptl3	146-167	10 s	0.653	0.125	0.125	FALSE
Angptl3	146-167	2 min	0.631	-0.155	0.155	FALSE
Angptl3	146-167	10 min	1.050	-0.204	0.204	FALSE
Angptl3	146-167	60 min	0.897	-0.312	0.312	FALSE
Angptl3	149-167	10 s	0.432	-0.225	0.225	FALSE
Angptl3	149-167	2 min	0.534	-0.217	0.217	FALSE
Angptl3	149-167	10 min	0.811	-0.436	0.436	FALSE
Angptl3	149-167	60 min	0.851	-0.436	0.436	FALSE
Angptl3	166-172	10 s	0.112	0.151	0.151	TRUE
Angptl3	166-172	2 min	0.129	0.169	0.169	TRUE
Angptl3	166-172	10 min	0.226	0.241	0.241	TRUE
Angptl3	166-172	60 min	0.284	-0.012	0.012	FALSE
Angptl3	168-178	10 s	0.148	0.078	0.078	FALSE
Angptl3	168-178	2 min	0.174	0.145	0.145	FALSE
Angptl3	168-178	10 min	0.254	0.114	0.114	FALSE
Angptl3	168-178	60 min	0.397	-0.055	0.055	FALSE
Angptl3	168-184	10 s	0.965	0.002	0.002	FALSE
Angptl3	168-184	2 min	1.292	-0.009	0.009	FALSE
Angptl3	168-184	10 min	0.718	0.135	0.135	FALSE
Angptl3	168-184	60 min	0.772	-0.508	0.508	FALSE
Angptl3	168-189	10 s	0.313	0.112	0.112	FALSE
Angptl3	168-189	2 min	0.414	-0.176	0.176	FALSE
Angptl3	168-189	10 min	0.714	-0.035	0.035	FALSE
Angptl3	168-189	60 min	0.784	-0.258	0.258	FALSE
Angptl3	171-189	10 s	0.275	0.189	0.189	FALSE
Angptl3	171-189	2 min	0.280	-0.138	0.138	FALSE
Angptl3	171-189	10 min	0.528	0.061	0.061	FALSE
Angptl3	171-189	60 min	1.738	-0.743	0.743	FALSE
Angptl3	173-189	10 s	0.396	0.183	0.183	FALSE
Angptl3	173-189	2 min	0.321	0.071	0.071	FALSE
Angptl3	173-189	10 min	0.706	0.454	0.454	FALSE
Angptl3	173-189	60 min	0.890	0.000	0.000	FALSE
Angptl3	177-189	10 s	0.274	0.096	0.096	FALSE
Angptl3	177-189	2 min	0.410	-0.094	0.094	FALSE
Angptl3	177-189	10 min	0.597	0.058	0.058	FALSE
Angptl3	177-189	60 min	0.845	-0.017	0.017	FALSE
Angptl3	179-189	10 s	0.287	0.192	0.192	FALSE
Angptl3	179-189	2 min	0.177	0.038	0.038	FALSE
Angptl3	179-189	10 min	0.339	0.170	0.170	FALSE
Angptl3	179-189	60 min	0.262	-0.012	0.012	FALSE
Angptl3	190-200	10 s	0.217	-0.009	0.009	FALSE
Angptl3	190-200	2 min	0.374	0.199	0.199	FALSE
Angptl3	190-200	10 min	0.273	-0.010	0.010	FALSE

Protein	peptide	Exposure	U=ku k=2	Δ (HX)t	ABS(T)	True = significant difference, reject Null (no difference)
Angptl3	190-203	10 s	0.231	0.063	0.063	FALSE
Angptl3	190-203	2 min	0.293	-0.056	0.056	FALSE
Angptl3	190-203	10 min	0.518	-0.038	0.038	FALSE
Angptl3	190-203	60 min	0.336	-0.139	0.139	FALSE
Angptl3	190-205	10 s	0.165	-0.115	0.115	FALSE
Angptl3	190-205	2 min	0.261	-0.186	0.186	FALSE
Angptl3	190-205	10 min	0.311	-0.191	0.191	FALSE
Angptl3	190-205	60 min	0.542	-0.296	0.296	FALSE
Angptl3	190-216	10 s	0.787	-0.720	0.720	FALSE
Angptl3	190-216	2 min	1.008	-0.582	0.582	FALSE
Angptl3	190-216	10 min	1.297	-0.536	0.536	FALSE
Angptl3	190-216	60 min	1.072	-0.642	0.642	FALSE
Angptl3	190-219	10 s	1.110	-0.394	0.394	FALSE
Angptl3	190-219	2 min	1.905	-0.165	0.165	FALSE
Angptl3	190-219	10 min	1.265	-1.082	1.082	FALSE
Angptl3	190-219	60 min	1.579	-1.057	1.057	FALSE
Angptl3	198-205	10 s	0.258	-0.197	0.197	FALSE
Angptl3	198-205	2 min	0.466	-0.322	0.322	FALSE
Angptl3	198-205	10 min	0.434	-0.207	0.207	FALSE
Angptl3	198-205	60 min	0.452	-0.237	0.237	FALSE
Angptl3	198-216	10 s	1.128	-0.102	0.102	FALSE
Angptl3	198-216	2 min	0.522	-0.098	0.098	FALSE
Angptl3	198-216	10 min	0.739	-0.245	0.245	FALSE
Angptl3	198-216	60 min	1.004	-0.323	0.323	FALSE
Angptl3	204-219	10 s	1.565	-0.652	0.652	FALSE
Angptl3	204-219	2 min	1.607	-0.095	0.095	FALSE
Angptl3	204-219	10 min	1.511	-0.156	0.156	FALSE
Angptl3	204-219	60 min	1.844	-0.556	0.556	FALSE
Angptl3	204-231	10 s	1.308	-1.124	1.124	FALSE
Angptl3	204-231	2 min	1.494	-0.946	0.946	FALSE
Angptl3	204-231	10 min	1.284	-0.358	0.358	FALSE
Angptl3	204-231	60 min	1.520	-0.582	0.582	FALSE
Angptl3	217-231	10 s	0.150	0.444	0.444	TRUE
Angptl3	217-231	2 min	0.526	0.361	0.361	FALSE
Angptl3	217-231	10 min	0.318	0.303	0.303	FALSE
Angptl3	217-231	60 min	0.275	-0.013	0.013	FALSE
Angptl3	232-259	10 s	1.607	-0.695	0.695	FALSE
Angptl3	232-259	2 min	1.715	-0.375	0.375	FALSE
Angptl3	232-259	10 min	1.691	-0.377	0.377	FALSE
Angptl3	232-259	60 min	1.384	-0.909	0.909	FALSE
Angptl3	253-259	10 s	0.223	-0.118	0.118	FALSE
Angptl3	253-259	2 min	0.266	-0.183	0.183	FALSE
Angptl3	253-259	10 min	0.194	-0.119	0.119	FALSE
Angptl3	253-259	60 min	0.305	-0.219	0.219	FALSE
Angptl3	260-273	10 s	0.211	0.165	0.165	FALSE
Angptl3	260-273	2 min	0.270	0.180	0.180	FALSE
Angptl3	260-273	10 min	0.393	0.016	0.016	FALSE
Angptl3	260-273	60 min	0.205	-0.174	0.174	FALSE
Angptl3	274-284	10 s	0.361	0.083	0.083	FALSE
Angptl3	274-284	2 min	0.496	-0.131	0.131	FALSE
Angptl3	274-284	10 min	0.587	0.043	0.043	FALSE
Angptl3	274-284	60 min	0.746	-0.108	0.108	FALSE
Angptl3	276-284	10 s	0.156	-0.012	0.012	FALSE
Angptl3	276-284	2 min	0.183	-0.053	0.053	FALSE
Angptl3	276-284	10 min	0.230	0.008	0.008	FALSE
Angptl3	276-284	60 min	0.332	-0.006	0.006	FALSE
Angptl3	285-290	10 s	0.146	0.099	0.099	FALSE
Angptl3	285-290	2 min	0.191	0.077	0.077	FALSE
Angptl3	285-290	10 min	0.191	0.063	0.063	FALSE
Angptl3	285-290	60 min	0.166	0.046	0.046	FALSE

Protein	peptide	Exposure	U=ku k=2	Δ (HX)t	ABS(T)	True = significant difference, reject Null (no difference)
Angptl3	310-316	2 min	0.080	-0.059	0.059	FALSE
Angptl3	310-316	10 min	0.043	-0.017	0.017	FALSE
Angptl3	310-316	60 min	0.047	0.000	0.000	FALSE
Angptl3	310-319	10 s	0.153	0.077	0.077	FALSE
Angptl3	310-319	2 min	0.176	-0.004	0.004	FALSE
Angptl3	310-319	10 min	0.212	0.002	0.002	FALSE
Angptl3	310-319	60 min	0.147	-0.032	0.032	FALSE
Angptl3	313-319	10 s	0.159	0.105	0.105	FALSE
Angptl3	313-319	2 min	0.102	0.010	0.010	FALSE
Angptl3	313-319	10 min	0.108	0.043	0.043	FALSE
Angptl3	313-319	60 min	0.094	0.062	0.062	FALSE
Angptl3	320-325	10 s	0.043	0.051	0.051	TRUE
Angptl3	320-325	2 min	0.067	-0.040	0.040	FALSE
Angptl3	320-325	10 min	0.093	0.030	0.030	FALSE
Angptl3	320-325	60 min	0.052	0.040	0.040	FALSE
Angptl3	320-328	10 s	0.095	-0.102	0.102	TRUE
Angptl3	320-328	2 min	0.125	-0.075	0.075	FALSE
Angptl3	320-328	10 min	0.141	-0.028	0.028	FALSE
Angptl3	320-328	60 min	0.110	-0.030	0.030	FALSE
Angptl3	320-329	10 s	0.086	0.103	0.103	TRUE
Angptl3	320-329	2 min	0.101	0.167	0.167	TRUE
Angptl3	320-329	10 min	0.096	0.093	0.093	FALSE
Angptl3	320-329	60 min	0.121	0.129	0.129	TRUE
Angptl3	320-331	10 s	0.141	0.013	0.013	FALSE
Angptl3	320-331	2 min	0.111	-0.089	0.089	FALSE
Angptl3	320-331	10 min	0.123	-0.092	0.092	FALSE
Angptl3	320-331	60 min	0.085	-0.108	0.108	TRUE
Angptl3	322-332	10 s	0.261	-0.035	0.035	FALSE
Angptl3	322-332	2 min	0.319	0.150	0.150	FALSE
Angptl3	322-332	10 min	0.362	-0.055	0.055	FALSE
Angptl3	322-332	60 min	0.336	0.012	0.012	FALSE
Angptl3	333-342	10 s	0.568	-0.317	0.317	FALSE
Angptl3	333-342	2 min	0.266	-0.095	0.095	FALSE
Angptl3	333-342	10 min	0.416	-0.286	0.286	FALSE
Angptl3	333-342	60 min	0.440	-0.300	0.300	FALSE
Angptl3	335-340	10 s	0.113	-0.001	0.001	FALSE
Angptl3	335-340	2 min	0.092	-0.012	0.012	FALSE
Angptl3	335-340	10 min	0.123	0.002	0.002	FALSE
Angptl3	335-340	60 min	0.108	-0.040	0.040	FALSE
Angptl3	335-342	10 s	0.157	-0.055	0.055	FALSE
Angptl3	335-342	2 min	0.145	0.015	0.015	FALSE
Angptl3	335-342	10 min	0.158	-0.038	0.038	FALSE
Angptl3	335-342	60 min	0.221	-0.094	0.094	FALSE
Angptl3	335-346	10 s	0.149	-0.169	0.169	TRUE
Angptl3	335-346	2 min	0.208	-0.110	0.110	FALSE
Angptl3	335-346	10 min	0.246	-0.137	0.137	FALSE
Angptl3	335-346	60 min	0.309	-0.181	0.181	FALSE
Angptl3	336-342	10 s	0.186	0.003	0.003	FALSE
Angptl3	336-342	2 min	0.319	0.028	0.028	FALSE
Angptl3	336-342	10 min	0.279	0.005	0.005	FALSE
Angptl3	336-342	60 min	0.320	-0.099	0.099	FALSE
Angptl3	343-349	10 s	0.120	0.092	0.092	FALSE
Angptl3	343-349	2 min	0.148	0.048	0.048	FALSE
Angptl3	343-349	10 min	0.243	0.013	0.013	FALSE
Angptl3	343-349	60 min	0.324	0.039	0.039	FALSE
Angptl3	363-377	10 s	0.105	-0.014	0.014	FALSE
Angptl3	363-377	2 min	0.279	0.036	0.036	FALSE
Angptl3	363-377	10 min	0.346	0.046	0.046	FALSE
Angptl3	363-377	60 min	0.503	-0.103	0.103	FALSE
Angptl3	363-378	10 s	0.367	-0.064	0.064	FALSE

Protein	peptide	Exposure	U=ku k=2	Δ (HX)t	ABS(T)	True = significant difference, reject Null (no difference)
Angptl3	363-378	10 min	0.616	-0.054	0.054	FALSE
Angptl3	363-378	60 min	0.764	-0.152	0.152	FALSE
Angptl3	363-379	10 s	0.440	-0.270	0.270	FALSE
Angptl3	363-379	2 min	0.564	-0.270	0.270	FALSE
Angptl3	363-379	10 min	0.532	-0.224	0.224	FALSE
Angptl3	363-379	60 min	0.358	-0.241	0.241	FALSE
Angptl3	363-381	10 s	1.102	0.066	0.066	FALSE
Angptl3	363-381	2 min	0.996	0.084	0.084	FALSE
Angptl3	363-381	10 min	1.088	0.243	0.243	FALSE
Angptl3	363-381	60 min	0.979	0.291	0.291	FALSE
Angptl3	365-377	10 s	0.098	-0.120	0.120	TRUE
Angptl3	365-377	2 min	0.263	-0.020	0.020	FALSE
Angptl3	365-377	10 min	0.262	-0.108	0.108	FALSE
Angptl3	365-377	60 min	0.546	-0.279	0.279	FALSE
Angptl3	365-378	10 s	0.195	-0.122	0.122	FALSE
Angptl3	365-378	2 min	0.273	0.000	0.000	FALSE
Angptl3	365-378	10 min	0.406	-0.076	0.076	FALSE
Angptl3	365-378	60 min	0.460	-0.238	0.238	FALSE
Angptl3	365-379	10 s	0.131	-0.101	0.101	FALSE
Angptl3	365-379	2 min	0.263	-0.070	0.070	FALSE
Angptl3	365-379	10 min	0.353	-0.103	0.103	FALSE
Angptl3	365-379	60 min	0.371	-0.174	0.174	FALSE
Angptl3	365-381	10 s	0.532	0.083	0.083	FALSE
Angptl3	365-381	2 min	0.690	-0.006	0.006	FALSE
Angptl3	365-381	10 min	0.570	0.233	0.233	FALSE
Angptl3	365-381	60 min	0.957	-0.011	0.011	FALSE
Angptl3	378-386	10 s	0.213	-0.043	0.043	FALSE
Angptl3	378-386	2 min	0.279	-0.079	0.079	FALSE
Angptl3	378-386	10 min	0.238	-0.026	0.026	FALSE
Angptl3	378-386	60 min	0.205	-0.008	0.008	FALSE
Angptl3	379-386	10 s	0.080	-0.029	0.029	FALSE
Angptl3	379-386	2 min	0.084	-0.022	0.022	FALSE
Angptl3	379-386	10 min	0.110	-0.030	0.030	FALSE
Angptl3	379-386	60 min	0.089	-0.020	0.020	FALSE
Angptl3	380-388	10 s	0.225	0.073	0.073	FALSE
Angptl3	380-388	2 min	0.243	0.011	0.011	FALSE
Angptl3	380-388	10 min	0.340	0.199	0.199	FALSE
Angptl3	380-388	60 min	0.392	0.042	0.042	FALSE
Angptl3	389-407	10 s	0.384	0.121	0.121	FALSE
Angptl3	389-407	2 min	0.435	-0.029	0.029	FALSE
Angptl3	389-407	10 min	0.442	-0.071	0.071	FALSE
Angptl3	389-407	60 min	0.430	-0.047	0.047	FALSE
Angptl3	408-413	10 s	0.100	-0.114	0.114	TRUE
Angptl3	408-413	2 min	0.128	-0.064	0.064	FALSE
Angptl3	408-413	10 min	0.207	-0.084	0.084	FALSE
Angptl3	408-413	60 min	0.165	-0.175	0.175	TRUE
Angptl3	408-416	10 s	0.136	-0.079	0.079	FALSE
Angptl3	408-416	2 min	0.177	-0.162	0.162	FALSE
Angptl3	408-416	10 min	0.156	-0.066	0.066	FALSE
Angptl3	408-416	60 min	0.181	-0.149	0.149	FALSE
Angptl3	408-441	10 s	1.058	-0.680	0.680	FALSE
Angptl3	408-441	2 min	1.164	-0.113	0.113	FALSE
Angptl3	408-441	10 min	1.408	-0.386	0.386	FALSE
Angptl3	408-441	60 min	1.373	-0.825	0.825	FALSE
Angptl3	417-441	10 s	0.515	-0.272	0.272	FALSE
Angptl3	417-441	2 min	0.309	0.178	0.178	FALSE
Angptl3	417-441	10 min	0.350	-0.064	0.064	FALSE
Angptl3	417-441	60 min	0.696	-0.264	0.264	FALSE
Angptl3	417-445	10 s	1.450	-0.367	0.367	FALSE
Angptl3	417-445	2 min	1.458	-0.086	0.086	FALSE

Protein	peptide	Exposure	U=ku k=2	Δ (HX)t	ABS(T)	True = significant difference, reject Null (no difference)
Angptl3	417-445	60 min	1.140	-0.267	0.267	FALSE
Angptl3	433-441	10 s	0.253	-0.376	0.376	TRUE
Angptl3	433-441	2 min	0.241	-0.166	0.166	FALSE
Angptl3	433-441	10 min	0.289	-0.328	0.328	TRUE
Angptl3	433-441	60 min	0.330	-0.328	0.328	FALSE
Angptl3	433-445	10 s	0.233	-0.095	0.095	FALSE
Angptl3	433-445	2 min	0.369	-0.033	0.033	FALSE
Angptl3	433-445	10 min	0.387	-0.069	0.069	FALSE
Angptl3	433-445	60 min	0.442	-0.152	0.152	FALSE
Angptl3	442-450	10 s	0.144	0.042	0.042	FALSE
Angptl3	442-450	2 min	0.120	0.065	0.065	FALSE
Angptl3	442-450	10 min	0.183	0.097	0.097	FALSE
Angptl3	442-450	60 min	0.098	0.154	0.154	TRUE
Angptl3	443-450	10 s	0.138	0.005	0.005	FALSE
Angptl3	443-450	2 min	0.162	-0.089	0.089	FALSE
Angptl3	443-450	10 min	0.117	-0.054	0.054	FALSE
Angptl3	443-450	60 min	0.050	-0.019	0.019	FALSE
Angptl3	446-463	10 s	0.916	-1.126	1.126	TRUE
Angptl3	446-463	2 min	0.867	-0.699	0.699	FALSE
Angptl3	446-463	10 min	0.489	-0.864	0.864	TRUE
Angptl3	446-463	60 min	0.571	-0.320	0.320	FALSE
Angptl3	449-461	10 s	0.402	-0.290	0.290	FALSE
Angptl3	449-461	2 min	0.314	-0.104	0.104	FALSE
Angptl3	449-461	10 min	0.455	-0.341	0.341	FALSE
Angptl3	449-461	60 min	0.333	-0.177	0.177	FALSE
Angptl3	451-459	10 s	0.442	-0.262	0.262	FALSE
Angptl3	451-459	2 min	0.567	0.146	0.146	FALSE
Angptl3	451-459	10 min	0.408	-0.173	0.173	FALSE
Angptl3	451-459	60 min	0.481	0.046	0.046	FALSE
Angptl3	451-461	10 s	0.243	-0.607	0.607	TRUE
Angptl3	451-461	2 min	0.270	-0.568	0.568	TRUE
Angptl3	451-461	10 min	0.385	-0.322	0.322	FALSE
Angptl3	451-461	60 min	0.321	-0.363	0.363	TRUE
Angptl3	451-463	10 s	0.343	-0.298	0.298	FALSE
Angptl3	451-463	2 min	0.443	-0.065	0.065	FALSE
Angptl3	451-463	10 min	0.428	-0.125	0.125	FALSE
Angptl3	451-463	60 min	0.422	-0.209	0.209	FALSE
Angptl3	462-471	10 s	0.508	-0.357	0.357	FALSE
Angptl3	462-471	2 min	0.482	0.000	0.000	FALSE
Angptl3	462-471	10 min	0.450	-0.042	0.042	FALSE
Angptl3	462-471	60 min	0.551	-0.378	0.378	FALSE
Angptl3	464-471	10 s	0.249	-0.215	0.215	FALSE
Angptl3	464-471	2 min	0.311	-0.078	0.078	FALSE
Angptl3	464-471	10 min	0.280	-0.102	0.102	FALSE
Angptl3	464-471	60 min	0.265	-0.173	0.173	FALSE
Angptl8	36-49	10 s	0.327	1.820	1.820	TRUE
Angptl8	36-49	2 min	0.372	2.451	2.451	TRUE
Angptl8	36-49	10 min	0.525	2.766	2.766	TRUE
Angptl8	36-49	60 min	0.390	1.842	1.842	TRUE
Angptl8	38-49	10 s	0.092	1.546	1.546	TRUE
Angptl8	38-49	2 min	0.200	1.864	1.864	TRUE
Angptl8	38-49	10 min	0.257	1.814	1.814	TRUE
Angptl8	38-49	60 min	0.218	1.070	1.070	TRUE
Angptl8	39-49	10 s	0.066	1.699	1.699	TRUE
Angptl8	39-49	2 min	0.199	2.136	2.136	TRUE
Angptl8	39-49	10 min	0.222	2.074	2.074	TRUE
Angptl8	39-49	60 min	0.357	1.106	1.106	TRUE
Angptl8	50-62	10 s	0.333	0.771	0.771	TRUE
Angptl8	50-62	2 min	0.638	0.787	0.787	TRUE
Angptl8	50-62	10 min	0.678	0.542	0.542	FALSE

Protein	peptide	Exposure	U=ku k=2	Δ (HX)t	ABS(T)	True = significant difference, reject Null (no difference)
Angptl8	50-64	10 s	0.127	1.088	1.088	TRUE
Angptl8	50-64	2 min	0.221	1.284	1.284	TRUE
Angptl8	50-64	10 min	0.182	1.220	1.220	TRUE
Angptl8	50-64	60 min	0.305	0.734	0.734	TRUE
Angptl8	53-64	10 s	1.053	0.621	0.621	FALSE
Angptl8	53-64	2 min	1.191	1.020	1.020	FALSE
Angptl8	53-64	10 min	0.889	0.755	0.755	FALSE
Angptl8	53-64	60 min	1.380	0.148	0.148	FALSE
Angptl8	65-76	10 s	0.095	0.353	0.353	TRUE
Angptl8	65-76	2 min	0.200	0.474	0.474	TRUE
Angptl8	65-76	10 min	0.207	0.410	0.410	TRUE
Angptl8	65-76	60 min	0.145	0.122	0.122	FALSE
Angptl8	77-91	10 s	0.203	0.120	0.120	FALSE
Angptl8	77-91	2 min	0.190	-0.046	0.046	FALSE
Angptl8	77-91	10 min	0.296	0.237	0.237	FALSE
Angptl8	77-91	60 min	0.393	0.041	0.041	FALSE
Angptl8	77-94	10 s	0.372	0.192	0.192	FALSE
Angptl8	77-94	2 min	0.292	-0.241	0.241	FALSE
Angptl8	77-94	10 min	0.376	0.237	0.237	FALSE
Angptl8	77-94	60 min	0.350	-0.726	0.726	TRUE
Angptl8	77-95	10 s	0.313	0.207	0.207	FALSE
Angptl8	77-95	2 min	0.282	-0.076	0.076	FALSE
Angptl8	77-95	10 min	0.455	0.306	0.306	FALSE
Angptl8	77-95	60 min	0.415	0.145	0.145	FALSE
Angptl8	77-103	10 s	0.486	0.372	0.372	FALSE
Angptl8	77-103	2 min	0.325	0.001	0.001	FALSE
Angptl8	77-103	10 min	0.495	0.483	0.483	FALSE
Angptl8	77-103	60 min	0.534	0.118	0.118	FALSE
Angptl8	81-91	10 s	0.163	0.006	0.006	FALSE
Angptl8	81-91	2 min	0.243	-0.144	0.144	FALSE
Angptl8	81-91	10 min	0.281	-0.038	0.038	FALSE
Angptl8	81-91	60 min	0.656	-0.147	0.147	FALSE
Angptl8	81-94	10 s	0.228	0.105	0.105	FALSE
Angptl8	81-94	2 min	0.289	0.051	0.051	FALSE
Angptl8	81-94	10 min	0.436	0.120	0.120	FALSE
Angptl8	81-94	60 min	0.861	0.017	0.017	FALSE
Angptl8	81-95	10 s	0.210	0.180	0.180	FALSE
Angptl8	81-95	2 min	0.208	0.062	0.062	FALSE
Angptl8	81-95	10 min	0.392	0.270	0.270	FALSE
Angptl8	81-95	60 min	0.694	0.135	0.135	FALSE
Angptl8	92-103	10 s	0.331	-0.114	0.114	FALSE
Angptl8	92-103	2 min	0.145	-0.342	0.342	TRUE
Angptl8	92-103	10 min	0.299	-0.104	0.104	FALSE
Angptl8	92-103	60 min	0.124	-0.436	0.436	TRUE
Angptl8	96-103	10 s	0.130	0.136	0.136	TRUE
Angptl8	96-103	2 min	0.164	-0.103	0.103	FALSE
Angptl8	96-103	10 min	0.240	0.129	0.129	FALSE
Angptl8	96-103	60 min	0.497	0.152	0.152	FALSE
Angptl8	104-109	10 s	0.065	0.105	0.105	TRUE
Angptl8	104-109	2 min	0.092	0.060	0.060	FALSE
Angptl8	104-109	10 min	0.082	0.060	0.060	FALSE
Angptl8	104-109	60 min	0.069	0.049	0.049	FALSE
Angptl8	104-124	10 s	1.210	0.234	0.234	FALSE
Angptl8	104-124	2 min	1.138	0.067	0.067	FALSE
Angptl8	104-124	10 min	0.768	0.068	0.068	FALSE
Angptl8	104-124	60 min	1.061	0.561	0.561	FALSE
Angptl8	106-128	10 s	0.500	0.181	0.181	FALSE
Angptl8	106-128	2 min	0.660	-0.242	0.242	FALSE
Angptl8	106-128	10 min	0.545	-0.234	0.234	FALSE
Angptl8	106-128	60 min	0.609	0.030	0.030	FALSE

Protein	peptide	Exposure	U=ku k=2	Δ (HX)t	ABS(T)	True = significant difference, reject Null (no difference)
Angptl8	108-124	2 min	0.308	0.023	0.023	FALSE
Angptl8	108-124	10 min	0.520	0.138	0.138	FALSE
Angptl8	108-124	60 min	0.634	0.027	0.027	FALSE
Angptl8	108-127	10 s	0.344	0.128	0.128	FALSE
Angptl8	108-127	2 min	0.357	-0.020	0.020	FALSE
Angptl8	108-127	10 min	2.377	-0.273	0.273	FALSE
Angptl8	108-127	60 min	1.396	-0.133	0.133	FALSE
Angptl8	108-128	10 s	0.221	0.292	0.292	TRUE
Angptl8	108-128	2 min	0.160	-0.097	0.097	FALSE
Angptl8	108-128	10 min	0.351	0.023	0.023	FALSE
Angptl8	108-128	60 min	0.474	-0.528	0.528	TRUE
Angptl8	108-133	10 s	0.386	0.469	0.469	TRUE
Angptl8	108-133	2 min	0.203	-0.297	0.297	TRUE
Angptl8	108-133	10 min	0.461	-0.010	0.010	FALSE
Angptl8	108-133	60 min	0.356	-0.450	0.450	TRUE
Angptl8	111-128	10 s	0.325	0.164	0.164	FALSE
Angptl8	111-128	2 min	0.312	0.047	0.047	FALSE
Angptl8	111-128	10 min	0.628	0.177	0.177	FALSE
Angptl8	111-128	60 min	0.621	0.231	0.231	FALSE
Angptl8	111-133	10 s	0.234	0.160	0.160	FALSE
Angptl8	111-133	2 min	0.365	-0.173	0.173	FALSE
Angptl8	111-133	10 min	0.482	0.029	0.029	FALSE
Angptl8	111-133	60 min	1.071	0.275	0.275	FALSE
Angptl8	125-133	10 s	0.087	0.071	0.071	FALSE
Angptl8	125-133	2 min	0.044	-0.044	0.044	FALSE
Angptl8	125-133	10 min	0.074	-0.028	0.028	FALSE
Angptl8	125-133	60 min	0.165	-0.140	0.140	FALSE
Angptl8	126-133	10 s	0.136	-0.074	0.074	FALSE
Angptl8	126-133	2 min	0.096	-0.021	0.021	FALSE
Angptl8	126-133	10 min	0.042	0.036	0.036	FALSE
Angptl8	126-133	60 min	0.187	-0.209	0.209	TRUE
Angptl8	139-148	10 s	0.377	-0.350	0.350	FALSE
Angptl8	139-148	2 min	0.402	-0.145	0.145	FALSE
Angptl8	139-148	10 min	0.359	-0.214	0.214	FALSE
Angptl8	139-148	60 min	0.329	-0.364	0.364	TRUE
Angptl8	150-163	10 s	0.725	0.165	0.165	FALSE
Angptl8	150-163	2 min	0.748	0.442	0.442	FALSE
Angptl8	150-163	10 min	0.874	0.402	0.402	FALSE
Angptl8	150-163	60 min	0.936	0.548	0.548	FALSE
Angptl8	153-163	10 s	0.238	-0.045	0.045	FALSE
Angptl8	153-163	2 min	0.291	-0.056	0.056	FALSE
Angptl8	153-163	10 min	0.434	-0.075	0.075	FALSE
Angptl8	153-163	60 min	0.540	-0.143	0.143	FALSE
Angptl8	153-177	10 s	0.414	0.022	0.022	FALSE
Angptl8	153-177	2 min	0.393	-0.165	0.165	FALSE
Angptl8	153-177	10 min	0.746	-0.175	0.175	FALSE
Angptl8	153-177	60 min	0.991	-0.255	0.255	FALSE
Angptl8	164-177	10 s	0.167	0.049	0.049	FALSE
Angptl8	164-177	2 min	0.153	-0.103	0.103	FALSE
Angptl8	164-177	10 min	0.243	0.005	0.005	FALSE
Angptl8	164-177	60 min	0.275	-0.036	0.036	FALSE
Angptl8	166-177	10 s	0.303	0.010	0.010	FALSE
Angptl8	166-177	2 min	0.548	-0.120	0.120	FALSE
Angptl8	166-177	10 min	0.394	-0.061	0.061	FALSE
Angptl8	166-177	60 min	0.987	0.250	0.250	FALSE
Angptl8	176-184	10 s	0.228	0.314	0.314	TRUE
Angptl8	176-184	2 min	0.251	0.129	0.129	FALSE
Angptl8	176-184	10 min	0.356	0.187	0.187	FALSE
Angptl8	176-184	60 min	0.150	0.058	0.058	FALSE
Angptl8	177-190	10 s	0.270	-0.195	0.195	FALSE

Protein	peptide	Exposure	U=ku k=2	Δ (HX)t	ABS(T)	True = significant difference, reject Null (no difference)
Angptl8	177-190	10 min	0.449	-0.522	0.522	TRUE
Angptl8	177-190	60 min	0.549	-0.437	0.437	FALSE
Angptl8	178-190	10 s	0.090	0.070	0.070	FALSE
Angptl8	178-190	2 min	0.198	-0.110	0.110	FALSE
Angptl8	178-190	10 min	0.256	-0.151	0.151	FALSE
Angptl8	178-190	60 min	0.341	-0.169	0.169	FALSE
Angptl8	178-191	10 s	0.186	0.139	0.139	FALSE
Angptl8	178-191	2 min	0.301	-0.115	0.115	FALSE
Angptl8	178-191	10 min	0.316	-0.117	0.117	FALSE
Angptl8	178-191	60 min	0.288	-0.155	0.155	FALSE
Angptl8	178-198	10 s	0.308	-0.260	0.260	FALSE
Angptl8	178-198	2 min	0.568	-0.314	0.314	FALSE
Angptl8	178-198	10 min	0.617	-0.300	0.300	FALSE
Angptl8	178-198	60 min	0.560	-0.368	0.368	FALSE
Angptl8	185-198	10 s	0.237	-0.202	0.202	FALSE
Angptl8	185-198	2 min	0.467	-0.194	0.194	FALSE
Angptl8	185-198	10 min	0.581	-0.270	0.270	FALSE
Angptl8	185-198	60 min	0.563	-0.282	0.282	FALSE

Table S5: Differential HX, combined uncertainty, and significance testing for individual HX times for ANGPTL3 and ANGPTL8 peptides in ANGPTL3/8 + antibody compared to unbound ANGPTL3/8

Protein	peptide	Exposure	U=ku k=2	Δ (HX)t	ABS(T)	True = significant difference, reject Null (no difference)
Angptl3	17-36	10 s	0.860	-0.235	0.235	FALSE
Angptl3	17-36	2 min	0.955	-0.239	0.239	FALSE
Angptl3	17-36	10 min	1.029	-0.237	0.237	FALSE
Angptl3	17-36	60 min	0.887	-0.325	0.325	FALSE
Angptl3	17-40	10 s	0.844	0.669	0.669	FALSE
Angptl3	17-40	2 min	0.977	0.149	0.149	FALSE
Angptl3	17-40	10 min	1.004	-0.095	0.095	FALSE
Angptl3	17-40	60 min	0.840	-0.235	0.235	FALSE
Angptl3	17-46	10 s	0.640	1.485	1.485	TRUE
Angptl3	17-46	2 min	1.031	1.665	1.665	TRUE
Angptl3	17-46	10 min	0.954	1.577	1.577	TRUE
Angptl3	17-46	60 min	0.844	0.752	0.752	FALSE
Angptl3	37-42	10 s	0.083	0.423	0.423	TRUE
Angptl3	37-42	2 min	0.153	0.074	0.074	FALSE
Angptl3	37-42	10 min	0.153	0.020	0.020	FALSE
Angptl3	37-42	60 min	0.109	-0.041	0.041	FALSE
Angptl3	37-46	10 s	0.116	1.469	1.469	TRUE
Angptl3	37-46	2 min	0.111	1.663	1.663	TRUE
Angptl3	37-46	10 min	0.156	1.625	1.625	TRUE
Angptl3	37-46	60 min	0.236	1.096	1.096	TRUE
Angptl3	41-46	10 s	0.091	0.990	0.990	TRUE
Angptl3	41-46	2 min	0.094	1.424	1.424	TRUE
Angptl3	41-46	10 min	0.099	1.424	1.424	TRUE
Angptl3	41-46	60 min	0.113	0.913	0.913	TRUE
Angptl3	45-58	10 s	0.172	1.113	1.113	TRUE
Angptl3	45-58	2 min	0.190	2.119	2.119	TRUE
Angptl3	45-58	10 min	0.312	2.874	2.874	TRUE
Angptl3	45-58	60 min	0.272	2.972	2.972	TRUE
Angptl3	45-59	10 s	0.175	1.177	1.177	TRUE
Angptl3	45-59	2 min	0.186	2.237	2.237	TRUE
Angptl3	45-59	10 min	0.613	3.331	3.331	TRUE
Angptl3	45-59	60 min	0.441	3.295	3.295	TRUE
Angptl3	45-60	10 s	0.105	1.009	1.009	TRUE
Angptl3	45-60	2 min	0.070	2.227	2.227	TRUE
Angptl3	45-60	10 min	0.147	3.136	3.136	TRUE
Angptl3	45-60	60 min	0.403	3.375	3.375	TRUE
Angptl3	47-53	10 s	0.099	0.506	0.506	TRUE
Angptl3	47-53	2 min	0.093	1.084	1.084	TRUE
Angptl3	47-53	10 min	0.066	1.468	1.468	TRUE
Angptl3	47-53	60 min	0.067	1.507	1.507	TRUE
Angptl3	47-58	10 s	0.149	0.828	0.828	TRUE
Angptl3	47-58	2 min	0.178	1.728	1.728	TRUE
Angptl3	47-58	10 min	0.200	2.669	2.669	TRUE
Angptl3	47-58	60 min	0.258	2.773	2.773	TRUE
Angptl3	47-59	10 s	0.140	0.759	0.759	TRUE
Angptl3	47-59	2 min	0.155	1.616	1.616	TRUE
Angptl3	47-59	10 min	0.206	2.577	2.577	TRUE
Angptl3	47-59	60 min	0.287	2.690	2.690	TRUE
Angptl3	47-60	10 s	0.144	0.714	0.714	TRUE
Angptl3	47-60	2 min	0.144	1.555	1.555	TRUE
Angptl3	47-60	10 min	0.215	2.535	2.535	TRUE
Angptl3	47-60	60 min	0.271	2.802	2.802	TRUE
Angptl3	47-74	10 s	0.546	1.257	1.257	TRUE
Angptl3	47-74	2 min	0.823	2.559	2.559	TRUE
Angptl3	47-74	10 min	1.166	4.472	4.472	TRUE
Angptl3	47-74	60 min	1.623	4.897	4.897	TRUE

Protein	peptide	Exposure	U=ku k=2	Δ (HX)t	ABS(T)	True = significant difference, reject Null (no difference)
Angptl3	47-75	2 min	1.029	2.330	2.330	TRUE
Angptl3	47-75	10 min	1.635	4.258	4.258	TRUE
Angptl3	47-75	60 min	1.852	4.825	4.825	TRUE
Angptl3	47-76	10 s	1.696	1.344	1.344	FALSE
Angptl3	47-76	2 min	1.709	2.481	2.481	TRUE
Angptl3	47-76	10 min	3.001	4.976	4.976	TRUE
Angptl3	47-76	60 min	2.923	4.801	4.801	TRUE
Angptl3	47-78	10 s	0.480	1.163	1.163	TRUE
Angptl3	47-78	2 min	0.787	2.287	2.287	TRUE
Angptl3	47-78	10 min	1.171	4.245	4.245	TRUE
Angptl3	47-78	60 min	1.464	4.670	4.670	TRUE
Angptl3	51-58	10 s	0.111	0.295	0.295	TRUE
Angptl3	51-58	2 min	0.198	0.631	0.631	TRUE
Angptl3	51-58	10 min	0.338	1.187	1.187	TRUE
Angptl3	51-58	60 min	0.411	1.252	1.252	TRUE
Angptl3	59-75	10 s	0.659	0.376	0.376	FALSE
Angptl3	59-75	2 min	1.213	0.566	0.566	FALSE
Angptl3	59-75	10 min	1.176	1.120	1.120	FALSE
Angptl3	59-75	60 min	1.724	0.540	0.540	FALSE
Angptl3	59-78	10 s	0.400	0.163	0.163	FALSE
Angptl3	59-78	2 min	0.752	0.101	0.101	FALSE
Angptl3	59-78	10 min	0.971	0.724	0.724	FALSE
Angptl3	59-78	60 min	1.112	1.603	1.603	TRUE
Angptl3	60-74	10 s	0.179	0.240	0.240	TRUE
Angptl3	60-74	2 min	0.203	0.374	0.374	TRUE
Angptl3	60-74	10 min	0.349	0.857	0.857	TRUE
Angptl3	60-74	60 min	0.478	1.043	1.043	TRUE
Angptl3	60-76	10 s	0.297	0.178	0.178	FALSE
Angptl3	60-76	2 min	0.422	0.337	0.337	FALSE
Angptl3	60-76	10 min	0.534	0.864	0.864	TRUE
Angptl3	60-76	60 min	0.737	0.976	0.976	TRUE
Angptl3	60-78	10 s	0.444	0.236	0.236	FALSE
Angptl3	60-78	2 min	0.528	0.228	0.228	FALSE
Angptl3	60-78	10 min	0.507	0.884	0.884	TRUE
Angptl3	60-78	60 min	0.685	1.099	1.099	TRUE
Angptl3	61-74	10 s	0.370	0.215	0.215	FALSE
Angptl3	61-74	2 min	0.474	0.340	0.340	FALSE
Angptl3	61-74	10 min	0.660	0.767	0.767	TRUE
Angptl3	61-74	60 min	1.031	0.899	0.899	FALSE
Angptl3	61-75	10 s	0.288	0.194	0.194	FALSE
Angptl3	61-75	2 min	0.346	0.388	0.388	TRUE
Angptl3	61-75	10 min	0.421	0.863	0.863	TRUE
Angptl3	61-75	60 min	0.675	1.147	1.147	TRUE
Angptl3	61-78	10 s	0.544	0.224	0.224	FALSE
Angptl3	61-78	2 min	0.586	0.171	0.171	FALSE
Angptl3	61-78	10 min	0.614	0.631	0.631	TRUE
Angptl3	61-78	60 min	1.973	0.578	0.578	FALSE
Angptl3	75-80	10 s	0.126	0.088	0.088	FALSE
Angptl3	75-80	2 min	0.120	0.093	0.093	FALSE
Angptl3	75-80	10 min	0.135	0.082	0.082	FALSE
Angptl3	75-80	60 min	0.148	-0.025	0.025	FALSE
Angptl3	75-82	10 s	0.436	-0.020	0.020	FALSE
Angptl3	75-82	2 min	0.669	-0.045	0.045	FALSE
Angptl3	75-82	10 min	0.960	0.052	0.052	FALSE
Angptl3	75-82	60 min	1.228	-0.082	0.082	FALSE
Angptl3	76-82	10 s	0.171	-0.029	0.029	FALSE
Angptl3	76-82	2 min	0.248	-0.161	0.161	FALSE
Angptl3	76-82	10 min	0.409	-0.128	0.128	FALSE
Angptl3	76-82	60 min	0.476	-0.170	0.170	FALSE
Angptl3	79-85	10 s	0.103	-0.002	0.002	FALSE

Protein	peptide	Exposure	U=ku k=2	Δ (HX)t	ABS(T)	True = significant difference, reject Null (no difference)
Angptl3	79-85	10 min	0.098	-0.028	0.028	FALSE
Angptl3	79-85	60 min	0.058	-0.064	0.064	TRUE
Angptl3	86-93	10 s	0.199	-0.009	0.009	FALSE
Angptl3	86-93	2 min	0.158	-0.065	0.065	FALSE
Angptl3	86-93	10 min	0.207	0.035	0.035	FALSE
Angptl3	86-93	60 min	0.155	0.039	0.039	FALSE
Angptl3	86-97	10 s	0.170	-0.087	0.087	FALSE
Angptl3	86-97	2 min	0.119	-0.157	0.157	TRUE
Angptl3	86-97	10 min	0.237	0.035	0.035	FALSE
Angptl3	86-97	60 min	0.324	0.014	0.014	FALSE
Angptl3	86-106	10 s	0.452	-0.067	0.067	FALSE
Angptl3	86-106	2 min	0.553	-0.261	0.261	FALSE
Angptl3	86-106	10 min	0.935	0.078	0.078	FALSE
Angptl3	86-106	60 min	1.380	-0.101	0.101	FALSE
Angptl3	86-109	10 s	0.449	-0.048	0.048	FALSE
Angptl3	86-109	2 min	0.490	-0.251	0.251	FALSE
Angptl3	86-109	10 min	0.700	0.087	0.087	FALSE
Angptl3	86-109	60 min	1.295	0.053	0.053	FALSE
Angptl3	88-97	10 s	0.282	0.025	0.025	FALSE
Angptl3	88-97	2 min	0.319	-0.001	0.001	FALSE
Angptl3	88-97	10 min	0.282	0.215	0.215	FALSE
Angptl3	88-97	60 min	0.808	0.123	0.123	FALSE
Angptl3	98-109	10 s	0.125	-0.031	0.031	FALSE
Angptl3	98-109	2 min	0.215	-0.138	0.138	FALSE
Angptl3	98-109	10 min	0.436	-0.014	0.014	FALSE
Angptl3	98-109	60 min	0.753	-0.051	0.051	FALSE
Angptl3	99-109	10 s	0.088	0.030	0.030	FALSE
Angptl3	99-109	2 min	0.178	-0.114	0.114	FALSE
Angptl3	99-109	10 min	0.354	0.082	0.082	FALSE
Angptl3	99-109	60 min	0.569	0.018	0.018	FALSE
Angptl3	121-127	10 s	0.272	-0.041	0.041	FALSE
Angptl3	121-127	2 min	0.337	0.009	0.009	FALSE
Angptl3	121-127	10 min	0.249	0.056	0.056	FALSE
Angptl3	121-127	60 min	0.372	-0.008	0.008	FALSE
Angptl3	124-131	10 s	0.279	-0.096	0.096	FALSE
Angptl3	124-131	2 min	0.290	-0.142	0.142	FALSE
Angptl3	124-131	10 min	0.197	0.001	0.001	FALSE
Angptl3	124-131	60 min	0.264	-0.004	0.004	FALSE
Angptl3	124-133	10 s	0.153	0.062	0.062	FALSE
Angptl3	124-133	2 min	0.150	0.092	0.092	FALSE
Angptl3	124-133	10 min	0.319	0.163	0.163	FALSE
Angptl3	124-133	60 min	0.302	0.149	0.149	FALSE
Angptl3	128-133	10 s	0.128	0.032	0.032	FALSE
Angptl3	128-133	2 min	0.106	0.015	0.015	FALSE
Angptl3	128-133	10 min	0.134	0.016	0.016	FALSE
Angptl3	128-133	60 min	0.147	-0.005	0.005	FALSE
Angptl3	132-137	10 s	0.091	0.026	0.026	FALSE
Angptl3	132-137	2 min	0.077	-0.007	0.007	FALSE
Angptl3	132-137	10 min	0.100	0.003	0.003	FALSE
Angptl3	132-137	60 min	0.083	-0.014	0.014	FALSE
Angptl3	134-162	10 s	1.227	0.005	0.005	FALSE
Angptl3	134-162	2 min	1.055	0.127	0.127	FALSE
Angptl3	134-162	10 min	2.064	-0.187	0.187	FALSE
Angptl3	134-162	60 min	1.554	-0.141	0.141	FALSE
Angptl3	138-148	10 s	1.167	0.074	0.074	FALSE
Angptl3	138-148	2 min	0.498	-0.066	0.066	FALSE
Angptl3	138-148	10 min	0.464	0.086	0.086	FALSE
Angptl3	138-148	60 min	0.410	0.193	0.193	FALSE
Angptl3	138-162	10 s	0.935	-0.334	0.334	FALSE
Angptl3	138-162	2 min	1.040	-0.193	0.193	FALSE

Protein	peptide	Exposure	U=ku k=2	Δ (HX)t	ABS(T)	True = significant difference, reject Null (no difference)
Angptl3	138-162	60 min	1.088	-0.363	0.363	FALSE
Angptl3	138-166	10 s	0.995	-0.132	0.132	FALSE
Angptl3	138-166	2 min	1.246	-0.085	0.085	FALSE
Angptl3	138-166	10 min	1.123	-0.215	0.215	FALSE
Angptl3	138-166	60 min	1.425	-0.307	0.307	FALSE
Angptl3	138-167	10 s	0.694	-0.372	0.372	FALSE
Angptl3	138-167	2 min	0.883	-0.275	0.275	FALSE
Angptl3	138-167	10 min	1.063	-0.283	0.283	FALSE
Angptl3	138-167	60 min	1.159	-0.399	0.399	FALSE
Angptl3	140-167	10 s	0.474	-0.336	0.336	FALSE
Angptl3	140-167	2 min	0.993	-0.370	0.370	FALSE
Angptl3	140-167	10 min	1.242	-0.393	0.393	FALSE
Angptl3	140-167	60 min	0.957	-0.351	0.351	FALSE
Angptl3	145-167	10 s	1.058	-0.276	0.276	FALSE
Angptl3	145-167	2 min	1.302	-0.461	0.461	FALSE
Angptl3	145-167	10 min	1.043	-0.307	0.307	FALSE
Angptl3	145-167	60 min	1.119	-0.465	0.465	FALSE
Angptl3	146-167	10 s	0.755	0.062	0.062	FALSE
Angptl3	146-167	2 min	0.694	-0.136	0.136	FALSE
Angptl3	146-167	10 min	1.164	0.375	0.375	FALSE
Angptl3	146-167	60 min	1.027	0.000	0.000	FALSE
Angptl3	149-167	10 s	0.607	-0.047	0.047	FALSE
Angptl3	149-167	2 min	0.601	-0.082	0.082	FALSE
Angptl3	149-167	10 min	0.884	-0.055	0.055	FALSE
Angptl3	149-167	60 min	1.165	0.055	0.055	FALSE
Angptl3	166-172	10 s	0.146	-0.054	0.054	FALSE
Angptl3	166-172	2 min	0.117	-0.185	0.185	TRUE
Angptl3	166-172	10 min	0.183	-0.047	0.047	FALSE
Angptl3	166-172	60 min	0.165	-0.169	0.169	TRUE
Angptl3	168-178	10 s	0.207	0.046	0.046	FALSE
Angptl3	168-178	2 min	0.284	0.061	0.061	FALSE
Angptl3	168-178	10 min	0.406	0.115	0.115	FALSE
Angptl3	168-178	60 min	0.571	0.009	0.009	FALSE
Angptl3	168-184	10 s	0.842	-0.167	0.167	FALSE
Angptl3	168-184	2 min	1.224	-0.165	0.165	FALSE
Angptl3	168-184	10 min	0.815	0.153	0.153	FALSE
Angptl3	168-184	60 min	1.618	0.220	0.220	FALSE
Angptl3	168-189	10 s	0.307	-0.159	0.159	FALSE
Angptl3	168-189	2 min	0.436	-0.277	0.277	FALSE
Angptl3	168-189	10 min	0.854	-0.358	0.358	FALSE
Angptl3	168-189	60 min	1.118	-0.587	0.587	FALSE
Angptl3	171-189	10 s	0.323	-0.077	0.077	FALSE
Angptl3	171-189	2 min	0.305	-0.178	0.178	FALSE
Angptl3	171-189	10 min	0.689	-0.085	0.085	FALSE
Angptl3	171-189	60 min	1.862	-0.869	0.869	FALSE
Angptl3	173-189	10 s	0.408	-0.057	0.057	FALSE
Angptl3	173-189	2 min	0.447	-0.213	0.213	FALSE
Angptl3	173-189	10 min	0.779	-0.097	0.097	FALSE
Angptl3	173-189	60 min	1.158	-0.243	0.243	FALSE
Angptl3	177-189	10 s	0.256	-0.065	0.065	FALSE
Angptl3	177-189	2 min	0.333	-0.079	0.079	FALSE
Angptl3	177-189	10 min	0.638	-0.043	0.043	FALSE
Angptl3	177-189	60 min	0.969	-0.231	0.231	FALSE
Angptl3	179-189	10 s	0.245	0.012	0.012	FALSE
Angptl3	179-189	2 min	0.175	-0.086	0.086	FALSE
Angptl3	179-189	10 min	0.312	0.063	0.063	FALSE
Angptl3	179-189	60 min	0.340	-0.128	0.128	FALSE
Angptl3	190-200	10 s	0.500	-0.170	0.170	FALSE
Angptl3	190-200	2 min	0.442	-0.103	0.103	FALSE
Angptl3	190-200	10 min	0.585	-0.271	0.271	FALSE

Protein	peptide	Exposure	U=ku k=2	Δ (HX)t	ABS(T)	True = significant difference, reject Null (no difference)
Angptl3	190-203	10 s	0.295	-0.069	0.069	FALSE
Angptl3	190-203	2 min	0.331	-0.229	0.229	FALSE
Angptl3	190-203	10 min	0.491	-0.083	0.083	FALSE
Angptl3	190-203	60 min	0.618	-0.028	0.028	FALSE
Angptl3	190-205	10 s	0.112	-0.172	0.172	TRUE
Angptl3	190-205	2 min	0.474	-0.330	0.330	FALSE
Angptl3	190-205	10 min	0.480	-0.122	0.122	FALSE
Angptl3	190-205	60 min	0.753	-0.165	0.165	FALSE
Angptl3	204-219	10 s	1.572	-0.161	0.161	FALSE
Angptl3	204-219	2 min	1.686	0.167	0.167	FALSE
Angptl3	204-219	10 min	1.679	0.084	0.084	FALSE
Angptl3	204-219	60 min	1.631	-0.121	0.121	FALSE
Angptl3	204-231	10 s	1.660	-0.757	0.757	FALSE
Angptl3	204-231	2 min	2.330	-0.626	0.626	FALSE
Angptl3	204-231	10 min	1.440	-0.650	0.650	FALSE
Angptl3	204-231	60 min	1.857	-1.122	1.122	FALSE
Angptl3	217-231	10 s	0.271	-0.039	0.039	FALSE
Angptl3	217-231	2 min	0.445	0.023	0.023	FALSE
Angptl3	217-231	10 min	0.319	-0.061	0.061	FALSE
Angptl3	217-231	60 min	0.396	-0.080	0.080	FALSE
Angptl3	232-259	10 s	1.206	-0.135	0.135	FALSE
Angptl3	232-259	2 min	1.883	0.072	0.072	FALSE
Angptl3	232-259	10 min	1.770	-0.191	0.191	FALSE
Angptl3	232-259	60 min	1.715	-0.567	0.567	FALSE
Angptl3	253-259	10 s	0.348	-0.061	0.061	FALSE
Angptl3	253-259	2 min	0.321	-0.160	0.160	FALSE
Angptl3	253-259	10 min	0.279	-0.049	0.049	FALSE
Angptl3	253-259	60 min	0.407	-0.136	0.136	FALSE
Angptl3	260-273	10 s	0.272	-0.116	0.116	FALSE
Angptl3	260-273	2 min	0.364	-0.218	0.218	FALSE
Angptl3	260-273	10 min	0.435	-0.064	0.064	FALSE
Angptl3	260-273	60 min	0.376	-0.049	0.049	FALSE
Angptl3	274-284	10 s	0.418	-0.064	0.064	FALSE
Angptl3	274-284	2 min	0.513	-0.137	0.137	FALSE
Angptl3	274-284	10 min	0.690	-0.044	0.044	FALSE
Angptl3	274-284	60 min	0.847	-0.126	0.126	FALSE
Angptl3	276-284	10 s	0.161	-0.056	0.056	FALSE
Angptl3	276-284	2 min	0.207	-0.085	0.085	FALSE
Angptl3	276-284	10 min	0.270	-0.076	0.076	FALSE
Angptl3	276-284	60 min	0.375	-0.089	0.089	FALSE
Angptl3	285-290	10 s	0.165	0.125	0.125	FALSE
Angptl3	285-290	2 min	0.157	0.128	0.128	FALSE
Angptl3	285-290	10 min	0.178	0.094	0.094	FALSE
Angptl3	285-290	60 min	0.204	0.052	0.052	FALSE
Angptl3	310-316	10 s	0.078	-0.014	0.014	FALSE
Angptl3	310-316	2 min	0.049	-0.040	0.040	FALSE
Angptl3	310-316	10 min	0.049	0.040	0.040	FALSE
Angptl3	310-316	60 min	0.063	0.011	0.011	FALSE
Angptl3	310-319	10 s	0.415	-0.106	0.106	FALSE
Angptl3	310-319	2 min	0.295	0.035	0.035	FALSE
Angptl3	310-319	10 min	0.317	0.060	0.060	FALSE
Angptl3	310-319	60 min	0.215	0.066	0.066	FALSE
Angptl3	313-319	10 s	0.143	-0.018	0.018	FALSE
Angptl3	313-319	2 min	0.097	-0.065	0.065	FALSE
Angptl3	313-319	10 min	0.110	0.022	0.022	FALSE
Angptl3	313-319	60 min	0.092	0.031	0.031	FALSE
Angptl3	320-325	10 s	0.059	0.004	0.004	FALSE
Angptl3	320-325	2 min	0.059	-0.013	0.013	FALSE
Angptl3	320-325	10 min	0.058	0.061	0.061	TRUE
Angptl3	320-325	60 min	0.057	0.022	0.022	FALSE

Protein	peptide	Exposure	U=ku k=2	Δ (HX)t	ABS(T)	True = significant difference, reject Null (no difference)
Angptl3	320-328	2 min	0.080	0.005	0.005	FALSE
Angptl3	320-328	10 min	0.110	0.084	0.084	FALSE
Angptl3	320-328	60 min	0.083	0.055	0.055	FALSE
Angptl3	320-329	10 s	0.092	0.042	0.042	FALSE
Angptl3	320-329	2 min	0.128	0.084	0.084	FALSE
Angptl3	320-329	10 min	0.132	0.083	0.083	FALSE
Angptl3	320-329	60 min	0.151	0.085	0.085	FALSE
Angptl3	320-331	10 s	0.185	0.017	0.017	FALSE
Angptl3	320-331	2 min	0.112	0.009	0.009	FALSE
Angptl3	320-331	10 min	0.124	-0.038	0.038	FALSE
Angptl3	320-331	60 min	0.116	-0.066	0.066	FALSE
Angptl3	322-332	10 s	0.295	0.001	0.001	FALSE
Angptl3	322-332	2 min	0.326	0.108	0.108	FALSE
Angptl3	322-332	10 min	0.376	0.115	0.115	FALSE
Angptl3	322-332	60 min	0.396	-0.019	0.019	FALSE
Angptl3	333-342	10 s	0.213	-0.037	0.037	FALSE
Angptl3	333-342	2 min	0.286	-0.129	0.129	FALSE
Angptl3	333-342	10 min	0.358	-0.186	0.186	FALSE
Angptl3	333-342	60 min	0.340	-0.195	0.195	FALSE
Angptl3	335-340	10 s	0.099	0.035	0.035	FALSE
Angptl3	335-340	2 min	0.095	-0.011	0.011	FALSE
Angptl3	335-340	10 min	0.127	-0.001	0.001	FALSE
Angptl3	335-340	60 min	0.132	-0.011	0.011	FALSE
Angptl3	335-342	10 s	0.130	0.094	0.094	FALSE
Angptl3	335-342	2 min	0.218	0.130	0.130	FALSE
Angptl3	335-342	10 min	0.211	0.188	0.188	FALSE
Angptl3	335-342	60 min	0.317	0.085	0.085	FALSE
Angptl3	335-346	10 s	0.142	-0.022	0.022	FALSE
Angptl3	335-346	2 min	0.250	-0.001	0.001	FALSE
Angptl3	335-346	10 min	0.297	0.077	0.077	FALSE
Angptl3	335-346	60 min	0.422	0.043	0.043	FALSE
Angptl3	336-342	10 s	0.241	0.069	0.069	FALSE
Angptl3	336-342	2 min	0.356	0.004	0.004	FALSE
Angptl3	336-342	10 min	0.356	0.112	0.112	FALSE
Angptl3	336-342	60 min	0.392	-0.039	0.039	FALSE
Angptl3	343-349	10 s	0.192	-0.019	0.019	FALSE
Angptl3	343-349	2 min	0.201	-0.090	0.090	FALSE
Angptl3	343-349	10 min	0.249	-0.009	0.009	FALSE
Angptl3	343-349	60 min	0.358	0.059	0.059	FALSE
Angptl3	363-377	10 s	0.235	-0.094	0.094	FALSE
Angptl3	363-377	2 min	0.443	-0.031	0.031	FALSE
Angptl3	363-377	10 min	0.602	0.087	0.087	FALSE
Angptl3	363-377	60 min	0.670	-0.028	0.028	FALSE
Angptl3	363-378	10 s	0.388	-0.167	0.167	FALSE
Angptl3	363-378	2 min	0.602	-0.188	0.188	FALSE
Angptl3	363-378	10 min	0.763	-0.121	0.121	FALSE
Angptl3	363-378	60 min	0.764	-0.249	0.249	FALSE
Angptl3	363-379	10 s	0.273	-0.114	0.114	FALSE
Angptl3	363-379	2 min	0.366	-0.158	0.158	FALSE
Angptl3	363-379	10 min	0.604	-0.121	0.121	FALSE
Angptl3	363-379	60 min	0.455	-0.348	0.348	FALSE
Angptl3	363-381	10 s	1.069	0.084	0.084	FALSE
Angptl3	363-381	2 min	1.247	-0.253	0.253	FALSE
Angptl3	363-381	10 min	1.307	-0.215	0.215	FALSE
Angptl3	363-381	60 min	1.080	-0.169	0.169	FALSE
Angptl3	365-377	10 s	0.131	-0.053	0.053	FALSE
Angptl3	365-377	2 min	0.398	-0.019	0.019	FALSE
Angptl3	365-377	10 min	0.386	0.113	0.113	FALSE
Angptl3	365-377	60 min	0.449	-0.032	0.032	FALSE
Angptl3	365-378	10 s	0.211	-0.142	0.142	FALSE

Protein	peptide	Exposure	U=ku k=2	Δ (HX)t	ABS(T)	True = significant difference, reject Null (no difference)
Angptl3	365-378	10 min	0.464	0.050	0.050	FALSE
Angptl3	365-378	60 min	0.599	-0.055	0.055	FALSE
Angptl3	365-379	10 s	0.410	0.113	0.113	FALSE
Angptl3	365-379	2 min	0.356	0.119	0.119	FALSE
Angptl3	365-379	10 min	0.492	-0.333	0.333	FALSE
Angptl3	365-379	60 min	0.526	-0.005	0.005	FALSE
Angptl3	365-381	10 s	0.570	-0.023	0.023	FALSE
Angptl3	365-381	2 min	0.599	-0.042	0.042	FALSE
Angptl3	365-381	10 min	0.824	0.204	0.204	FALSE
Angptl3	365-381	60 min	1.031	-0.094	0.094	FALSE
Angptl3	378-386	10 s	0.208	-0.014	0.014	FALSE
Angptl3	378-386	2 min	0.224	-0.023	0.023	FALSE
Angptl3	378-386	10 min	0.178	-0.029	0.029	FALSE
Angptl3	378-386	60 min	0.197	-0.021	0.021	FALSE
Angptl3	379-386	10 s	0.098	-0.012	0.012	FALSE
Angptl3	379-386	2 min	0.086	-0.037	0.037	FALSE
Angptl3	379-386	10 min	0.093	-0.028	0.028	FALSE
Angptl3	379-386	60 min	0.080	-0.010	0.010	FALSE
Angptl3	380-388	10 s	0.212	0.097	0.097	FALSE
Angptl3	380-388	2 min	0.281	0.044	0.044	FALSE
Angptl3	380-388	10 min	0.506	0.151	0.151	FALSE
Angptl3	380-388	60 min	0.451	0.063	0.063	FALSE
Angptl3	389-407	10 s	0.422	0.062	0.062	FALSE
Angptl3	389-407	2 min	0.484	-0.063	0.063	FALSE
Angptl3	389-407	10 min	0.486	-0.124	0.124	FALSE
Angptl3	389-407	60 min	0.497	-0.054	0.054	FALSE
Angptl3	408-413	10 s	0.104	-0.024	0.024	FALSE
Angptl3	408-413	2 min	0.154	-0.087	0.087	FALSE
Angptl3	408-413	10 min	0.174	-0.120	0.120	FALSE
Angptl3	408-413	60 min	0.149	-0.109	0.109	FALSE
Angptl3	408-416	10 s	0.195	0.024	0.024	FALSE
Angptl3	408-416	2 min	0.175	-0.033	0.033	FALSE
Angptl3	408-416	10 min	0.259	0.106	0.106	FALSE
Angptl3	408-416	60 min	0.228	-0.034	0.034	FALSE
Angptl3	408-441	10 s	1.381	-0.199	0.199	FALSE
Angptl3	408-441	2 min	1.596	0.051	0.051	FALSE
Angptl3	408-441	10 min	1.870	0.091	0.091	FALSE
Angptl3	408-441	60 min	1.964	-0.484	0.484	FALSE
Angptl3	417-441	10 s	0.728	-0.095	0.095	FALSE
Angptl3	417-441	2 min	0.652	0.126	0.126	FALSE
Angptl3	417-441	10 min	0.598	0.294	0.294	FALSE
Angptl3	417-441	60 min	1.070	-0.192	0.192	FALSE
Angptl3	417-445	10 s	1.629	-0.059	0.059	FALSE
Angptl3	417-445	2 min	1.781	-0.112	0.112	FALSE
Angptl3	417-445	10 min	1.772	-0.002	0.002	FALSE
Angptl3	417-445	60 min	1.505	-0.281	0.281	FALSE
Angptl3	433-441	10 s	0.292	0.022	0.022	FALSE
Angptl3	433-441	2 min	0.357	0.049	0.049	FALSE
Angptl3	433-441	10 min	0.363	0.059	0.059	FALSE
Angptl3	433-441	60 min	0.442	-0.046	0.046	FALSE
Angptl3	433-445	10 s	0.304	-0.084	0.084	FALSE
Angptl3	433-445	2 min	0.493	-0.122	0.122	FALSE
Angptl3	433-445	10 min	0.478	-0.096	0.096	FALSE
Angptl3	433-445	60 min	0.603	-0.204	0.204	FALSE
Angptl3	442-450	10 s	0.138	-0.028	0.028	FALSE
Angptl3	442-450	2 min	0.118	-0.032	0.032	FALSE
Angptl3	442-450	10 min	0.156	0.047	0.047	FALSE
Angptl3	442-450	60 min	0.132	0.066	0.066	FALSE
Angptl3	443-450	10 s	0.105	-0.018	0.018	FALSE
Angptl3	443-450	2 min	0.106	-0.231	0.231	TRUE

Protein	peptide	Exposure	U=ku k=2	Δ (HX)t	ABS(T)	True = significant difference, reject Null (no difference)
Angptl3	443-450	60 min	0.099	-0.058	0.058	FALSE
Angptl3	446-463	10 s	0.784	-0.234	0.234	FALSE
Angptl3	446-463	2 min	0.782	-0.389	0.389	FALSE
Angptl3	446-463	10 min	0.824	-0.041	0.041	FALSE
Angptl3	446-463	60 min	0.529	-0.071	0.071	FALSE
Angptl3	449-461	10 s	0.726	0.354	0.354	FALSE
Angptl3	449-461	2 min	0.470	0.366	0.366	FALSE
Angptl3	449-461	10 min	0.738	0.356	0.356	FALSE
Angptl3	449-461	60 min	0.518	0.285	0.285	FALSE
Angptl3	451-459	10 s	0.553	0.090	0.090	FALSE
Angptl3	451-459	2 min	0.528	0.087	0.087	FALSE
Angptl3	451-459	10 min	0.439	-0.215	0.215	FALSE
Angptl3	451-459	60 min	0.524	0.033	0.033	FALSE
Angptl3	451-461	10 s	0.338	-0.032	0.032	FALSE
Angptl3	451-461	2 min	0.373	-0.081	0.081	FALSE
Angptl3	451-461	10 min	0.427	0.057	0.057	FALSE
Angptl3	451-461	60 min	0.386	-0.062	0.062	FALSE
Angptl3	451-463	10 s	0.465	-0.020	0.020	FALSE
Angptl3	451-463	2 min	0.597	-0.003	0.003	FALSE
Angptl3	451-463	10 min	0.572	0.036	0.036	FALSE
Angptl3	451-463	60 min	0.601	-0.090	0.090	FALSE
Angptl3	462-471	10 s	0.638	0.085	0.085	FALSE
Angptl3	462-471	2 min	0.676	-0.013	0.013	FALSE
Angptl3	462-471	10 min	0.588	0.218	0.218	FALSE
Angptl3	462-471	60 min	0.761	-0.115	0.115	FALSE
Angptl3	464-471	10 s	0.332	-0.005	0.005	FALSE
Angptl3	464-471	2 min	0.429	-0.041	0.041	FALSE
Angptl3	464-471	10 min	0.403	0.085	0.085	FALSE
Angptl3	464-471	60 min	0.390	-0.037	0.037	FALSE
Angptl8	36-49	10 s	0.360	1.744	1.744	TRUE
Angptl8	36-49	2 min	0.355	2.961	2.961	TRUE
Angptl8	36-49	10 min	0.367	3.869	3.869	TRUE
Angptl8	36-49	60 min	0.384	4.344	4.344	TRUE
Angptl8	38-49	10 s	0.123	1.878	1.878	TRUE
Angptl8	38-49	2 min	0.201	2.824	2.824	TRUE
Angptl8	38-49	10 min	0.166	3.618	3.618	TRUE
Angptl8	38-49	60 min	0.250	3.745	3.745	TRUE
Angptl8	39-49	10 s	0.084	2.041	2.041	TRUE
Angptl8	39-49	2 min	0.236	3.124	3.124	TRUE
Angptl8	39-49	10 min	0.217	4.034	4.034	TRUE
Angptl8	39-49	60 min	0.242	4.106	4.106	TRUE
Angptl8	50-62	10 s	0.350	1.677	1.677	TRUE
Angptl8	50-62	2 min	0.725	2.220	2.220	TRUE
Angptl8	50-62	10 min	0.837	2.751	2.751	TRUE
Angptl8	50-62	60 min	0.996	1.877	1.877	TRUE
Angptl8	50-64	10 s	0.137	1.841	1.841	TRUE
Angptl8	50-64	2 min	0.244	2.463	2.463	TRUE
Angptl8	50-64	10 min	0.262	3.198	3.198	TRUE
Angptl8	50-64	60 min	0.387	2.245	2.245	TRUE
Angptl8	53-64	10 s	1.407	1.192	1.192	FALSE
Angptl8	53-64	2 min	1.563	1.788	1.788	TRUE
Angptl8	53-64	10 min	1.045	2.186	2.186	TRUE
Angptl8	53-64	60 min	1.725	1.154	1.154	FALSE
Angptl8	65-76	10 s	0.124	-0.021	0.021	FALSE
Angptl8	65-76	2 min	0.205	0.061	0.061	FALSE
Angptl8	65-76	10 min	0.318	0.083	0.083	FALSE
Angptl8	65-76	60 min	0.289	-0.143	0.143	FALSE
Angptl8	77-91	10 s	0.203	-0.002	0.002	FALSE
Angptl8	77-91	2 min	0.122	-0.088	0.088	FALSE
Angptl8	77-91	10 min	0.371	0.202	0.202	FALSE

Protein	peptide	Exposure	U=ku k=2	Δ (HX)t	ABS(T)	True = significant difference, reject Null (no difference)
Angptl8	77-94	10 s	0.334	0.034	0.034	FALSE
Angptl8	77-94	2 min	0.118	-0.119	0.119	TRUE
Angptl8	77-94	10 min	0.573	0.422	0.422	FALSE
Angptl8	77-94	60 min	0.436	0.257	0.257	FALSE
Angptl8	77-95	10 s	0.341	-0.085	0.085	FALSE
Angptl8	77-95	2 min	0.243	-0.091	0.091	FALSE
Angptl8	77-95	10 min	0.564	0.184	0.184	FALSE
Angptl8	77-95	60 min	0.552	0.153	0.153	FALSE
Angptl8	77-103	10 s	0.587	-0.136	0.136	FALSE
Angptl8	77-103	2 min	0.266	-0.173	0.173	FALSE
Angptl8	77-103	10 min	0.835	0.611	0.611	FALSE
Angptl8	77-103	60 min	0.869	0.393	0.393	FALSE
Angptl8	81-91	10 s	0.127	-0.033	0.033	FALSE
Angptl8	81-91	2 min	0.310	-0.031	0.031	FALSE
Angptl8	81-91	10 min	0.347	0.109	0.109	FALSE
Angptl8	81-91	60 min	0.781	0.023	0.023	FALSE
Angptl8	81-94	10 s	0.217	-0.085	0.085	FALSE
Angptl8	81-94	2 min	0.267	-0.031	0.031	FALSE
Angptl8	81-94	10 min	0.480	0.125	0.125	FALSE
Angptl8	81-94	60 min	0.898	-0.016	0.016	FALSE
Angptl8	81-95	10 s	0.301	-0.083	0.083	FALSE
Angptl8	81-95	2 min	0.283	-0.014	0.014	FALSE
Angptl8	81-95	10 min	0.446	0.179	0.179	FALSE
Angptl8	81-95	60 min	0.867	0.084	0.084	FALSE
Angptl8	92-103	10 s	0.158	-0.027	0.027	FALSE
Angptl8	92-103	2 min	0.110	0.024	0.024	FALSE
Angptl8	92-103	10 min	0.181	0.230	0.230	TRUE
Angptl8	92-103	60 min	0.229	0.096	0.096	FALSE
Angptl8	96-103	10 s	0.163	-0.044	0.044	FALSE
Angptl8	96-103	2 min	0.206	-0.265	0.265	TRUE
Angptl8	96-103	10 min	0.324	-0.033	0.033	FALSE
Angptl8	96-103	60 min	0.578	-0.210	0.210	FALSE
Angptl8	104-109	10 s	0.132	0.073	0.073	FALSE
Angptl8	104-109	2 min	0.131	0.115	0.115	FALSE
Angptl8	104-109	10 min	0.132	0.126	0.126	FALSE
Angptl8	104-109	60 min	0.120	0.194	0.194	TRUE
Angptl8	104-124	10 s	0.529	0.100	0.100	FALSE
Angptl8	104-124	2 min	0.568	-0.167	0.167	FALSE
Angptl8	104-124	10 min	0.869	-0.350	0.350	FALSE
Angptl8	104-124	60 min	0.920	-0.250	0.250	FALSE
Angptl8	106-128	10 s	0.359	-0.081	0.081	FALSE
Angptl8	106-128	2 min	0.262	-0.366	0.366	TRUE
Angptl8	106-128	10 min	0.364	-0.310	0.310	FALSE
Angptl8	106-128	60 min	0.385	-0.350	0.350	FALSE
Angptl8	108-124	10 s	0.314	-0.160	0.160	FALSE
Angptl8	108-124	2 min	0.290	-0.259	0.259	FALSE
Angptl8	108-124	10 min	0.498	-0.085	0.085	FALSE
Angptl8	108-124	60 min	0.579	-0.183	0.183	FALSE
Angptl8	108-127	10 s	0.442	-0.168	0.168	FALSE
Angptl8	108-127	2 min	0.383	-0.044	0.044	FALSE
Angptl8	108-127	10 min	0.447	-0.298	0.298	FALSE
Angptl8	108-127	60 min	0.417	-0.612	0.612	TRUE
Angptl8	108-128	10 s	0.529	-0.187	0.187	FALSE
Angptl8	108-128	2 min	0.422	-0.215	0.215	FALSE
Angptl8	108-128	10 min	0.489	-0.053	0.053	FALSE
Angptl8	108-128	60 min	0.797	-0.499	0.499	FALSE
Angptl8	108-133	10 s	0.466	-0.122	0.122	FALSE
Angptl8	108-133	2 min	0.232	-0.360	0.360	TRUE
Angptl8	108-133	10 min	0.468	-0.098	0.098	FALSE
Angptl8	108-133	60 min	0.589	-0.485	0.485	FALSE

Angptl8	111-128	10 s	0.385	0.081	0.081	FALSE
Angptl8	111-128	2 min	0.318	-0.067	0.067	FALSE
Angptl8	111-128	10 min	0.774	0.142	0.142	FALSE
Angptl8	111-128	60 min	0.745	0.218	0.218	FALSE
Angptl8	111-133	10 s	0.313	-0.111	0.111	FALSE
Angptl8	111-133	2 min	0.416	-0.103	0.103	FALSE
Angptl8	111-133	10 min	0.731	0.068	0.068	FALSE
Angptl8	111-133	60 min	0.925	-0.019	0.019	FALSE
Angptl8	125-133	10 s	0.084	-0.013	0.013	FALSE
Angptl8	125-133	2 min	0.049	-0.043	0.043	FALSE
Angptl8	125-133	10 min	0.080	-0.045	0.045	FALSE
Angptl8	125-133	60 min	0.220	-0.165	0.165	FALSE
Angptl8	126-133	10 s	0.085	-0.016	0.016	FALSE
Angptl8	126-133	2 min	0.075	-0.051	0.051	FALSE
Angptl8	126-133	10 min	0.094	-0.025	0.025	FALSE
Angptl8	126-133	60 min	0.270	-0.130	0.130	FALSE
Angptl8	139-148	10 s	0.372	-0.095	0.095	FALSE
Angptl8	139-148	2 min	0.426	-0.175	0.175	FALSE
Angptl8	139-148	10 min	0.403	-0.022	0.022	FALSE
Angptl8	139-148	60 min	0.337	-0.038	0.038	FALSE
Angptl8	150-163	10 s	0.634	-0.014	0.014	FALSE
Angptl8	150-163	2 min	0.702	-0.151	0.151	FALSE
Angptl8	150-163	10 min	0.607	-0.054	0.054	FALSE
Angptl8	150-163	60 min	0.677	-0.119	0.119	FALSE
Angptl8	153-163	10 s	0.178	-0.040	0.040	FALSE
Angptl8	153-163	2 min	0.361	-0.132	0.132	FALSE
Angptl8	153-163	10 min	0.514	0.055	0.055	FALSE
Angptl8	153-163	60 min	0.599	-0.137	0.137	FALSE
Angptl8	153-177	10 s	0.506	-0.096	0.096	FALSE
Angptl8	153-177	2 min	0.347	-0.314	0.314	FALSE
Angptl8	153-177	10 min	0.959	-0.227	0.227	FALSE
Angptl8	153-177	60 min	0.884	-0.464	0.464	FALSE
Angptl8	164-177	10 s	0.209	-0.097	0.097	FALSE
Angptl8	164-177	2 min	0.170	-0.202	0.202	TRUE
Angptl8	164-177	10 min	0.272	-0.103	0.103	FALSE
Angptl8	164-177	60 min	0.355	-0.220	0.220	FALSE
Angptl8	166-177	10 s	0.324	-0.041	0.041	FALSE
Angptl8	166-177	2 min	0.425	-0.049	0.049	FALSE
Angptl8	166-177	10 min	0.524	-0.073	0.073	FALSE
Angptl8	166-177	60 min	0.772	0.015	0.015	FALSE
Angptl8	176-184	10 s	0.293	0.078	0.078	FALSE
Angptl8	176-184	2 min	0.319	0.015	0.015	FALSE
Angptl8	176-184	10 min	0.244	0.033	0.033	FALSE
Angptl8	176-184	60 min	0.218	-0.095	0.095	FALSE
Angptl8	177-190	10 s	0.223	-0.169	0.169	FALSE
Angptl8	177-190	2 min	0.337	-0.352	0.352	TRUE
Angptl8	177-190	10 min	0.458	-0.318	0.318	FALSE
Angptl8	177-190	60 min	0.663	-0.361	0.361	FALSE
Angptl8	178-190	10 s	0.112	-0.067	0.067	FALSE
Angptl8	178-190	2 min	0.638	-0.096	0.096	FALSE
Angptl8	178-190	10 min	0.352	-0.134	0.134	FALSE
Angptl8	178-190	60 min	0.480	-0.217	0.217	FALSE
Angptl8	178-191	10 s	0.217	-0.073	0.073	FALSE
Angptl8	178-191	2 min	0.366	-0.193	0.193	FALSE
Angptl8	178-191	10 min	0.381	-0.131	0.131	FALSE
Angptl8	178-191	60 min	0.397	-0.135	0.135	FALSE
Angptl8	178-198	10 s	0.348	-0.171	0.171	FALSE
Angptl8	178-198	2 min	0.661	-0.266	0.266	FALSE
Angptl8	178-198	10 min	0.741	-0.125	0.125	FALSE
Angptl8	178-198	60 min	0.736	-0.235	0.235	FALSE
Angptl8	185-198	10 s	0.303	-0.084	0.084	FALSE

Protein	peptide	Exposure	U=ku k=2	Δ (HX)t	ABS(T)	True = significant difference, reject Null (no difference)
Angptl8	185-198	10 min	0.634	-0.042	0.042	FALSE
Angptl8	185-198	60 min	0.600	-0.121	0.121	FALSE

Figure S6

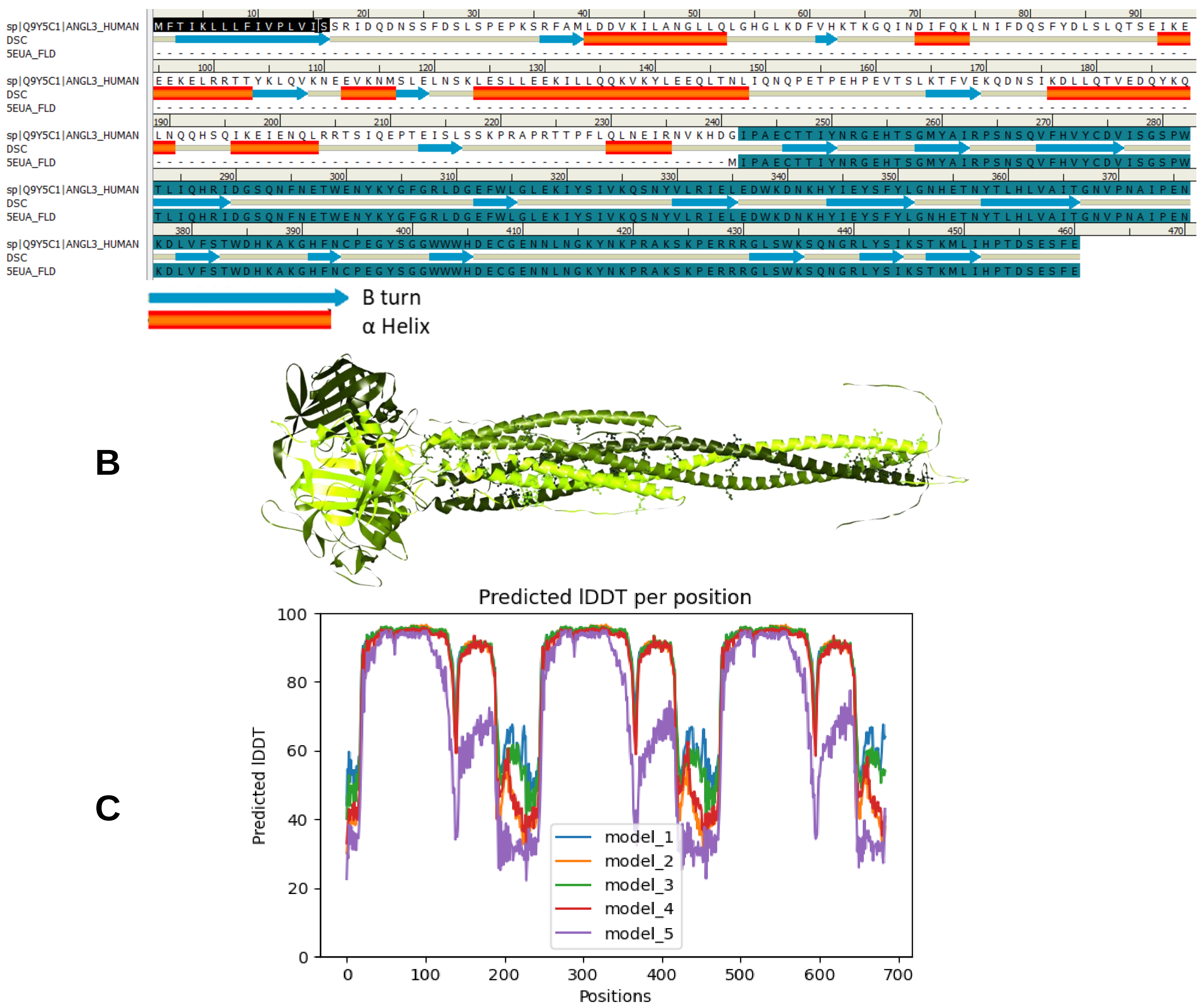

Figure S6. Modeling of ANGPTL3. (A) Full-length amino acid sequence of ANGPTL3, sp|Q9Y5C1| showing amino acids 1-460 aligned with the sequence from crystal structure 6EUA for fibrinogen-like domain (FLD) amino acids annotated by secondary structure in Discovery Studio (1-16 signal sequence on black background, 17-210 coiled coil domain, 241-460 fibrinogen-like domain). (B) Amino acids 17-210 were predicted by multiple models including AlphaFold2 as a coiled coil domain (CCD) from amino acids 34-153, followed by the turn and a second CCD from amino acids 159-210. The LOGICOIL algorithm predicted a trimer as the most probable assembly, leading us to build a model for the ANGPTL3 trimer using AlphFold2 for the CCDs and the 6EUA ANGPTL3 fibrinogen-like domain (FLD) trimer solved by X-ray diffraction. (C) Five predicted models are superimposed displaying high consistency of the prediction and IDDT score, a local superposition-free score for comparing protein structures and models using distance difference tests.

Figure S7

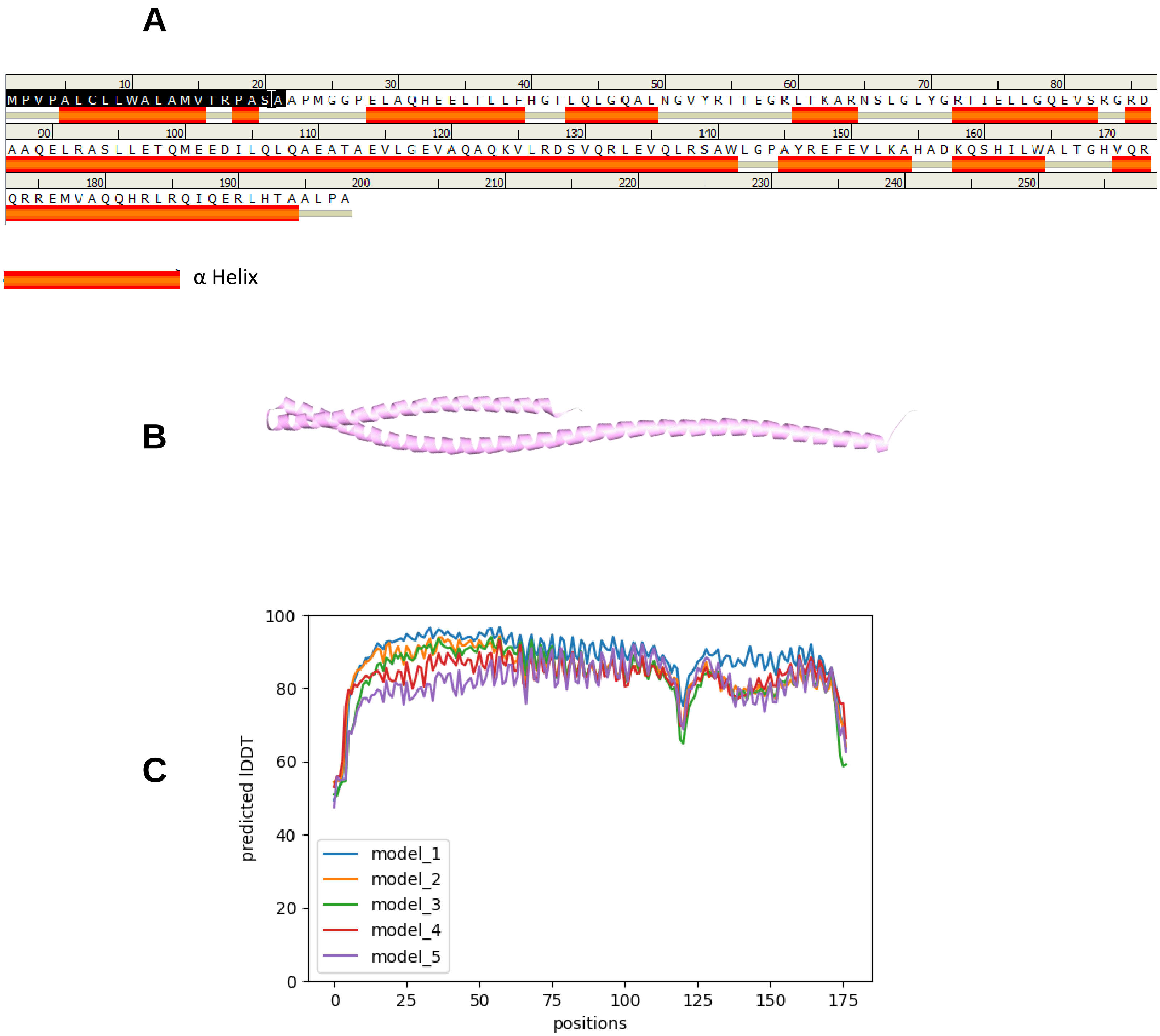

Figure S7. ANGPTL8 modeling. (A) Full length amino acid sequence of ANGPTL8, sp|Q9Y5C1| showing amino acids 1-198 (1-21 signal sequence on the black background, 22-198 as coiled coil domain). (B) Amino acids 21 to 198 were predicted by multiple models including AlphaFold2 as a coiled coil domain (CCD) structure with a turn from amino acids 120-124. (C) Five predicted models were superimposed showing consistency of the prediction supported by IDDT, a local superposition-free score for comparing protein structures and models using distance difference tests.

Figure S7 Continued

D

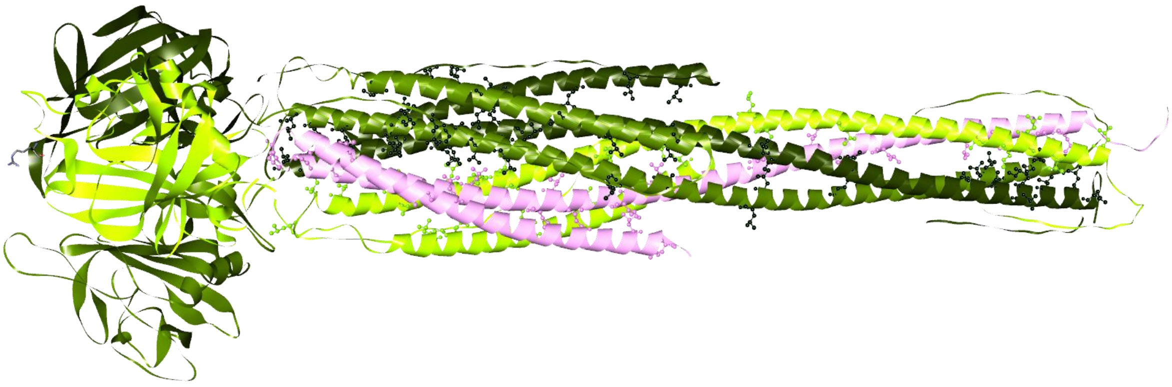

E

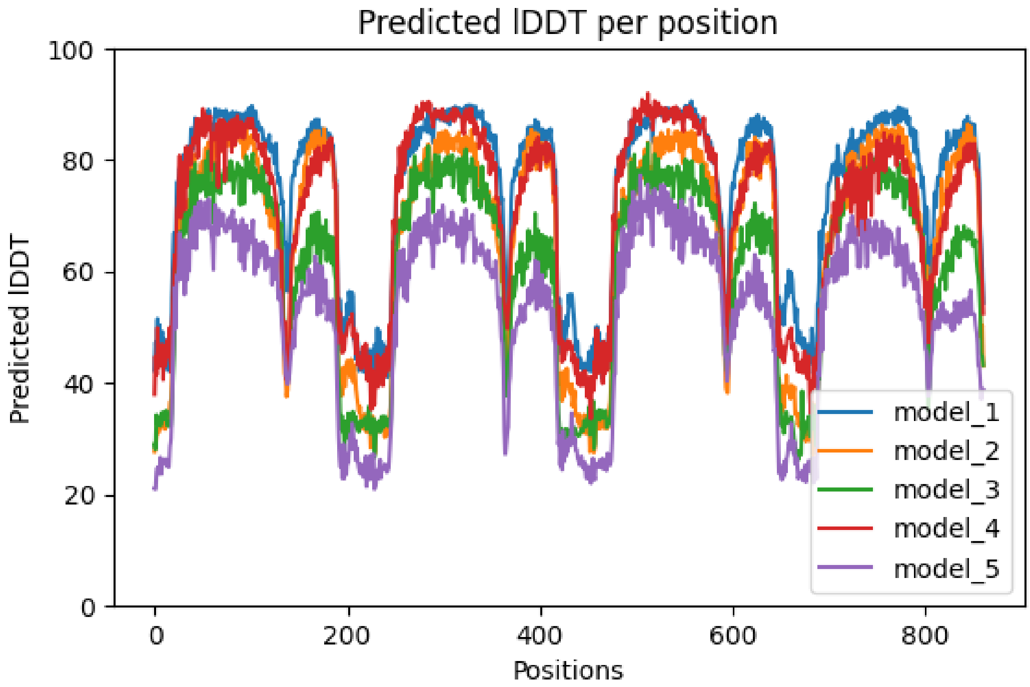

Figure S7 Continued. ANGPTL3/ANGPTL8 modeling. (D) Full-length AlphaFold2 model of the ANGPTL3 trimer together with the ANGPTL8 monomer, comprising the ANGPTL3/8 complex. (E) Five predicted models were superimposed showing consistency of the prediction supported by lDDT, a local superposition-free score for comparing protein structures and models using distance difference tests that evaluate local distance differences of all atoms in a model (including validation of stereochemical plausibility) for CCDs of the ANGPTL3 trimer followed by ANGPTL8 scoring.

Figure S8

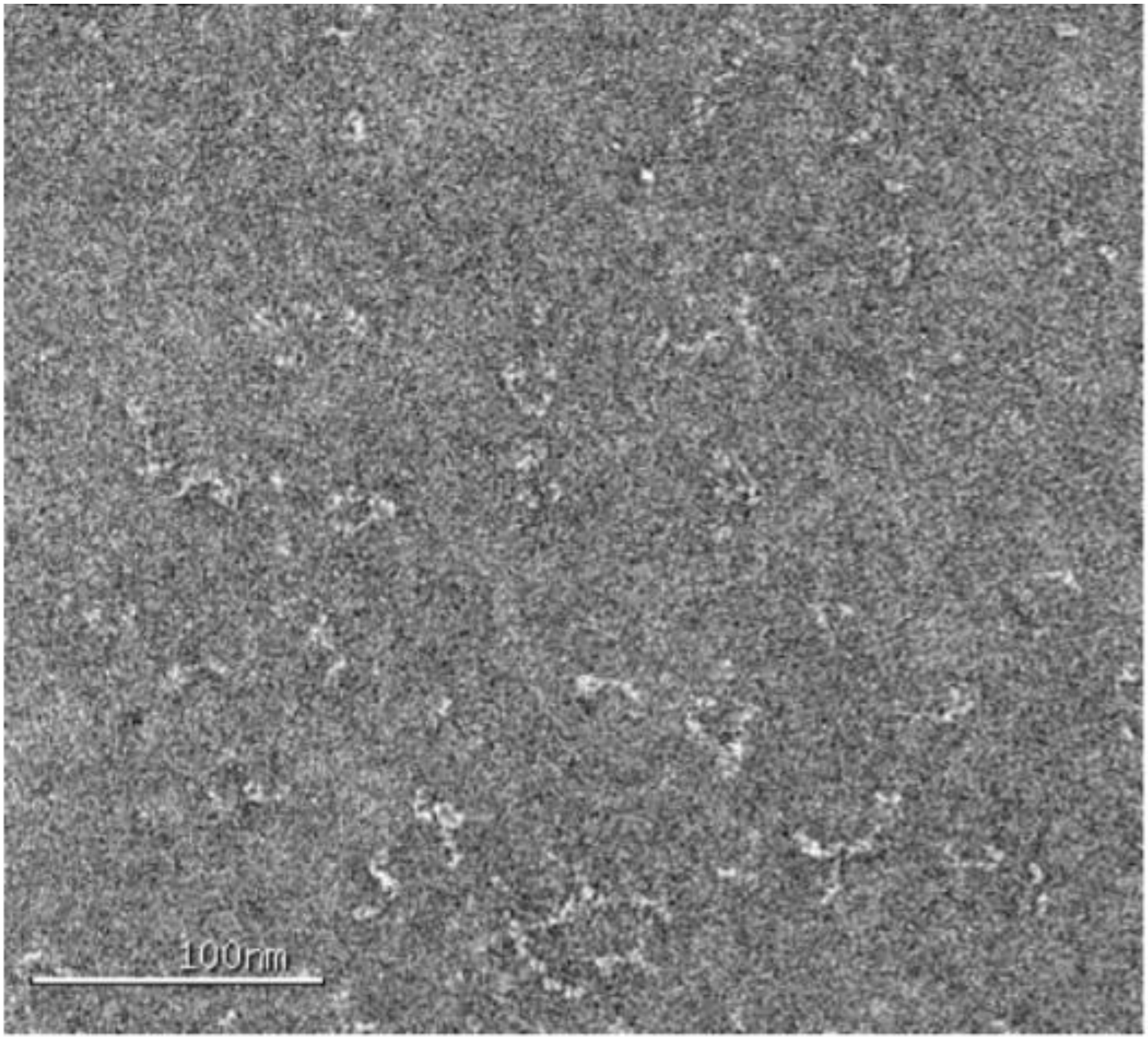

Figure S8: Negative stain transmission electron microscopy (TEM) image. Thumbnail images of the anti-ANGPTL3/8 antibody Fab bound to the ANGPTL3/8 complex were obtained by negative stain TEM at a magnification of 110,000x. The scale bar corresponds to 100 nm. The limit of resolution is approximately 1-2 nm.

Figure S9

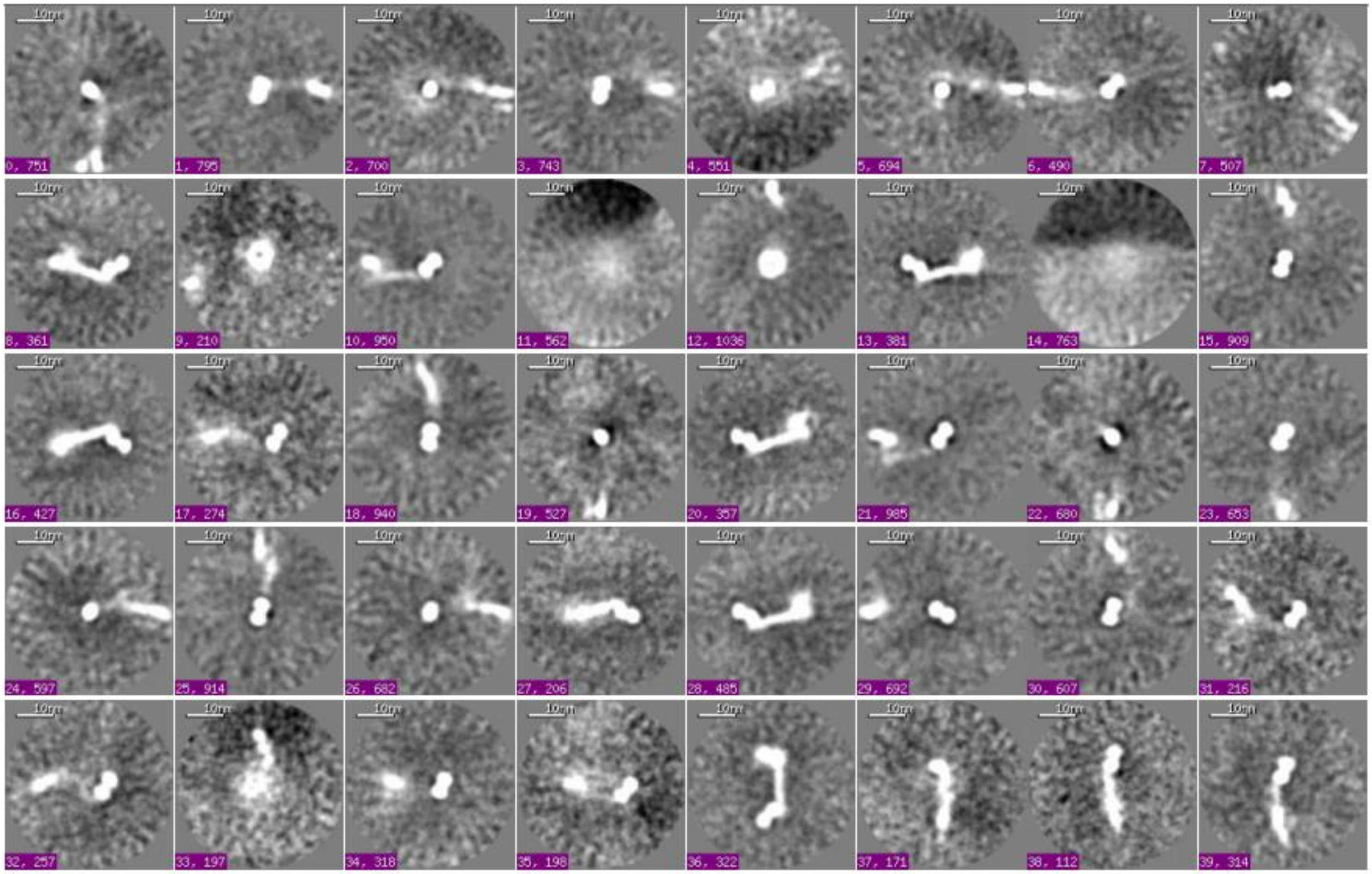

Figure S9: Thumbnails showing class averages obtained for the first round of alignments obtained by negative stain TEM. Thumbnail images of the anti-ANGPTL3/8 antibody Fab bound to the ANGPTL3/8 complex were obtained by negative stain TEM. The first number at the bottom of each particle states the class number. The second number states the number of particles contributing to that class.

Figure S10

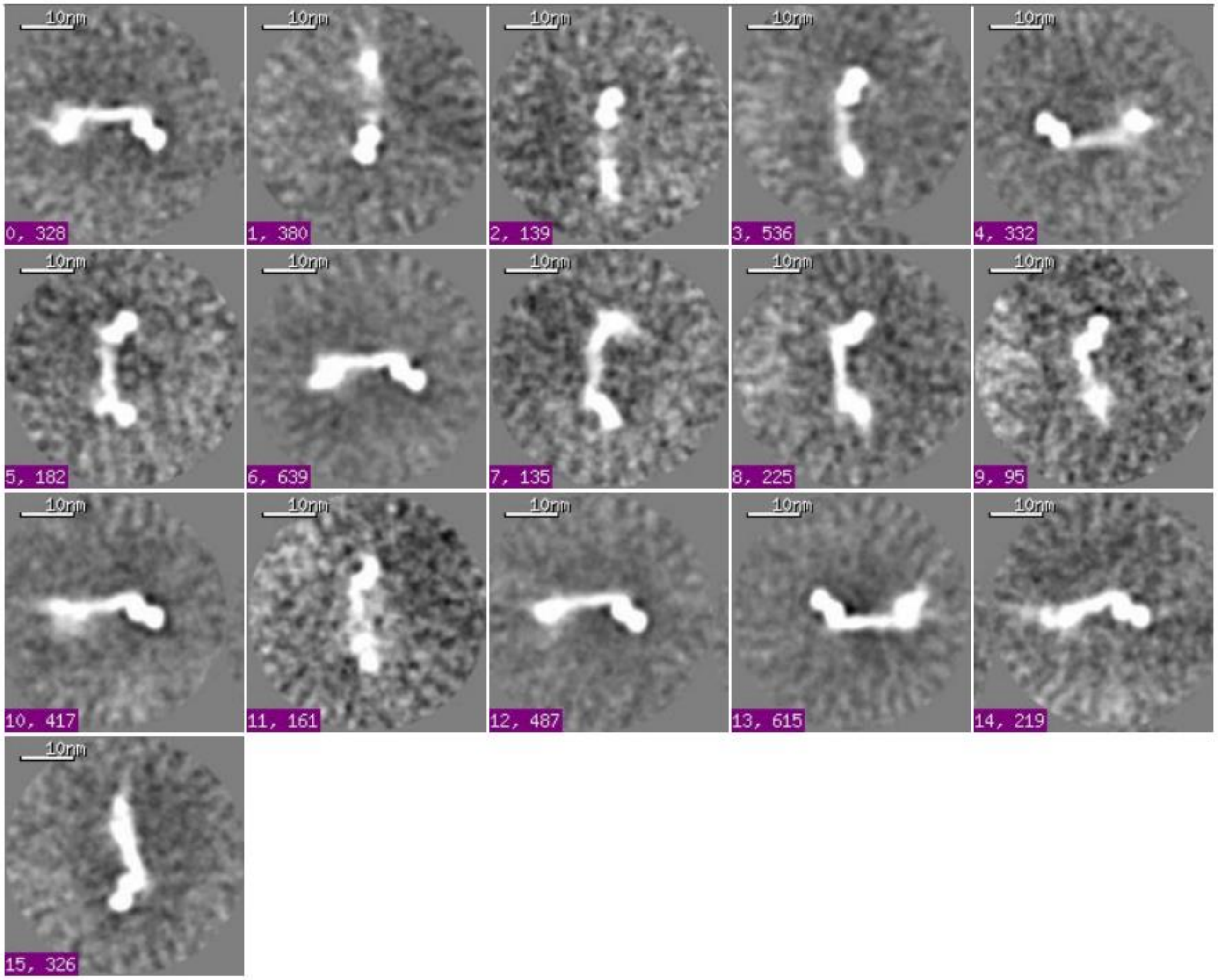

Figure S10: Thumbnails showing class averages obtained for the second round of alignments obtained by negative stain TEM. Thumbnail images of the anti-ANGPTL3/8 antibody Fab bound to the ANGPTL3/8 complex were obtained by negative stain TEM. The first number at the bottom of each particle states the class number. The second number states the number of particles contributing to that class.

Figure S11

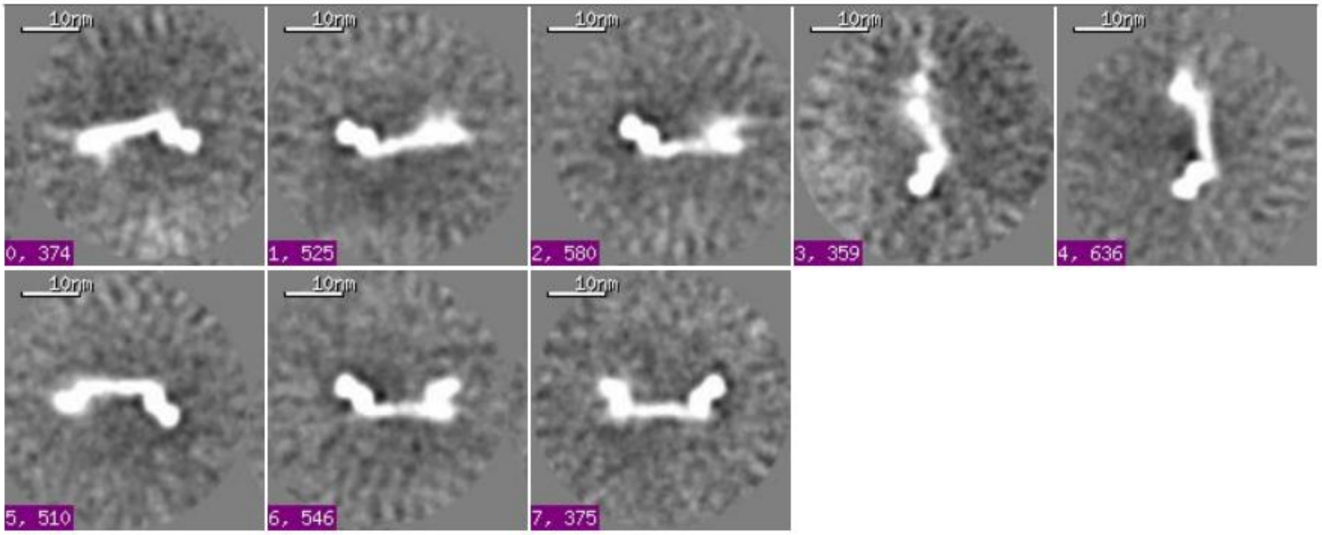

Figure S11: Thumbnails showing class averages obtained for the third round of alignments obtained by negative stain TEM. Thumbnail images of the anti-ANGPTL3/8 antibody Fab bound to the ANGPTL3/8 complex were obtained by negative stain TEM. The first number at the bottom of each particle states the class number. The second number states the number of particles contributing to that class.

Figure S12

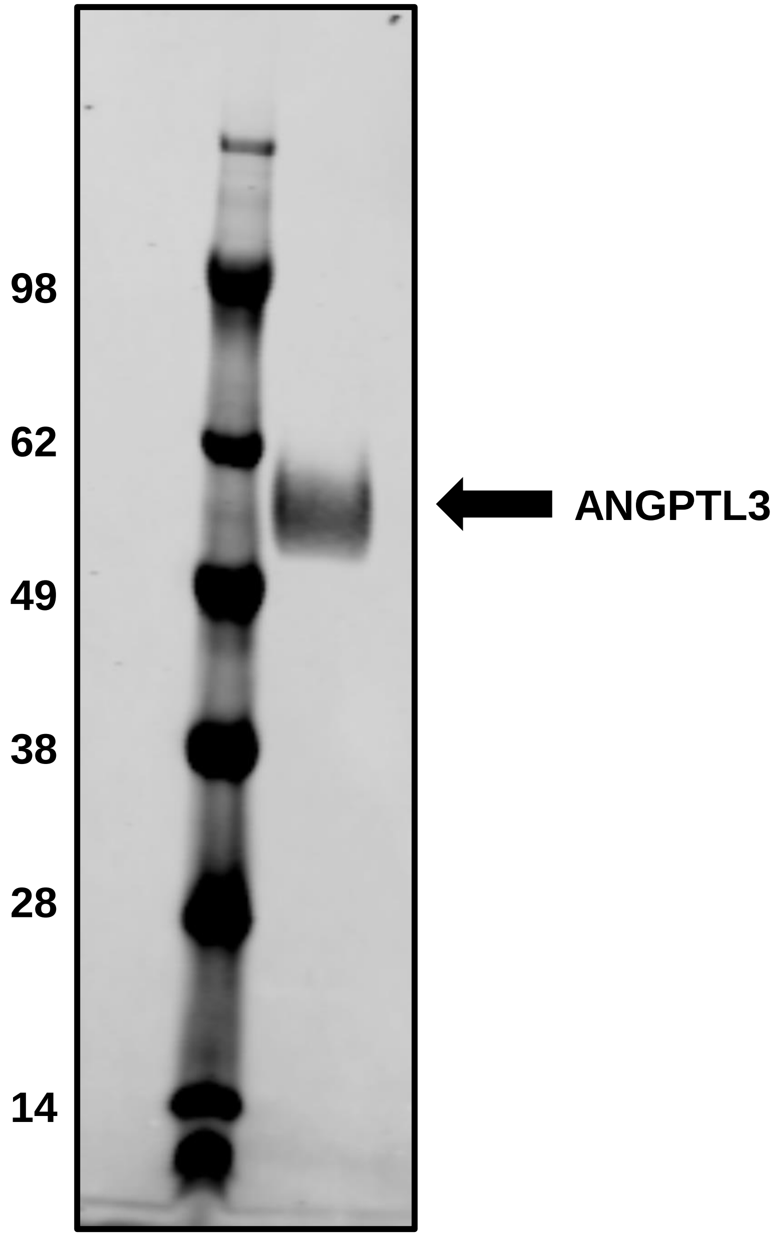

Figure S12: Analysis of ANGPTL3 in the ANGPTL3/8 complex by Western blotting. Ten picomoles of the ANGPTL3/8 complex were separated on a Novex 12% Bis-Tris gel and transferred to PVDF using an iBlot system (Thermo). Membranes were probed with a biotinylated anti-C-terminal ANGPTL3 antibody that recognized the fibrinogen-like domain (FLD) of ANGPTL3. Visualization was performed with Alexa Fluro 680-conjugated streptavidin. Images were recorded using an Odyssey CLx image system (LI-COR Biosciences). The presence of a single band corresponding to full-length ANGPTL3 indicated that ANGPTL3 molecules in the ANGPTL3/8 complex are intact and not cleaved.

Table S6

Amino acid at position 105 of HC	Binding ELISA OD
W	1.723
A	0.054
D	0.059
E	0.079
F	0.070
G	0.054
H	0.057
I	0.053
K	0.056
L	0.056
M	0.306
N	0.079
P	0.067
Q	0.060
R	0.060
S	0.058
T	0.063
V	0.065
Y	0.092

Table S6: Saturated mutagenesis for W105 in CDR3 of the heavy chain of the anti-ANGPTL3/8 antibody. Saturated mutagenesis using standard site-directed mutagenesis technology was undertaken to investigate every amino acid substitution (except Cys). In the binding ELISA, biotinylated antigen was added to allow for antibody/antigen binding, and antibody/antigen complexes were detected via colorimetric absorbance. The table shows the SPE data for mutagenesis at heavy chain CDR3 position W105, indicating loss of appreciable binding for all tested substitutions. In each case, binding to the ANGPTL3/8 complex was dramatically reduced.

Figure S13

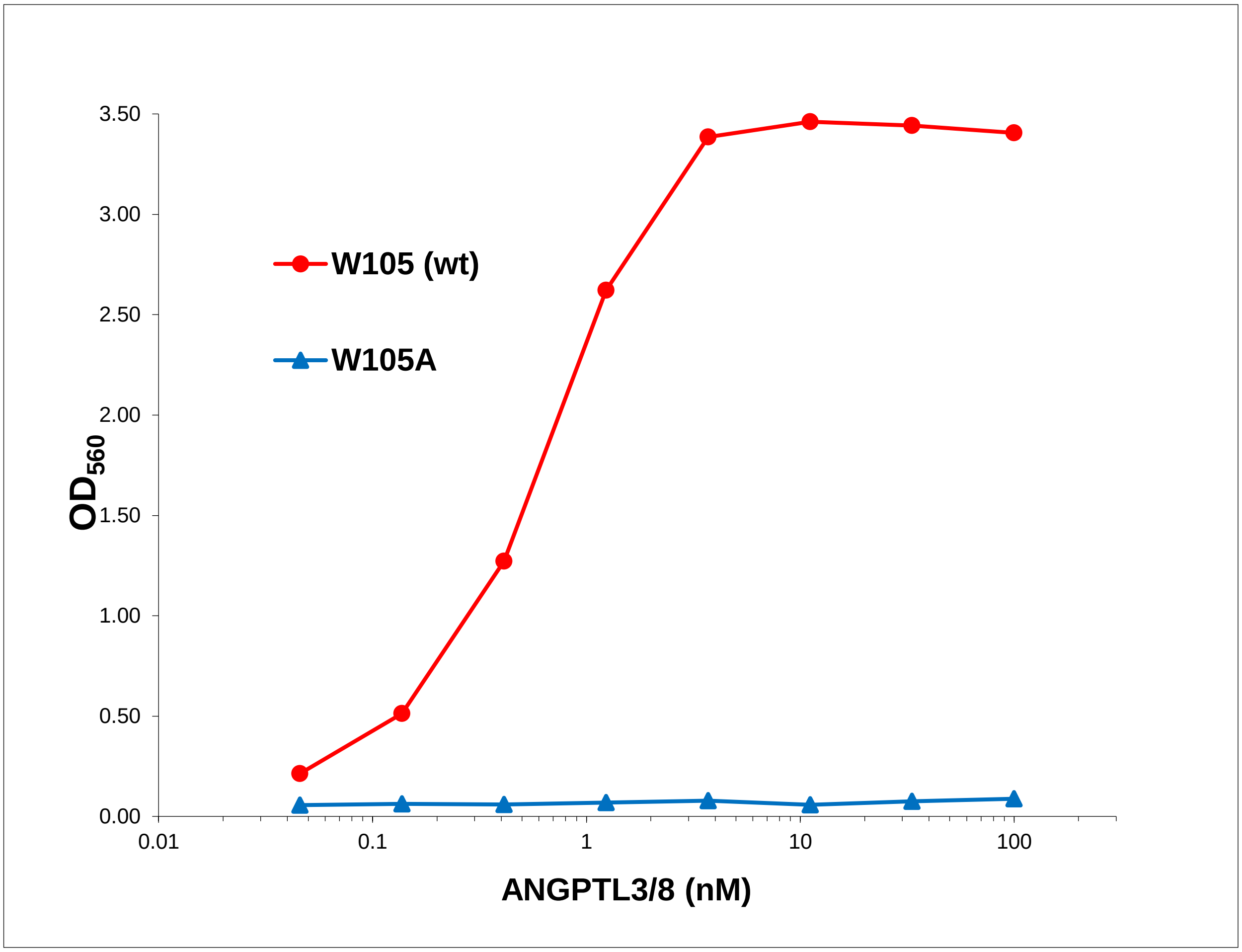

Figure S13: Titration binding studies for W105A variant of the anti-ANGPTL3/8 antibody. Additional antigen titration binding studies confirmed loss of binding to the ANGPTL3/8 complex for the variant W105A (removal of the aromatic side chain at position 105 of the heavy chain). The figure demonstrates complete loss of binding activity for W105A.
